# Large-Scale Identification of Mirtrons in *Arabidopsis* and Rice

**DOI:** 10.1371/journal.pone.0031163

**Published:** 2012-02-13

**Authors:** Yijun Meng, Chaogang Shao

**Affiliations:** 1 College of Life and Environmental Sciences, Hangzhou Normal University, Hangzhou, People's Republic of China; 2 College of Life Sciences, Huzhou University, Huzhou, People's Republic of China; University of Toronto, Canada

## Abstract

A new catalog of microRNA (miRNA) species called mirtrons has been discovered in animals recently, which originate from spliced introns of the gene transcripts. However, only one putative mirtron, osa-MIR1429, has been identified in rice (*Oryza sativa*). We employed a high-throughput sequencing (HTS) data- and structure-based approach to do a genome-wide search for the mirtron candidate in both *Arabidopsis* (*Arabidopsis thaliana*) and rice. Five and eighteen candidates were discovered in the two plants respectively. To investigate their biological roles, the targets of these mirtrons were predicted and validated based on degradome sequencing data. The result indicates that the mirtrons could guide target cleavages to exert their regulatory roles post-transcriptionally, which needs further experimental validation.

## Introduction

MicroRNAs, the well-known small RNA (sRNA) species of ∼21-nucleotide (nt) in length, play essential regulatory roles in gene expression in a vast range of organisms [1]–[3]. Different from the miRNA genes in animals [1], [4], which were mainly discovered within the introns or the exons of the coding or non-coding genes, most plant miRNAs were found to reside in the intergenic regions [5]. Generally, the primary transcripts of the miRNA genes should be subjected to two sequential cleavages by Drosha and Dicer in animals [1], [4], or Dicer-like 1 (DCL1) in plants [5], in order to generate functional mature miRNA molecules for post-transcriptional gene silencing (PTGS). However, some non-canonical biogenesis pathways have been discovered. Recently, a novel class of miRNAs named as mirtrons was widely recognized in animals [4], [6]–[14]. These miRNAs are generated from the spliced and subsequently debranched introns of the gene transcripts. Making them quite distinguishable from the other miRNA genes resided with the intronic regions, the mirtrons are processed through a Drosha-independent pathway [4], [8], [11], [13]. After Dicing, the miRNAs are separated from the miRNA/miRNA* duplexes, and incorporated into the Argonaute (AGO)-associated miRNA-induced silencing complexes (miRISCs) to mediate PTGS.

To date tens of mirtron genes have been uncovered in several animal species, such as *Drosophila melanogaster*, *Caenorhabditis elegans*, *Gallus gallus* and mammals [6], [7], [9], [10], [12], [13]. However, only one putative mirtron, osa-MIR1429, was identified in rice [15]. Thus, the question whether the mirtrons are also widespread in plants as in animals remain to be addressed. In this study, we interrogated this issue in both *Arabidopsis* and rice. Taking advantage of sRNA HTS data and secondary structure prediction, five and eighteen mirtron candidates were discovered in the two plants, respectively. The biological relevance of these mirtrons was primarily illustrated through degradome data-based target identification. Taken together, we did a first systemic search for the mirtron genes in the two model plants, and hoped that these findings could inspire further research efforts on this interesting topic.

## Results and Discussion

### Genome-wide Identification of Mirtron Candidates

The two model plants, *Arabidopsis* and rice, with well-annotated genomes were selected for this study. Considering the fact that the sequences of the currently registered plant miRNA precursors [according to miRBase (Release 17) [16]] are generally less than 300 nt in length (Figure S1), the introns with this length range were selected for secondary structure prediction by using RNAshapes [17]. Since almost all the canonical miRNA precursors could form simple stem-loop structures, the simplest structure (with a single hairpin structure in most cases) among the prediction results of an intron was selected for manual check. The ones capable of forming stable hairpin structures were retained for further filtering.

Then all the short reads from the retrieved sRNA HTS data sets were mapped onto these intron sequences, and all the perfectly matched ones were retained. The findings in animals demonstrated that the processing of the mirtron precursors could bypass the Drosha cleavages [13], which were required for canonical miRNA maturation [4]. Additionally, another two kinds of mirtrons, i.e. 5′ and 3′ tailed introns, were identified in mouse [13], [18]. Different from the canonical mirtrons as mentioned above, the processing of these mirtron precursors requires 5′-to-3′ and 3′-to-5′ trimming before they are subjected to Dicing respectively [14]. In this regard, the mirtron-generating introns were retained, and were classified into three catalogues (i.e. “match both ends”, “match 5′ end”, and “match 3′ end”; see “Materials and Methods” for details) based on HTS data mapping results.

**Figure 1:**
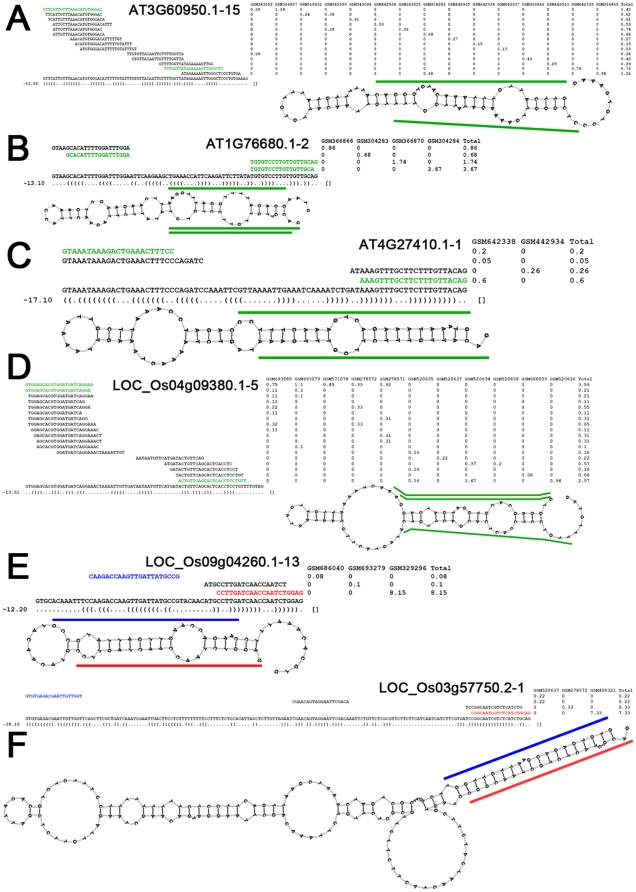
High-throughput sequencing (HTS) data- and structure-based identification of mirtrons in *Arabidopsis* and rice. (A) A “match 5′ end” mirtron resided within the 15^th^ intron of AT3G60950.1. (B) A “match 3′ end” mirtron resided within the second intron of AT1G76680.1. (C) A “match both ends” mirtron resided within the first intron of AT4G27410.1. (D) A “match 5′ end” mirtron resided within the 5^th^ intron of LOC_Os04g09380.1. (E) A “match 3′ end” mirtron resided within the 13^th^ intron of LOC_Os09g04260.1. (F) A “match both ends” mirtron resided within the first intron of LOC_Os03g57750.2. For all the panels, the short reads perfectly mapped to the mirtron precursors along with their normalized read counts in RPM (reads per million) are shown (see Table S1 for the small RNA HTS data sources and see “Materials and Methods” for read count normalization). The mature mirtrons with significantly higher expression levels compared to the coordinates on the other arms were highlighted in red color, and the coordinates were in blue. For the mirtron precursors generating mirtrons with indistinguishable expression levels on both arms, their mature mirtrons were highlighted in green color. The mature mirtrons and their coordinates were also indicated in the stem-loop structures of their precursors. The parenthesis-dot formed secondary structure expression along with the free energy, and the stem-loop structures were all predicted and generated by RNAshapes [17].

Although the canonical miRNA/miRNA* duplex possesses 2-nt 3′ overhangs at both ends which result from Drosha/Dicer- (in animals) or DCL1- (in plants) mediated cropping [1], [4], [5], recent studies on mirtrons in animals showed a wide-spread scene of unusual configuration of the duplex overhangs [6]. Thus, the mature mirtron duplex candidates were selected from the perfectly matched short reads (see “Materials and Methods” for details), and mapped onto the secondary structures of the corresponding introns for manual check. Only the introns that could generate short mirtron duplexes with 0 to 3-nt 3′ overhangs at both ends were finally considered to be the mirtron candidates. As a result, five and eighteen mirtron candidates were identified in *Arabidopsis* and rice, respectively (Figure 1 and Figure S2). The previously reported putative mirtron in rice, osa-MIR1429 [15], was also uncovered in this study, indicating the reliability of our HTS- and structure-based filtering criteria. Notably, one out of the five mitrons in *Arabidopsis*, and 12 out of 18 in rice reside within the introns of the transposable element (TE) genes (Table S3 and S4). Thus, whether the introns embedded within the TE genes are the hotspots for the birth mirtrons especially in rice needs to be investigated. Moreover, different from the canonical miRNA/miRNA* duplexes, a dominant portion of the mirtrons (17 out of 23) generate mature mirtron duplexes with indistinguishable expression levels. Thus, it is hard to tell the mature and the star species separately. Except for the putative mirtron osa-MIR1429, nearly all the mirtrons are expressed at considerably low levels with normalized read counts less than 10 RPM (reads per million) (Figure S2). Many newly evolved miRNA genes with low conservation were also observed to be expressed weakly [19]. From this point of view, whether the mirtron candidates identified here have evolved recently, and whether they could serve as a novel source for new canonical miRNA genes through a TE gene-mediated pathway need to be studied.

### Degradome Sequencing Data-based Identification of Mirtron Targets

Since the expression levels of most mature mirtrons generated from 5′ and 3′ arms of the precursors are indistinguishable, all the mature mirtron candidates identified on the stem-loop-structured precursors (all were marked in Figure S2; see sequence lists in Table S3 and S4) were recruited for functional analysis.

**Figure 2:**
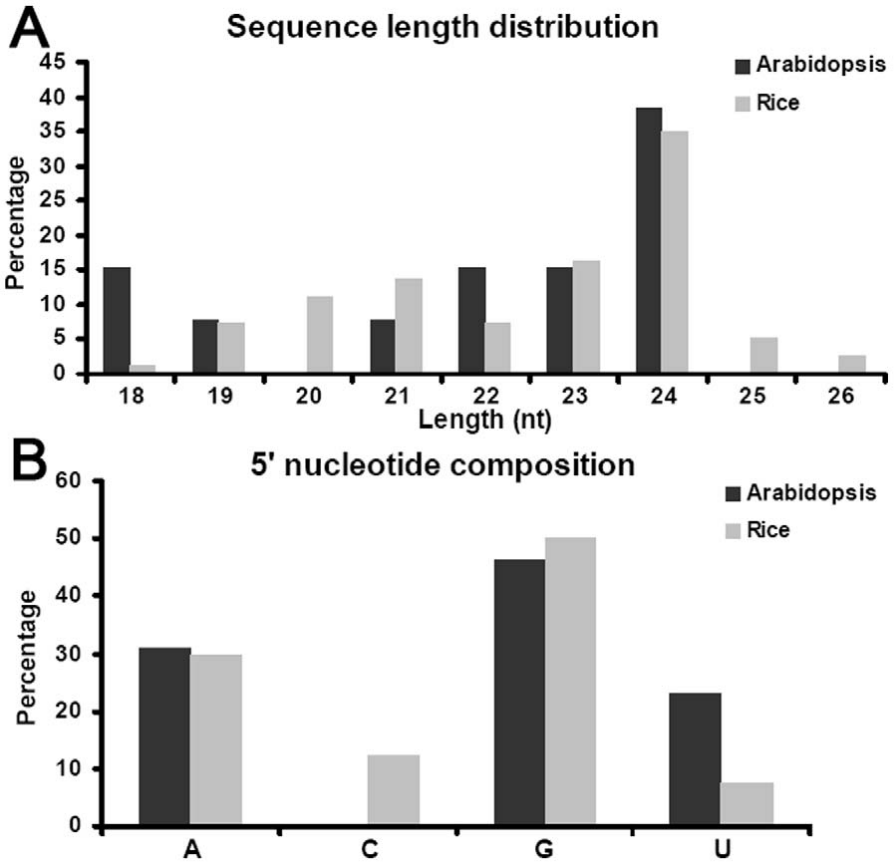
Sequence characteristics of the mature mirtrons identified in *Arabidopsis* and rice. (A) Sequence length distribution patterns. (B) 5′ terminal nucleotide compositions. See Table S3 and S4 for sequence information.

First, the sequence characteristics of all the mature mirtrons were analyzed. Different from the ∼21-nt miRNAs predominantly starting with 5′ U (uridine), a dominant portion of mature mirtrons are 24 nt in length, and begin with 5′ G (guanine) and 5′ A (adenosine) in both *Arabidopsis* and rice (Figure 2).

**Figure 3:**
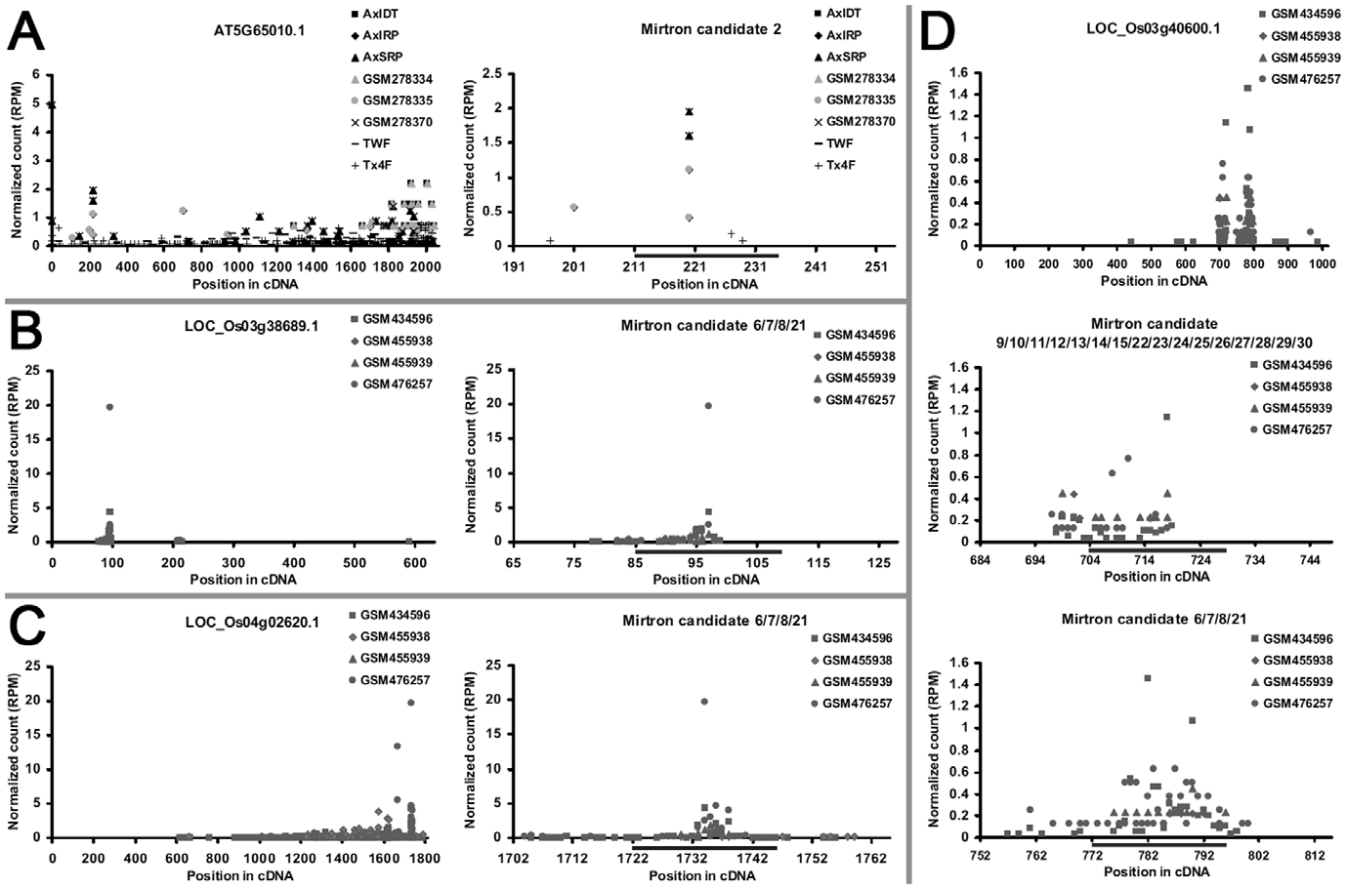
Degradome sequencing data-based identification of the targets of the mature mirtrons in *Arabidopsis* and rice. For all the sub-figures (A to D), the first panels depict the degradome signals all along the target transcripts, and the other panels provide detailed views of the cleavage signals within the regions surrounding the target recognition sites (denoted by gray horizontal lines). The transcript IDs are shown in the first panels, and the mirtron IDs are listed in the other panels (see Table S3 and S4 for the sequence information corresponding to the mirtron IDs). The *x* axes measure the positions of the signals along the transcripts, and the *y* axes measure the signal intensities based on normalized counts (in RPM, reads per million), allowing cross-library comparison. See Table S2 for the degradome data sets used in this analysis.

A transcriptome-wide target prediction was performed by using miRU algorithm [20], [21]. Then, degradome sequencing data-based validation of these predicted targets was carried out by employing t-plot (target plot)-based approach [22], [23] (see details in “Materials and Methods”). As a result, dozens of mirtron—target pairs were identified, most of which were supported by compelling cleavage signals in the middle of the target recognition sites of the mature mirtrons (Figure 3, Figure S3 and S4, and Table S5 and S6), indicating their target cleavage-based role in PTGS. Intriguingly, some targets in rice, such as LOC_Os03g40600.1 (Figure 3D), LOC_Os02g48390.1, and LOC_Os04g45665.1 (Figure S4), were found to be cleaved at two different sites of the transcripts by distinct mirtrons, which could be defined as mirtron-mediated co-regulation. It is likely that the co-regulation mechanism could serve to enhance the efficiency of the mirtron-involved PTGS, although it still needs experimental validation.

According to the gene annotations provided by TAIR and TIGR rice, only a few targets of rice mirtron encode transcription factors (TFs) (Table S6), which is different from the plant miRNAs that target numerous TF genes [3]. Instead, several mirtron targets were annotated to be involved in RNA metabolism in rice (e.g. LOC_Os03g62080.1, LOC_Os06g31210.1, and LOC_Os07g46600.1), and photosynthesis in both plants (e.g. AT2G30570.1 in Arabidopsis, and LOC_Os02g10390.1/2/3 in rice) (see details in Table S5 and S6). More interestingly, three target genes in *Arabidopsis* (AT1G36360.1, AT4G10460.1, and AT5G33386.1) and four in rice (LOC_Os01g05030.1, LOC_Os01g22770.1, LOC_Os04g16010.1, and LOC_Os09g09820.1) encode TEs (see details in Table S5 and S6). Considering the origination of many mirtrons from the introns of TE genes (e.g. Arab_mirtron_candidate_10/11 from AT4G05280.1; Rice_mirtron_candidate_1/2 from LOC_Os03g36170.1, Rice_mirtron_candidate_3/4/5 from LOC_Os04g09380.1, Rice_mirtron_candidate_16/17/18/19/20 from LOC_Os07g32220.1, etc. See details in Table S3 and S4), this observation raised a feedback regulatory circuit that TEs might be hotspots for mirtron generation, and in turn, the corresponding mirtrons could strictly modulate the expression of these TE genes at the transcriptional or the post-transcriptional level.

Taken together, the mirtrons and their targets identified based on our bioinformatics approach provide a basis for further experimental studies on the origin and the functions of the mirtrons in plants.

## Materials and Methods

### Data Sets Used in this Study

The sRNA HTS data sets of *Arabidopsis* and rice were retrieved from GEO (Gene Expression Omnibus; http://www.ncbi.nlm.nih.gov/geo/) [24] and CSRDB (Cereal Small RNAs Database; http://sundarlab.ucdavis.edu/smrnas/) [25]. See Table S1 for the accession numbers. The degradome sequencing data sets were retrieved from GEO and NGSDBs (Next-Gen Sequence Databases; http://mpss.udel.edu/) [26]. See Table S2 for the accessions. The gene annotation and sequence information of *Arabidopsis* and rice were retrieved from the FTP sites of The *Arabidopsis* Information Resource (TAIR, Release 7 and 10; ftp://ftp.arabidopsis.org/home/tair/Sequences/blast_datasets/) [27] and the rice genome annotation project established by The Institute for Genome Research (currently named the J. Craig Venter institute) (TIGR rice, Release 5 and 6.1; ftp://ftp.plantbiology.msu.edu/pub/data/Eukaryotic_Projects/o_sativa/annotation_dbs/pseudomolecules/) [28], respectively. The miRNA precursor sequences of the 15 plant species were downloaded from miRBase (Release 17; http://www.mirbase.org/) [16].

### Secondary Structure- and HTS Data-based Identification of Mirtron Candidates

Considering the average length of the pre-miRNAs (precursor microRNAs) of the currently annotated miRNA genes [according to miRBase (Release 17; http://www.mirbase.org/) [16]], the intron sequences retrieved from TAIR and TIGR rice that less than 300 nt in length were subjected to secondary structure prediction by using RNAshapes [17] in “Shape folding” mode with default parameters. The simplest structure (with single stem-loop region in most cases) among all the predicted results of an intron sequence was selected for manual check. The introns capable of forming stable hairpin-like structures were retained. Then, the sRNA HTS data were mapped onto these selected introns by BLAST algorithm [29], and all the perfectly matched ones were retained. In order to allow cross-library comparison, the normalized read count (in RPM, reads per million) of a short read from a specific library was calculated by dividing the raw count of this read by the total counts of the library, and then multiplied by 10^6^. According to the mapping results, the stem-loop-structure introns were classified into three categories: (1) “Match both ends”: the introns with HTS short reads perfectly mapped onto both the 5′ and the 3′ ends; (2) “Match 5′ end”: with reads mapped onto the 5′ ends of the introns; (3) “Match 3′ end”: with reads mapped onto the 3′ ends of the introns. Finally, the mature mirtron candidates along with their partners on the other ends were selected from the short reads clusters on the corresponding introns, and mapped onto the stem-loop structures for manual check. For the “match both ends” introns, the short reads mapped to the two ends of the introns were selected as mature mirtron candidates. For the “match 5′ end” and the “match 3′ end” introns, the reads mapped to the 5′ ends and the 3′ ends of the introns were considered as one of the mature mirtron candidates of the corresponding introns, respectively. And, the remaining candidates on the other arms of the stem-loop-structured introns were selected manually. For all three categories of the introns, the ones possessing pairs of mature mirtron candidates with 0 to 3-nt 3′ overhangs at both ends were finally considered to be the mirtron candidates.

### Prediction and Validation of the Targets of the Mirtrons

Target prediction was performed by using miRU algorithm [20], [21] with default parameters. The degradome sequencing data were utilized to validate the predicted mirtron—target pairs. First, the read counts of all the degradome reads from each library were normalized as described in the above section. Then, two-step filtering was performed to extract the most likely mirtron—target pairs. During the first step, the predicted mirtron binding sites along with the 50-nt surrounding sequences at both ends were collected in order to reduce the BLAST time. For the BLAST, all the collected degradome data sets (eleven of *Arabidopsis* and four of rice; see Table S2) were utilized at the same time to do a comprehensive search. It was based on the scenario that a mirtron—target pair was considered to be the candidate once the cleavage signal(s) existed in any data set(s). Two types of predicted targets were retained for further filtering: (1) there must be perfectly matched degradome reads with their 5′ ends resided within 8–14 nt region away from the 5′ ends of the target binding sites; or (2) the target transcripts should possess degradome reads at least partially located within the target binding sites, and their normalized counts should be significantly higher than the surrounding signals. These transcripts were subjected to a second BLAST, and the degradome signals along each transcript were obtained to provide a global view of the signal noise when compared to the signal intensity within a specific target binding site. Referring to our previous study [30], both the global and the local t-plots were drawn. Exhaustive manual filtering was performed, and only the transcripts with cleavage signals easy to be recognized were extracted as the potential mirtron—target pairs.

## Supporting Information

Figure S1 **Sequence length distribution of the miRBase-registered microRNA precursors belonging to 15 plant species.** The *x* axis marks the sequence length range, and the *y* axis measures the percentage of the microRNA precursors resided within a specific length range in a plant.
(PDF)

Figure S2 **All the mirtrons in** ***Arabidopsis*** **and rice.** The short reads perfectly mapped to the mirtron precursors along with their normalized read counts in RPM (reads per million) are shown (see Table S1 for the small RNA HTS data sources and see METHODS in the text for read count normalization). The mature mirtrons with significantly higher expression levels compared to the coordinates on the other arms were highlighted in red color, and the coordinates were in blue. For the mirtron precursors generating mirtrons with indistinguishable expression levels on both arms, their mature mirtrons were highlighted in green color. The mature mirtrons and their coordinates were also indicated in the stem-loop structures of their precursors. The parenthesis-dot formed secondary structure expression along with the free energy, and the stem-loop structures were all predicted and generated by RNAshapes (Steffen *et al.*, 2006).
(PDF)

Figure S3 **Degradome sequencing data-based identification of the targets of the mature mirtrons in** ***Arabidopsis*** **.** For all the sub-figures, the first panels depict the degradome signals all along the target transcripts, and the other panels provide detailed views of the cleavage signals within the regions surrounding the target recognition sites (denoted by blue horizontal lines). The transcript IDs are shown in the first panels, and the mirtron IDs are listed in the other panels (see Table S3 and S4 for the sequence information corresponding to the mirtron IDs). The *x* axes measure the positions of the signals along the transcripts, and the *y* axes measure the signal intensities based on normalized counts (in RPM, reads per million), allowing cross-library comparison. See Table S2 for the degradome data sets used in this analysis.
(PDF)

Figure S4 **Degradome sequencing data-based identification of the targets of the mature mirtrons in rice.** For all the sub-figures, the first panels depict the degradome signals all along the target transcripts, and the other panels provide detailed views of the cleavage signals within the regions surrounding the target recognition sites (denoted by blue horizontal lines). The transcript IDs are shown in the first panels, and the mirtron IDs are listed in the other panels (see Table S3 and S4 for the sequence information corresponding to the mirtron IDs). The *x* axes measure the positions of the signals along the transcripts, and the *y* axes measure the signal intensities based on normalized counts (in RPM, reads per million), allowing cross-library comparison. See Table S2 for the degradome data sets used in this analysis.
(PDF)

Table S1 **Plant small RNA high-throughput sequencing data sets used in this study.**
(PDF)

Table S2 **Plant degradome sequencing data sets used in this study.**
(PDF)

Table S3 **List of the mirtrons identified in** ***Arabidopsis*** **.** The sequences of the mature mirtrons, the IDs of the mirtron-generating introns (for example, AT3G60950.1-15 represents the 15^th^ intron resided within the transcript AT3G60950.1), and the corresponding annotations (TAIR 10) of the mirtron-containing genes are provided.
(XLS)

Table S4 **List of the mirtrons identified in rice.** The sequences of the mature mirtrons, the IDs of the mirtron-generating introns (for example, LOC_Os03g36170.1-6 represents the 6^th^ intron resided within the transcript LOC_Os03g36170.1), and the corresponding annotations (TIGR rice 6.1) of the mirtron-containing genes are provided.
(XLS)

Table S5 **List of the target genes of the mature mitrons in** ***Arabidopsis*** **.** The mirtron IDs, the transcript IDs of the target genes, and the target gene annotations (TAIR 10) are provided.
(XLS)

Table S6 **List of the target genes of the mature mitrons in rice.** The mirtron IDs, the transcript IDs of the target genes, and the target gene annotations (TIGR rice 6.1) are provided.
(XLS)
