# Supplementary figures and images for: Large-Scale Identification of Mirtrons in Arabidopsis and Rice
Source: PLoS One. 2012 Feb 13;7(2):e31163. doi: 10.1371/journal.pone.0031163 (PMC3278437; doi:10.1371/journal.pone.0031163)

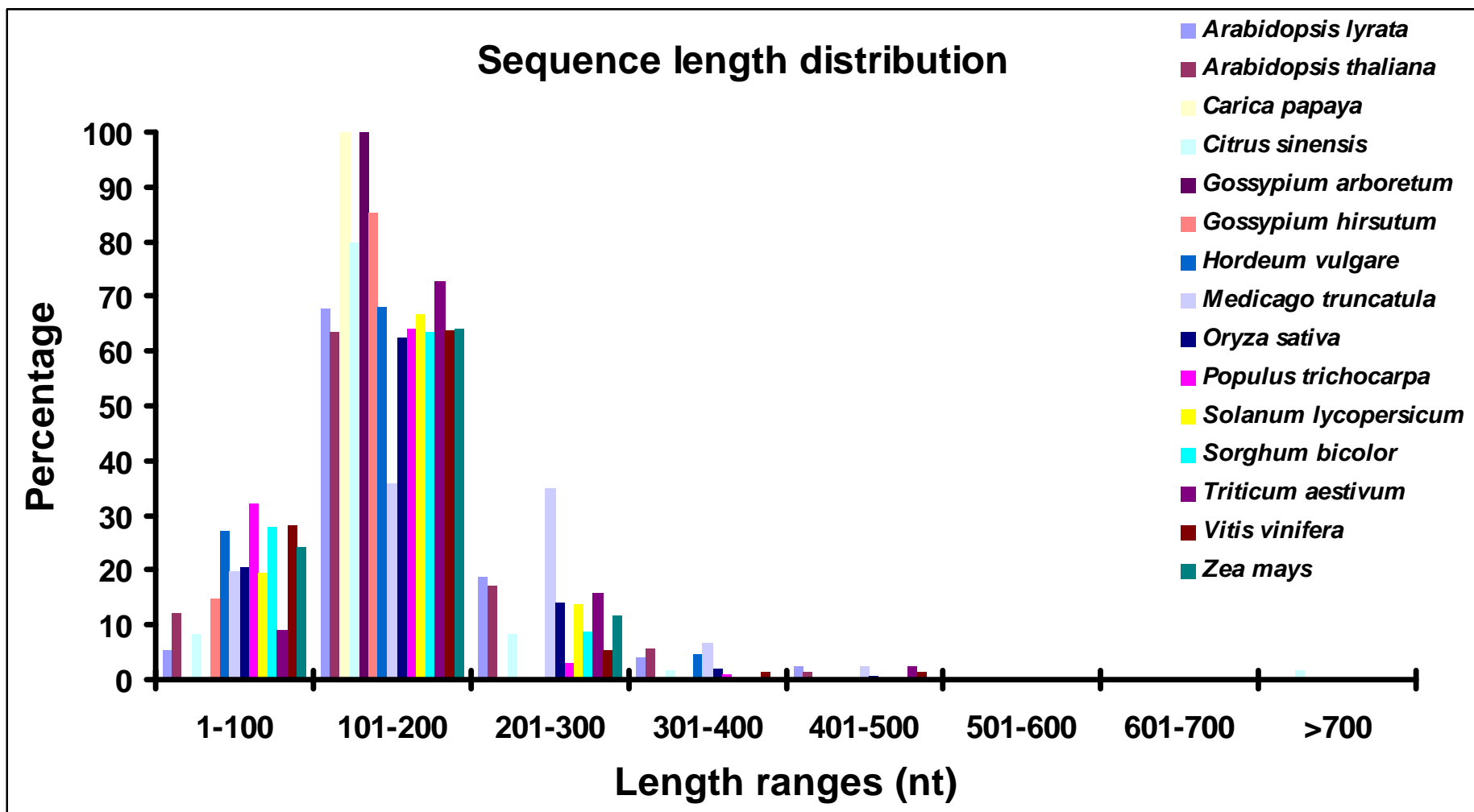

Supplement: Figure S1 — Sequence length distribution of the miRBase-registered microRNA precursors belonging to 15 plant species. The x axis marks the sequence length range, and the y axis measures the percentage of the microRNA precursors resided within a specific length range in a plant. (PDF) [file pone.0031163.s001.pdf]

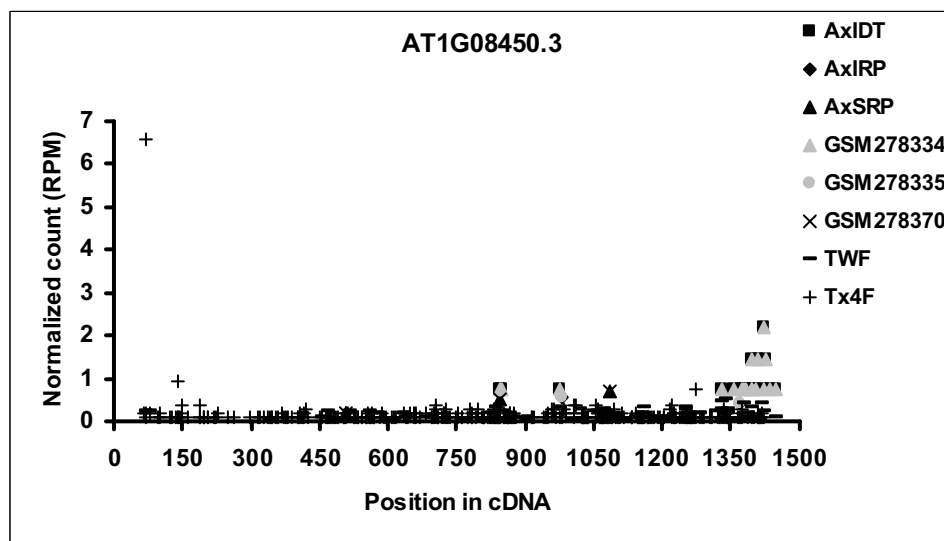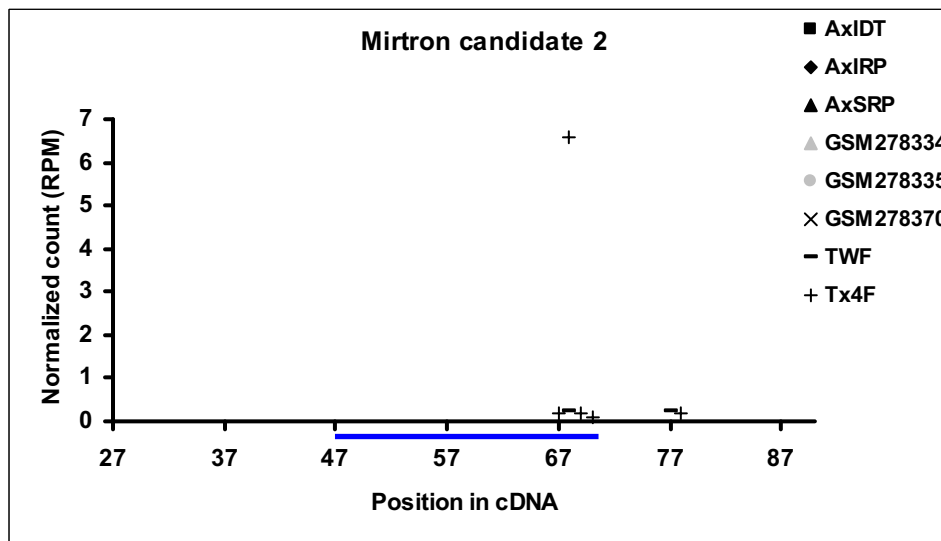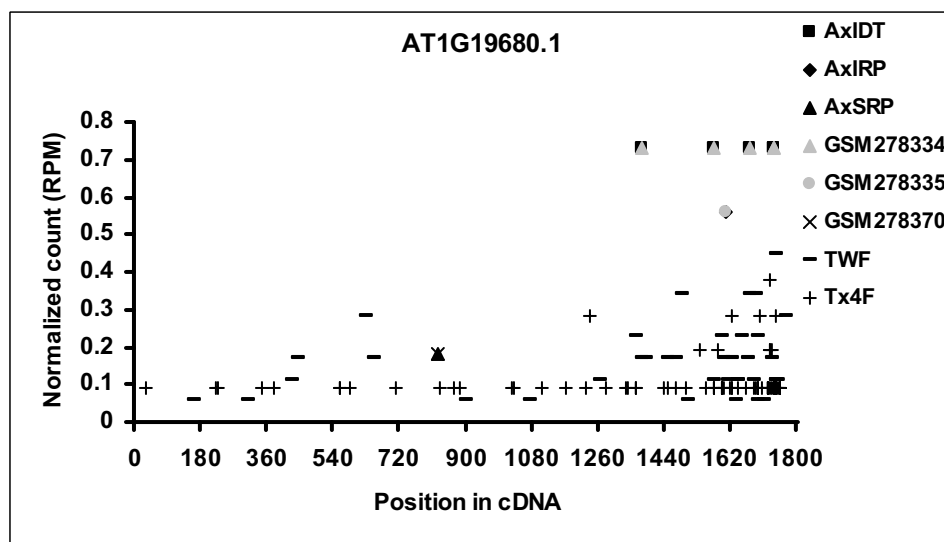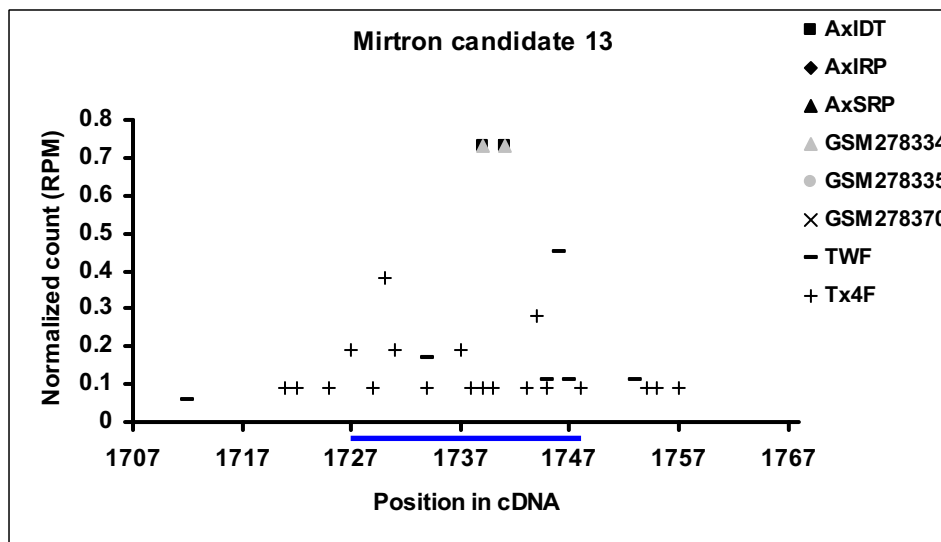

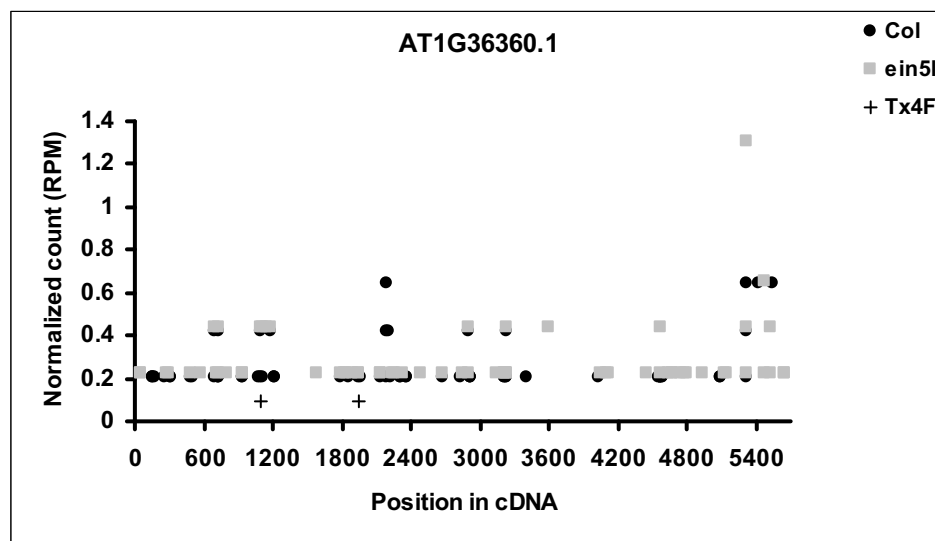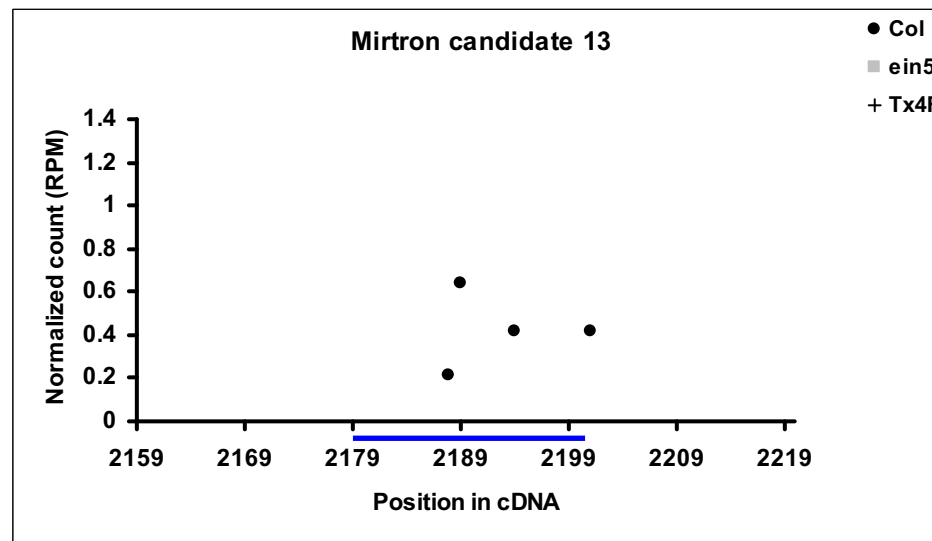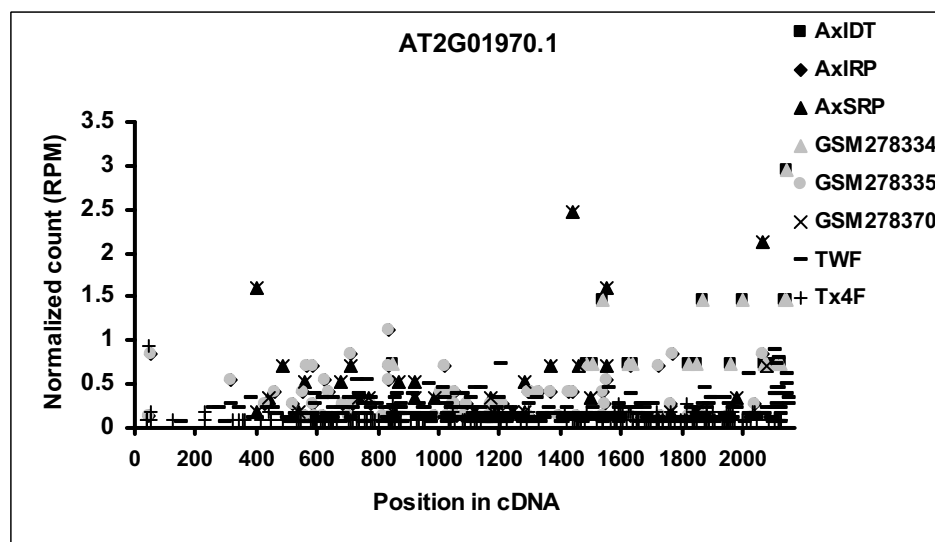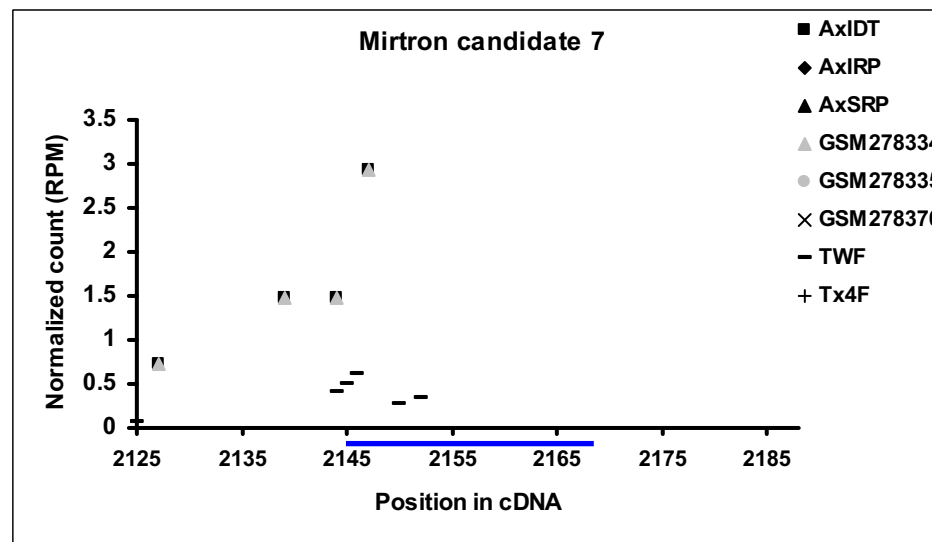

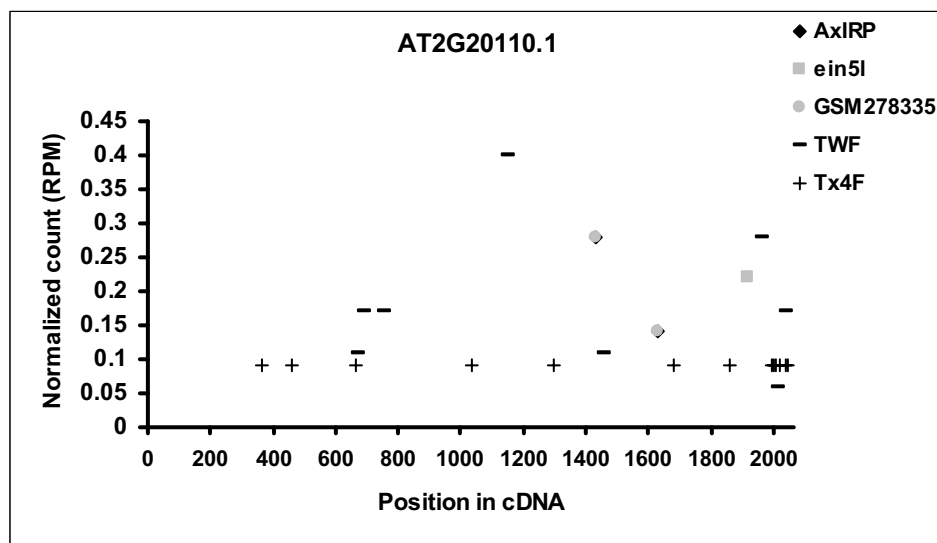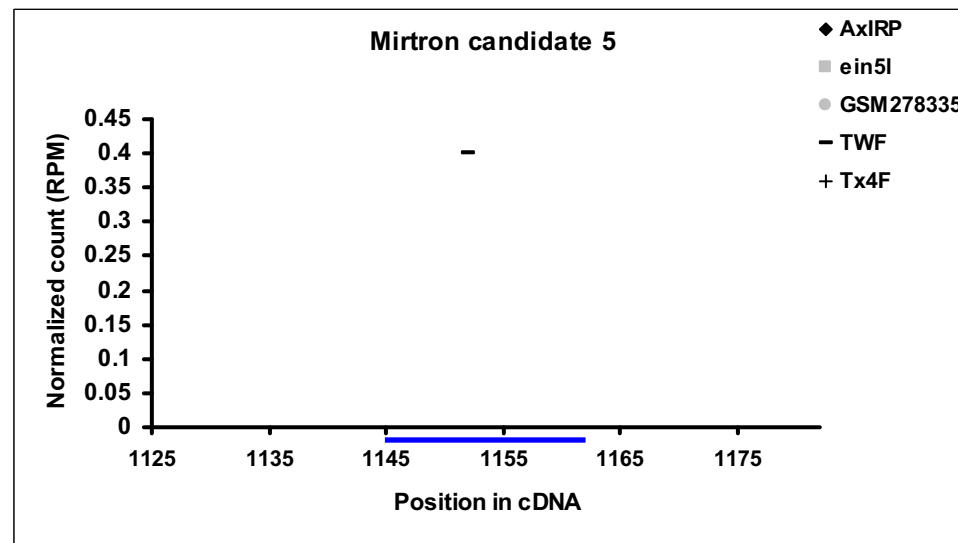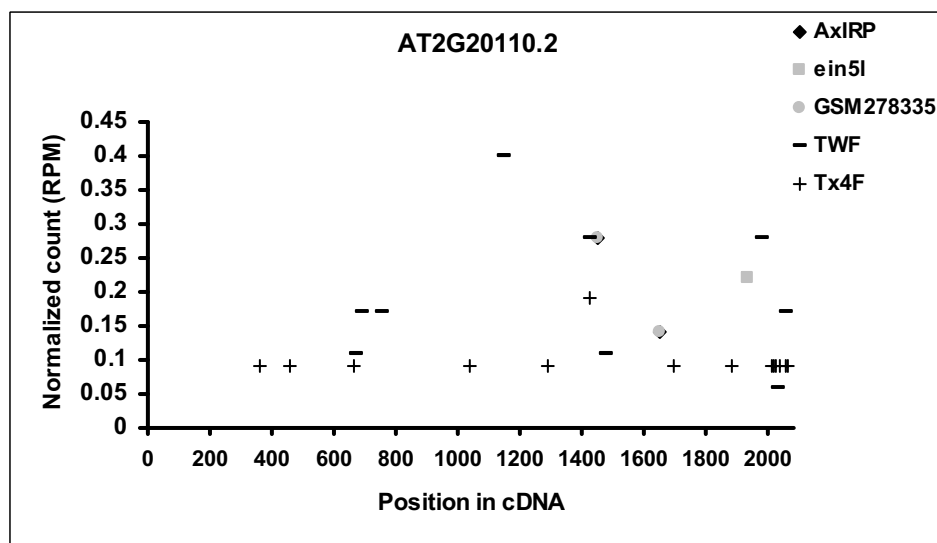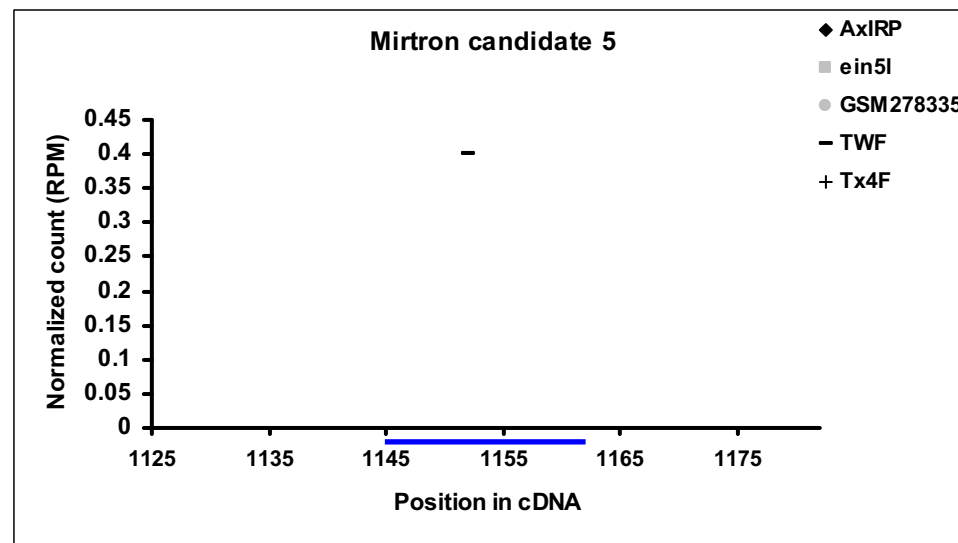

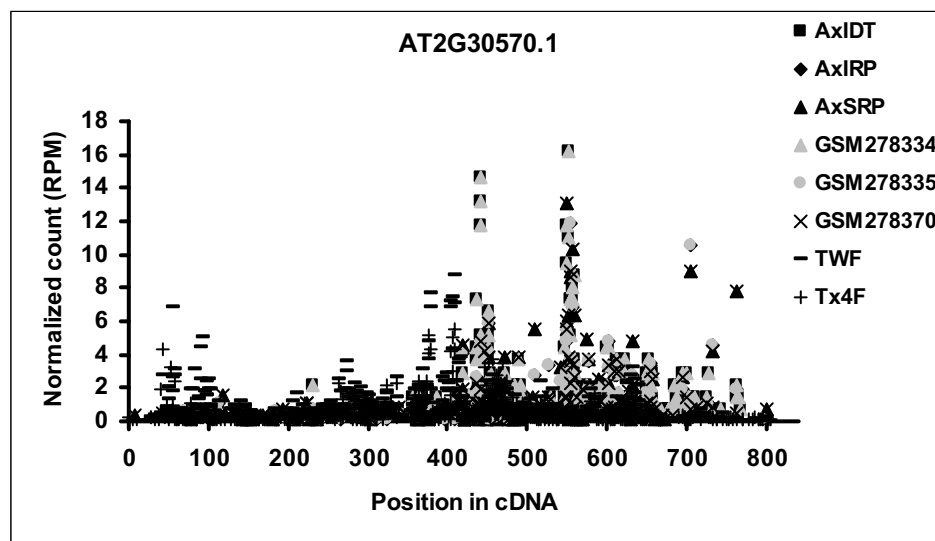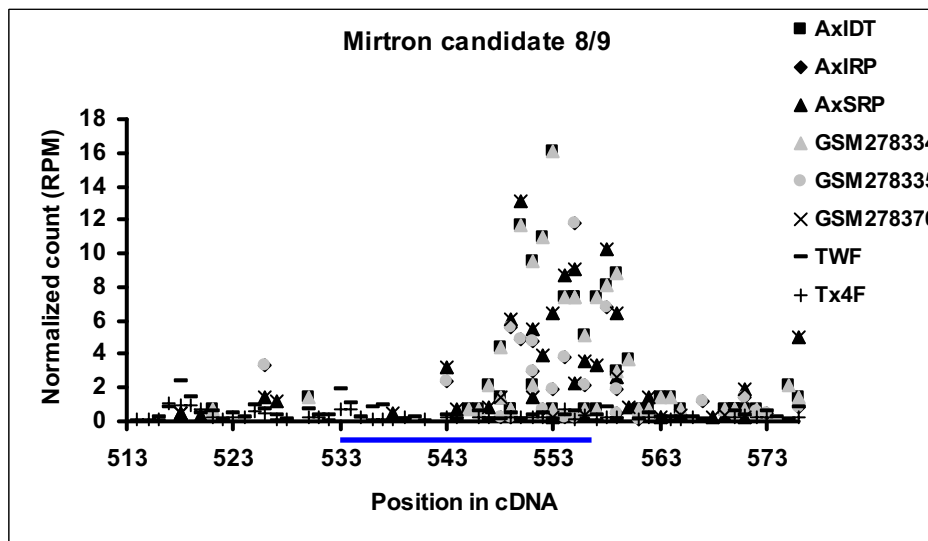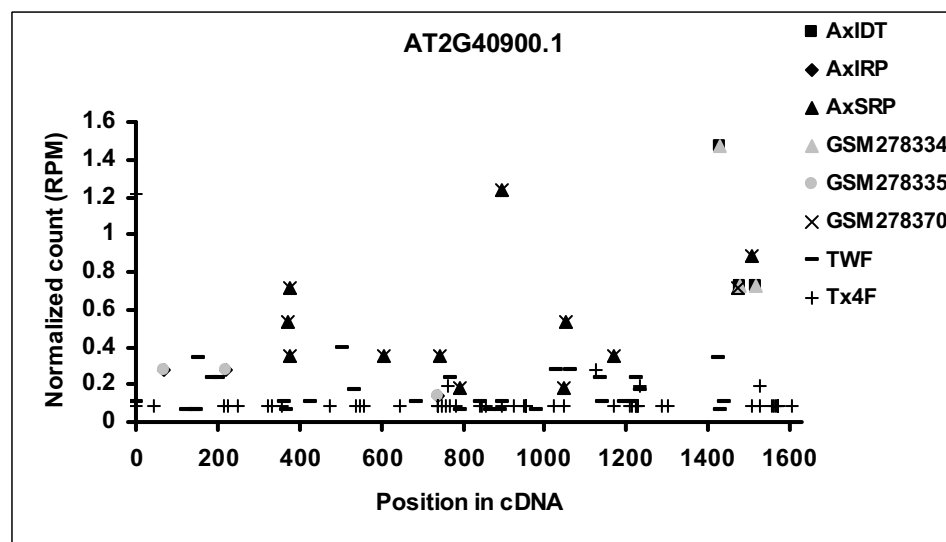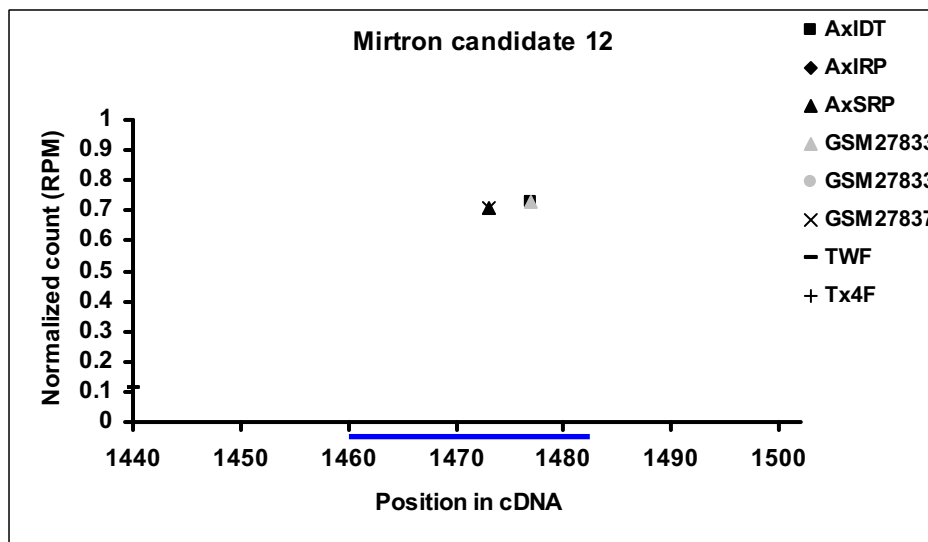

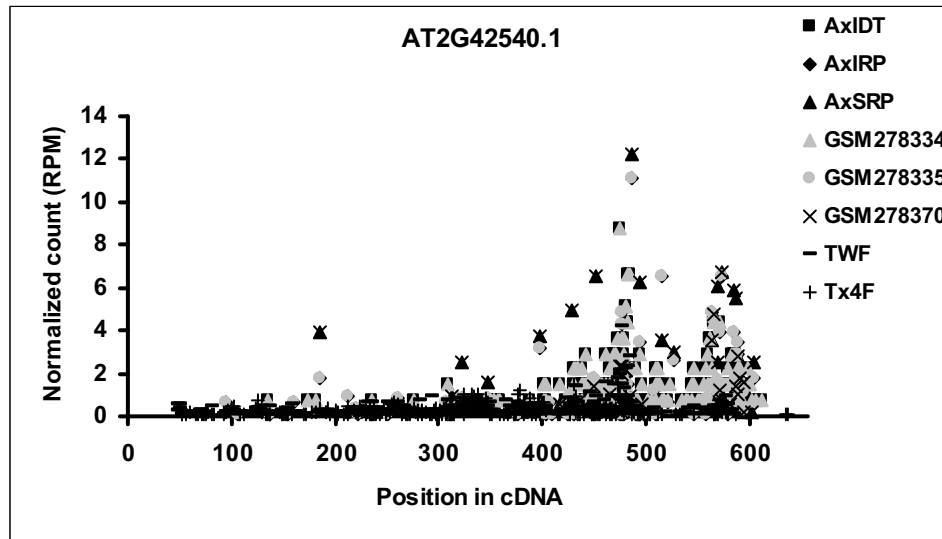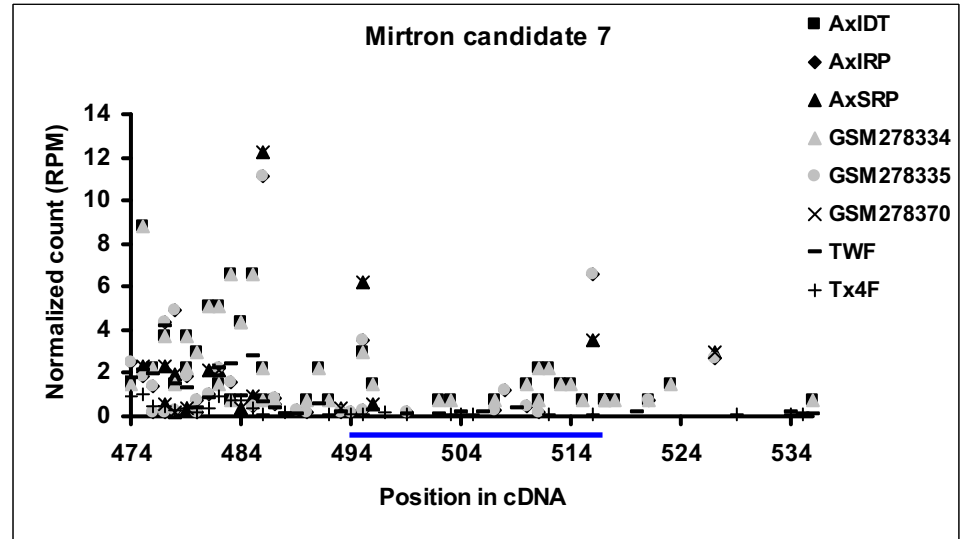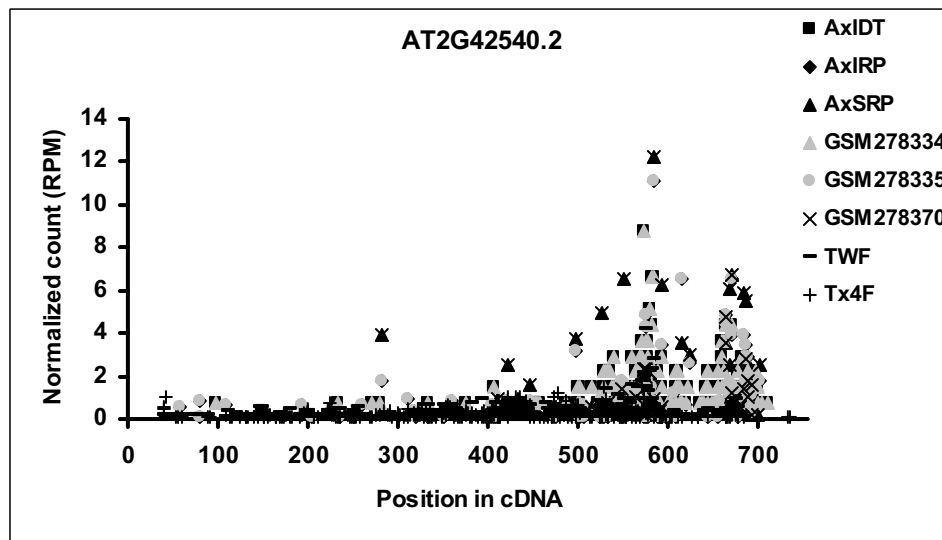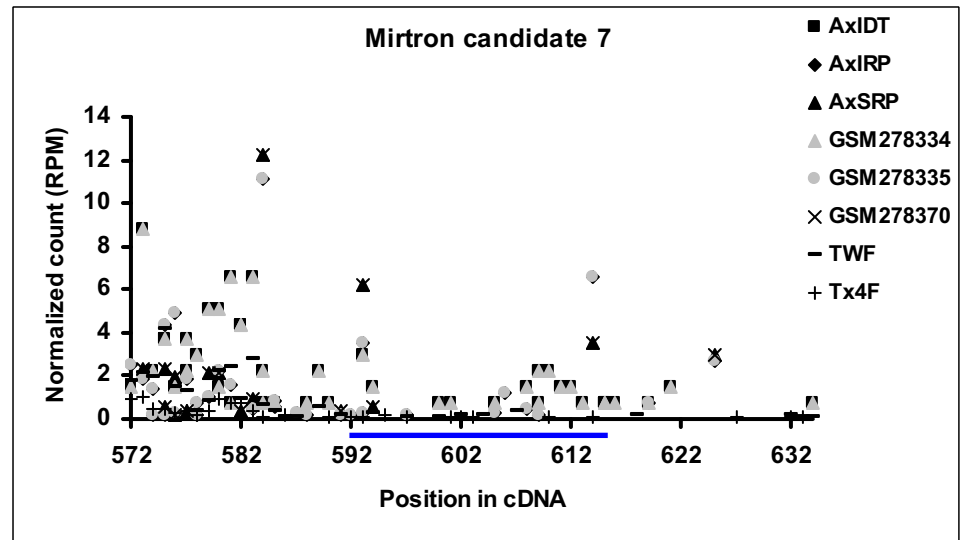

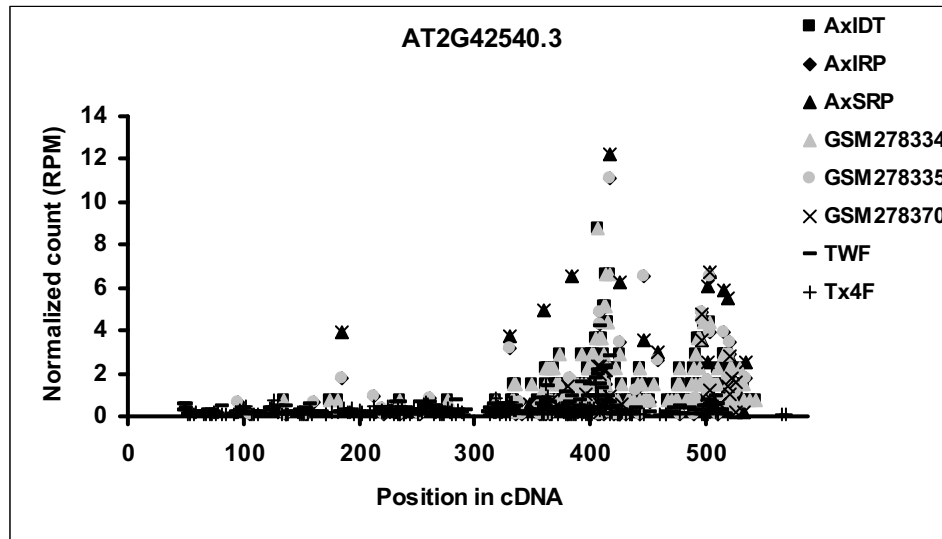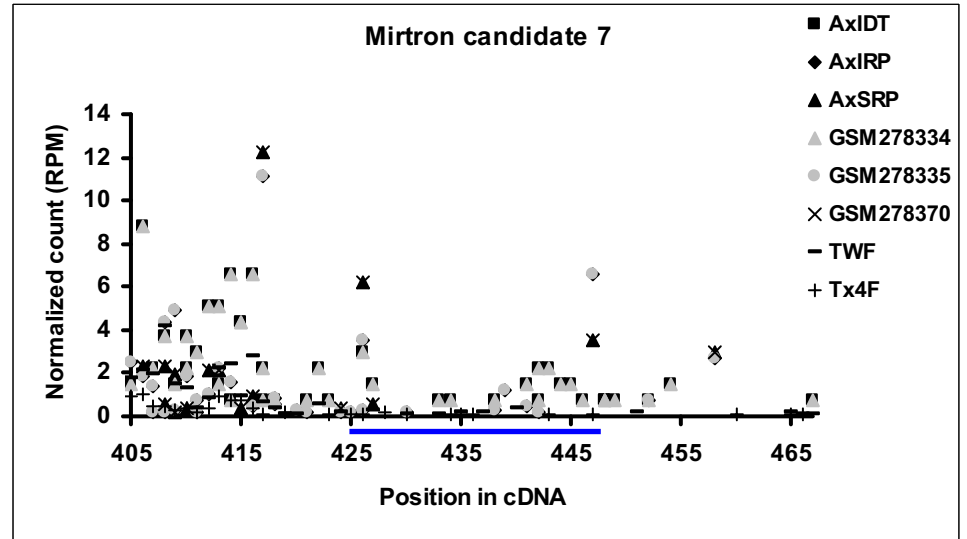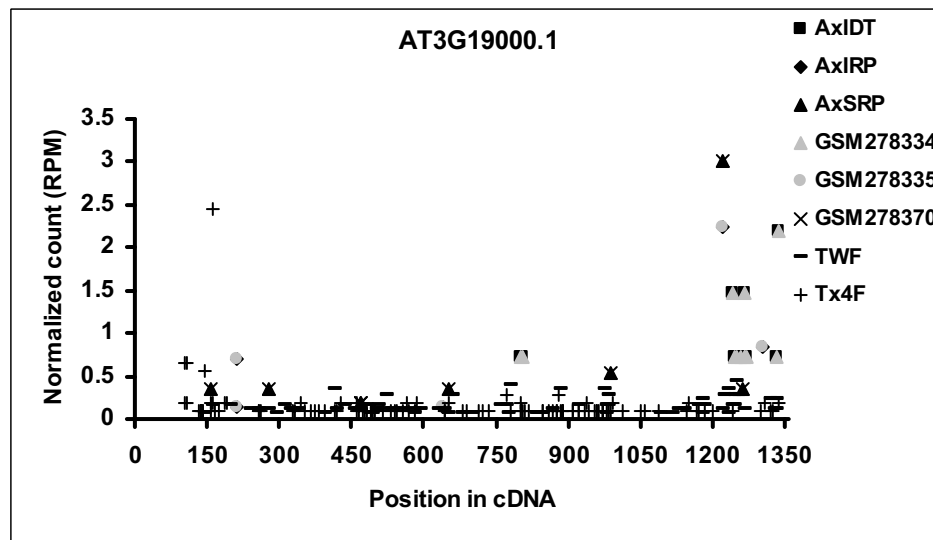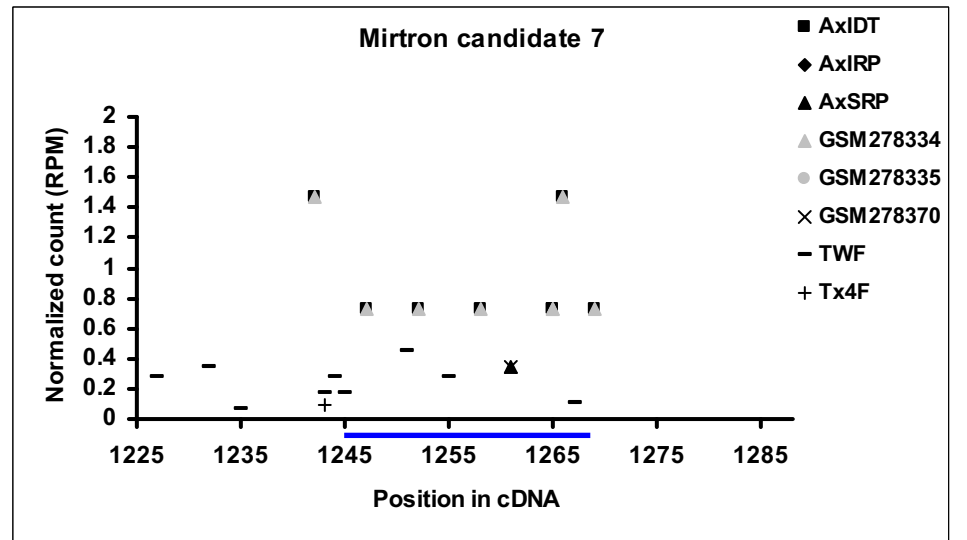

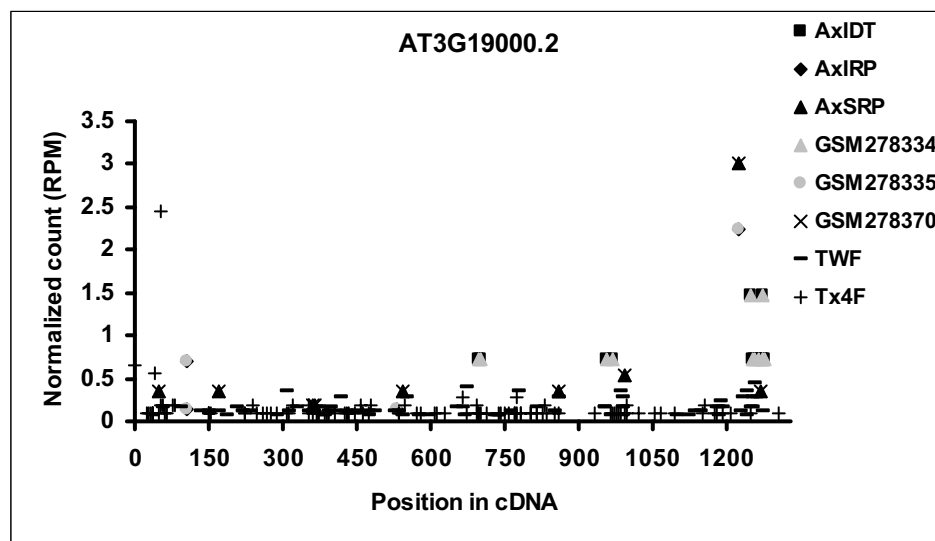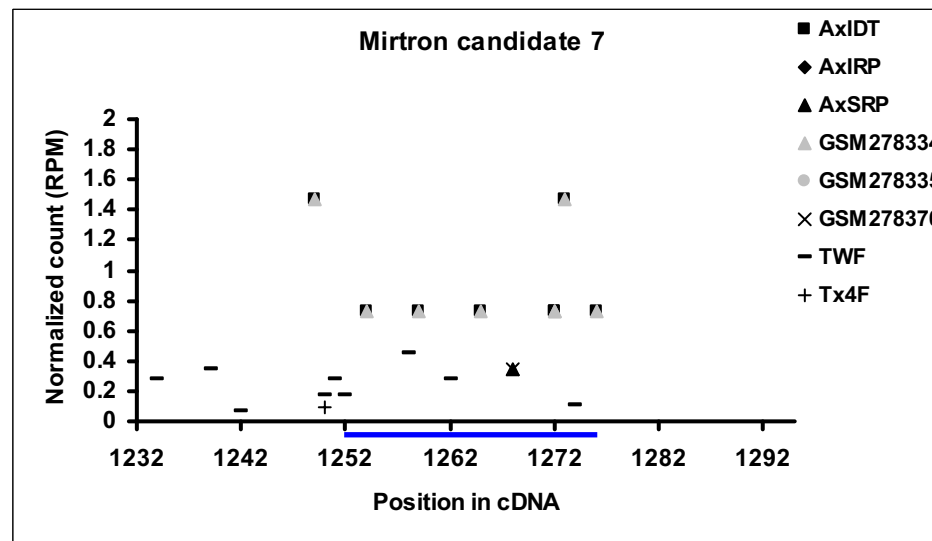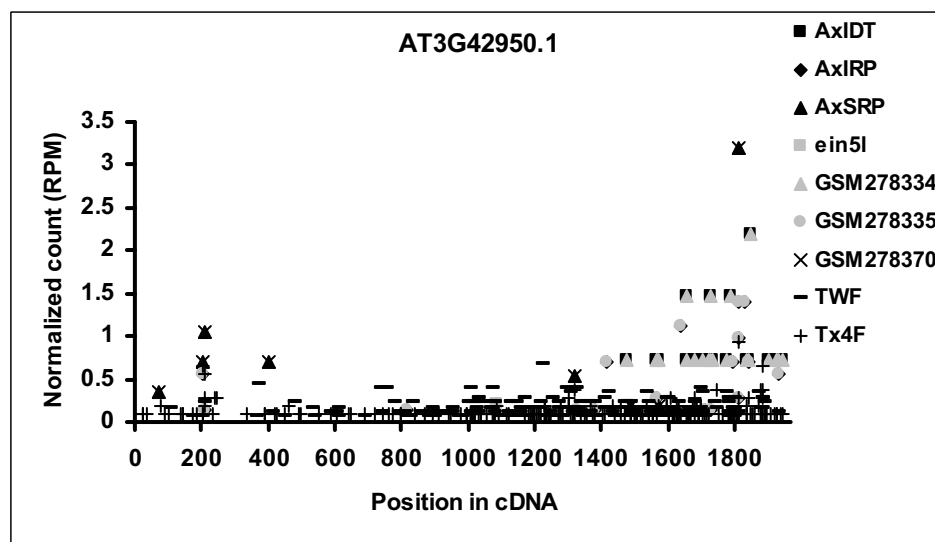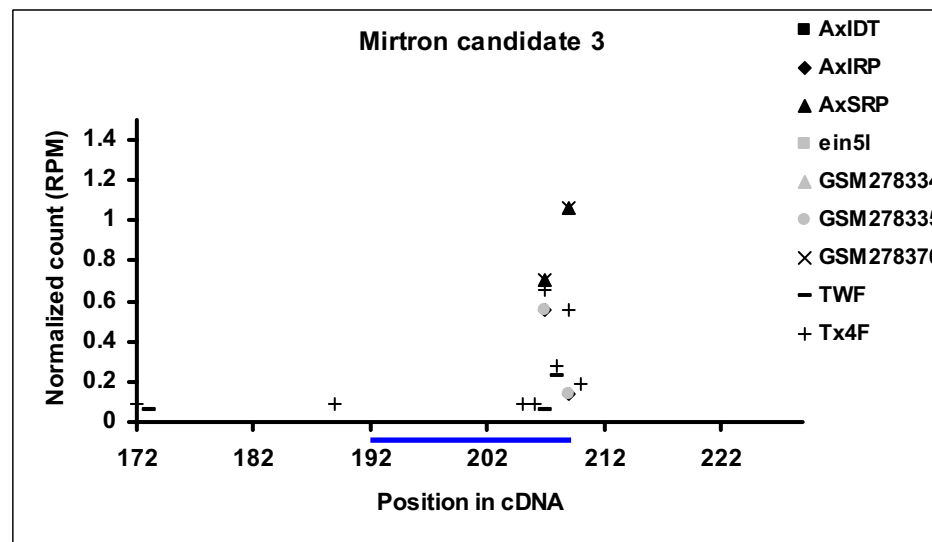

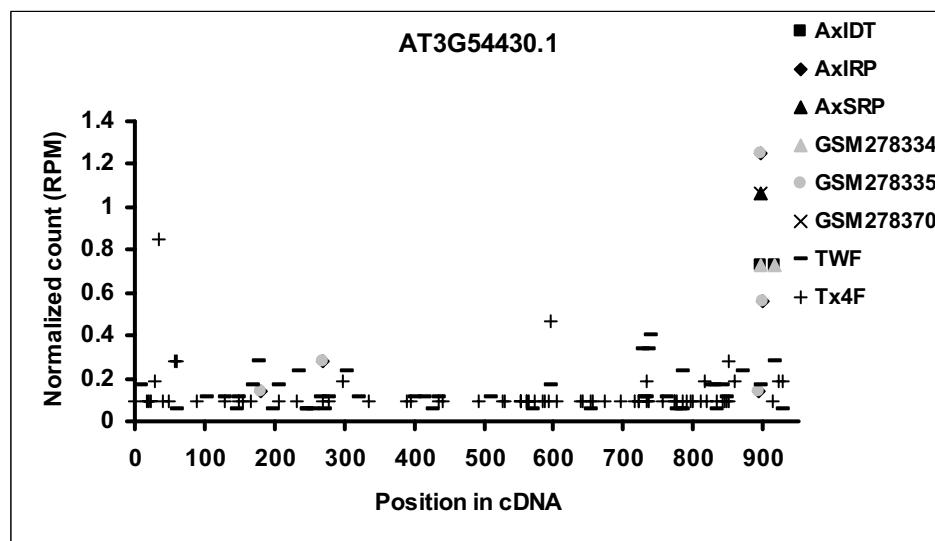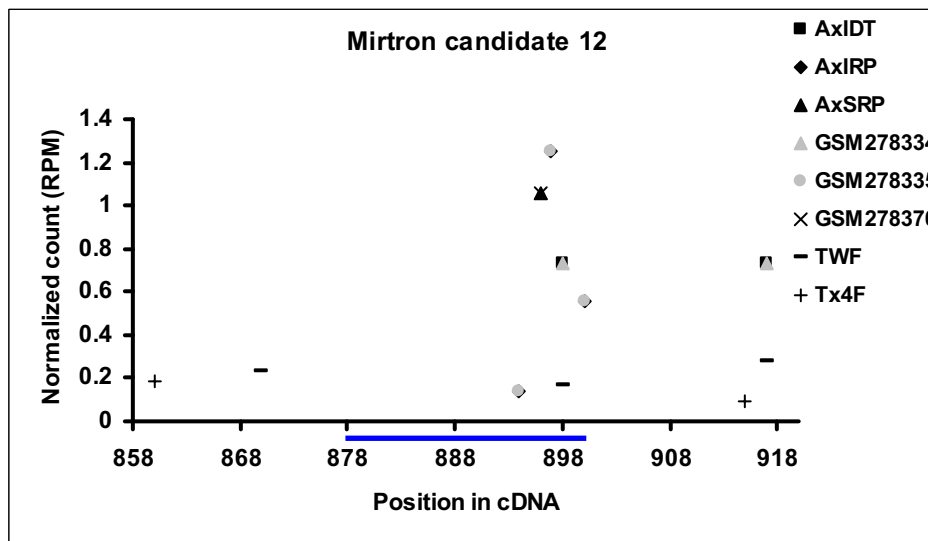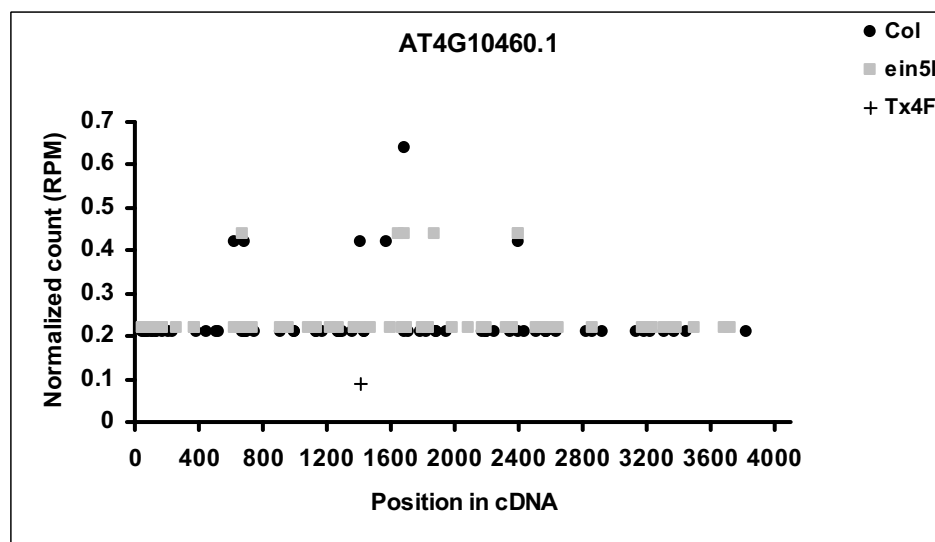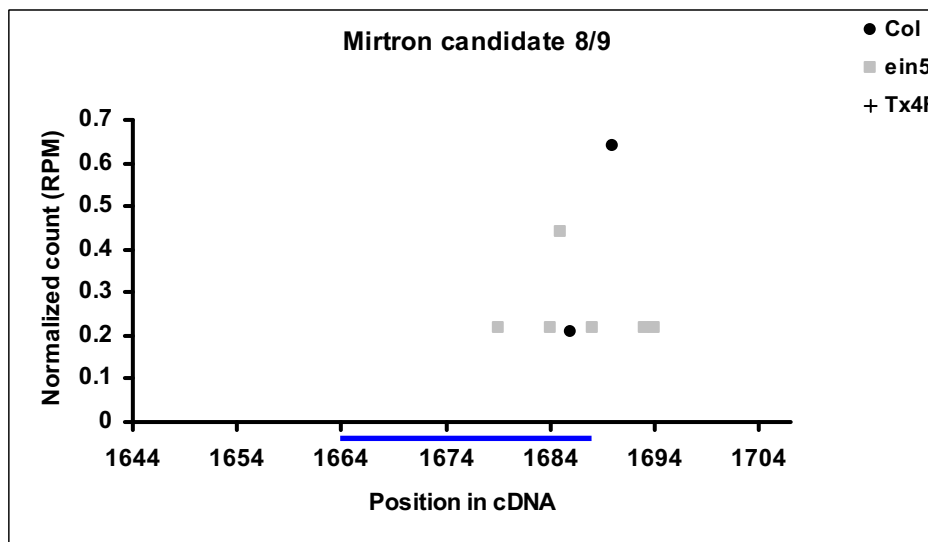

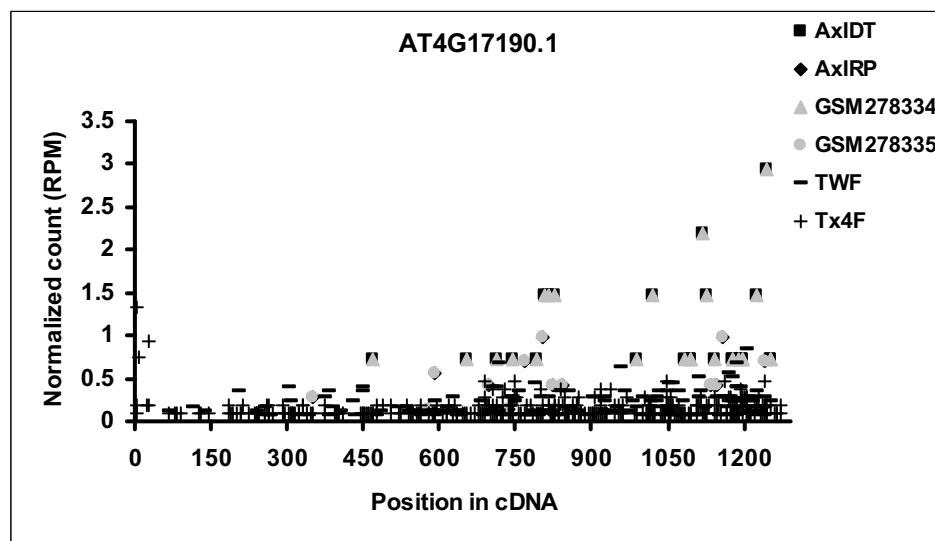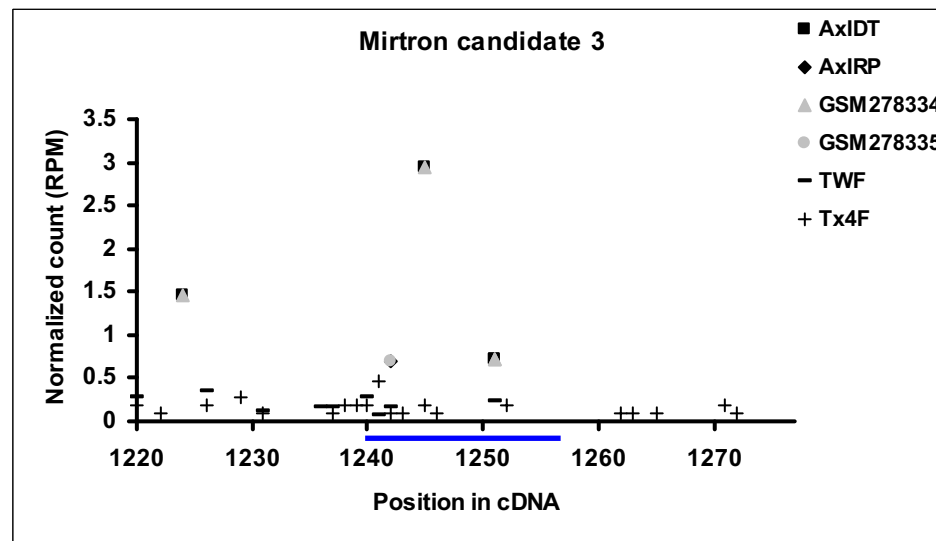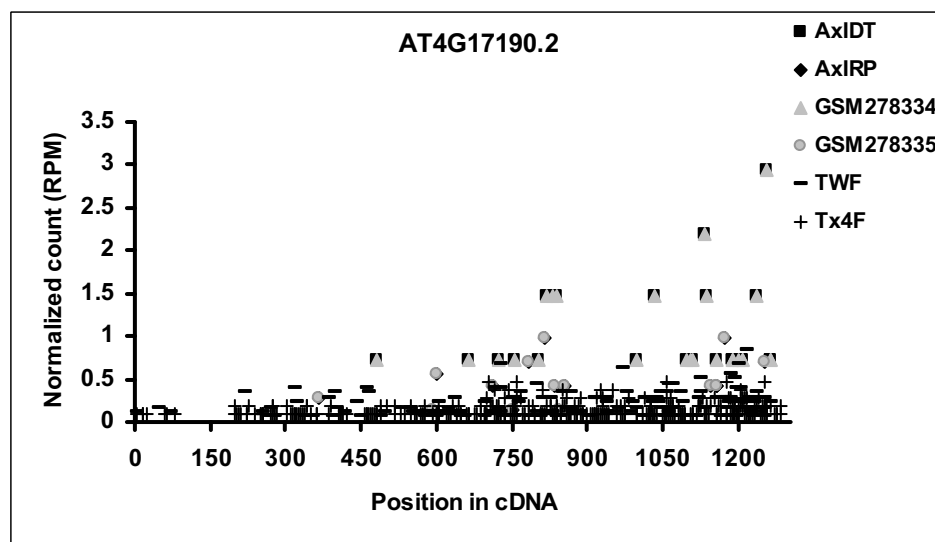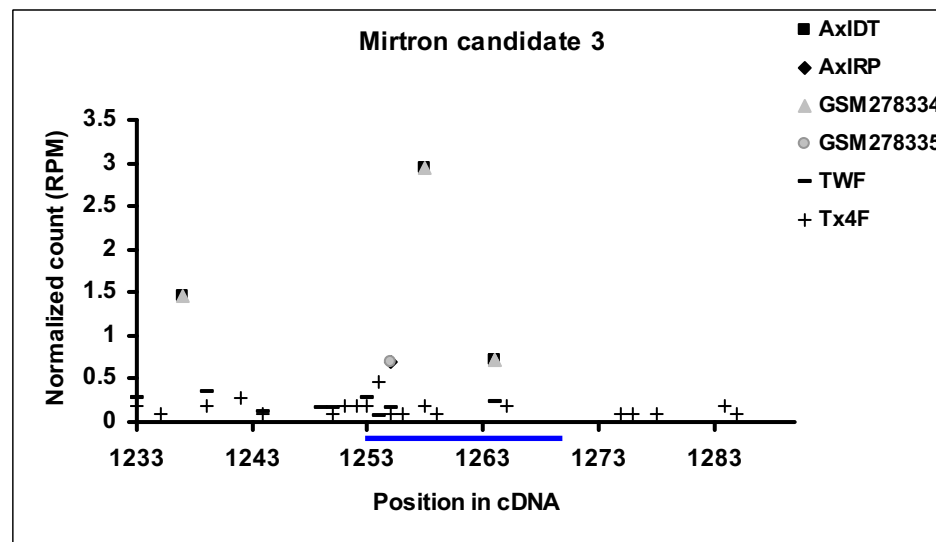

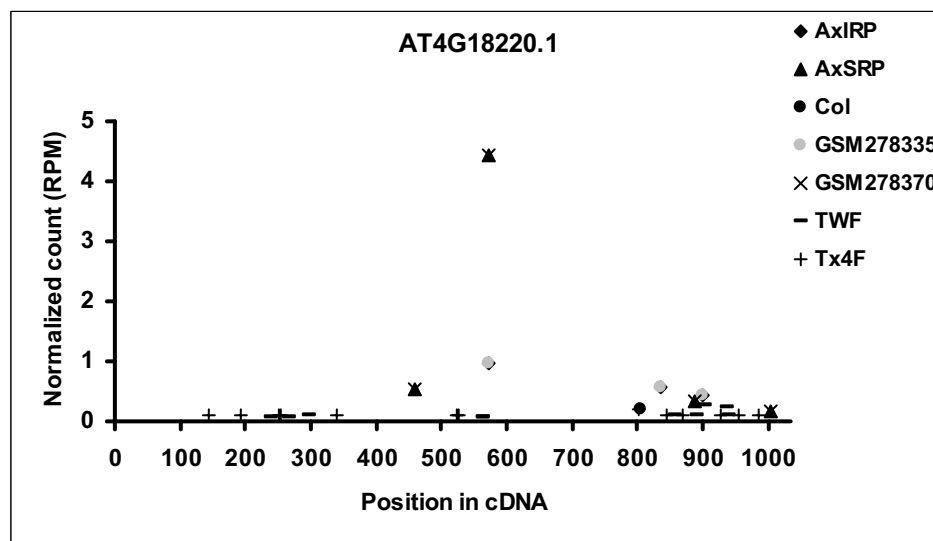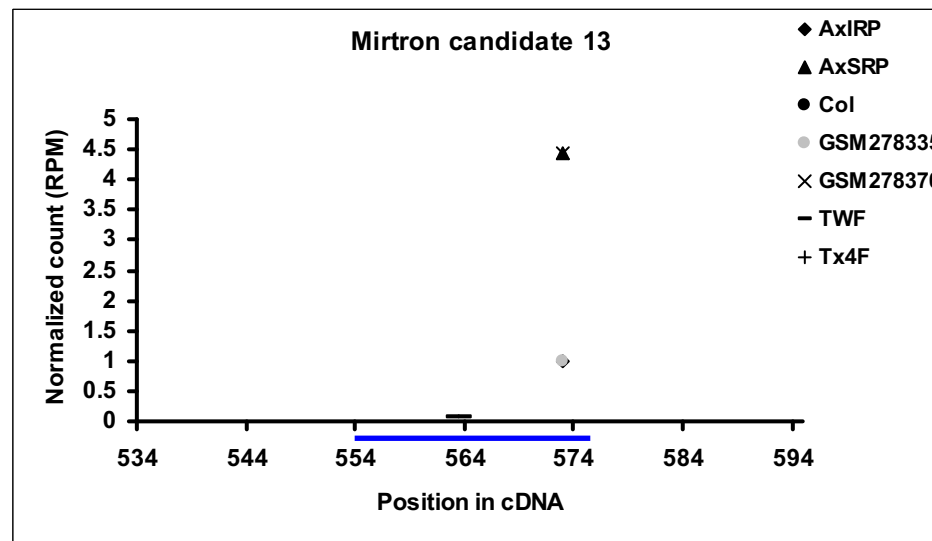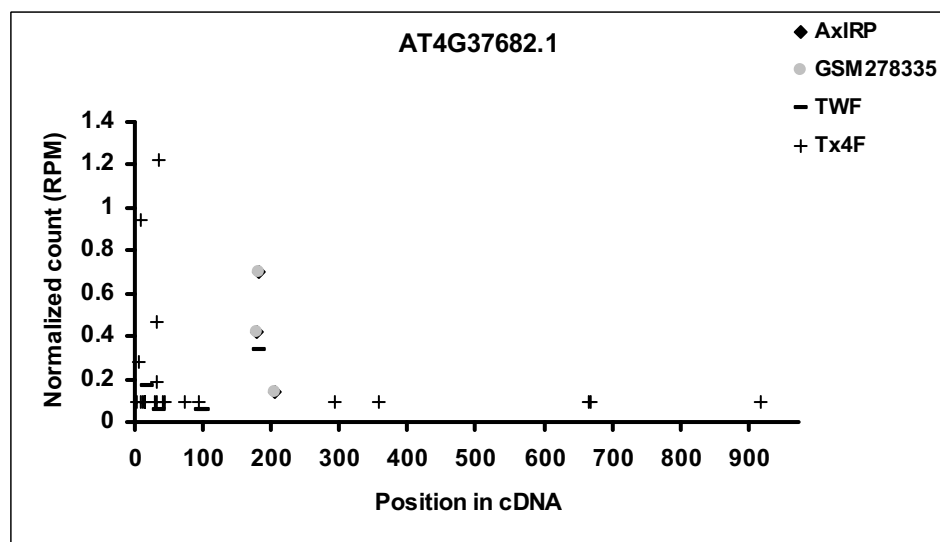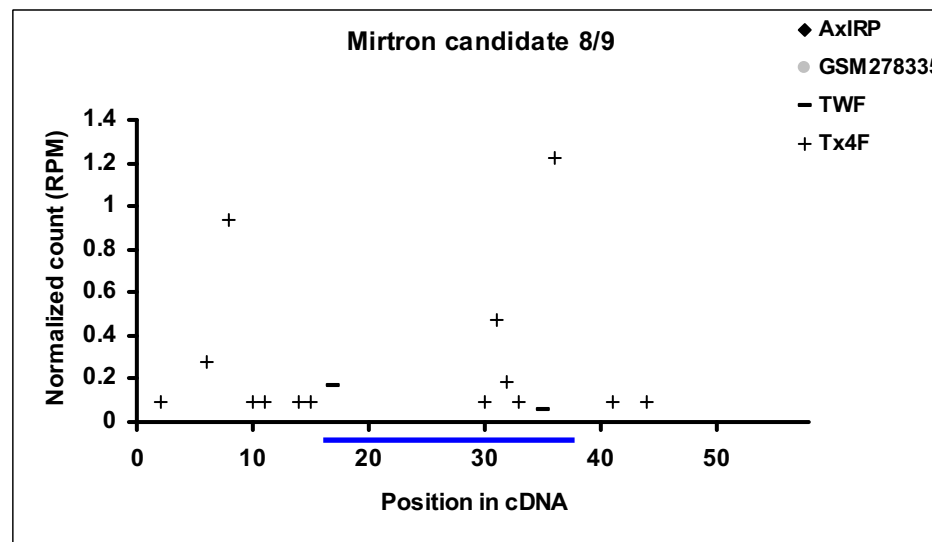

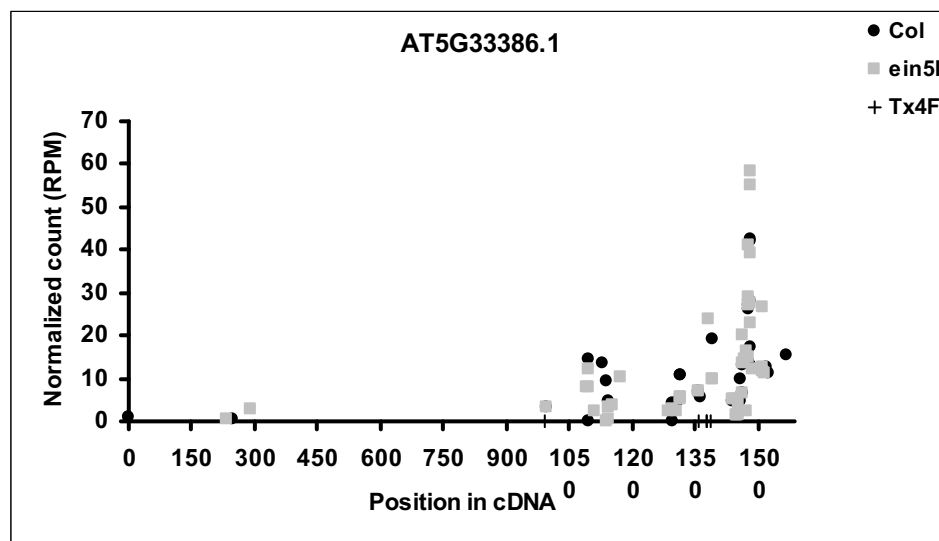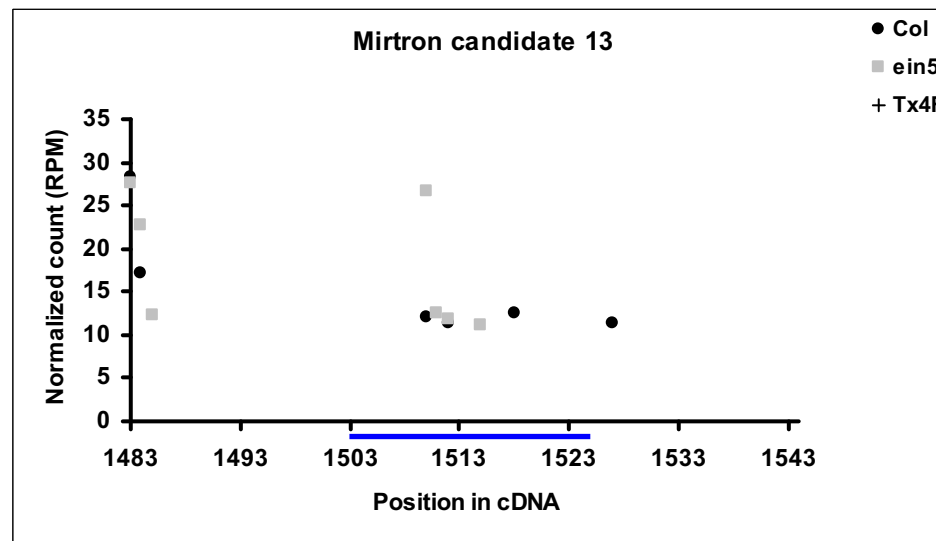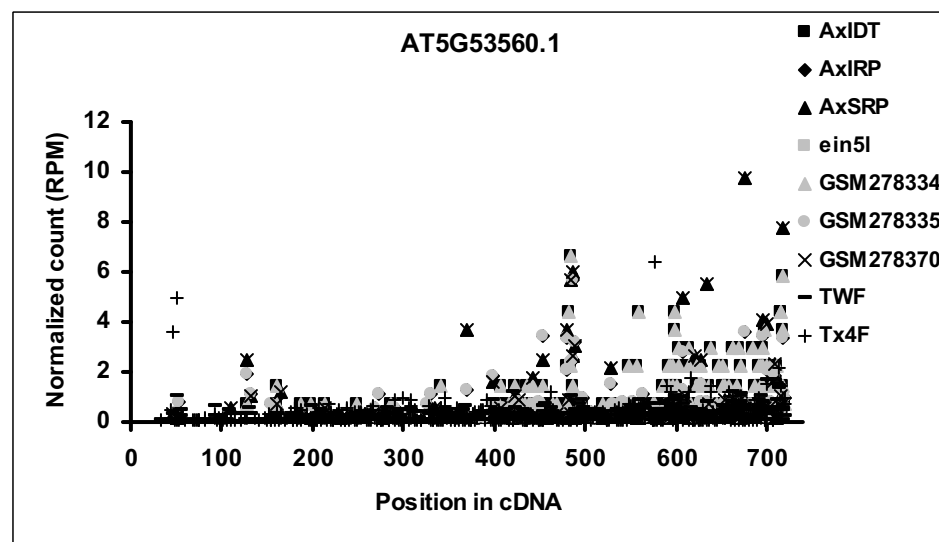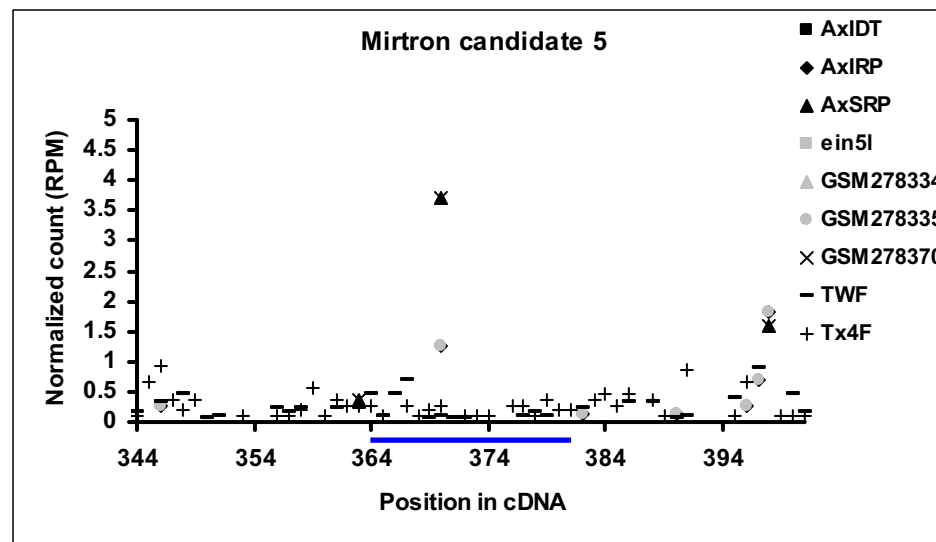

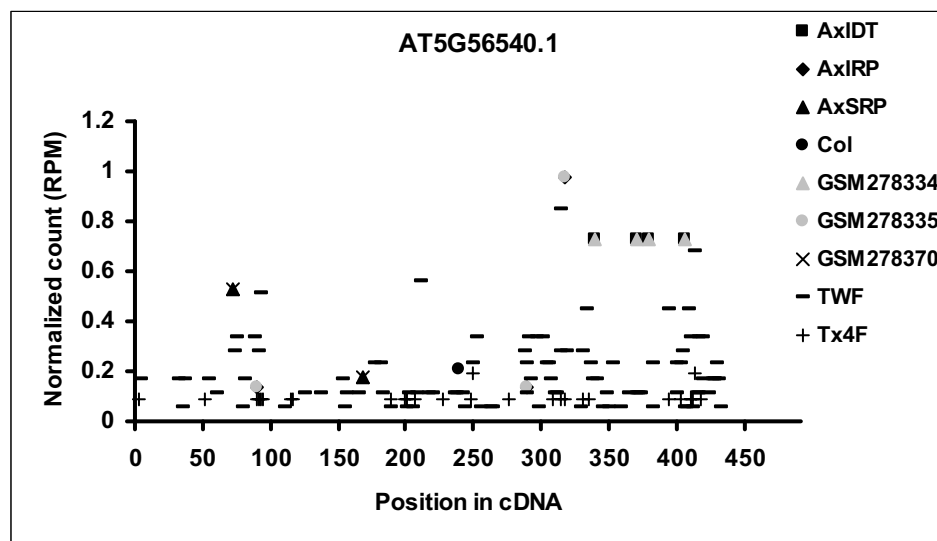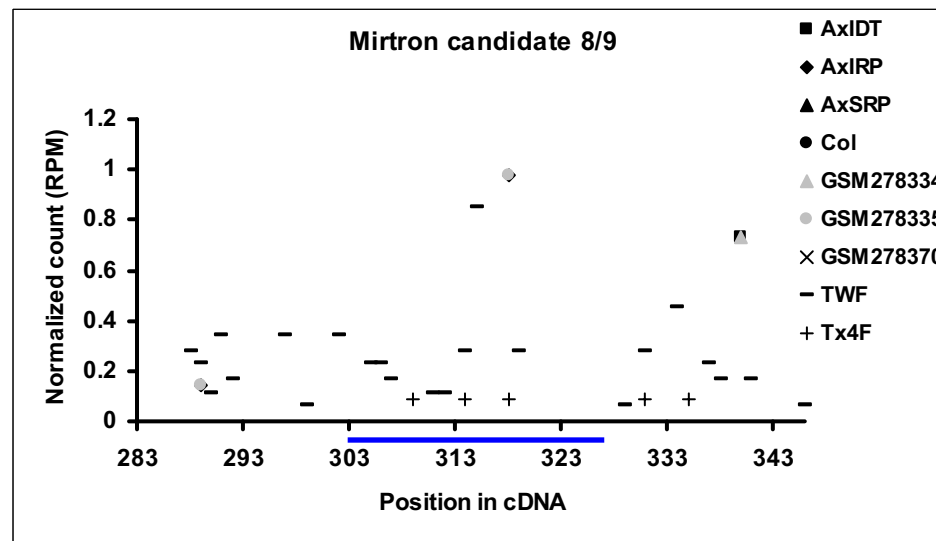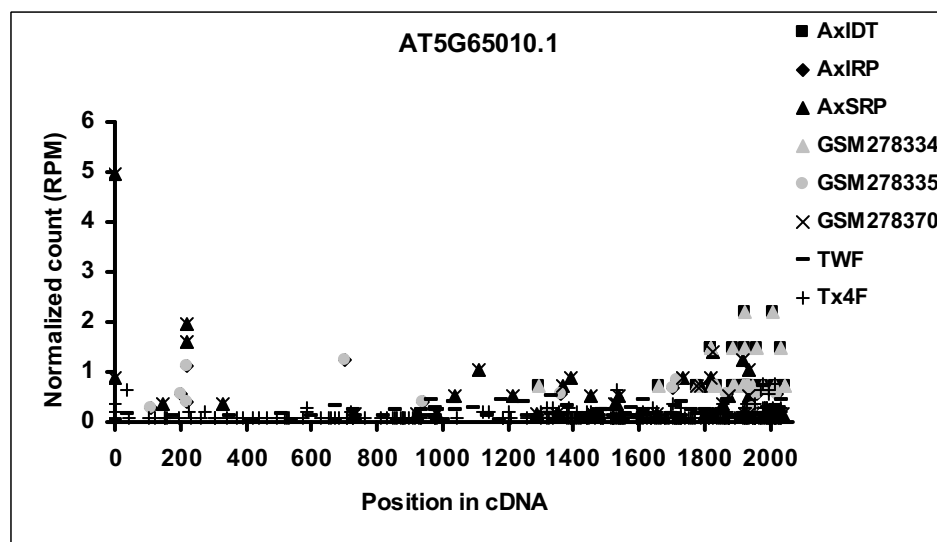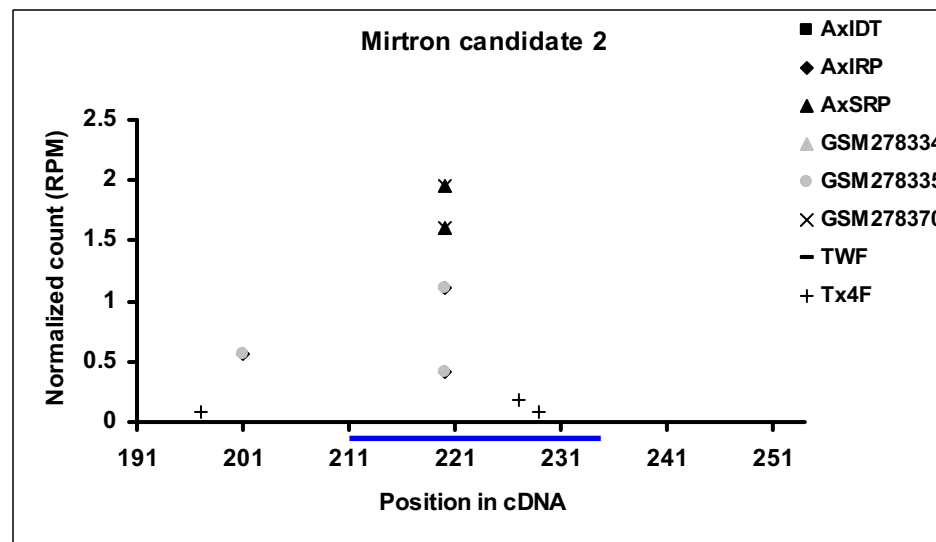

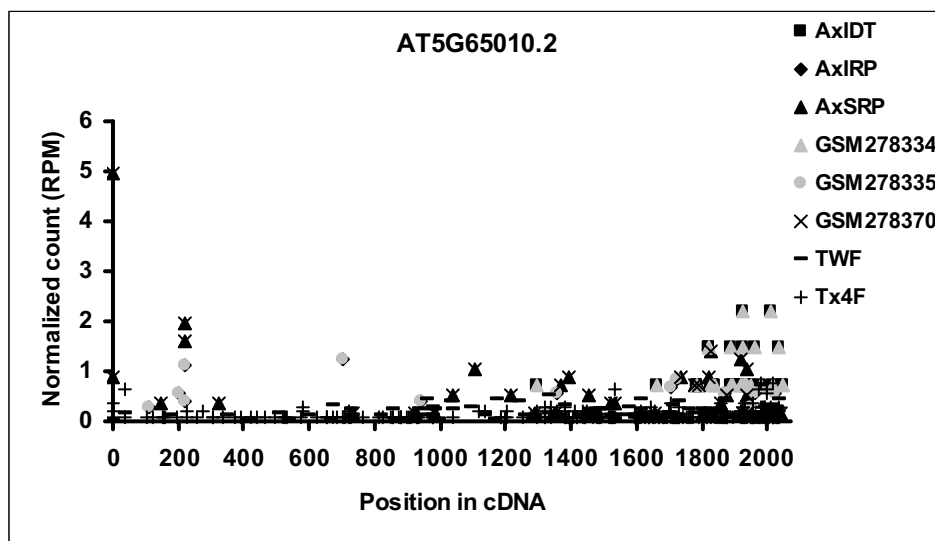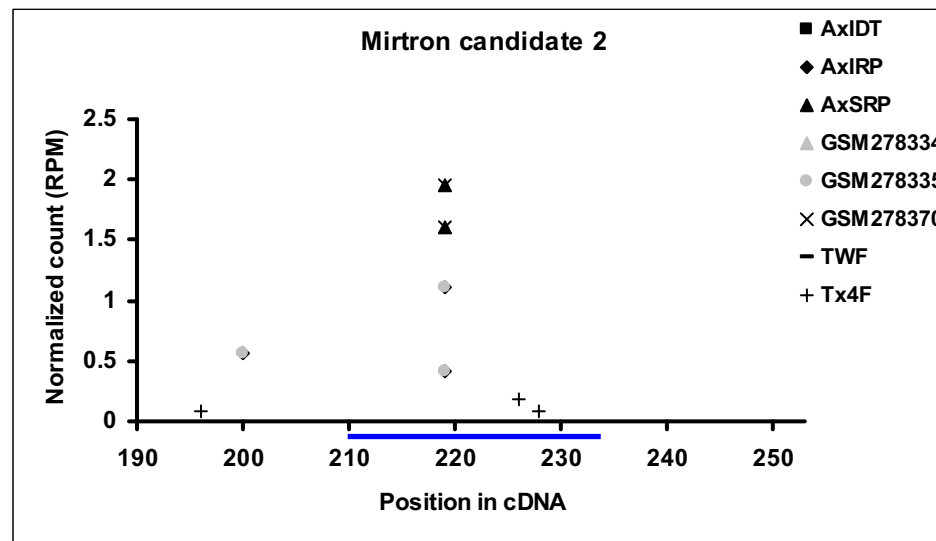

Supplement: Figure S3 — Degradome sequencing data-based identification of the targets of the mature mirtrons in Arabidopsis . For all the sub-figures, the first panels depict the degradome signals all along the target transcripts, and the other panels provide detailed views of the cleavage signals within the regions surrounding the target recognition sites (denoted by blue horizontal lines). The transcript IDs are shown in the first panels, and the mirtron IDs are listed in the other panels (see Table S3 and S4 for the sequence information corresponding to the mirtron IDs). The x axes measure the positions of the signals along the transcripts, and the y axes measure the signal intensities based on normalized counts (in RPM, reads per million), allowing cross-library comparison. See Table S2 for the degradome data sets used in this analysis. (PDF) [file pone.0031163.s003.pdf]

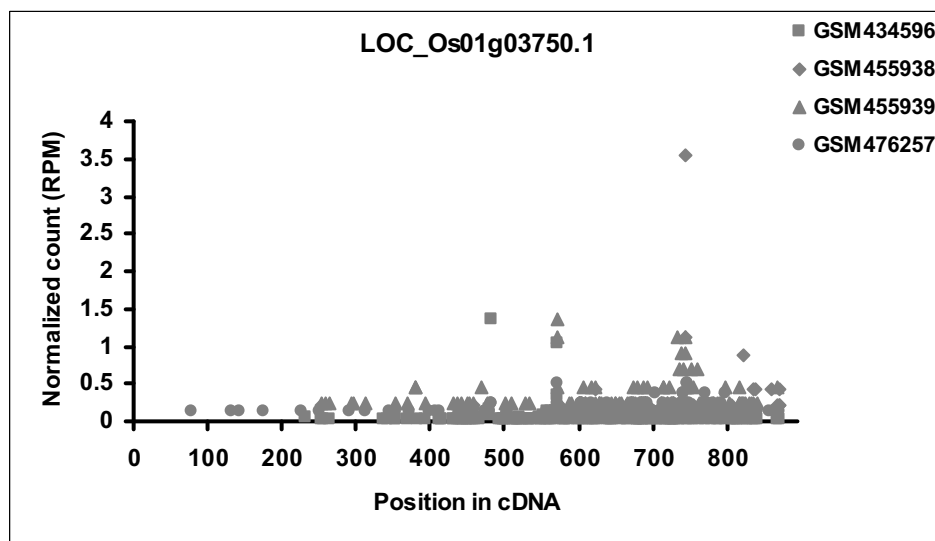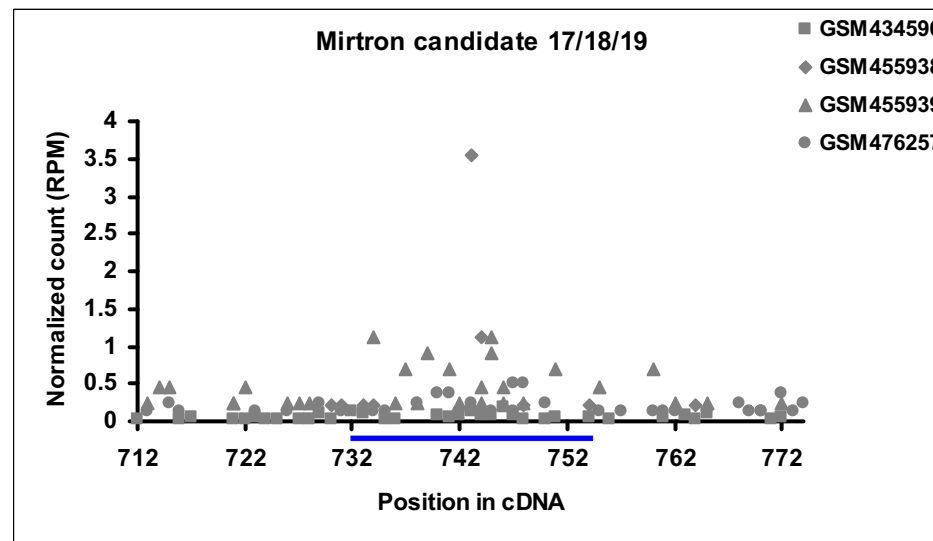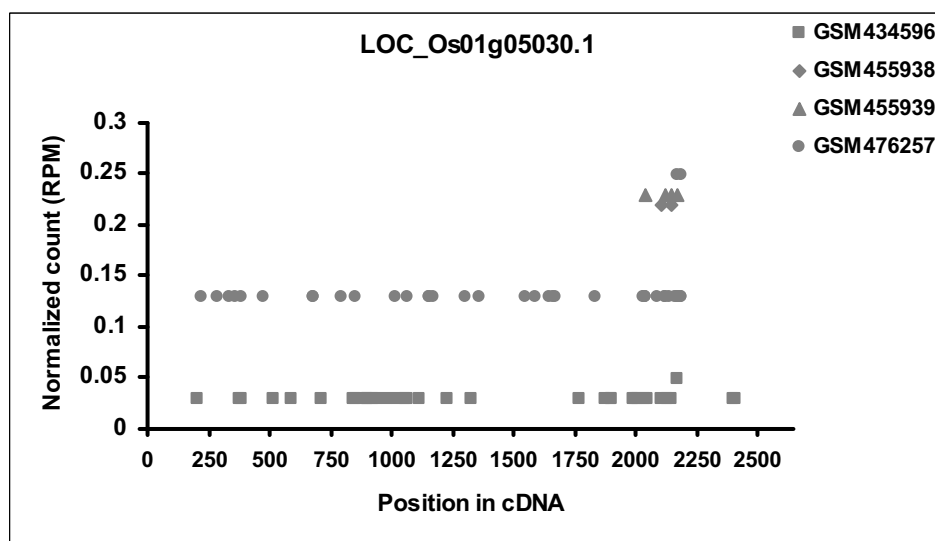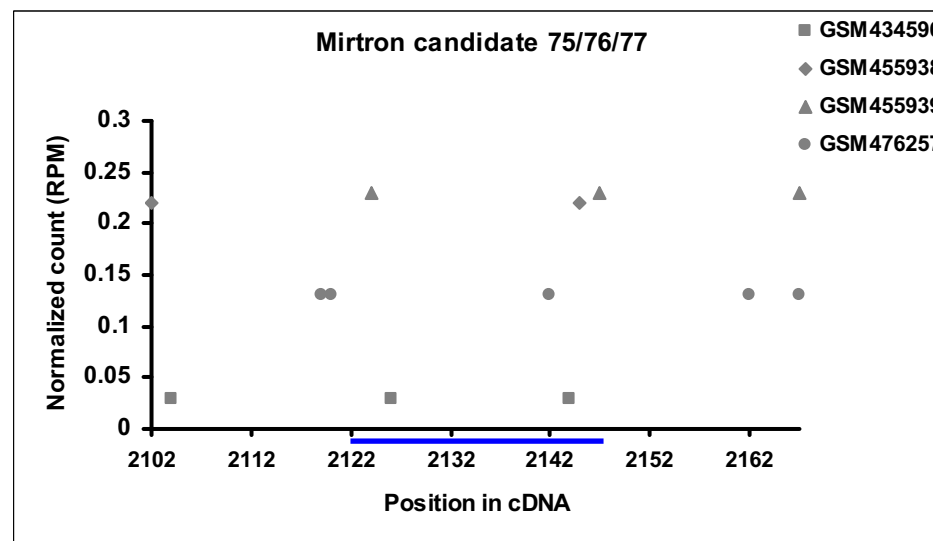

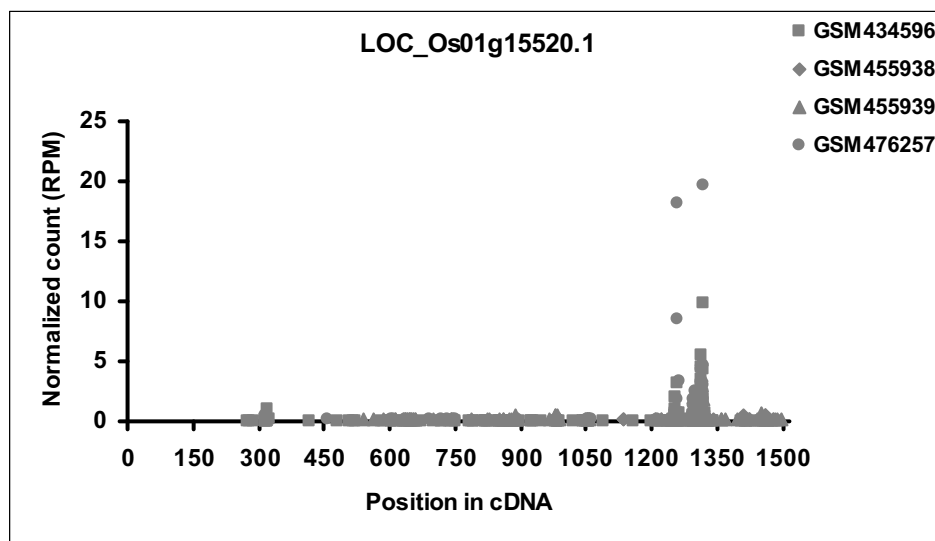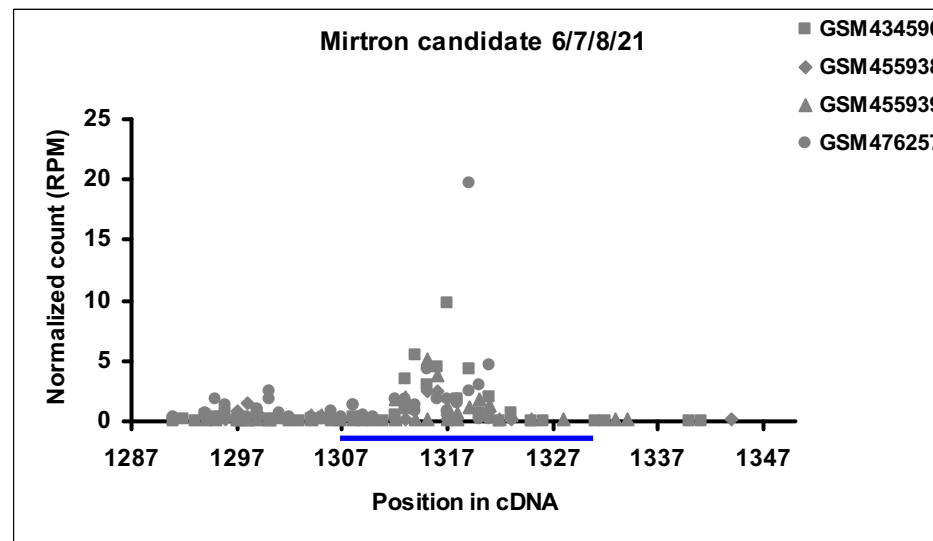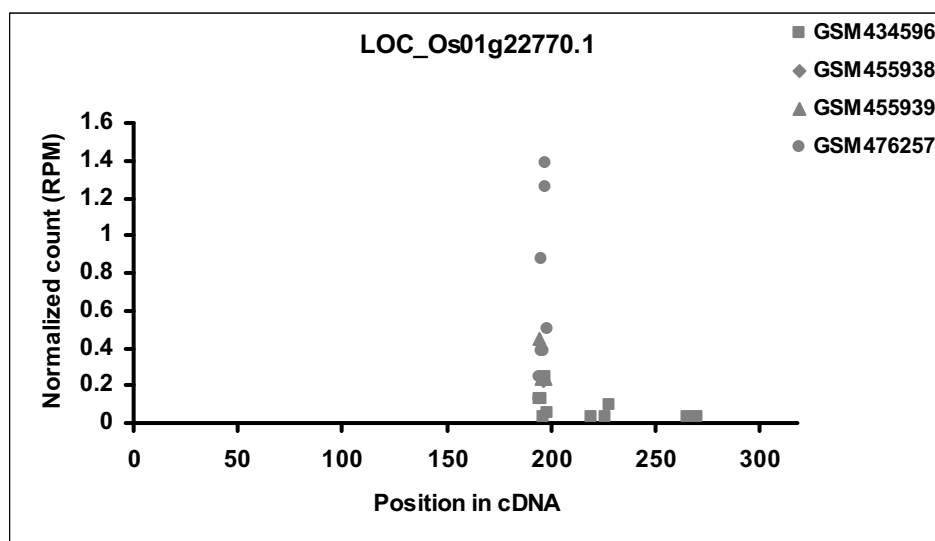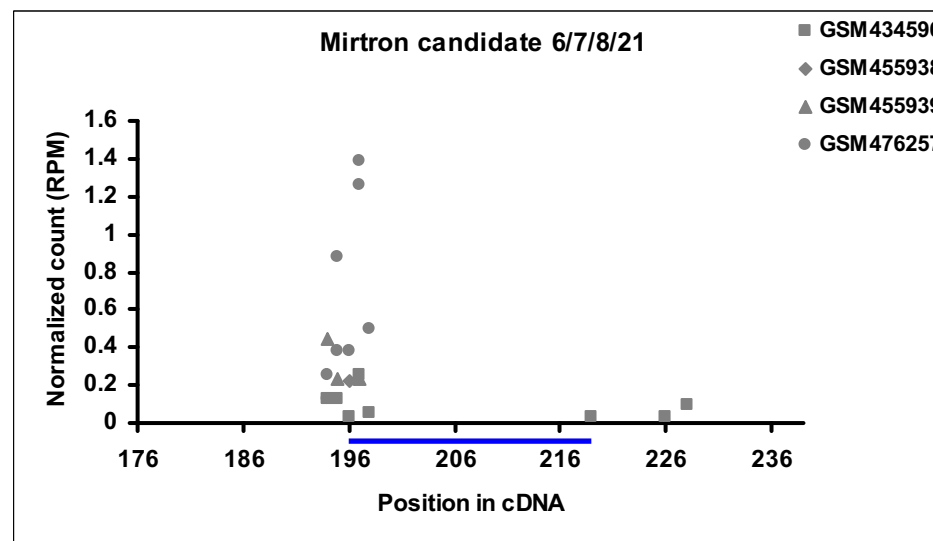

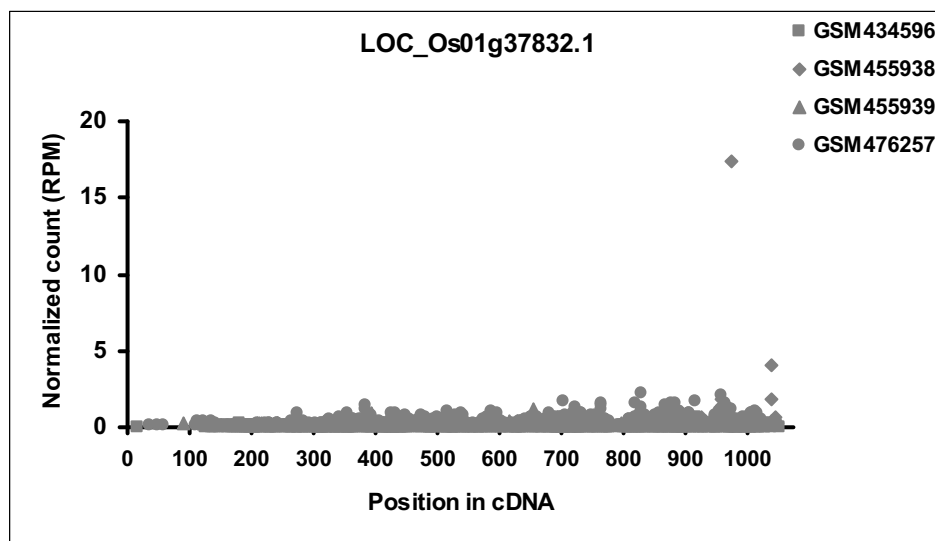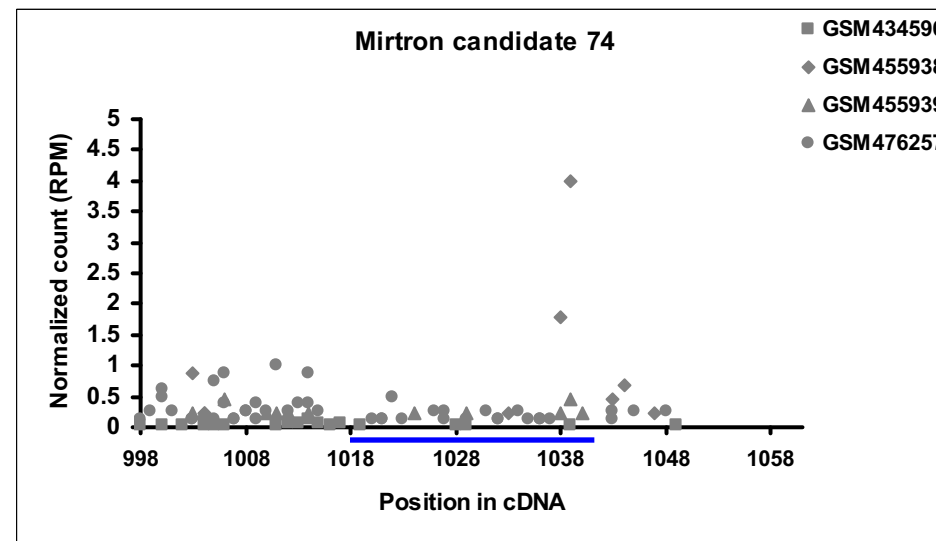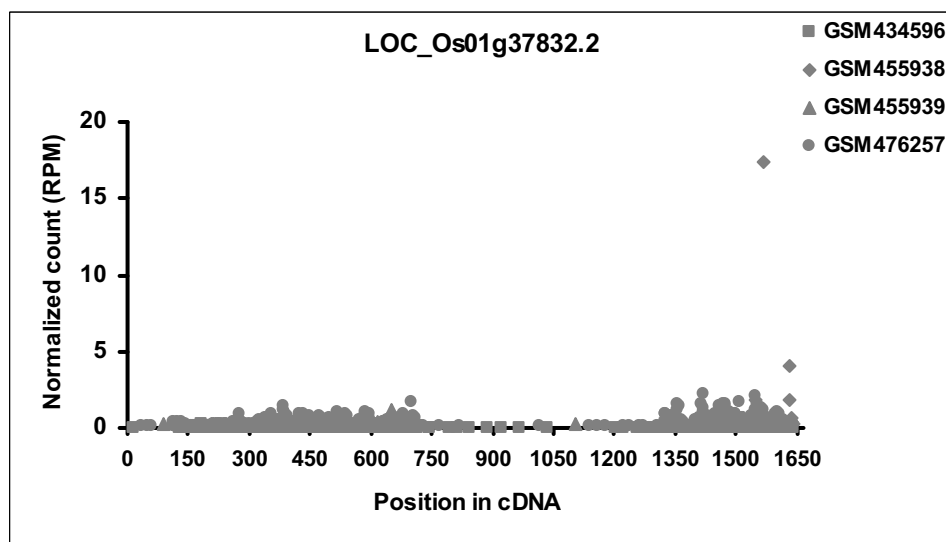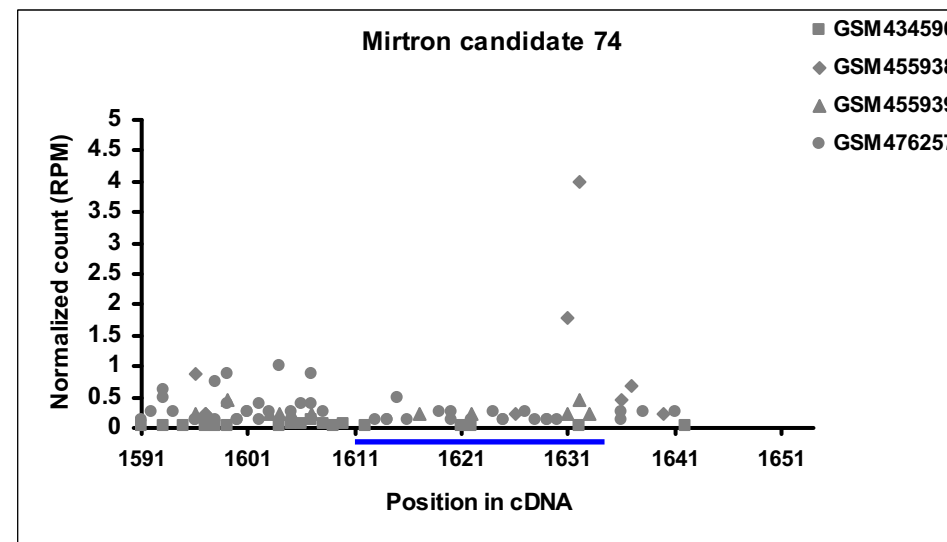

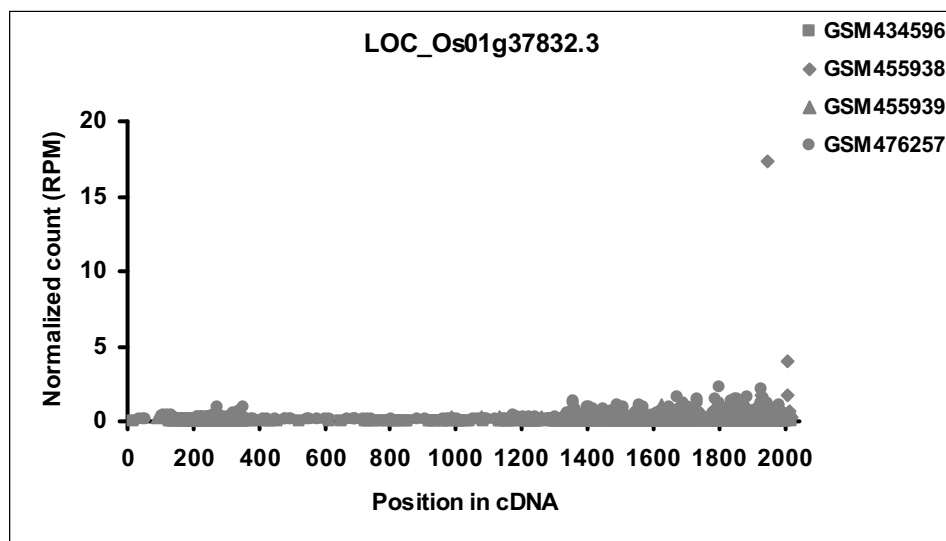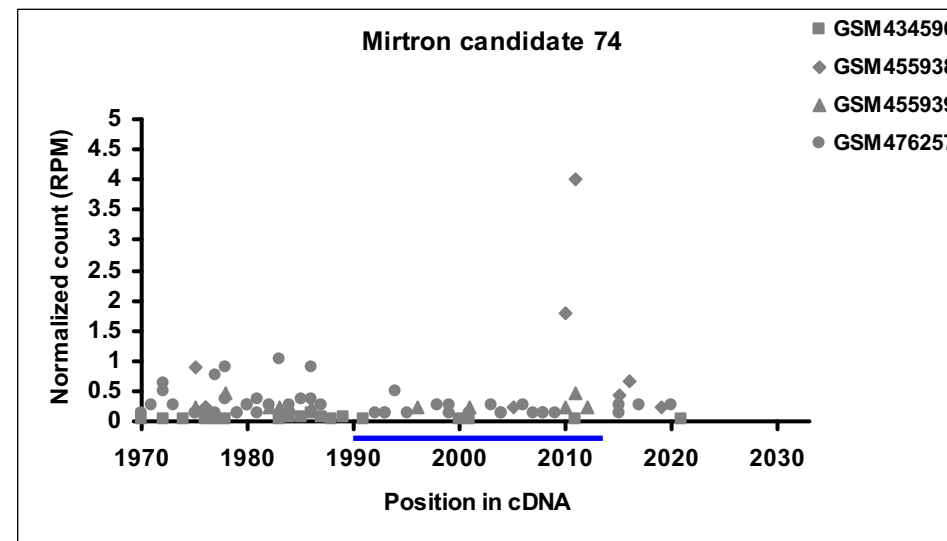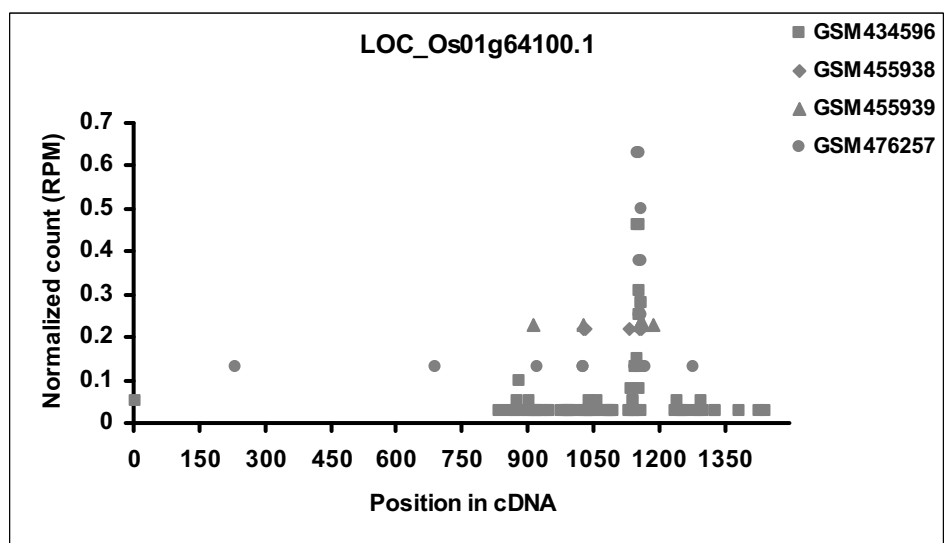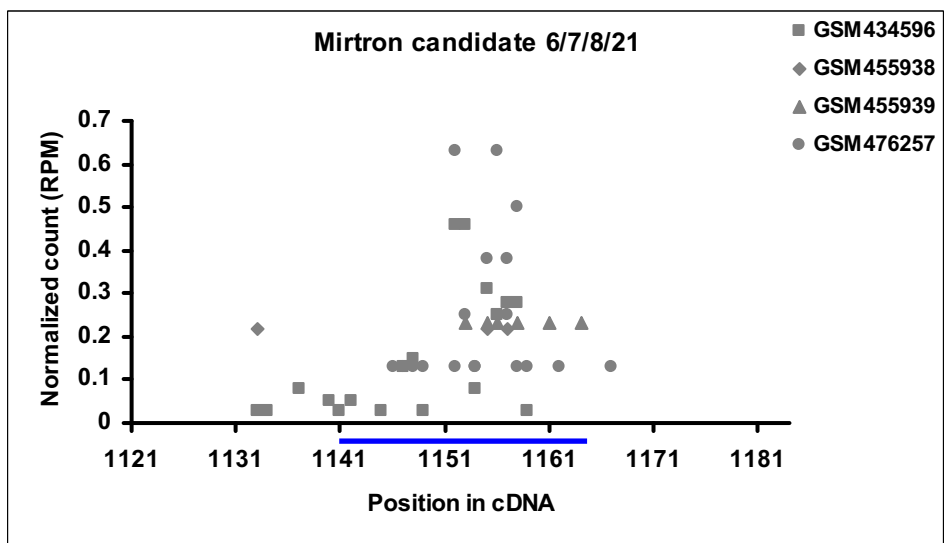

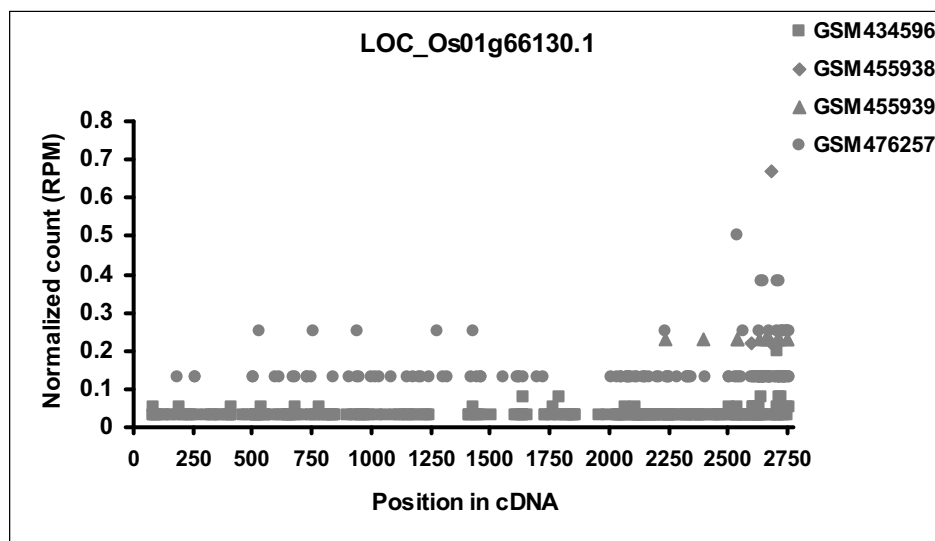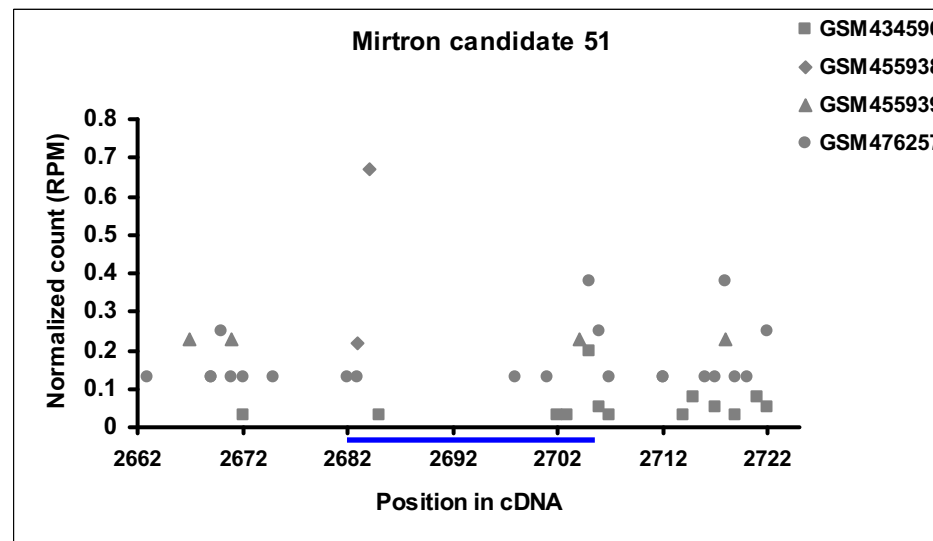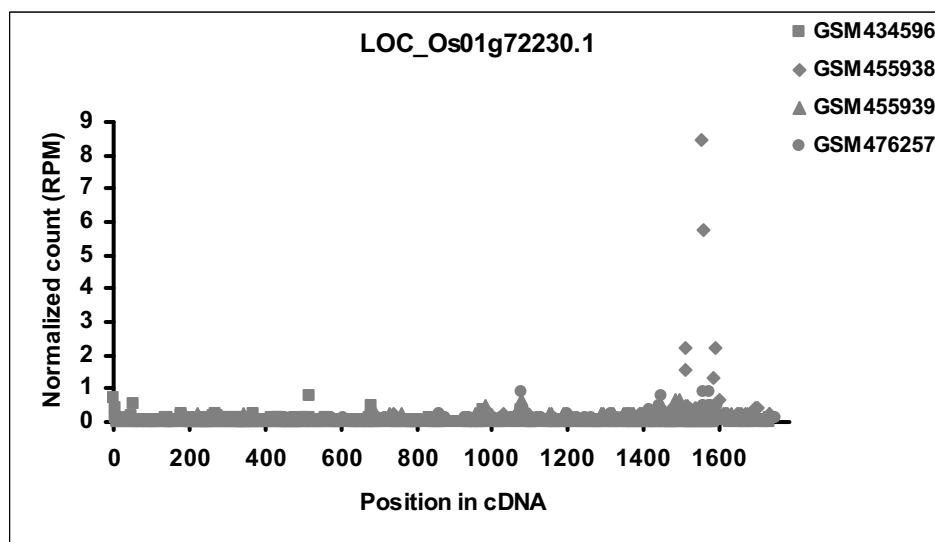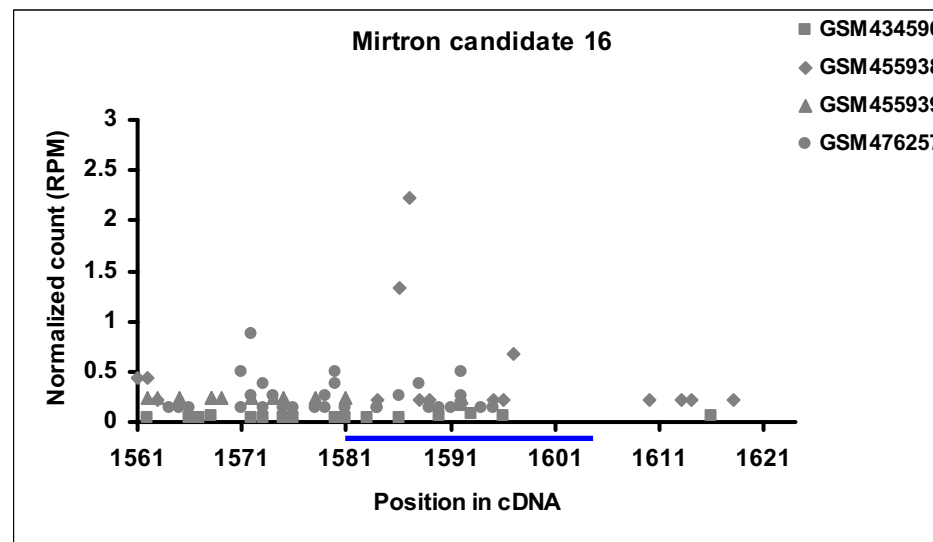

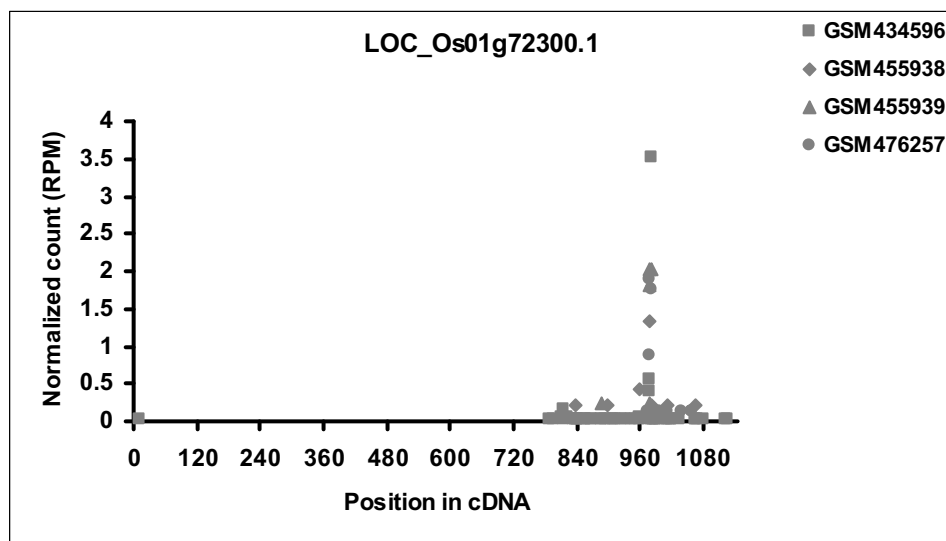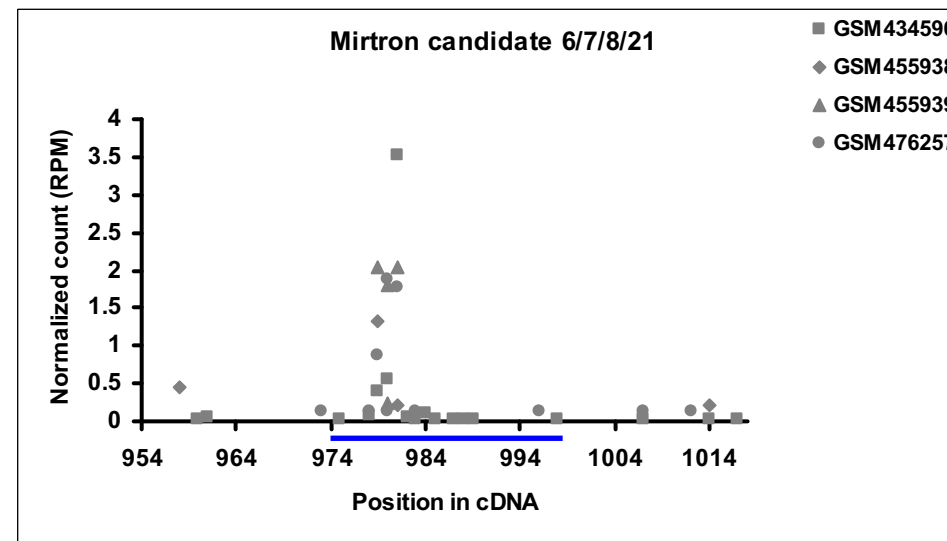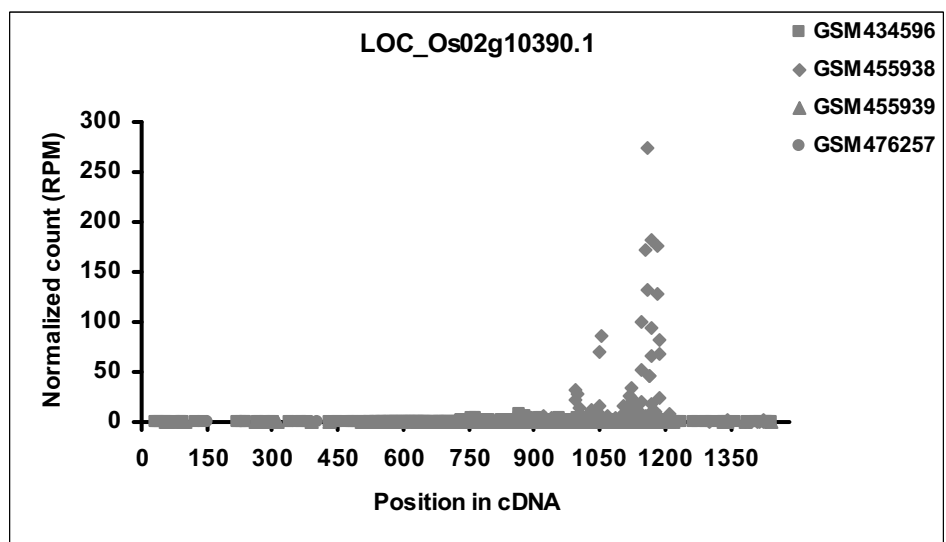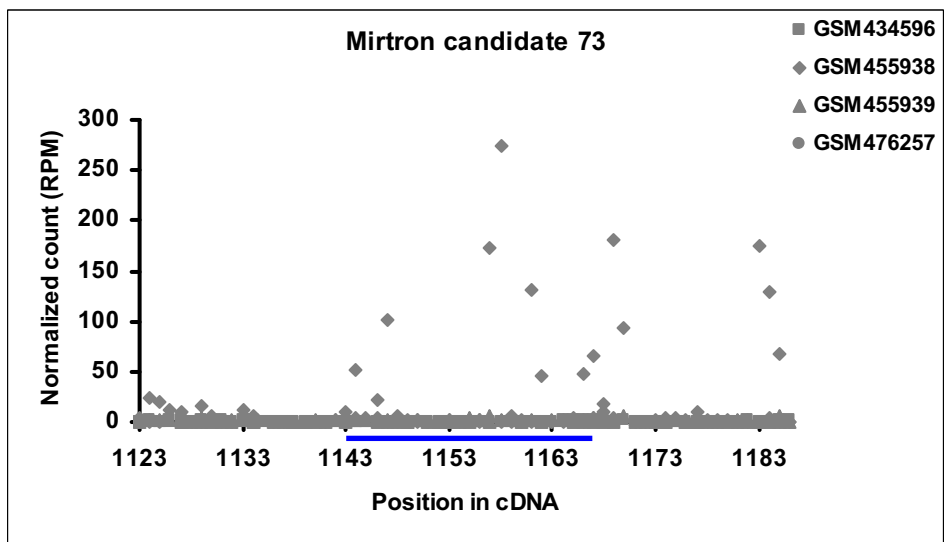

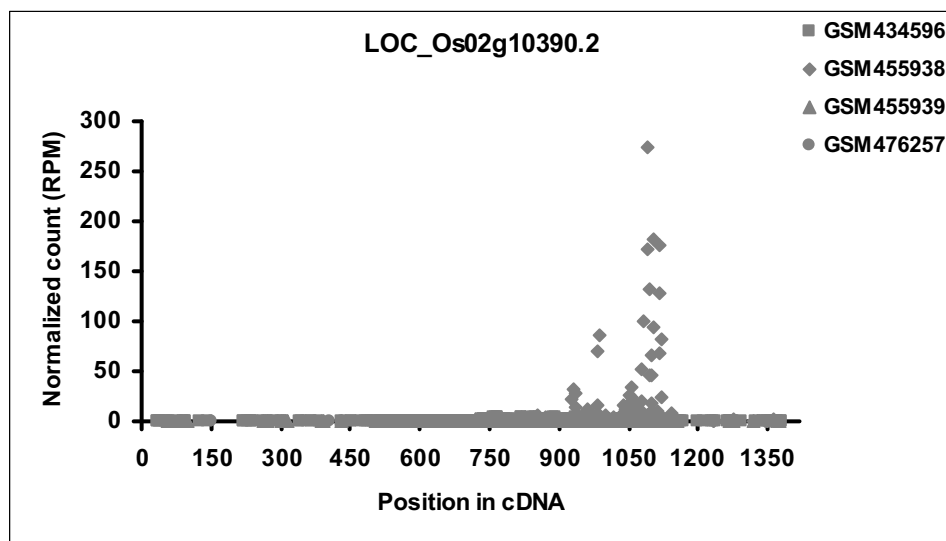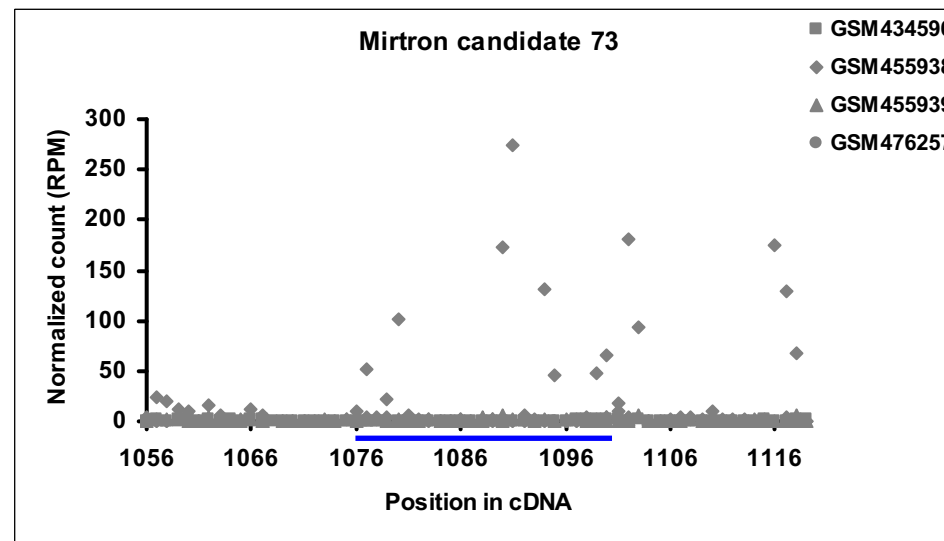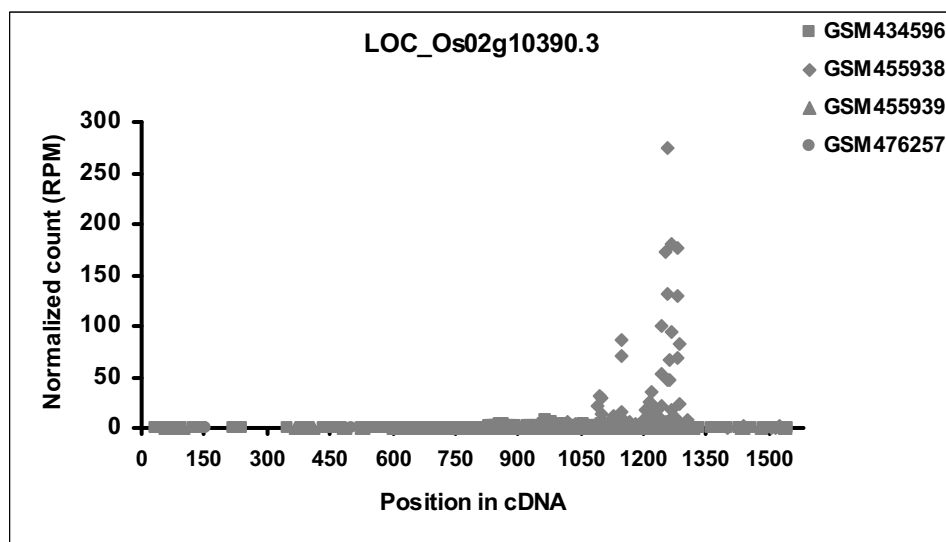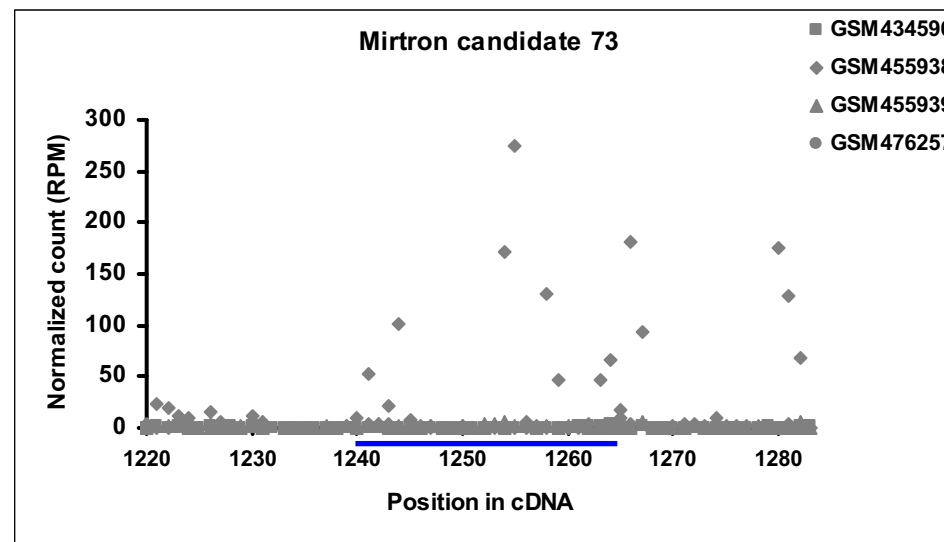

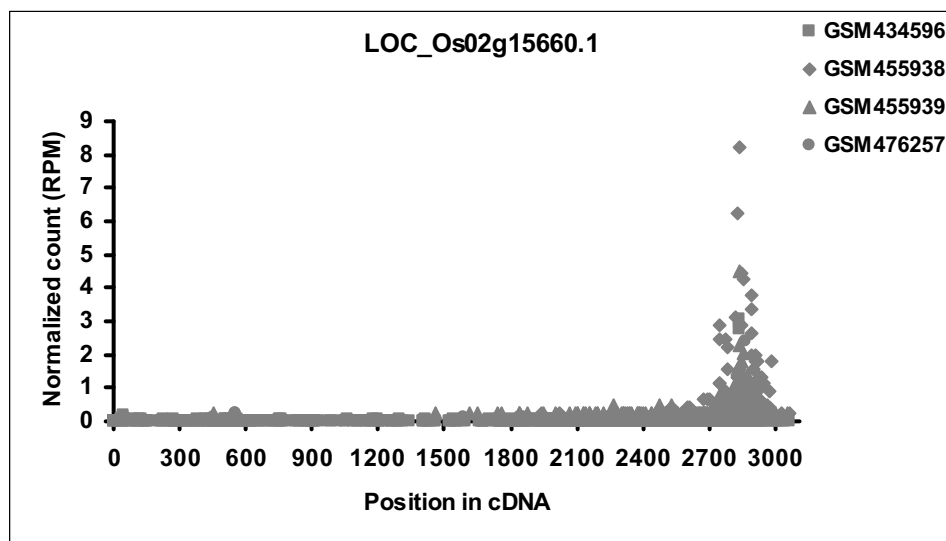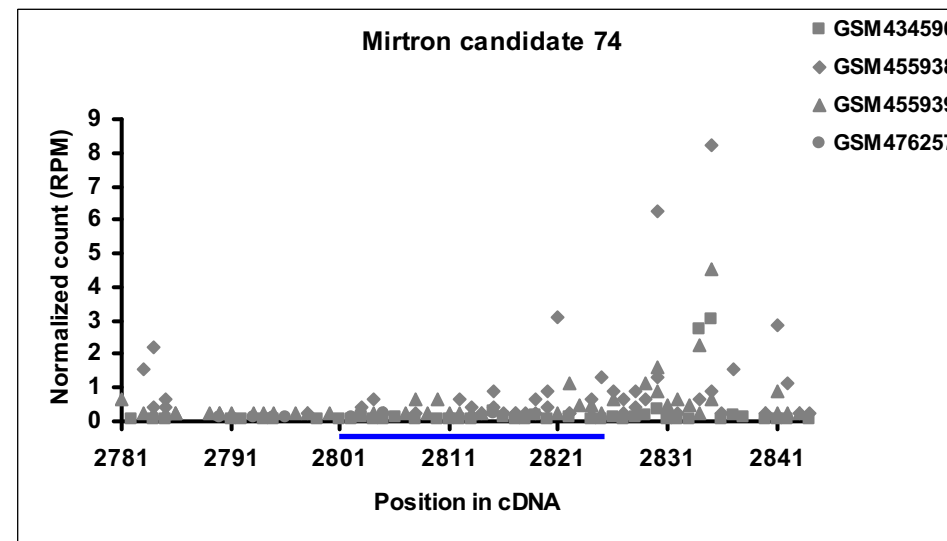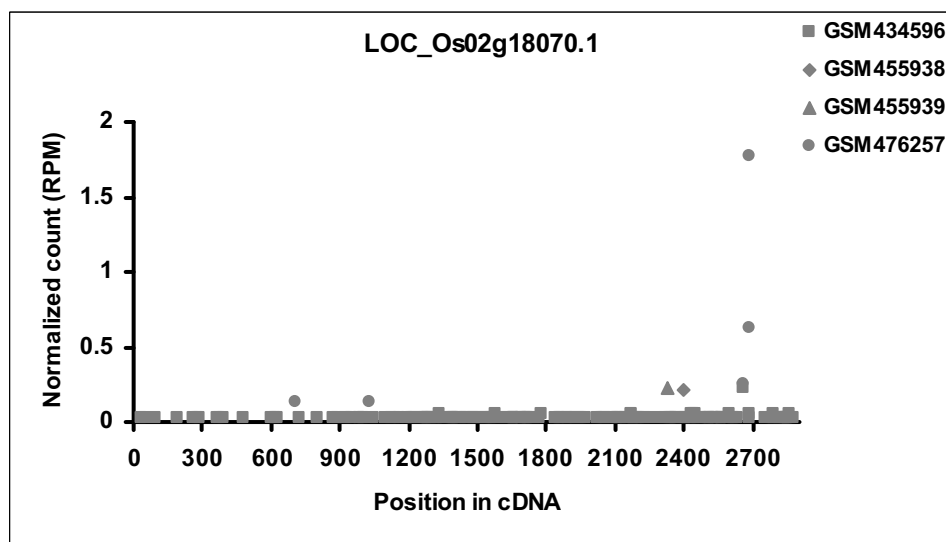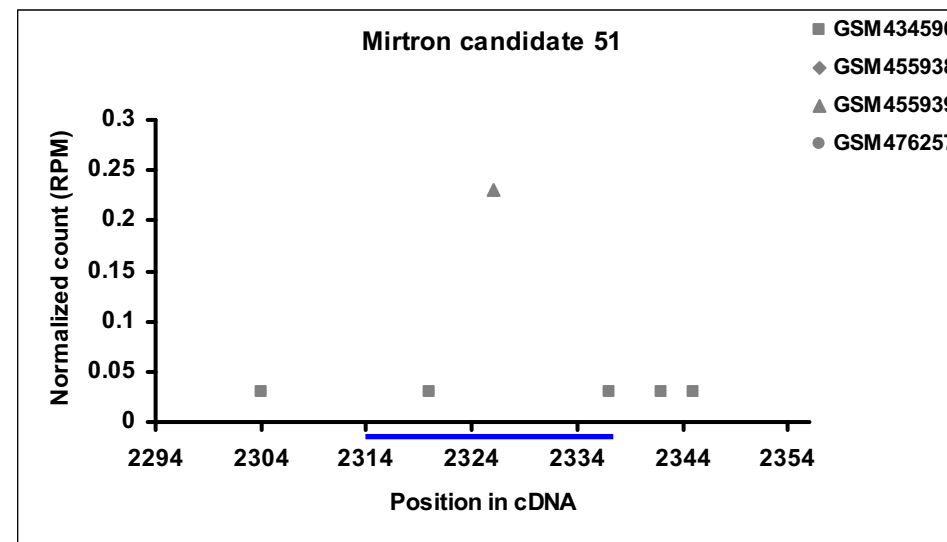

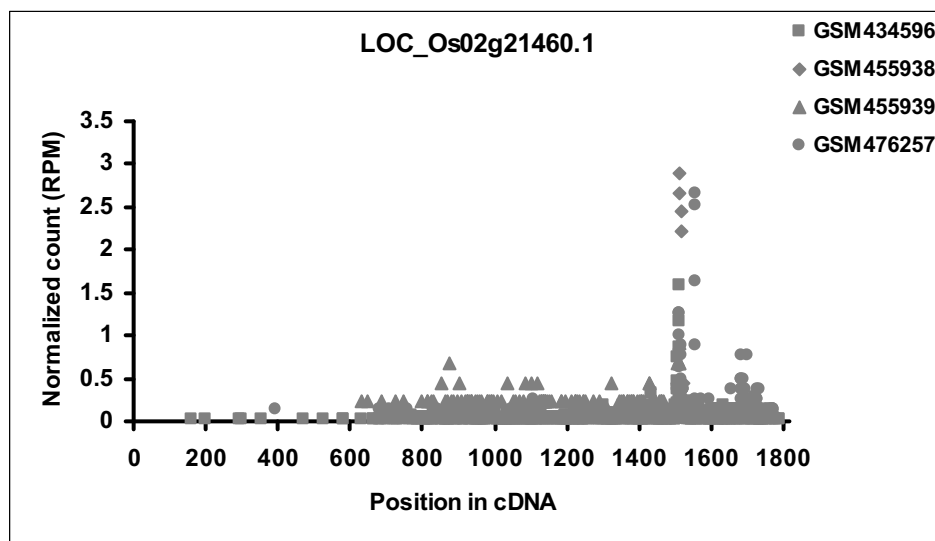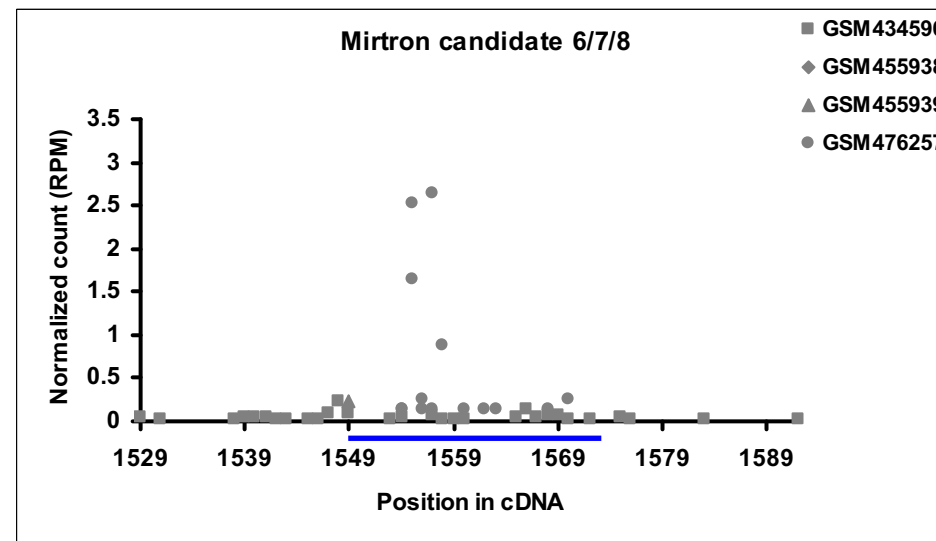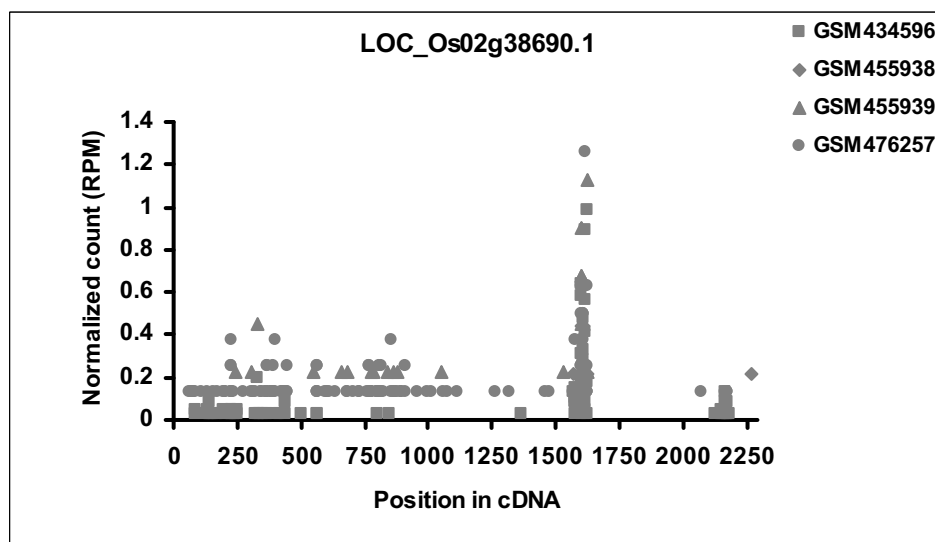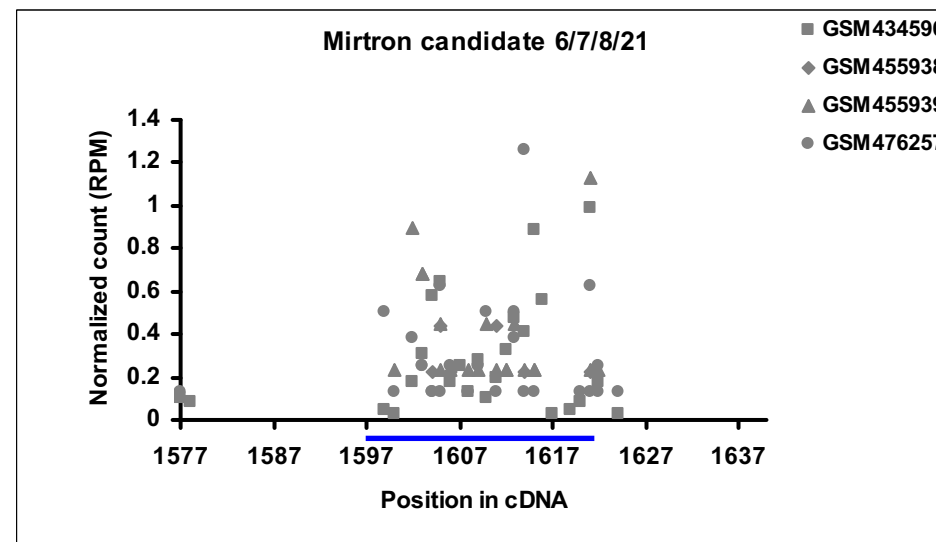

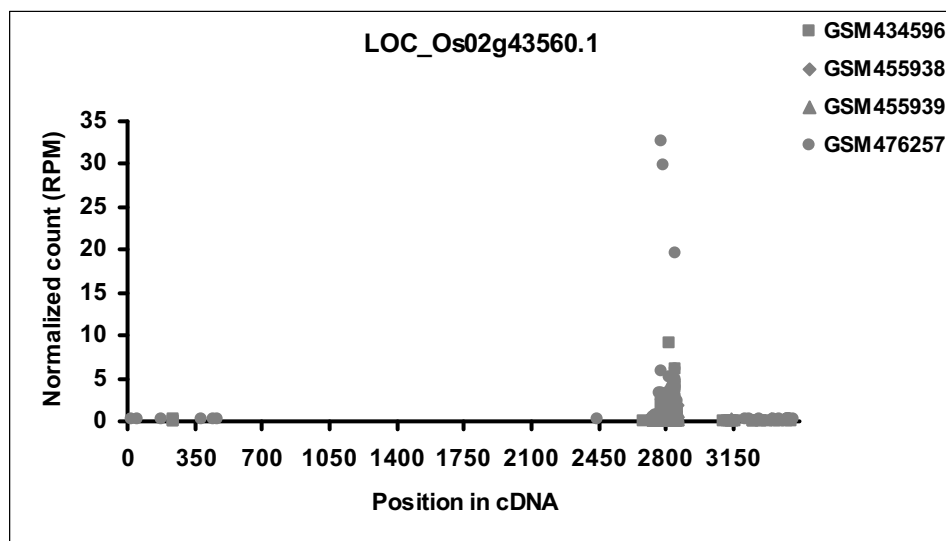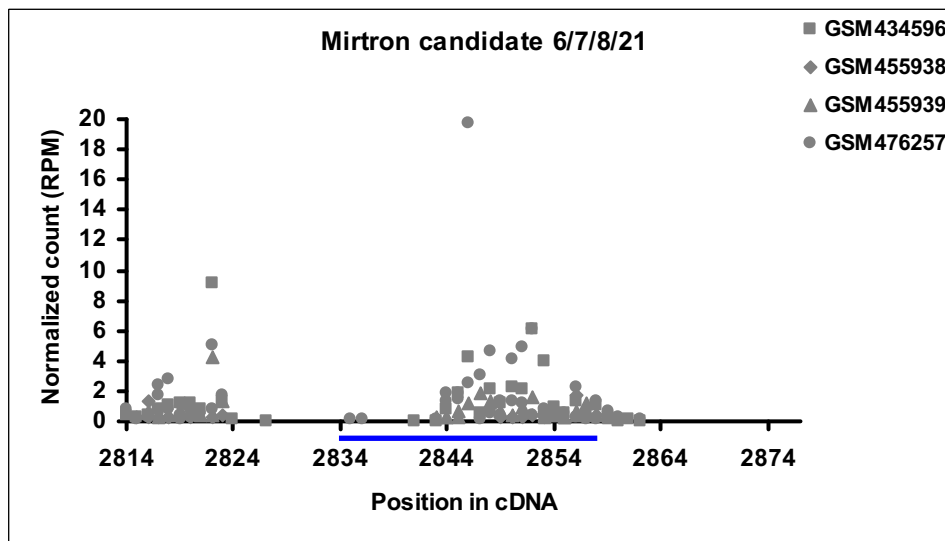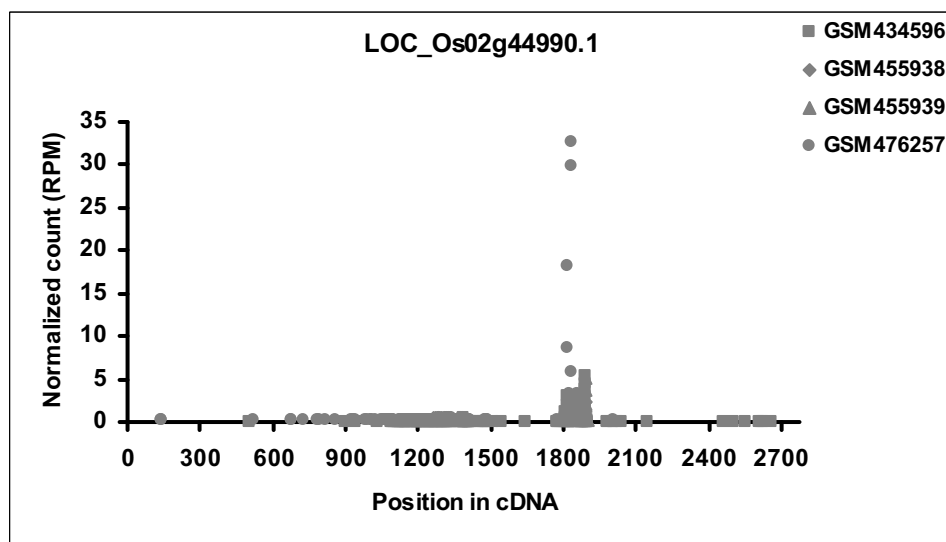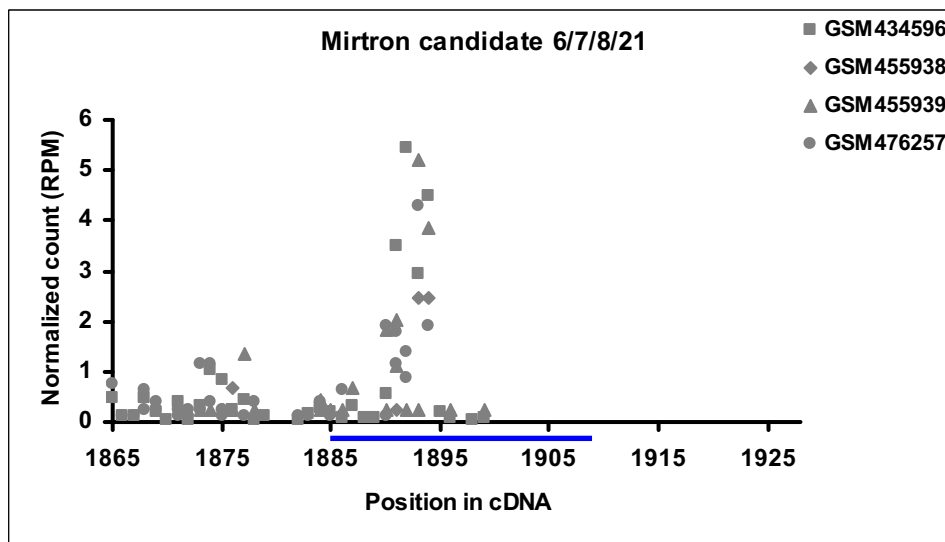

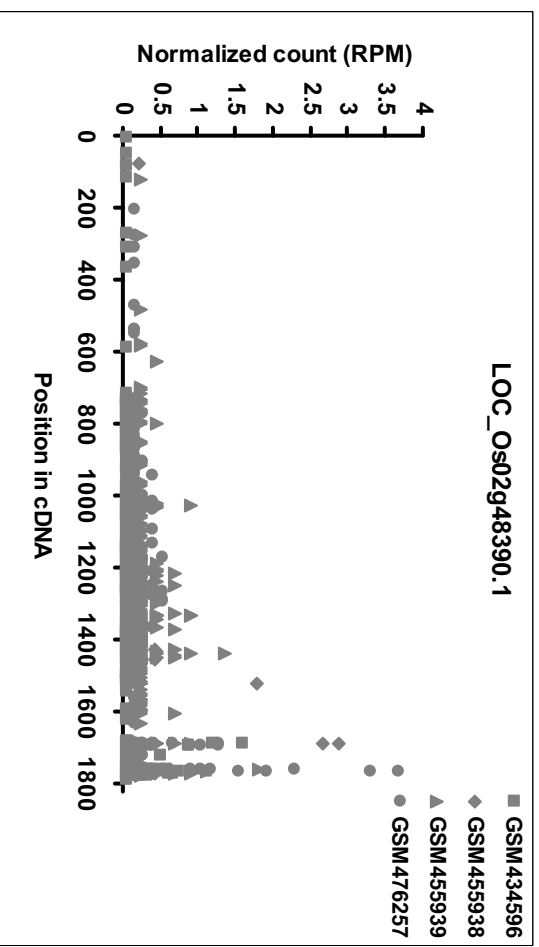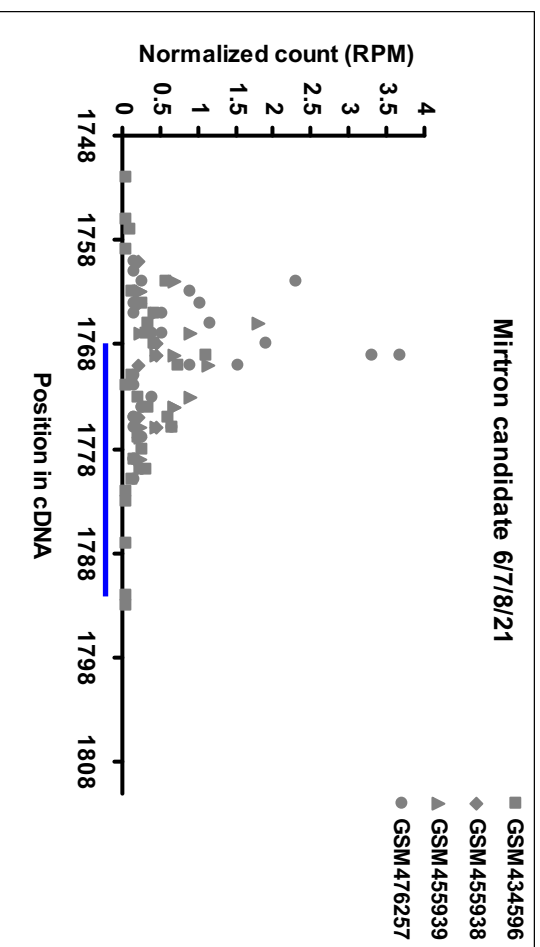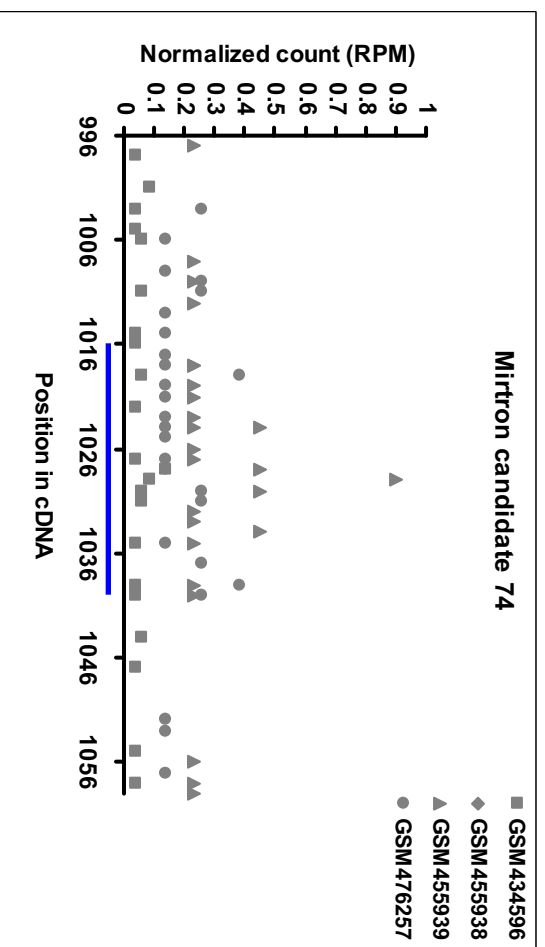

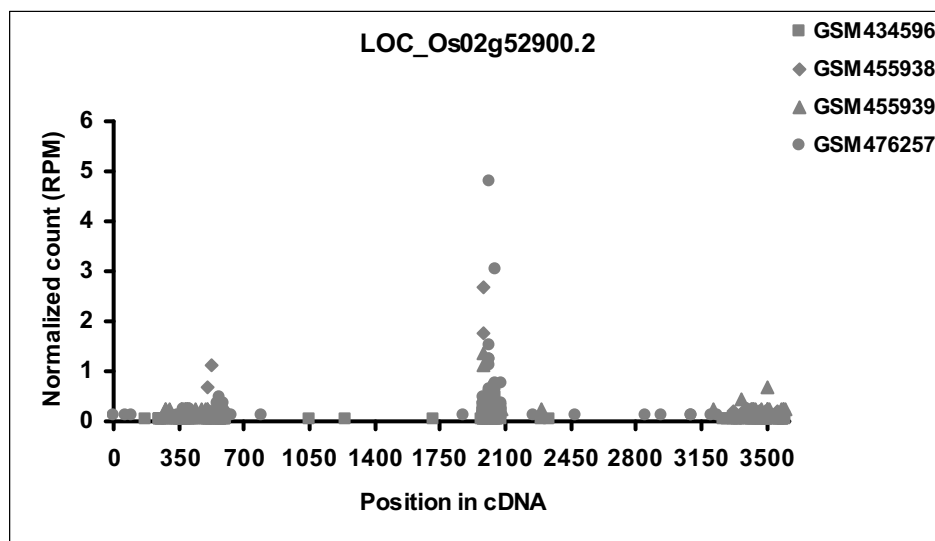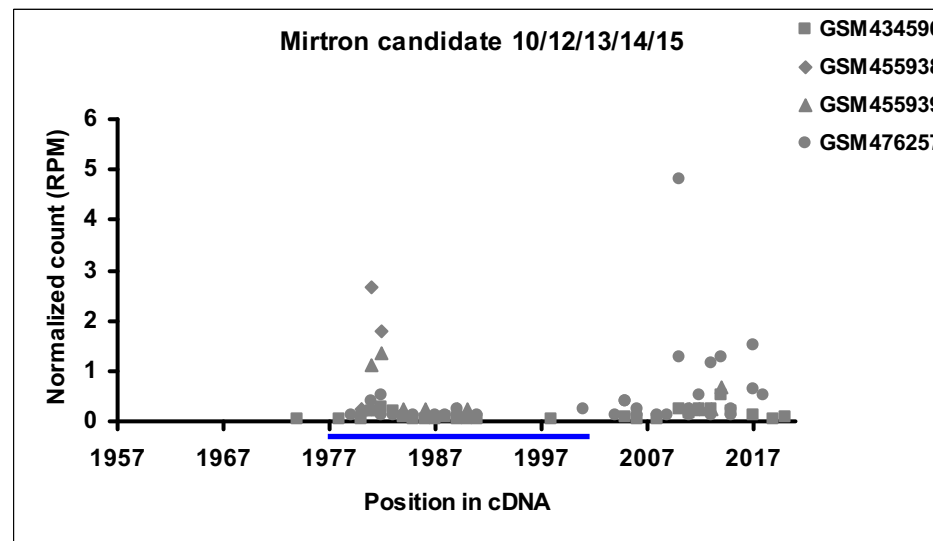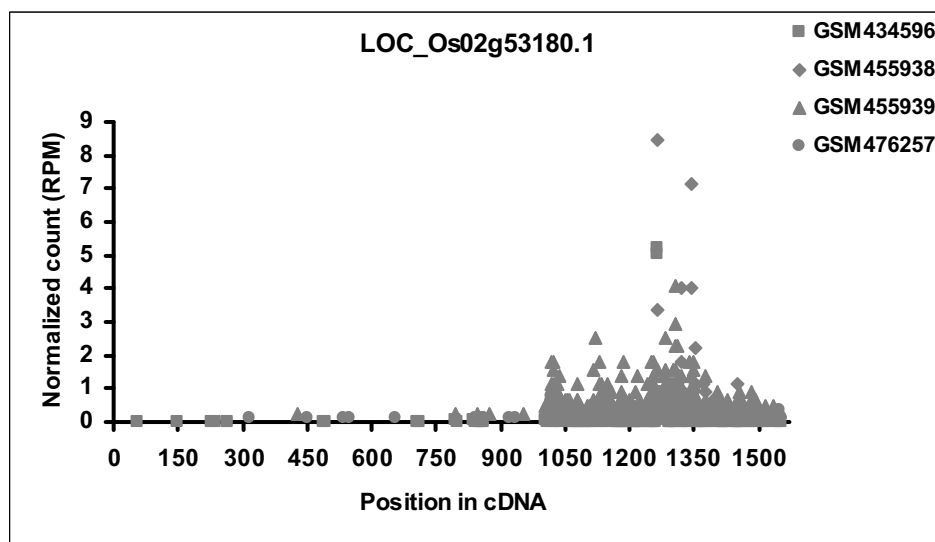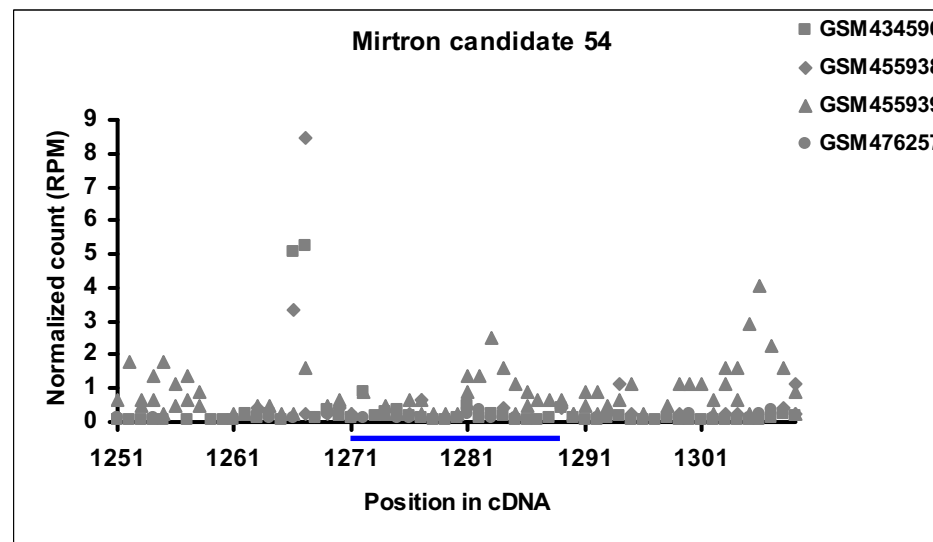

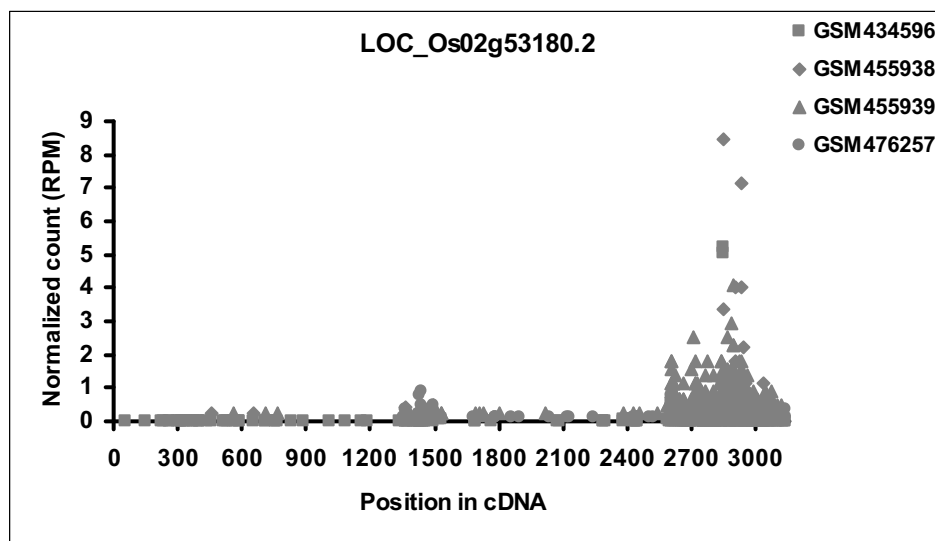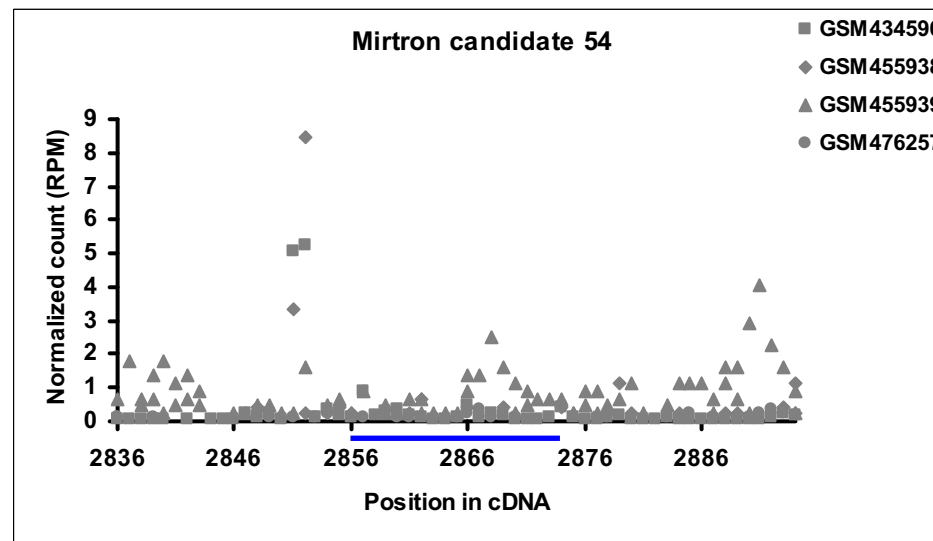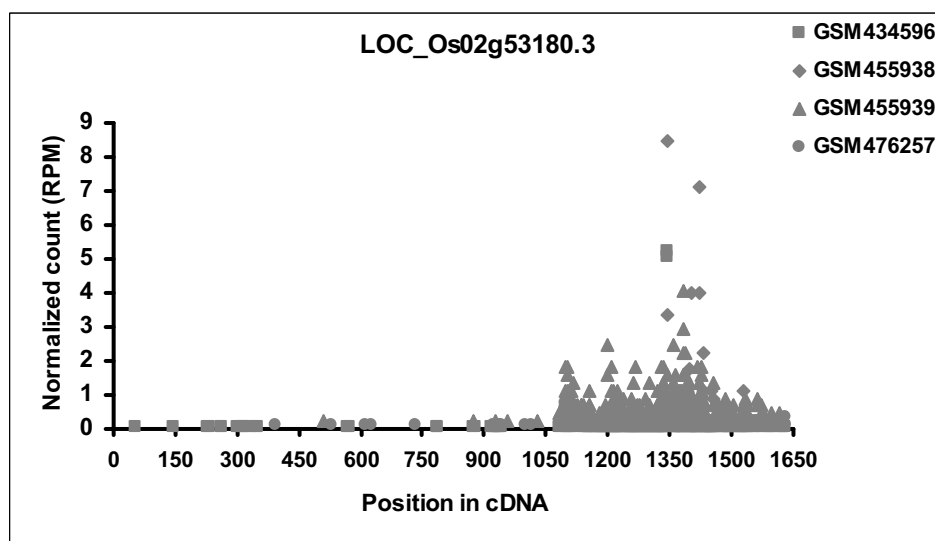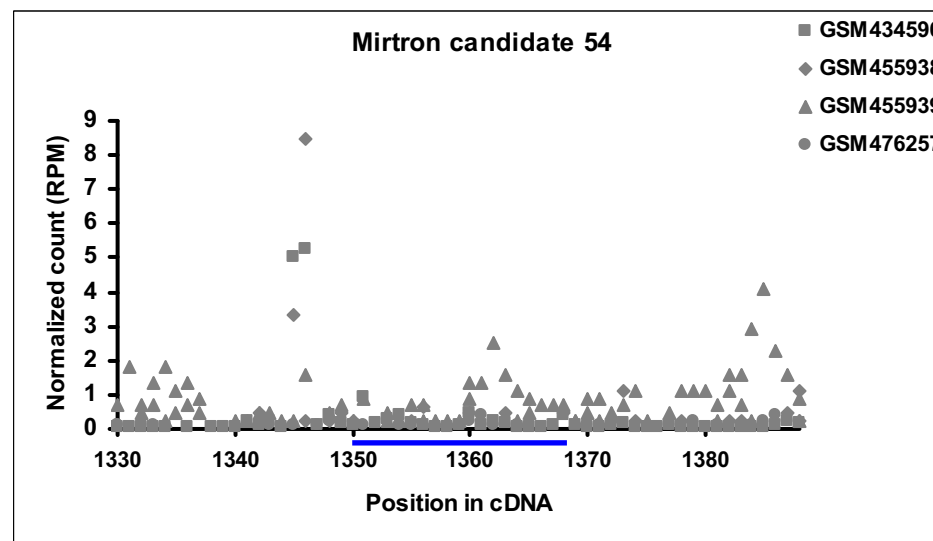

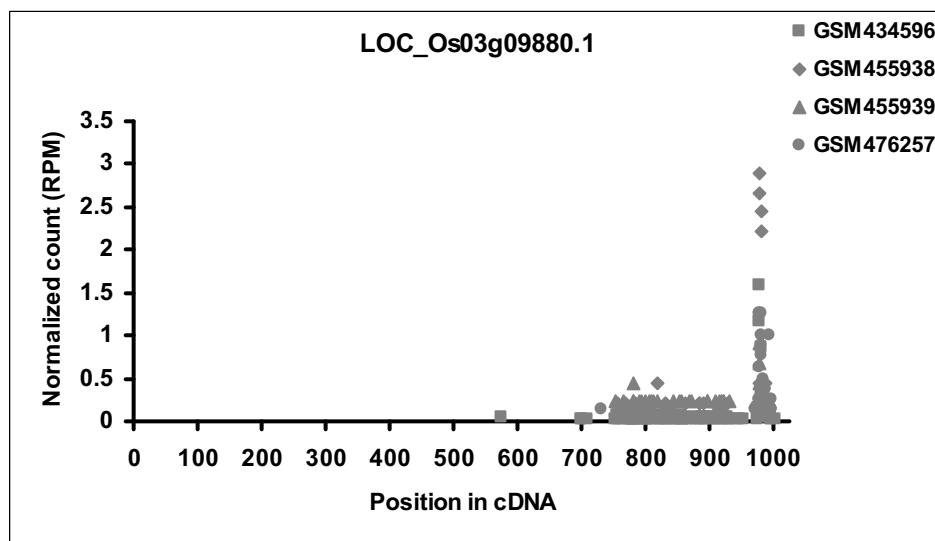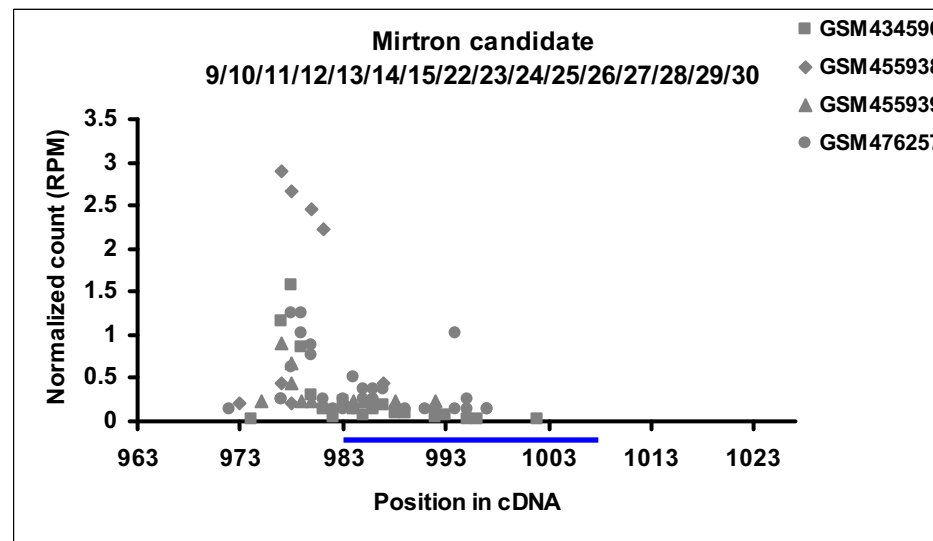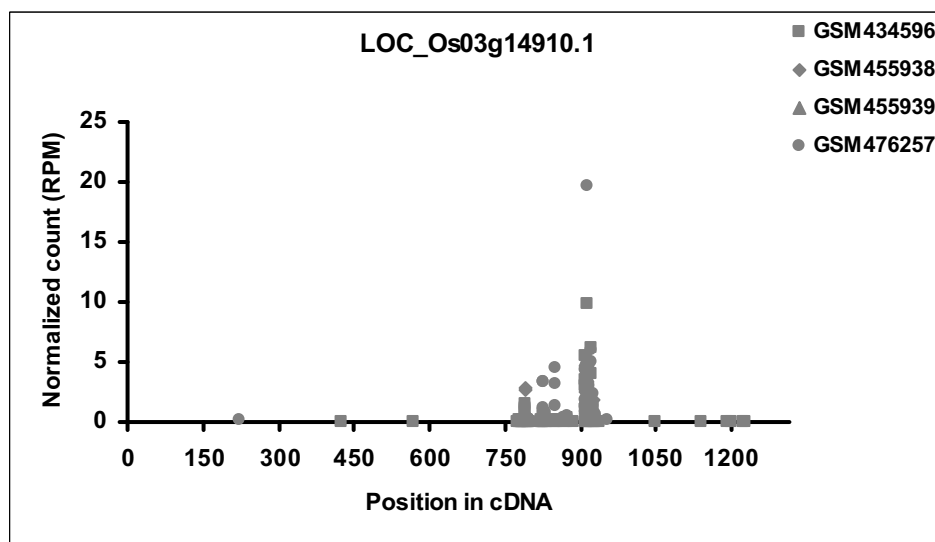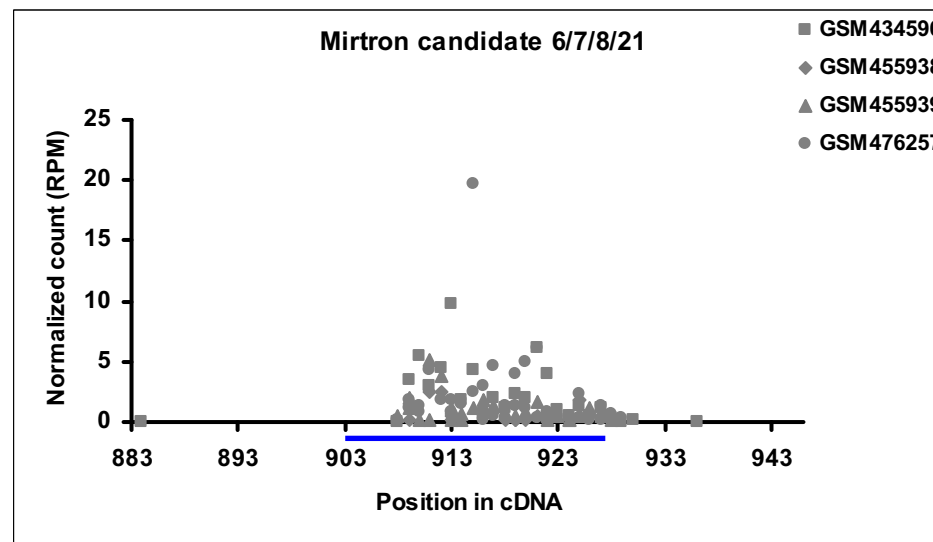

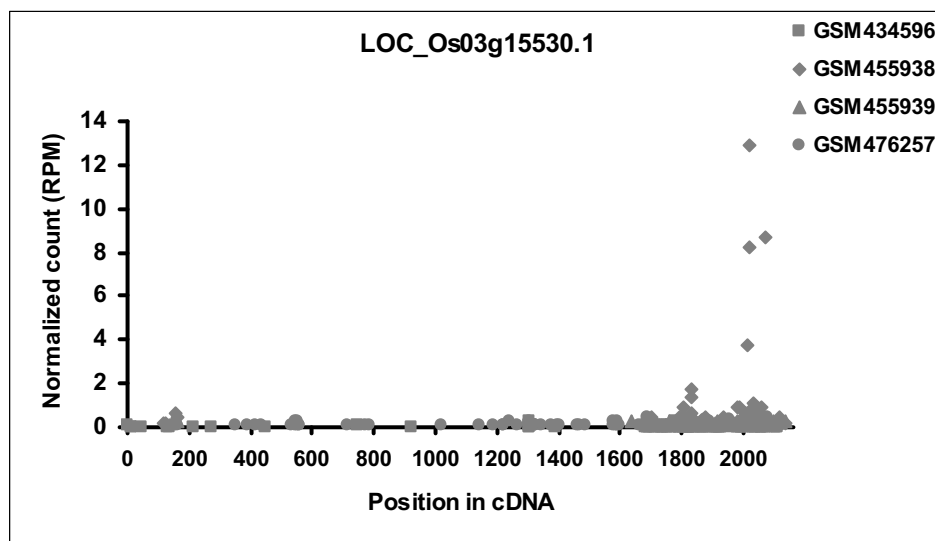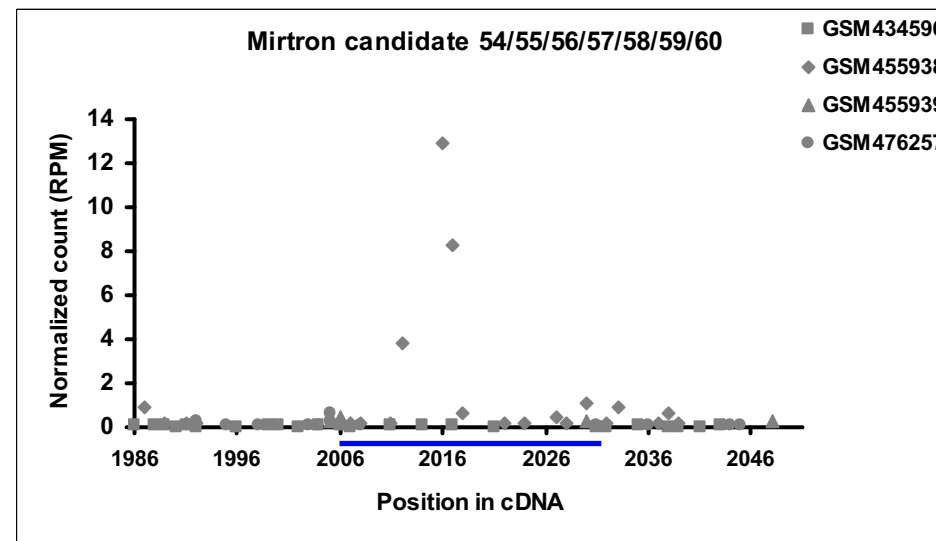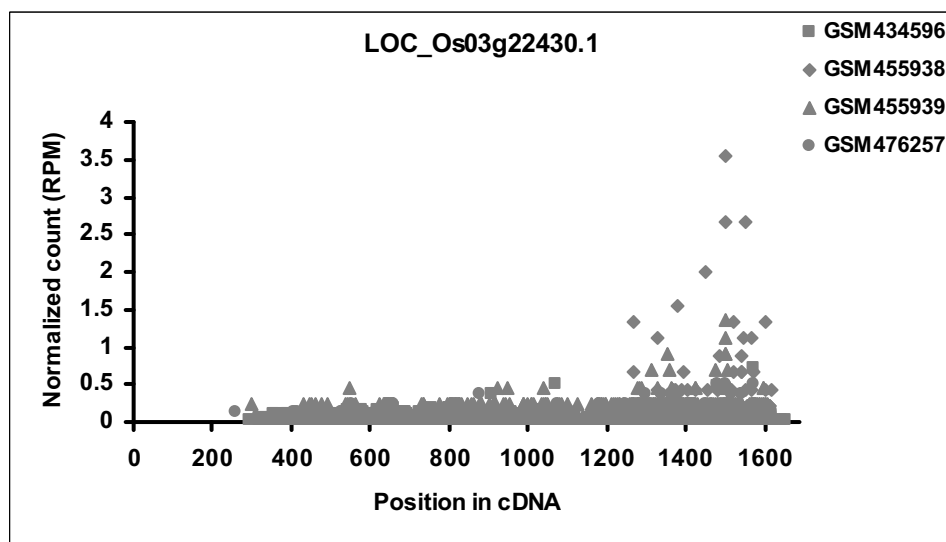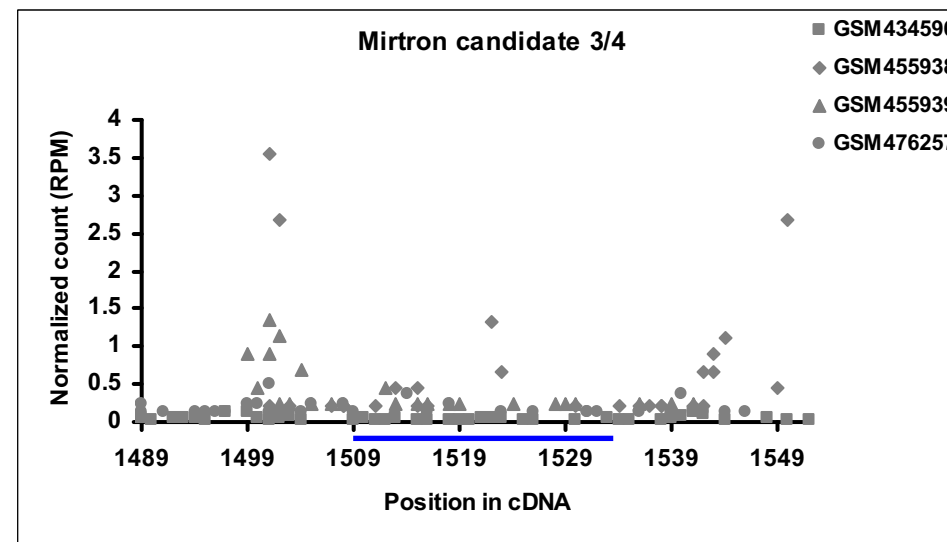

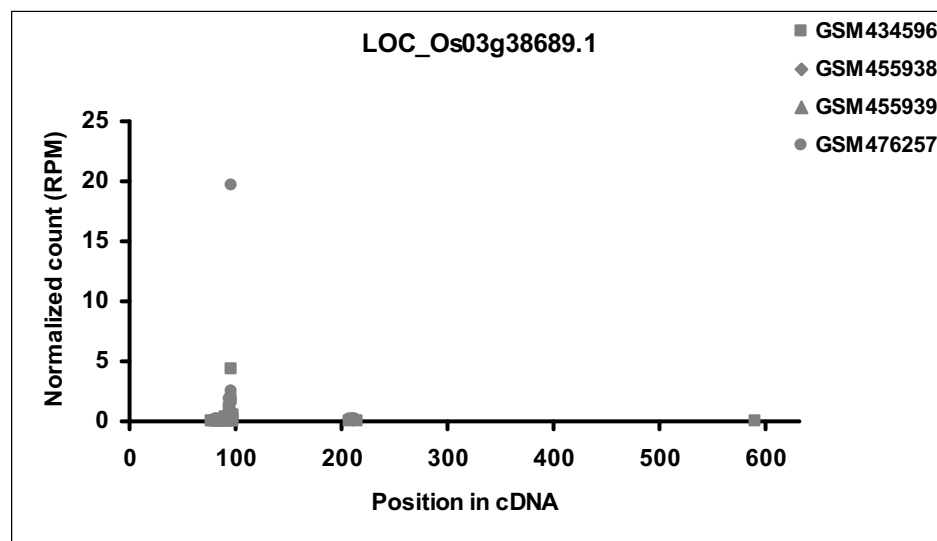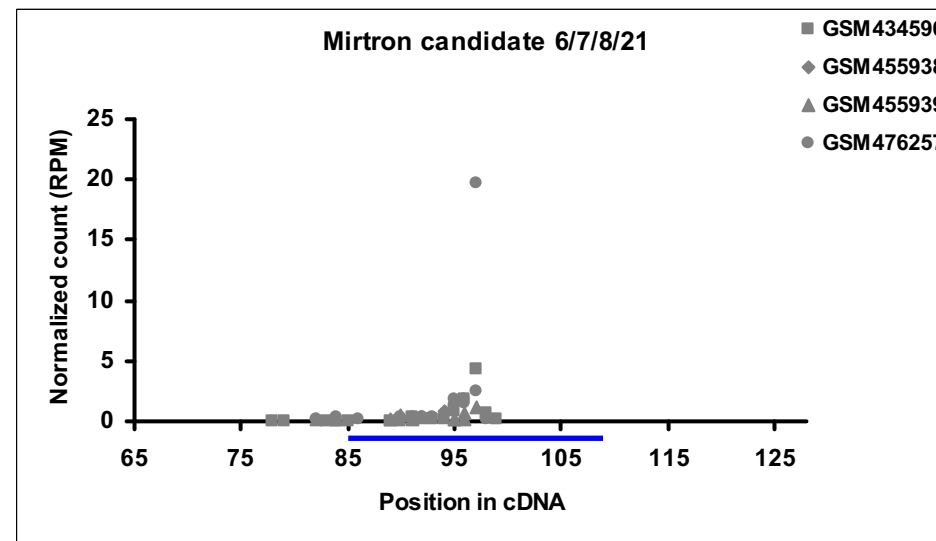

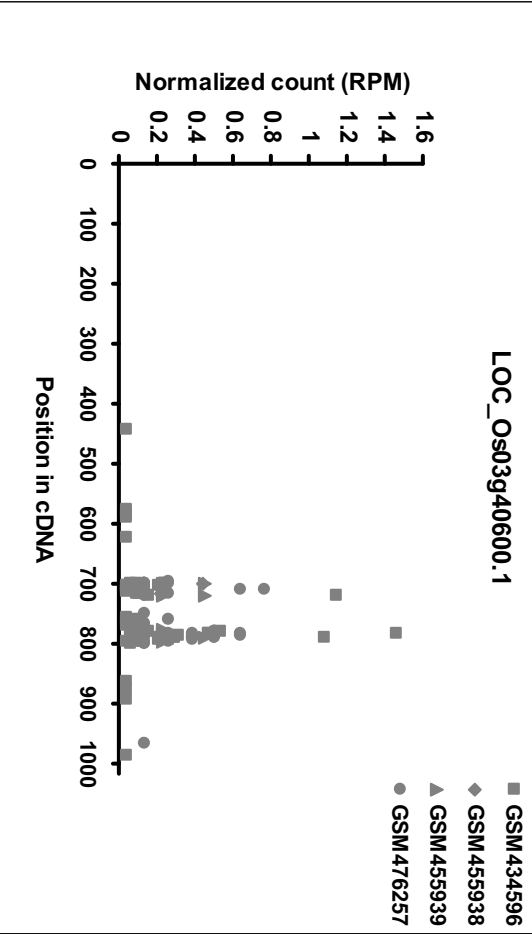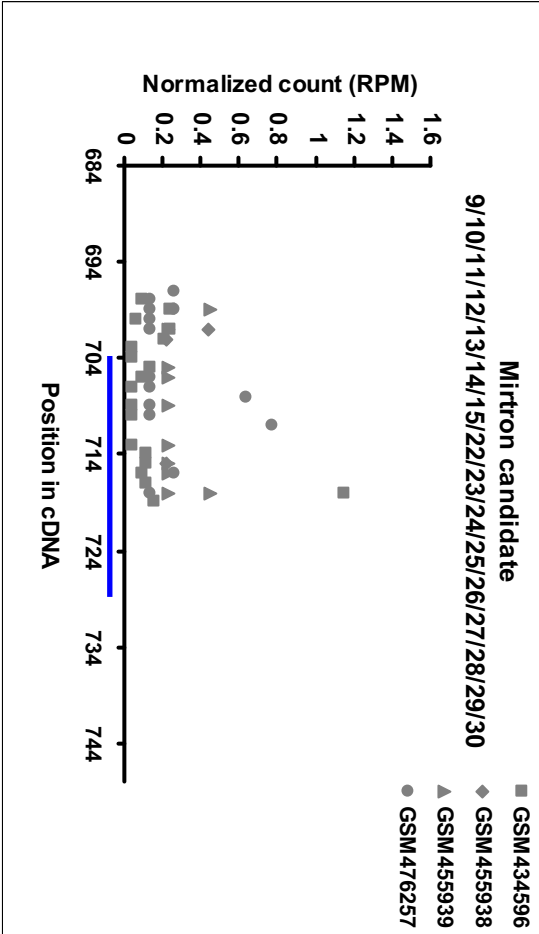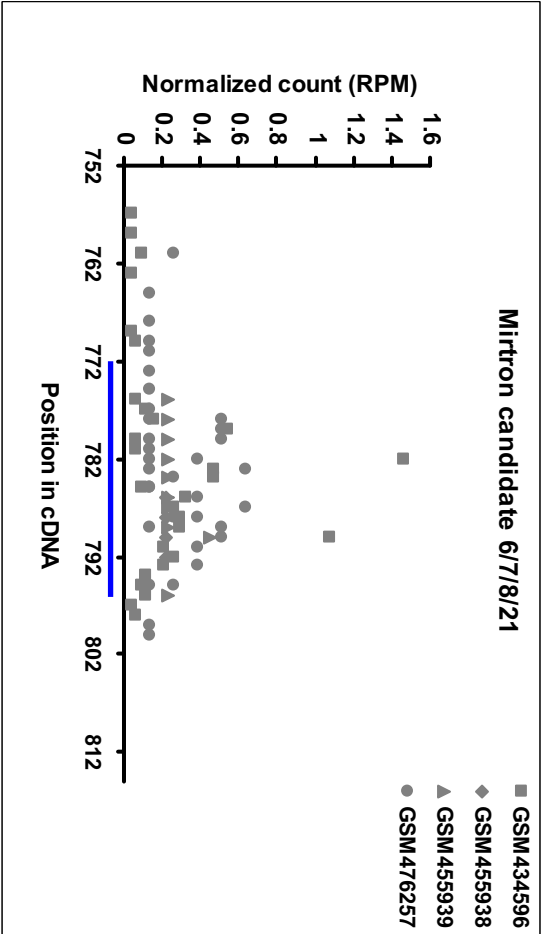

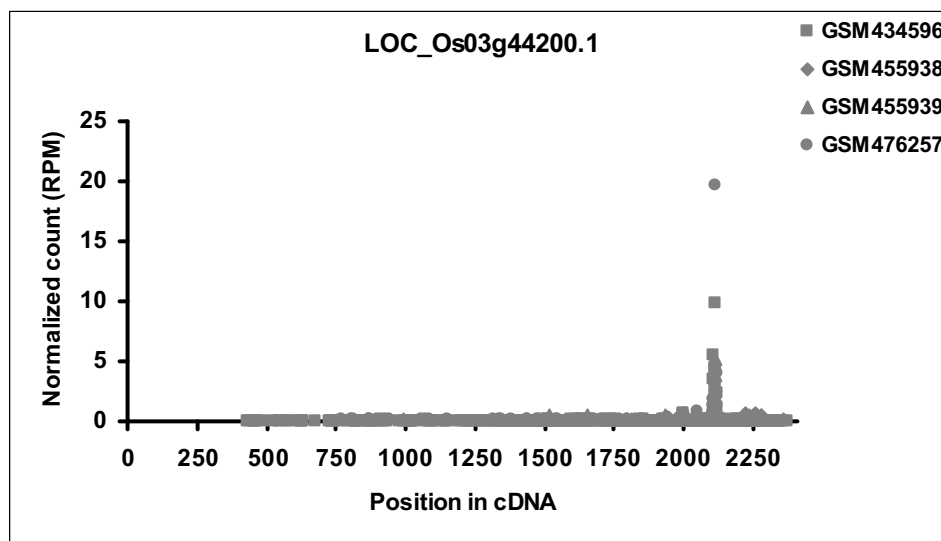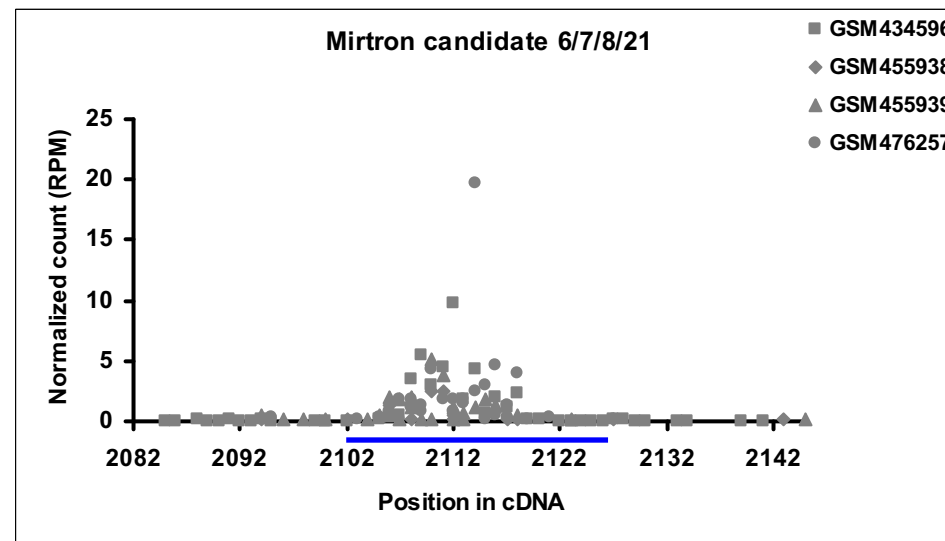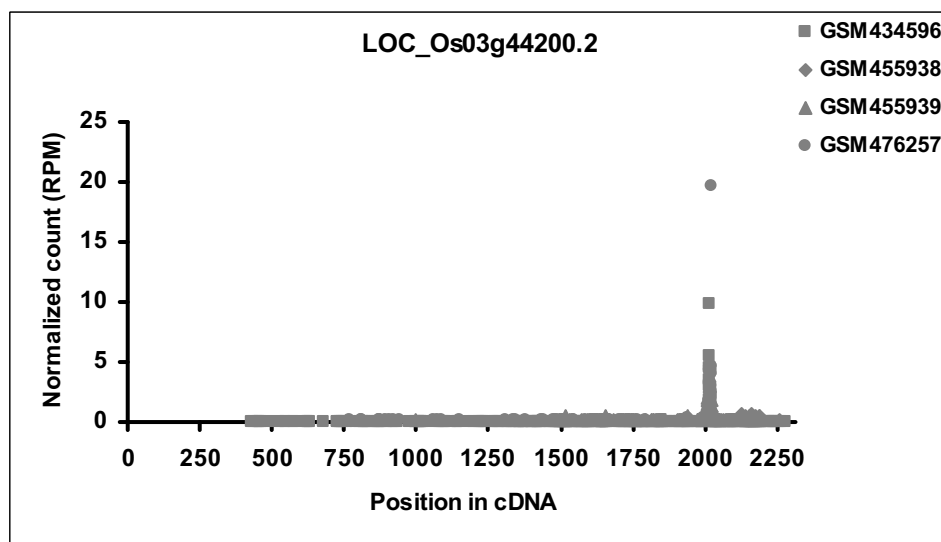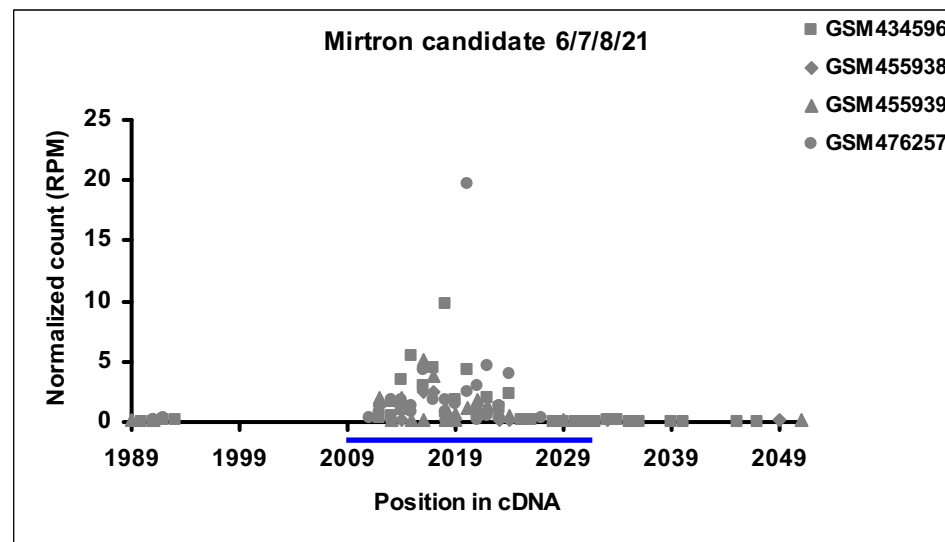

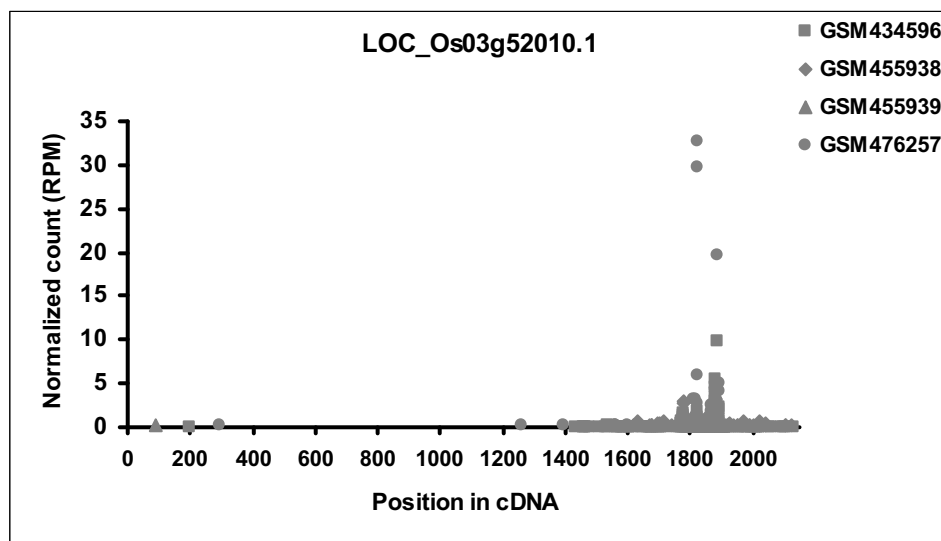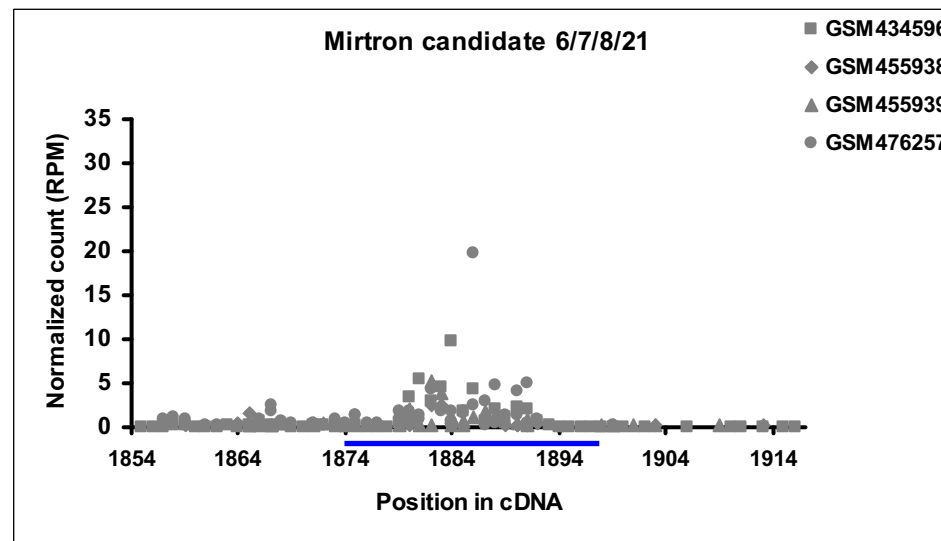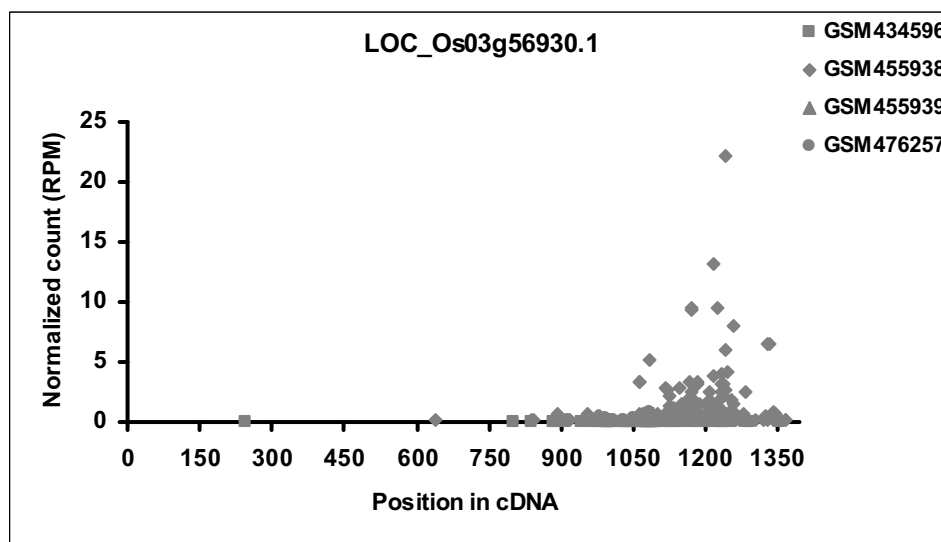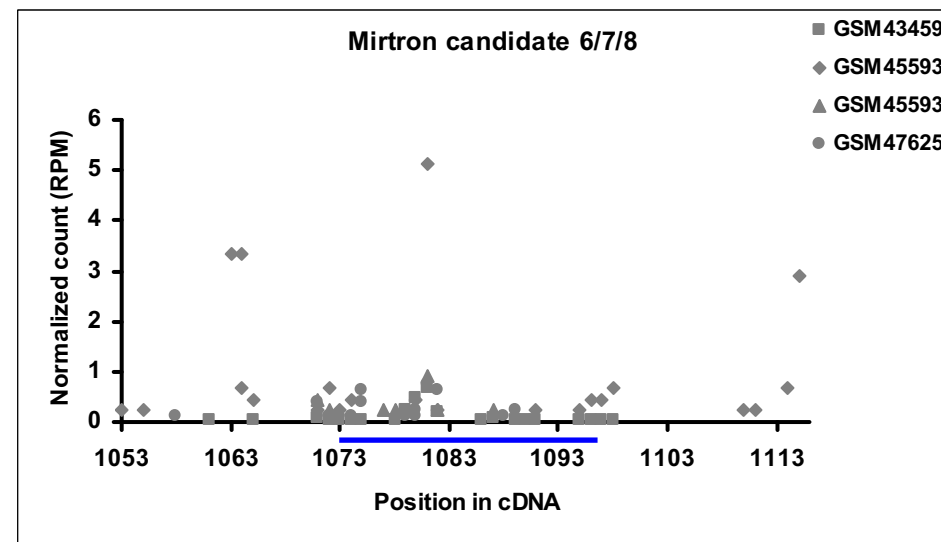

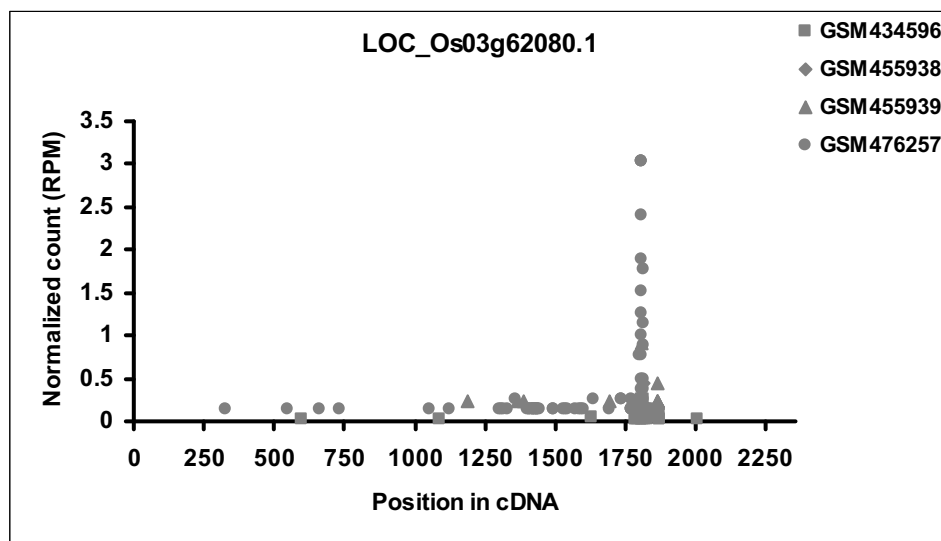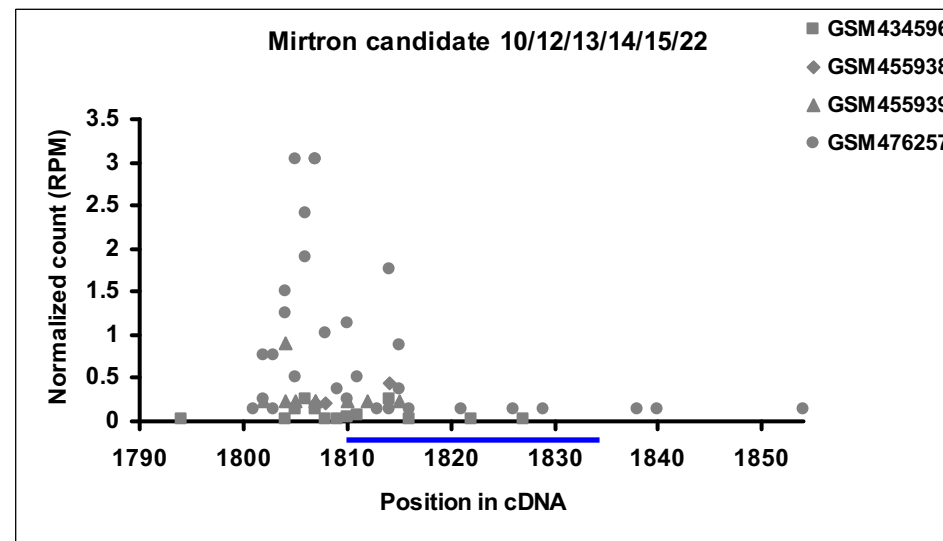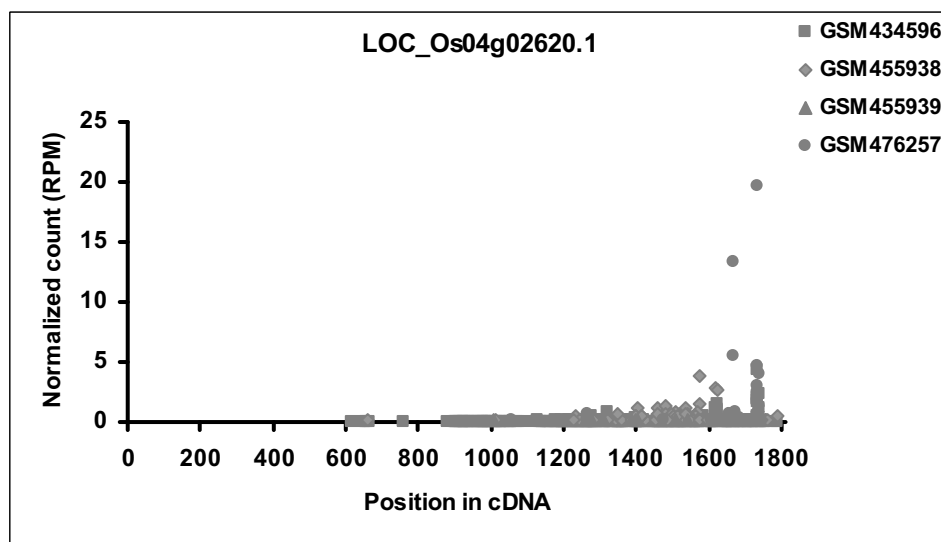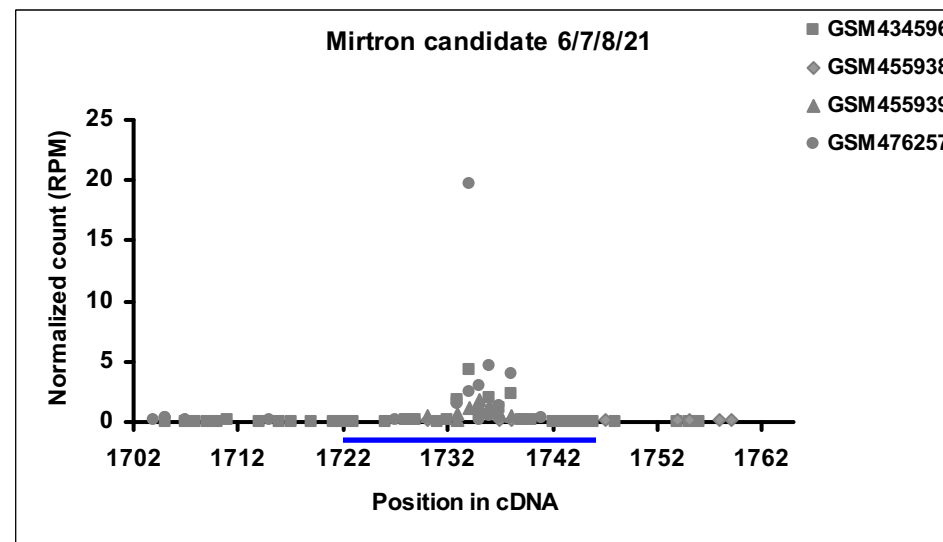

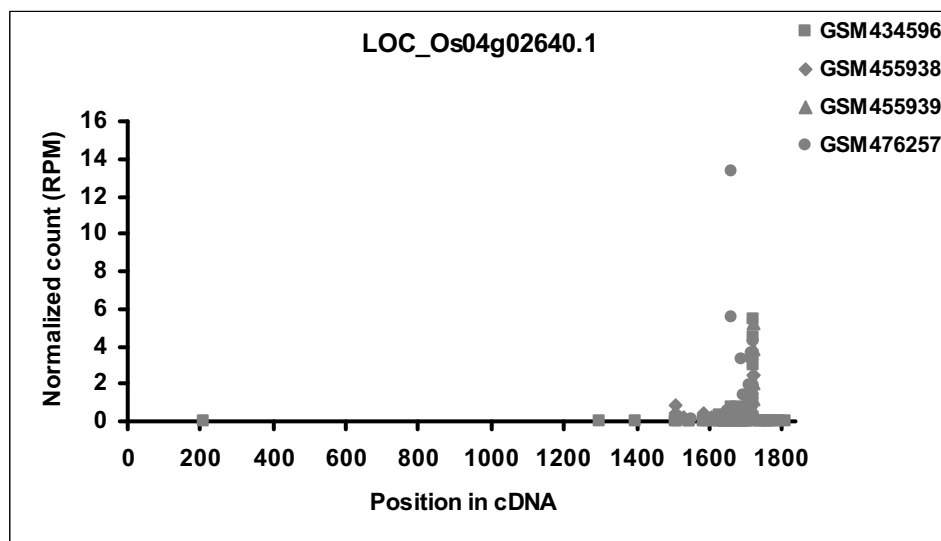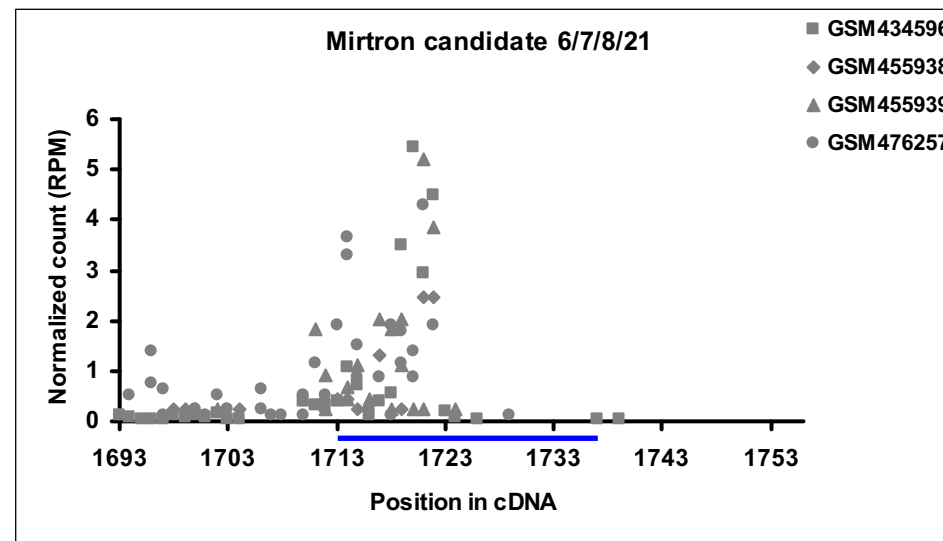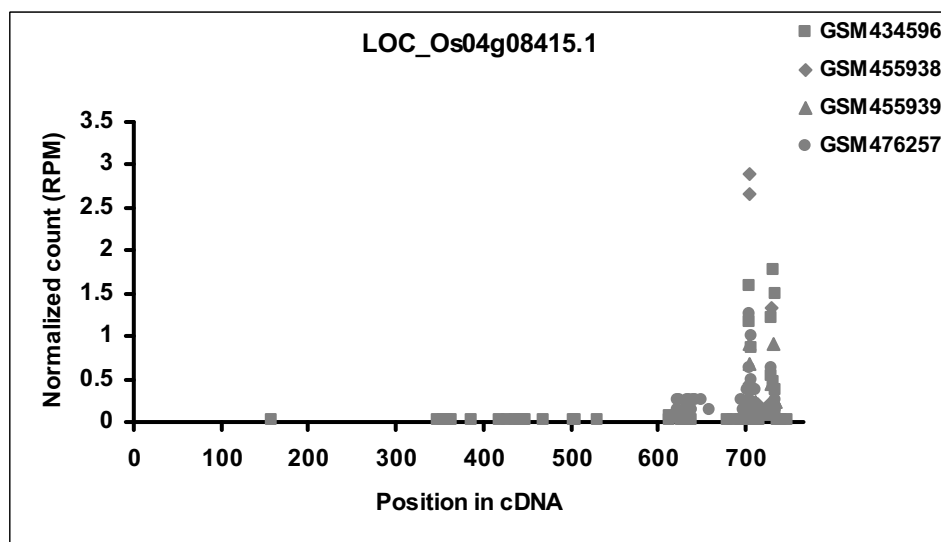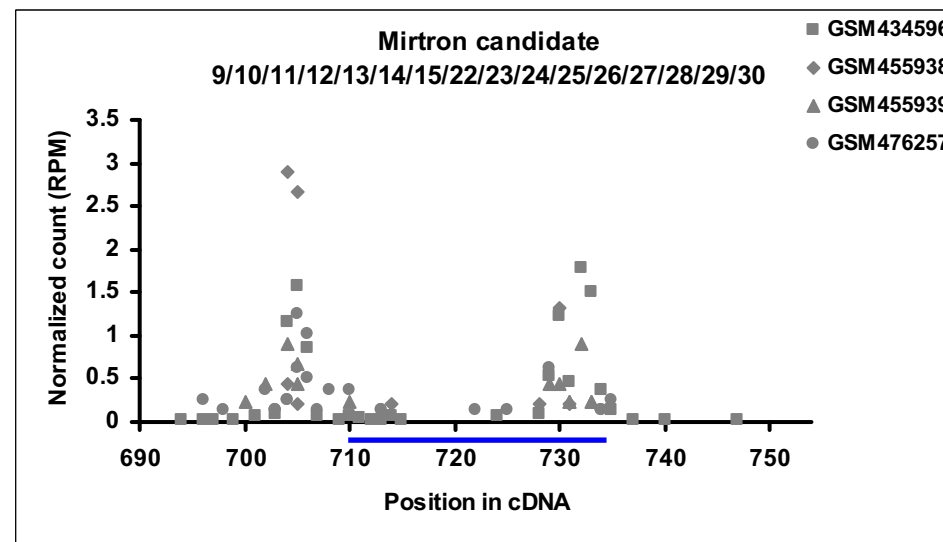

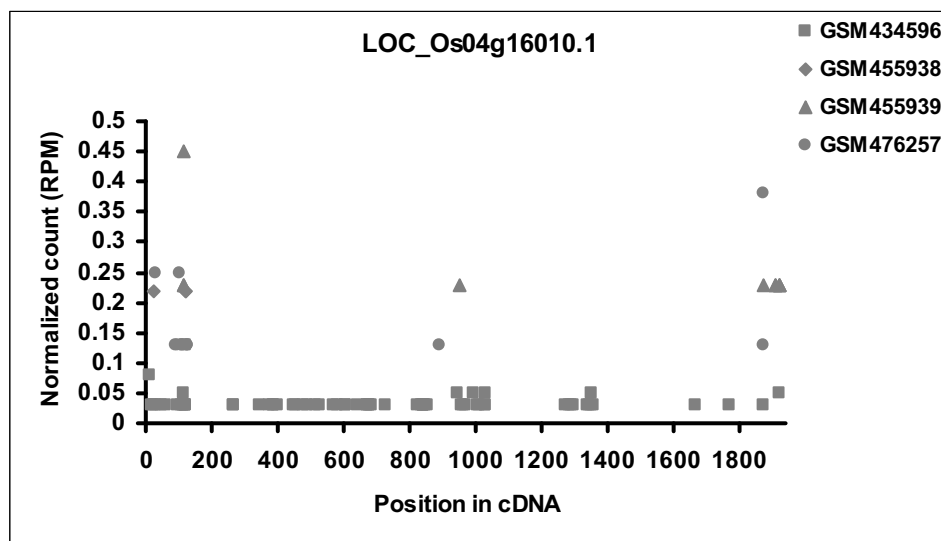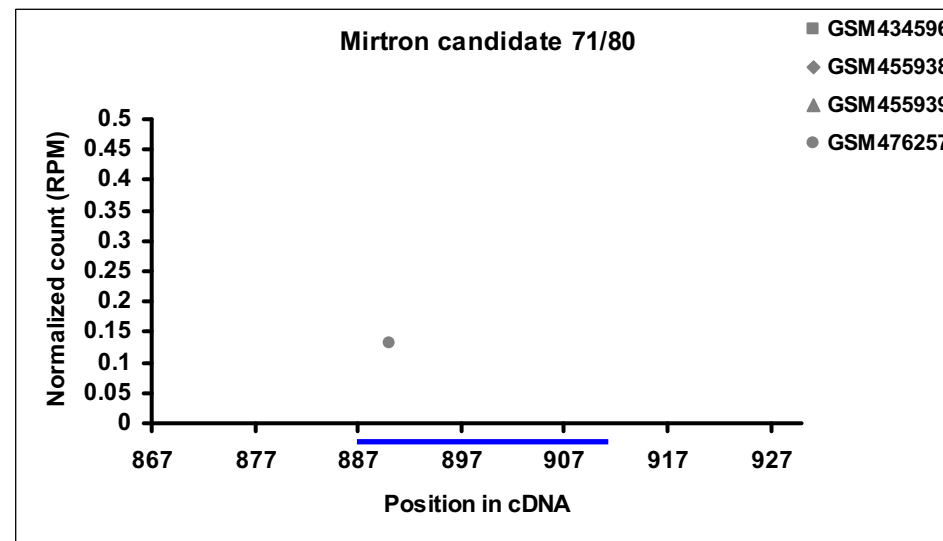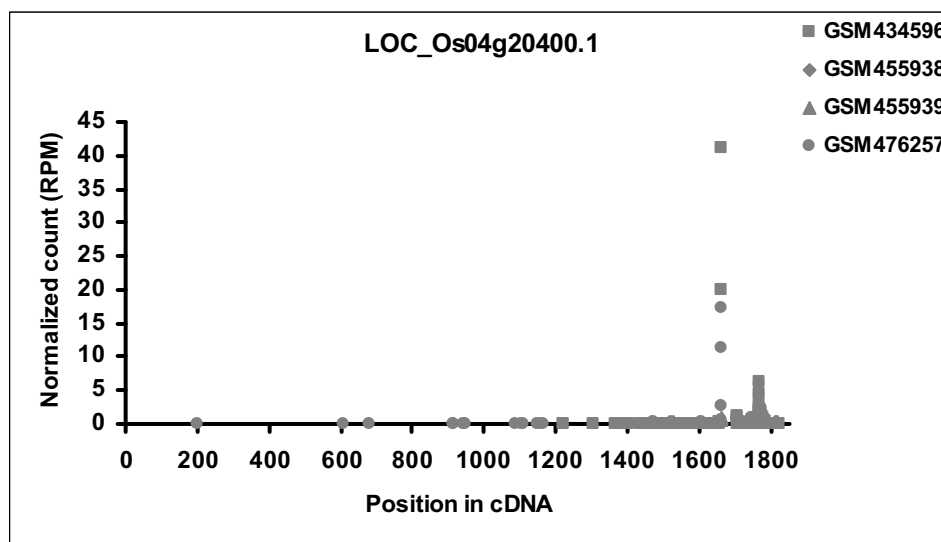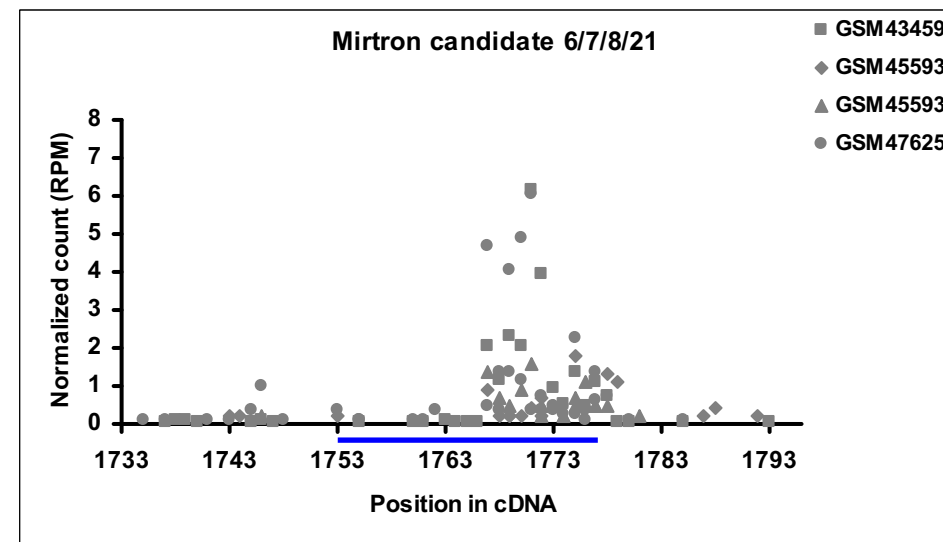

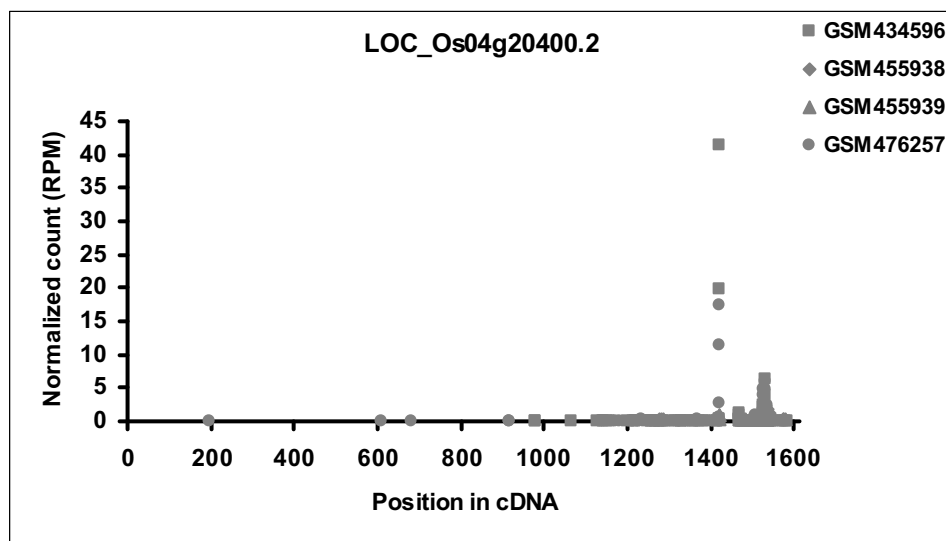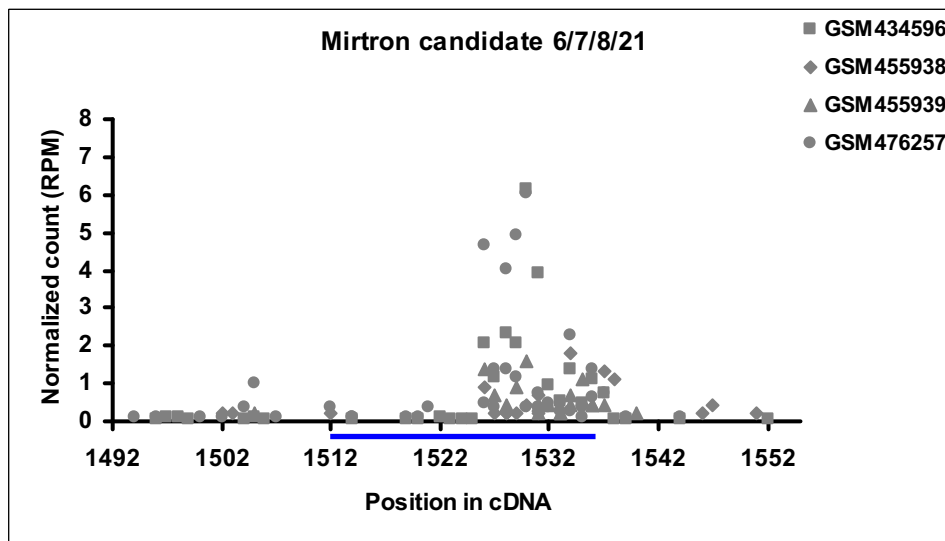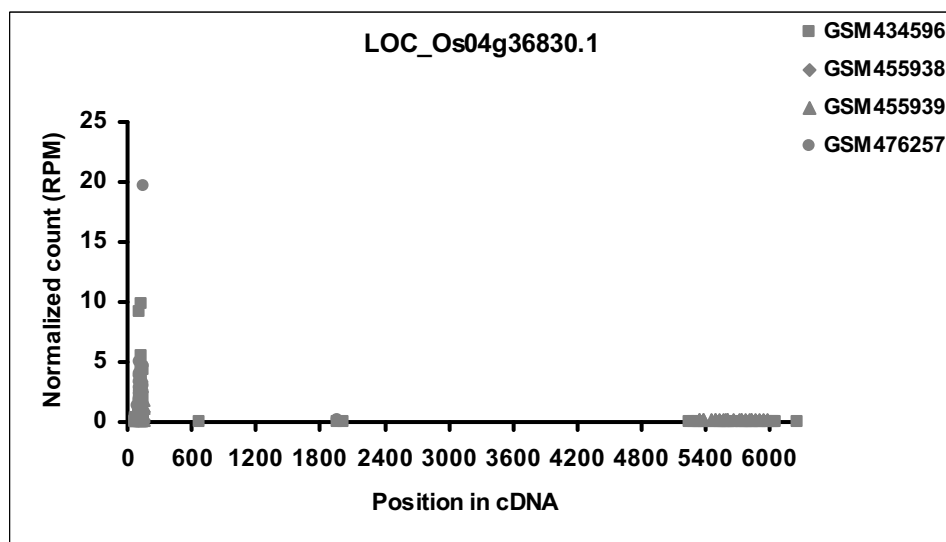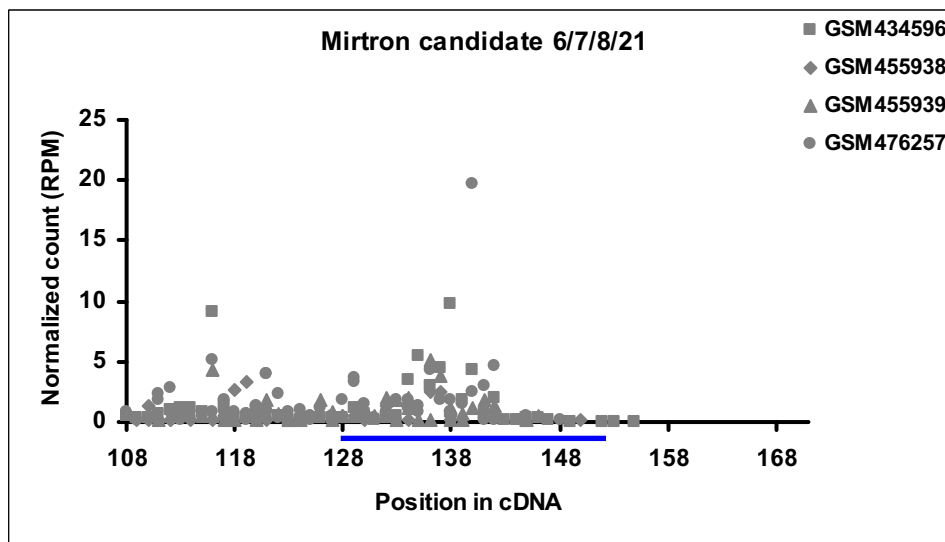

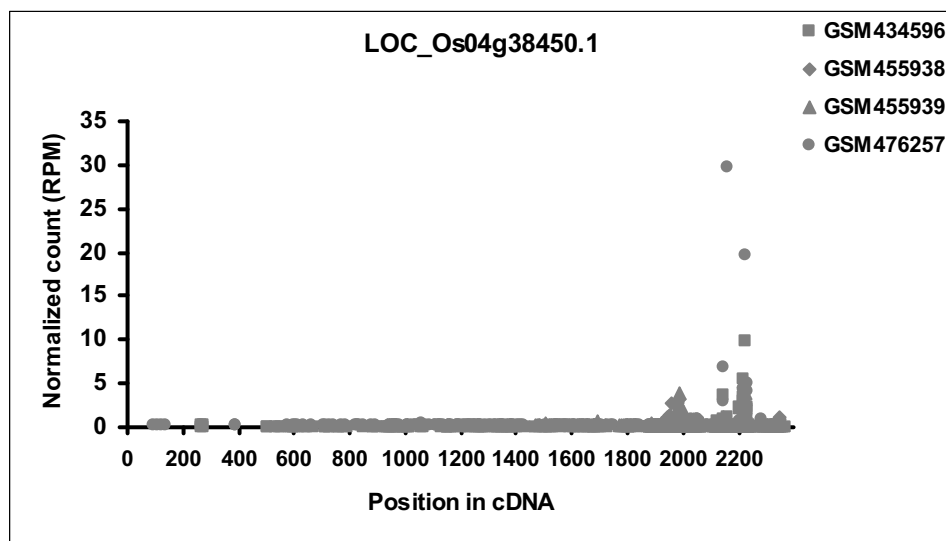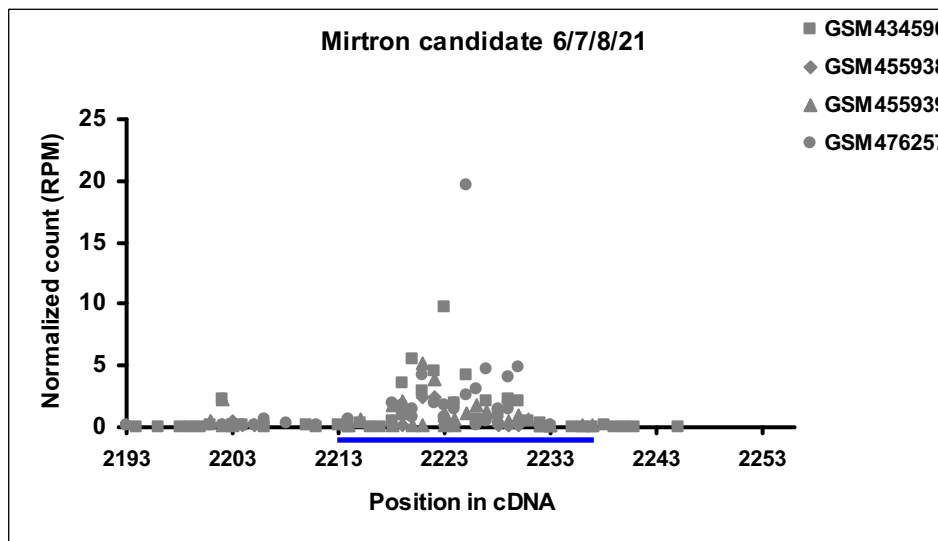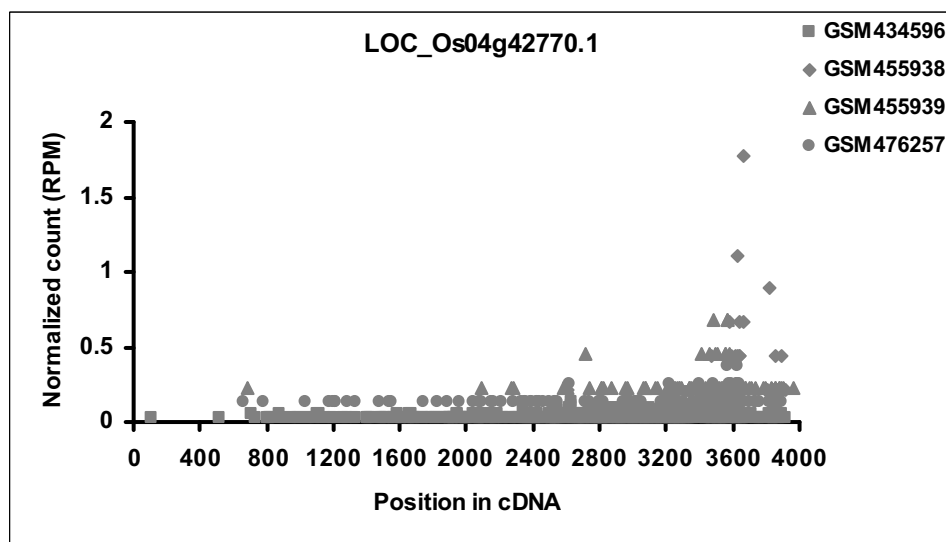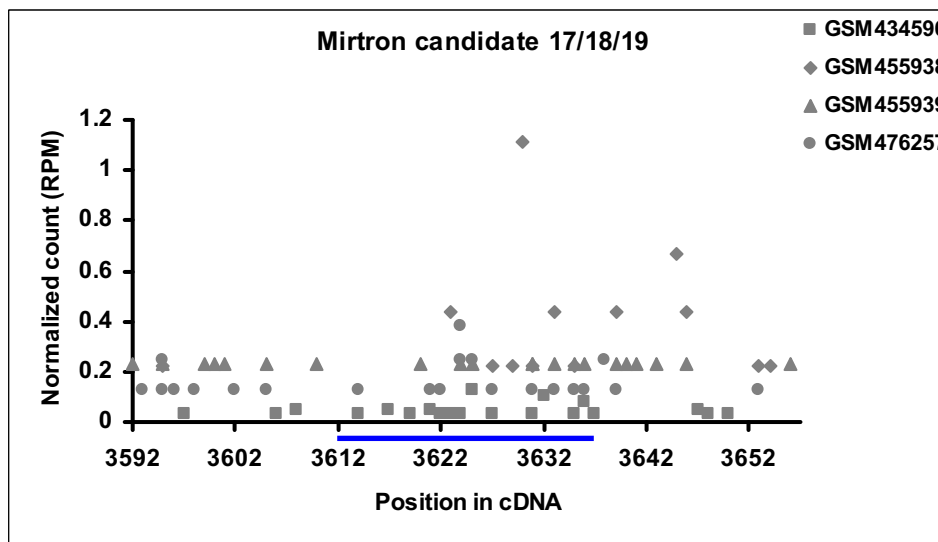

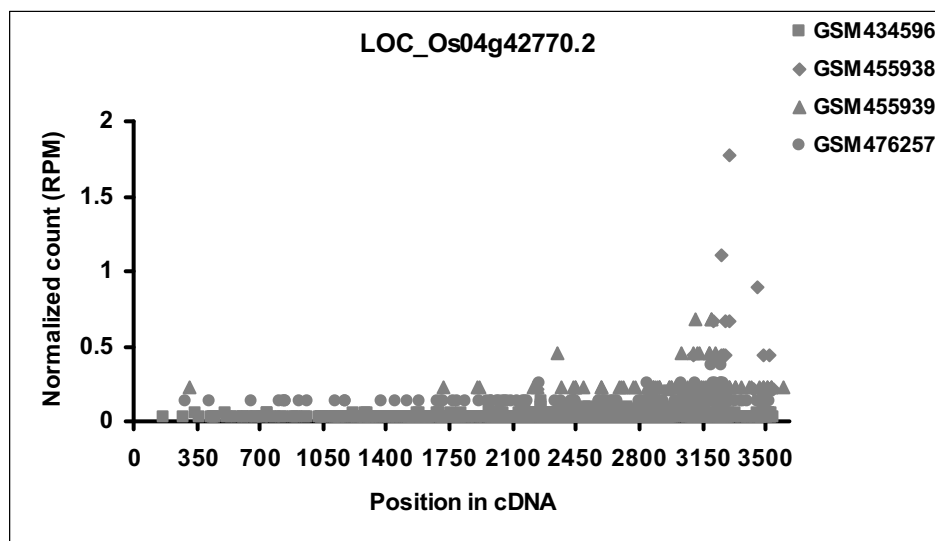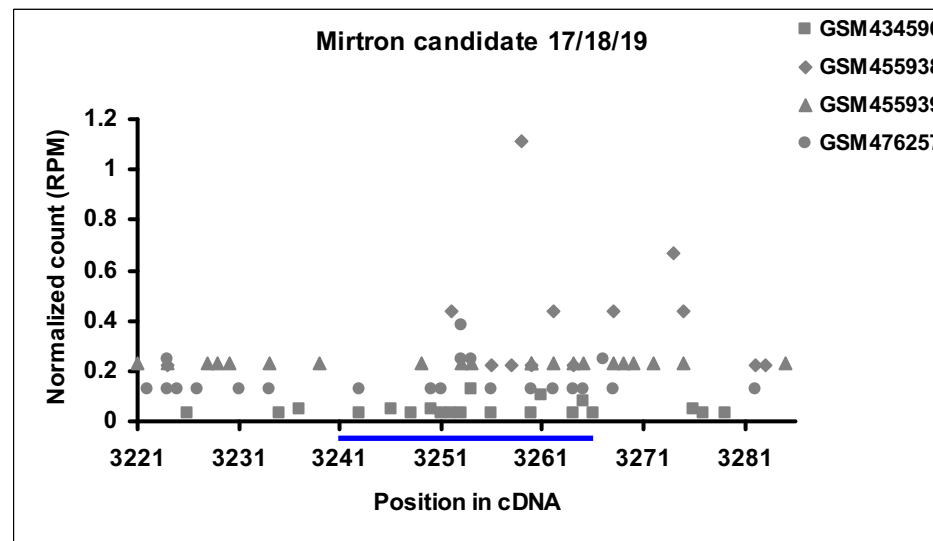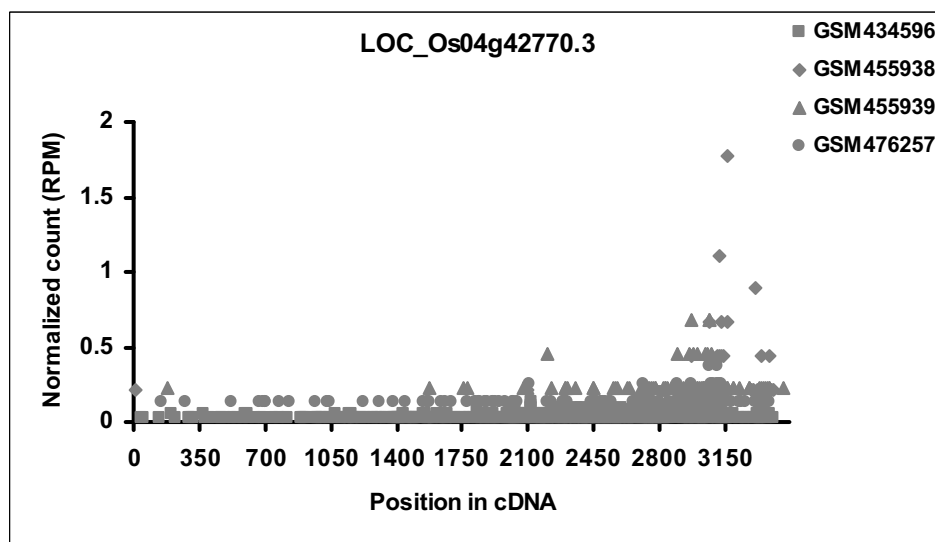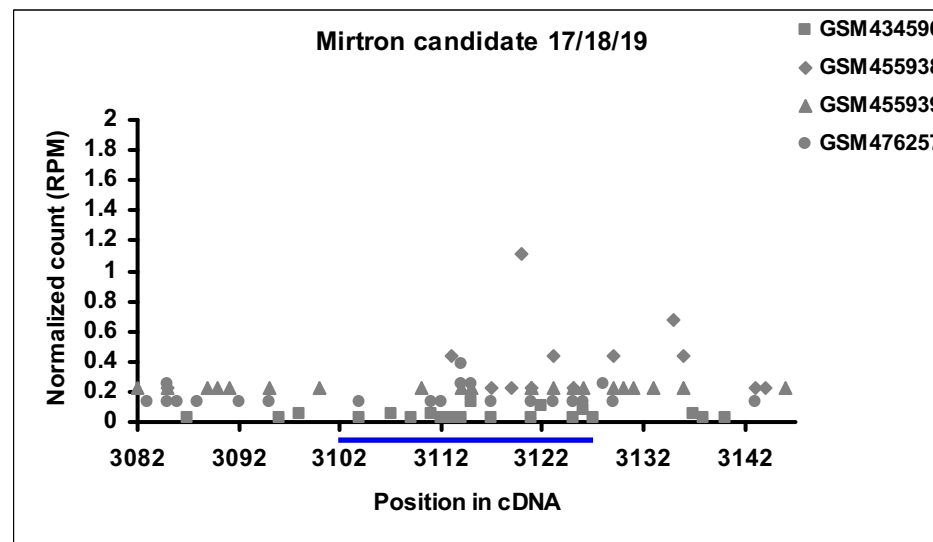

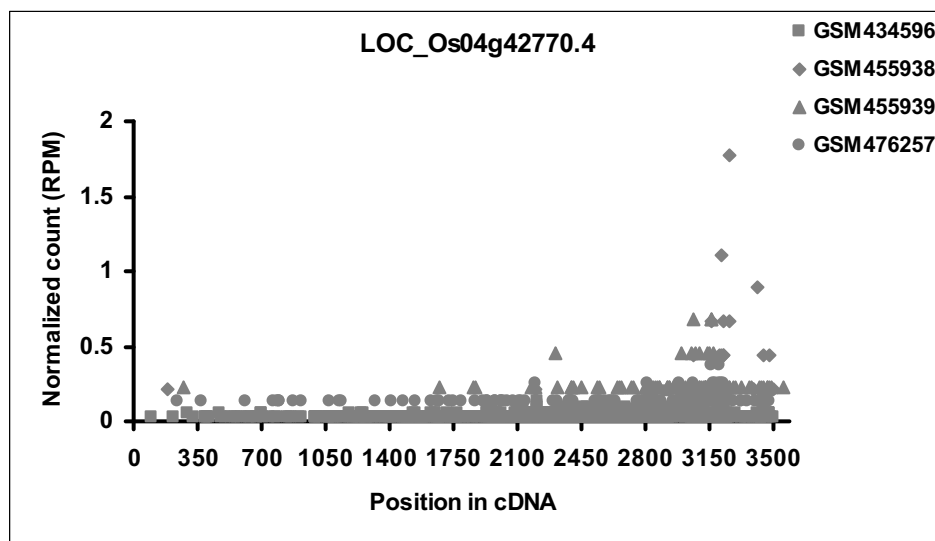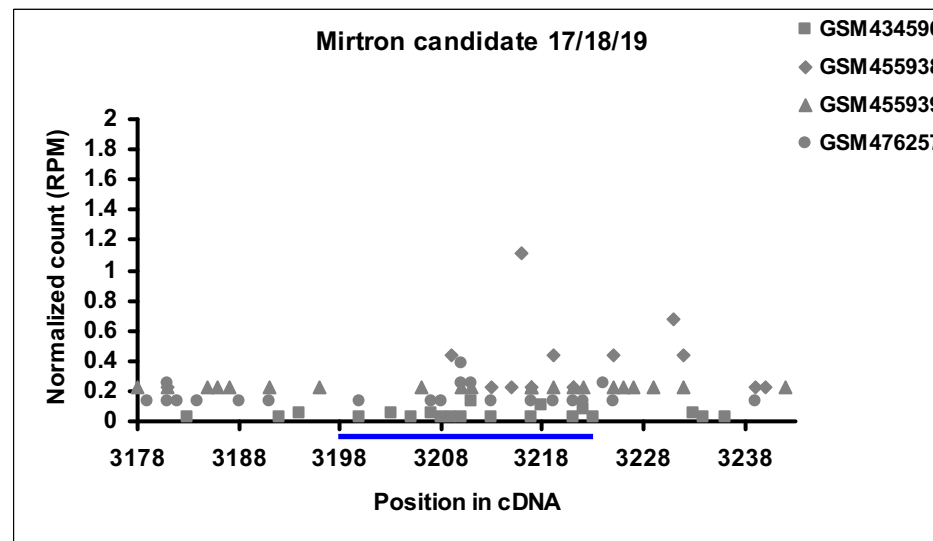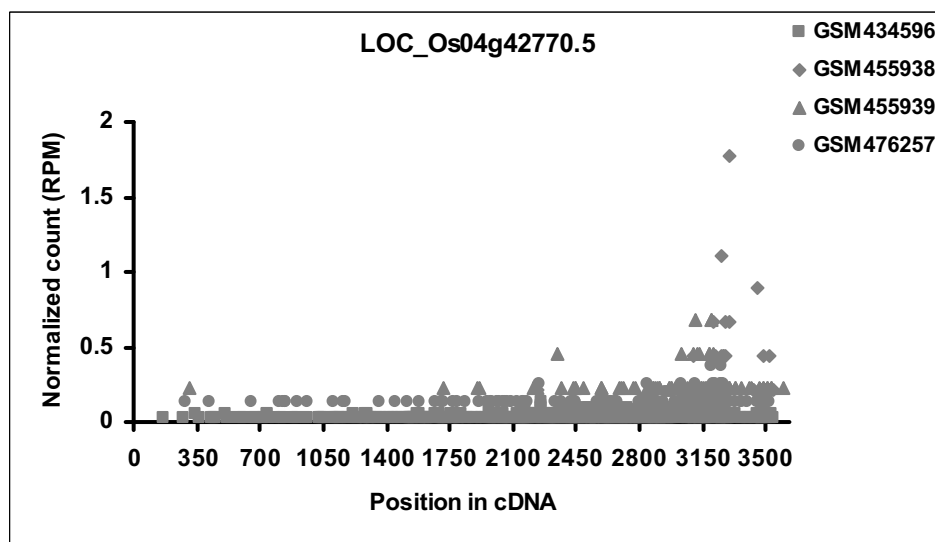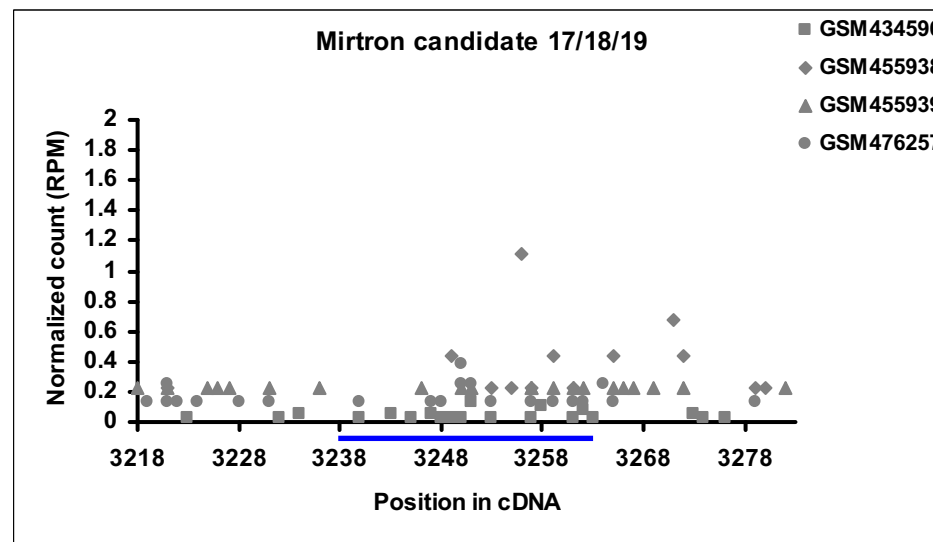

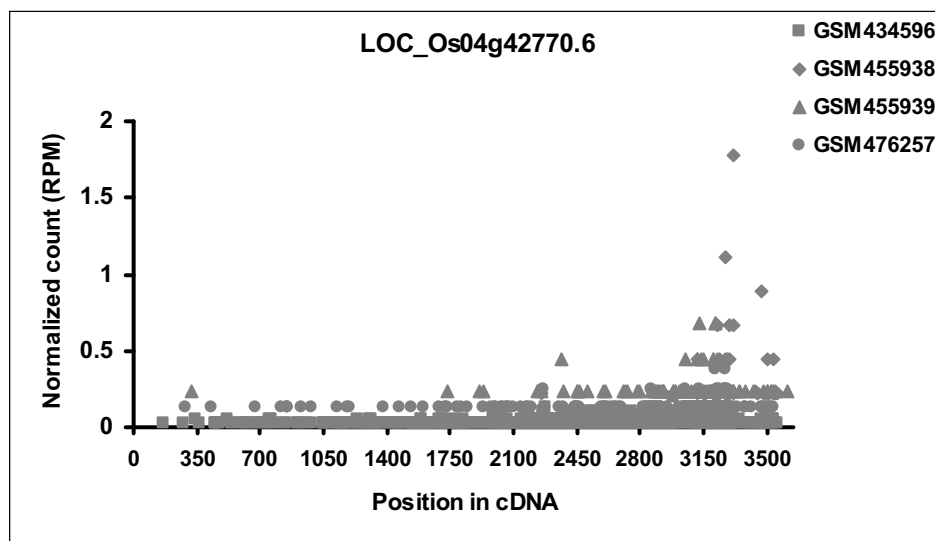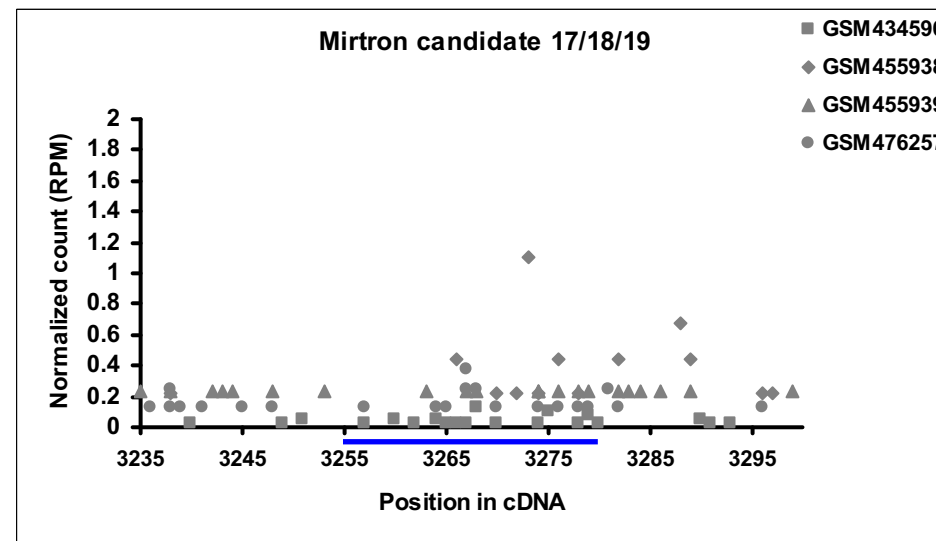

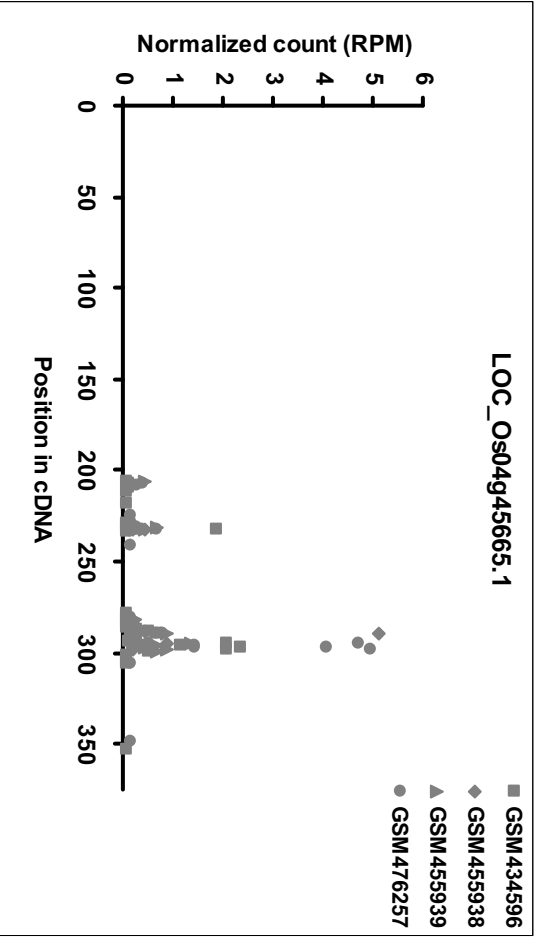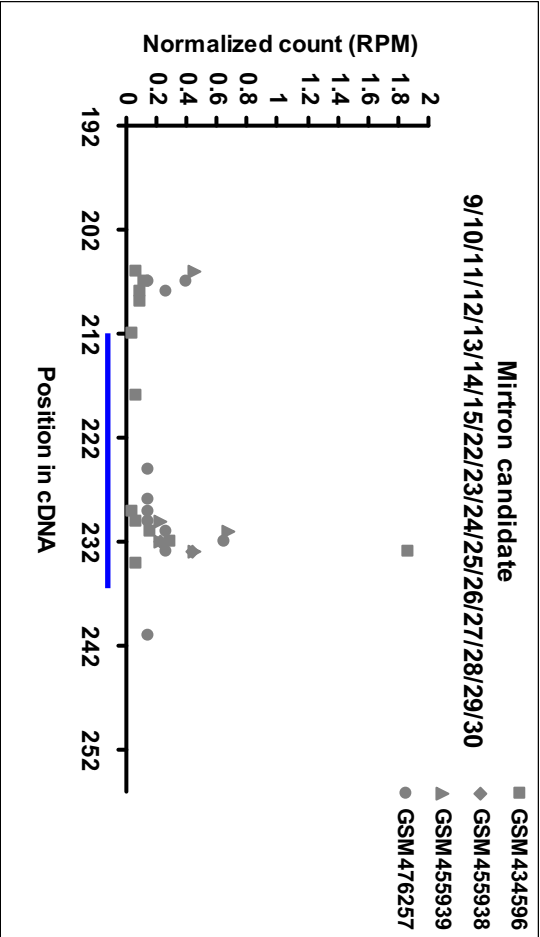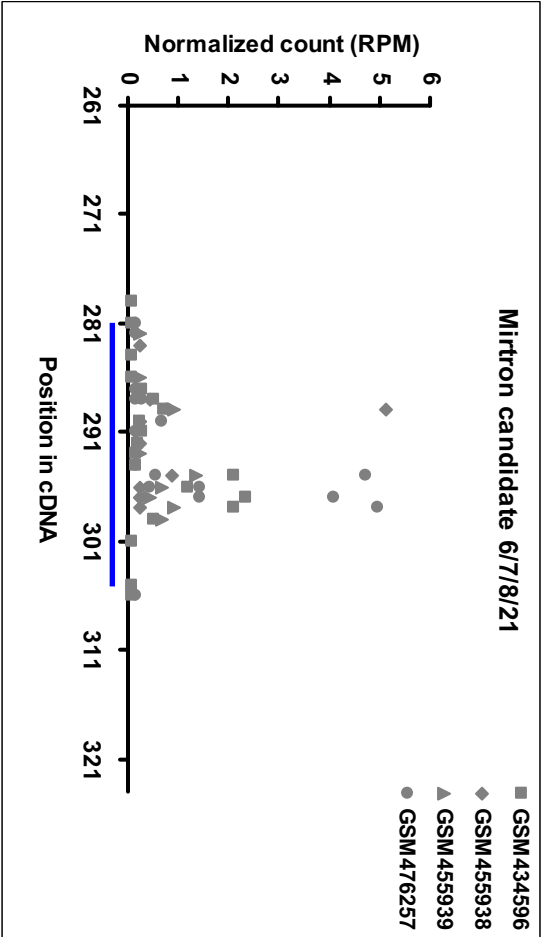

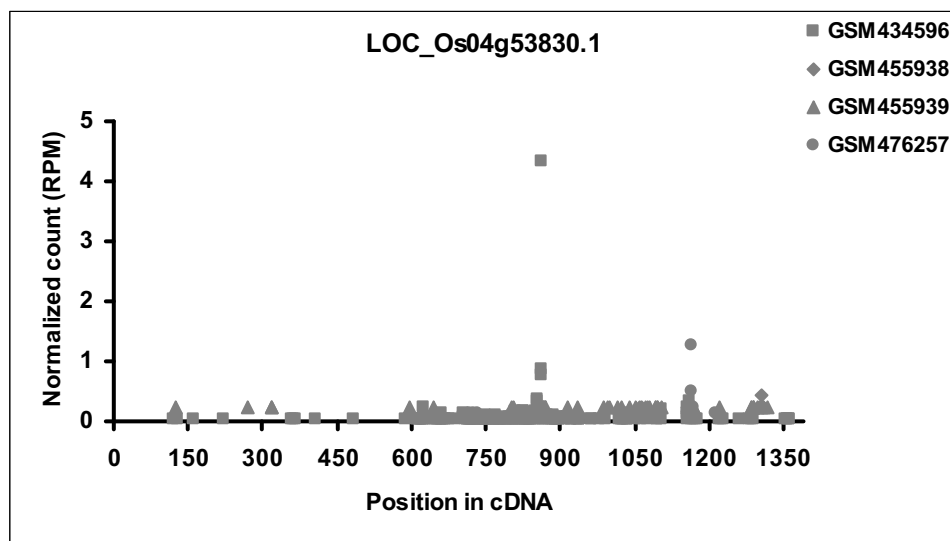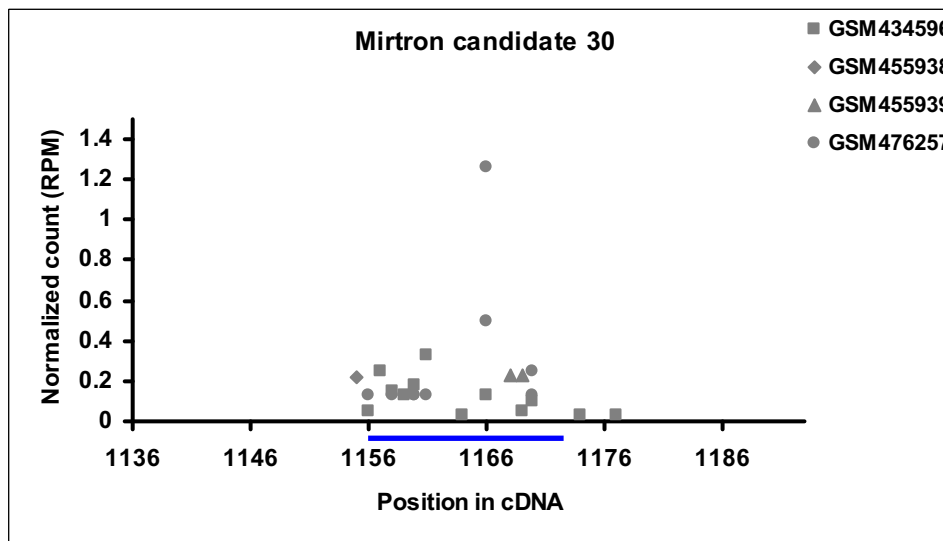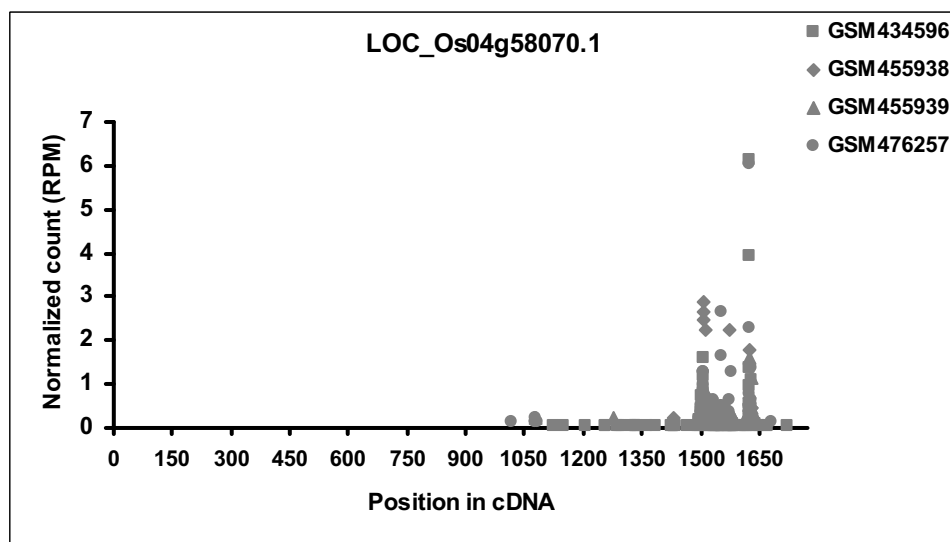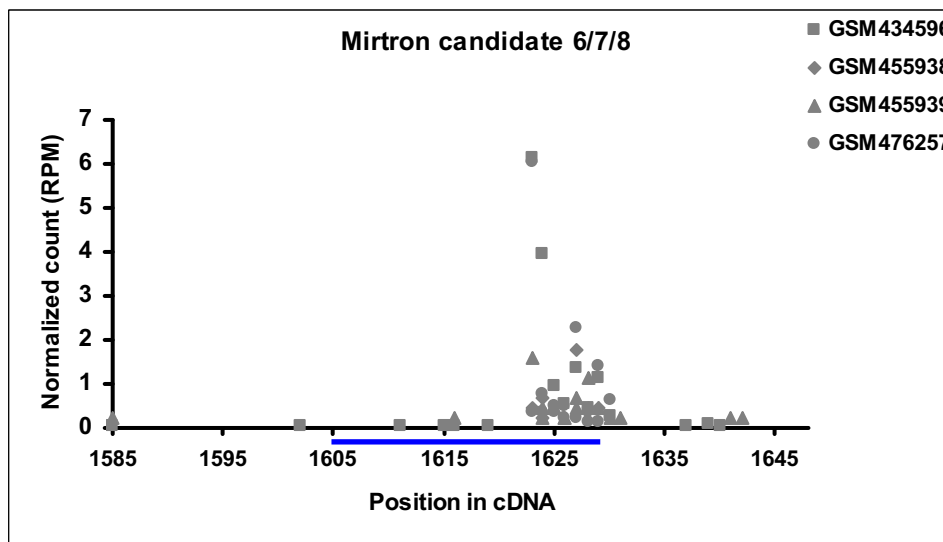

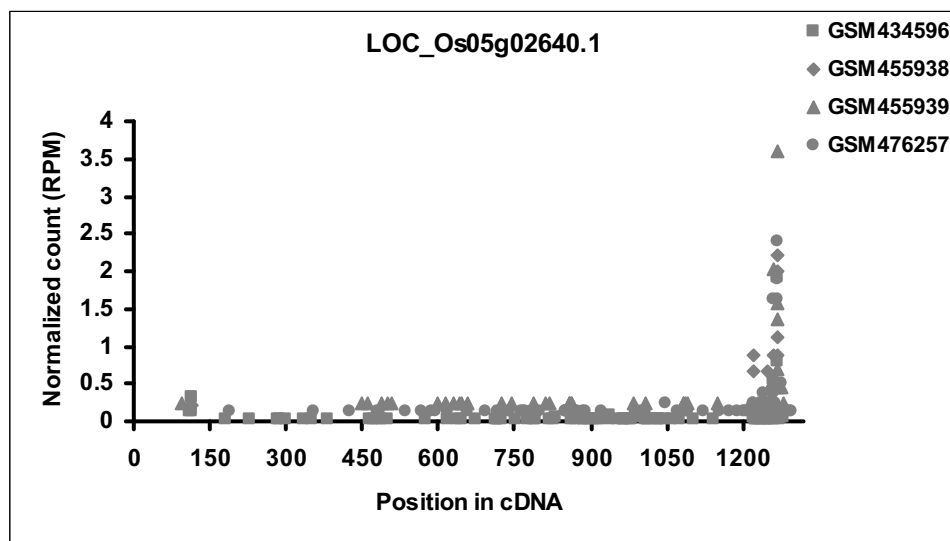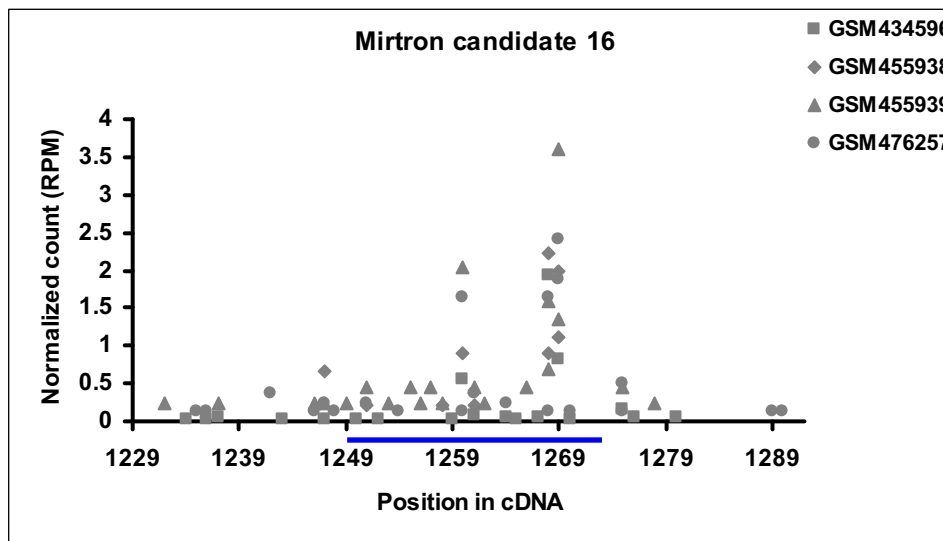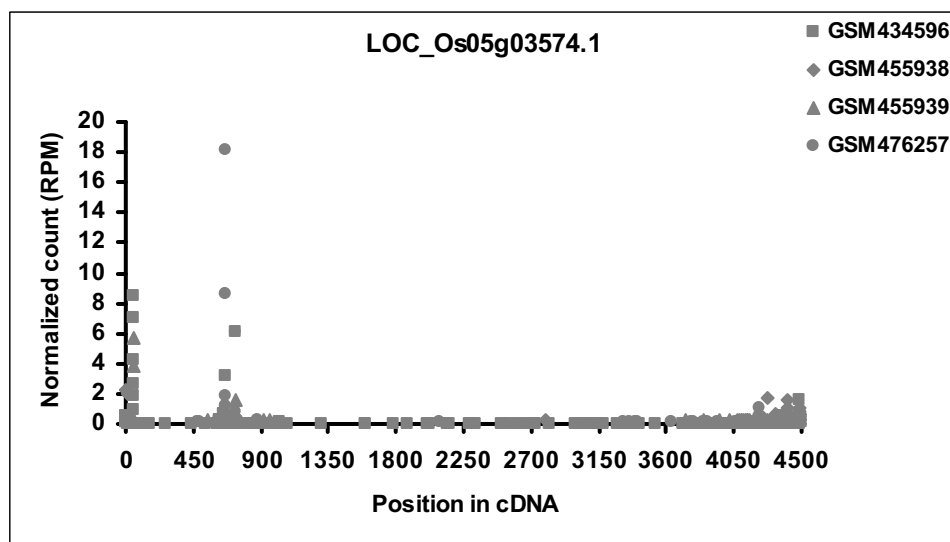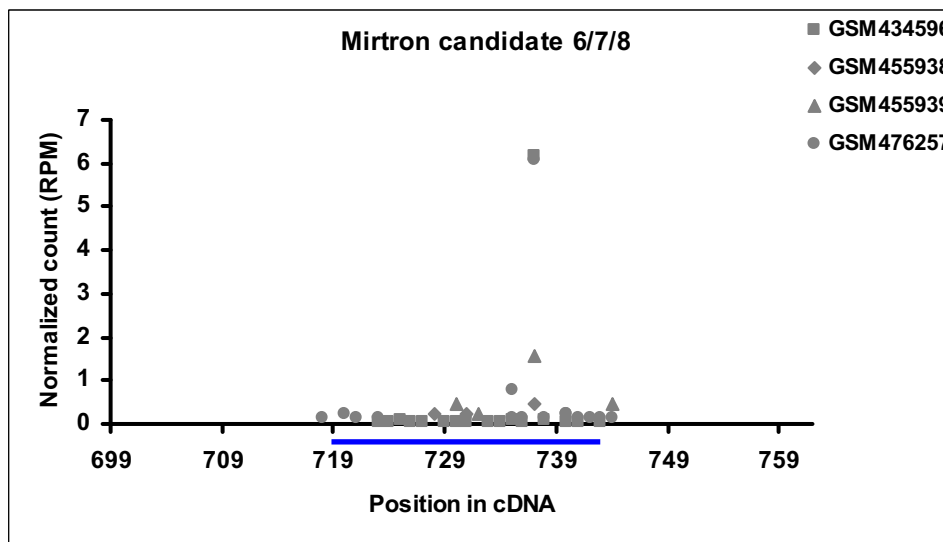

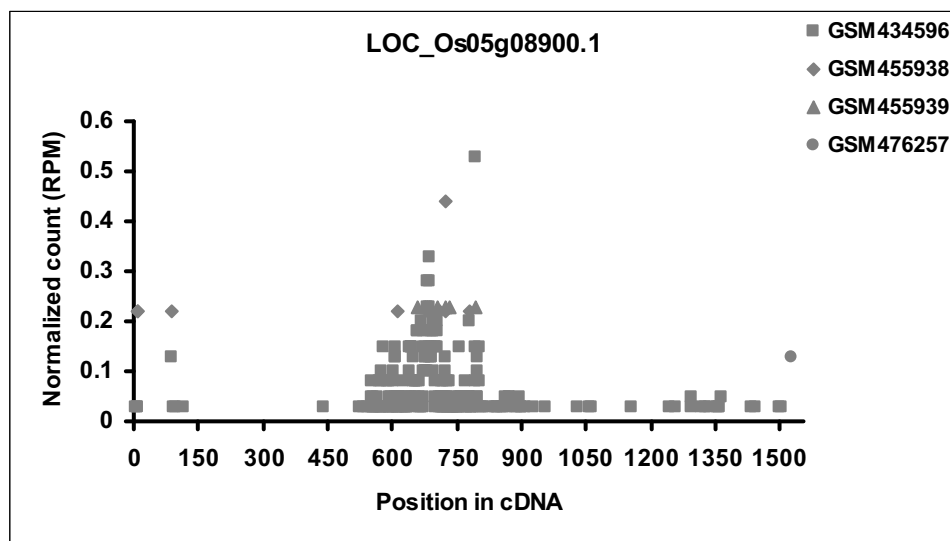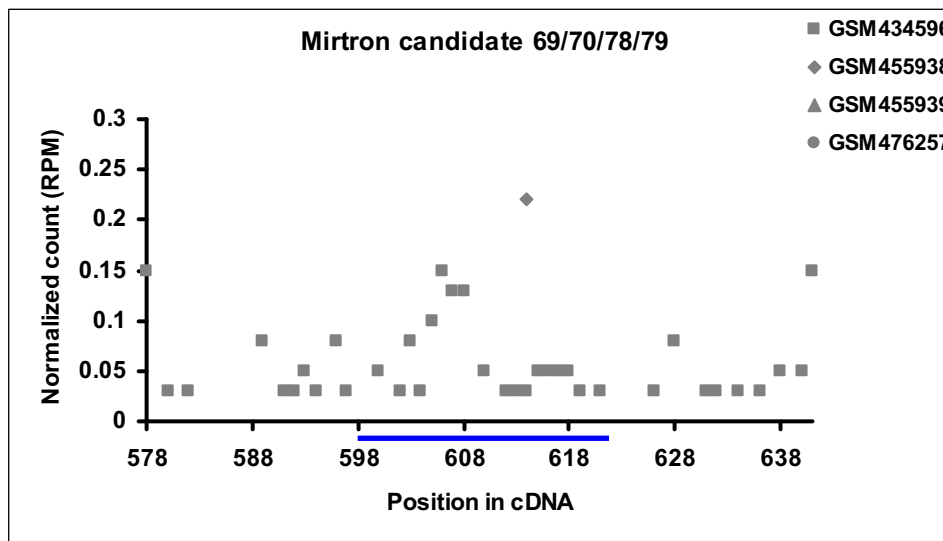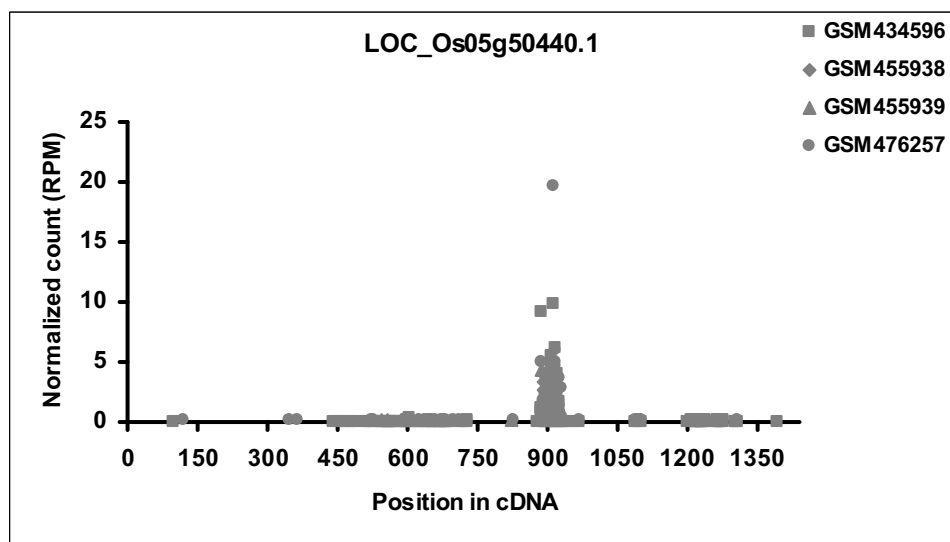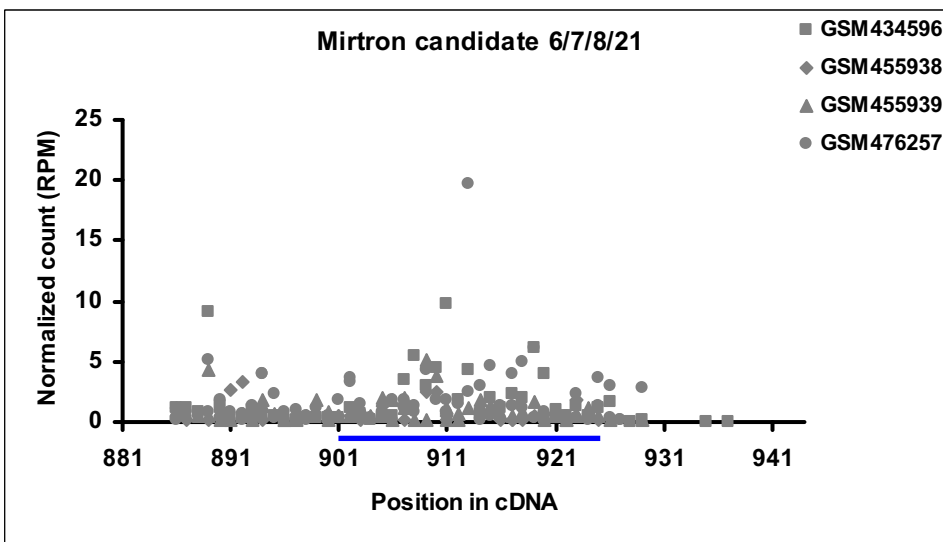

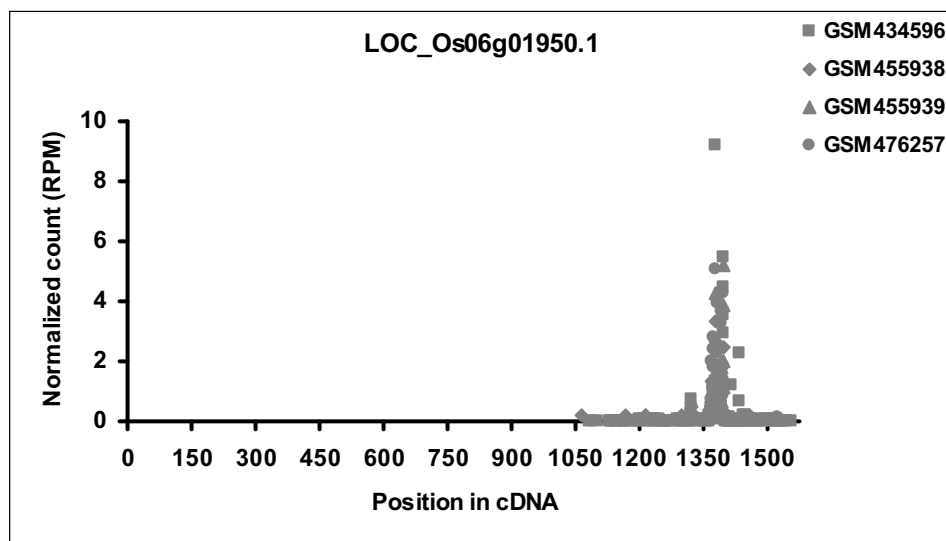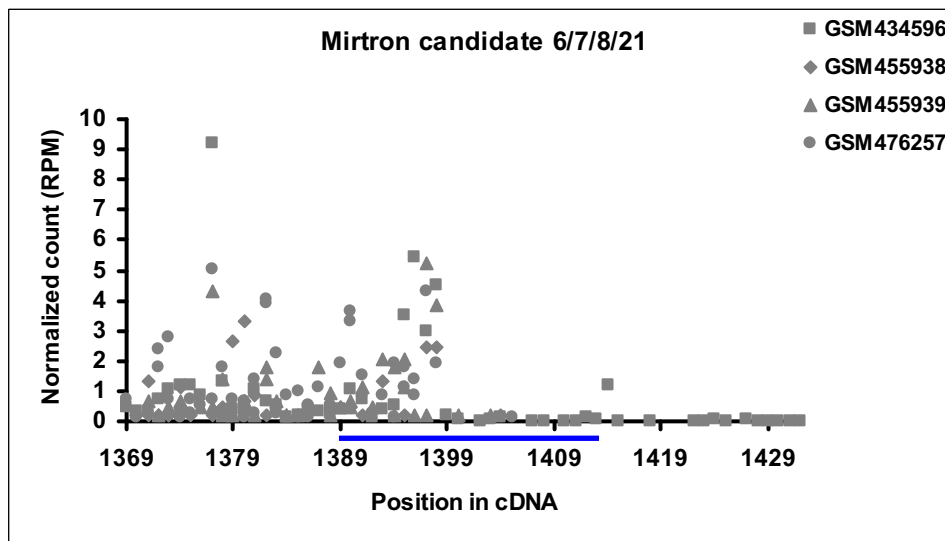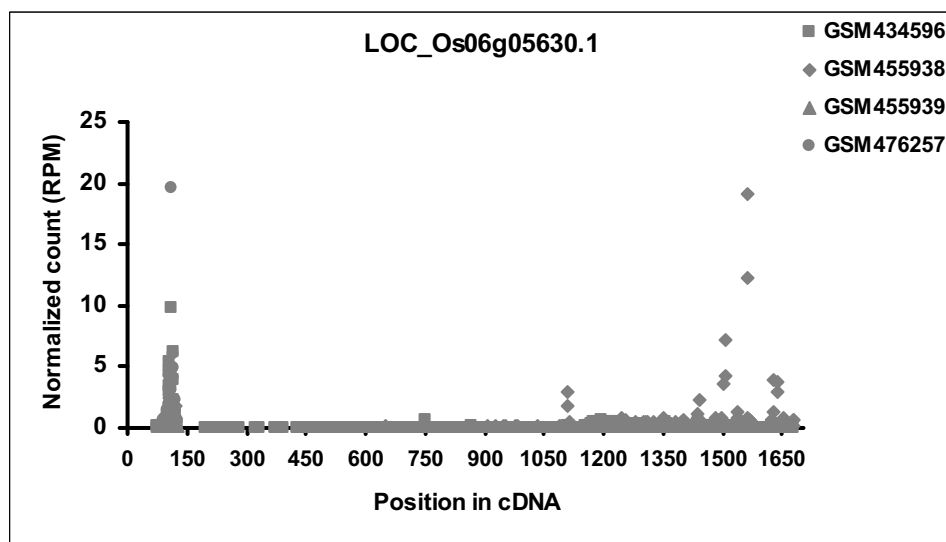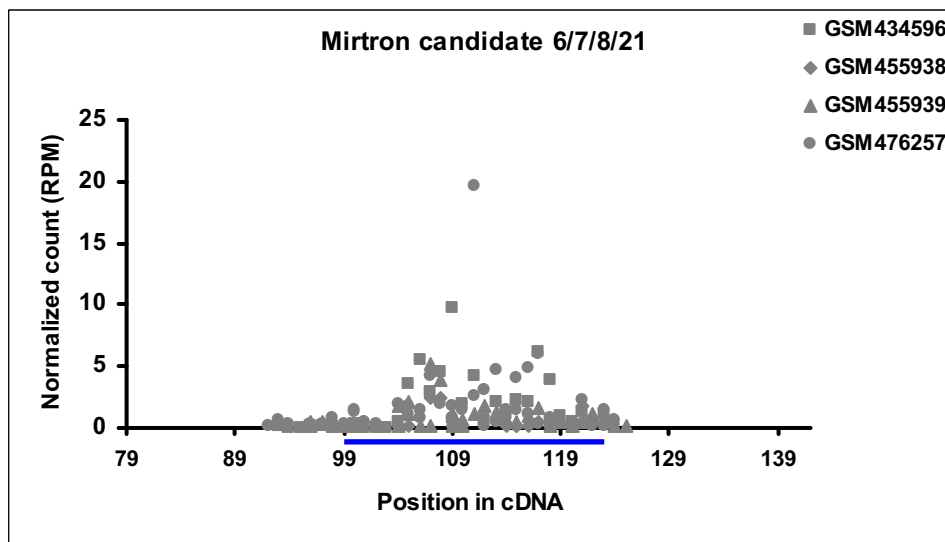

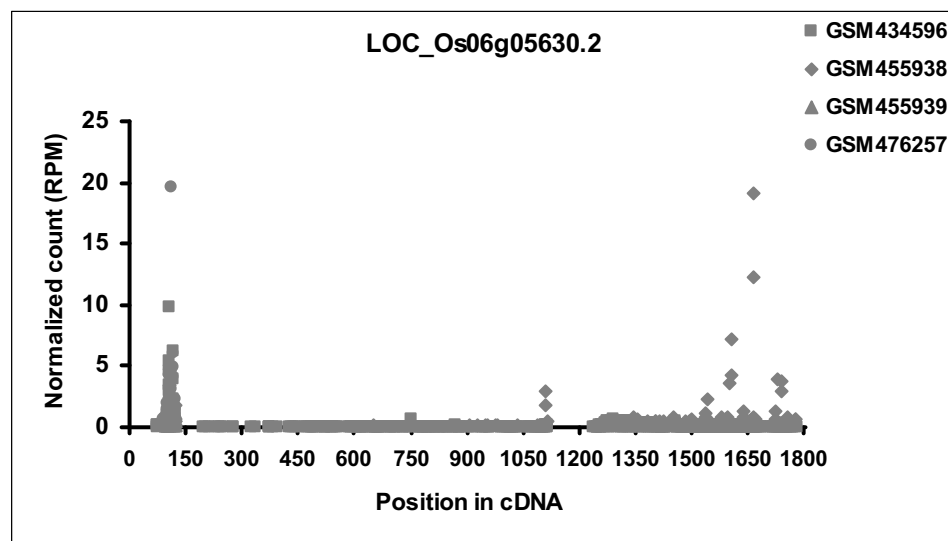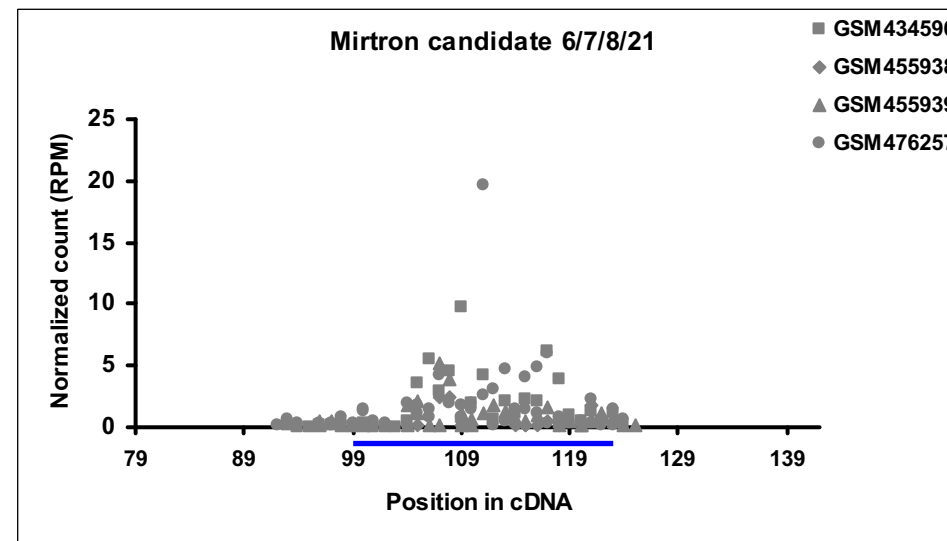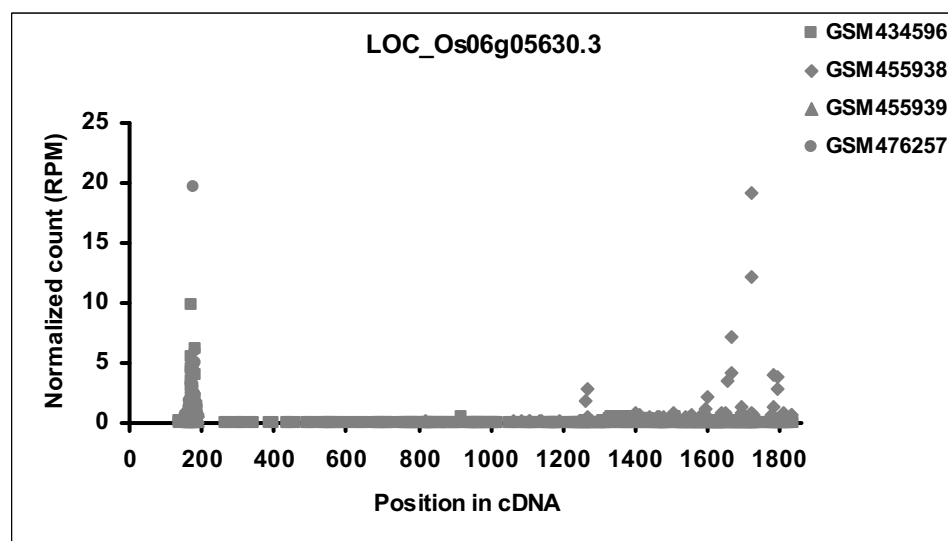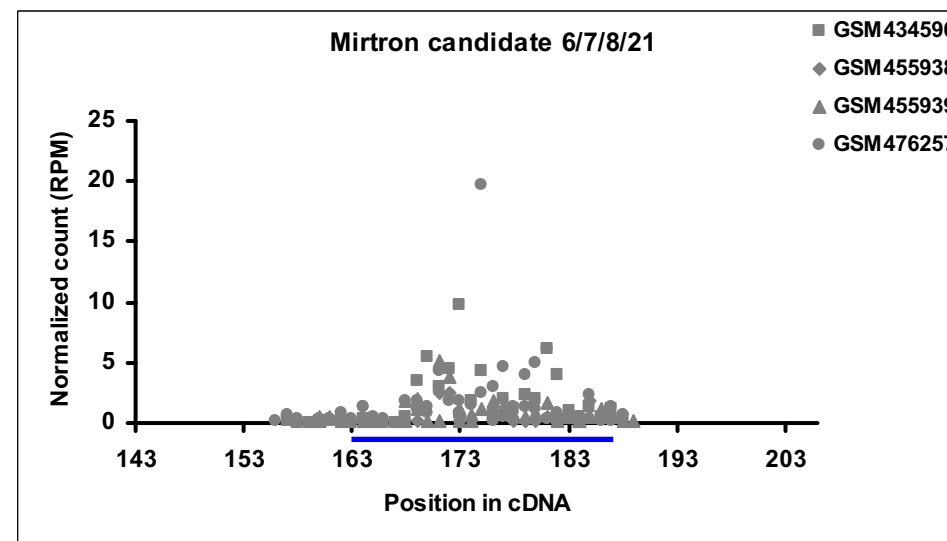

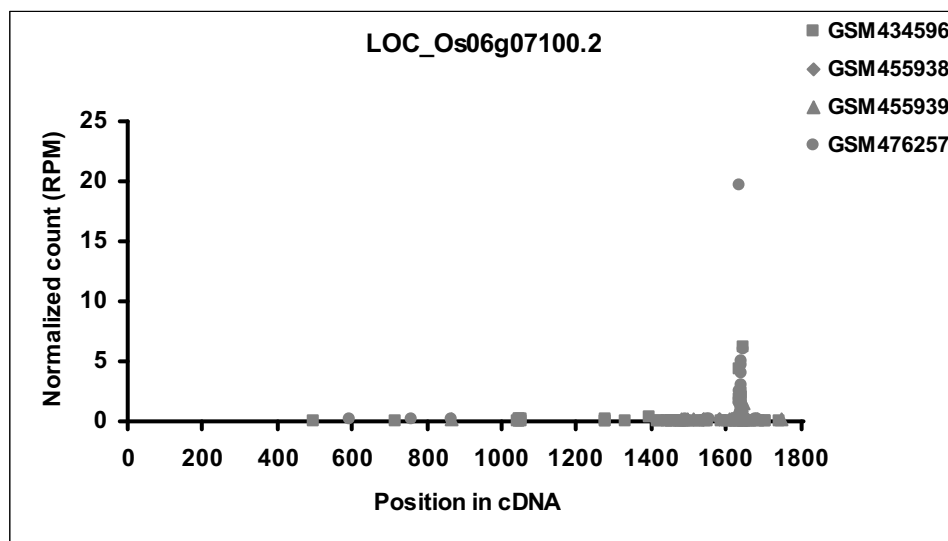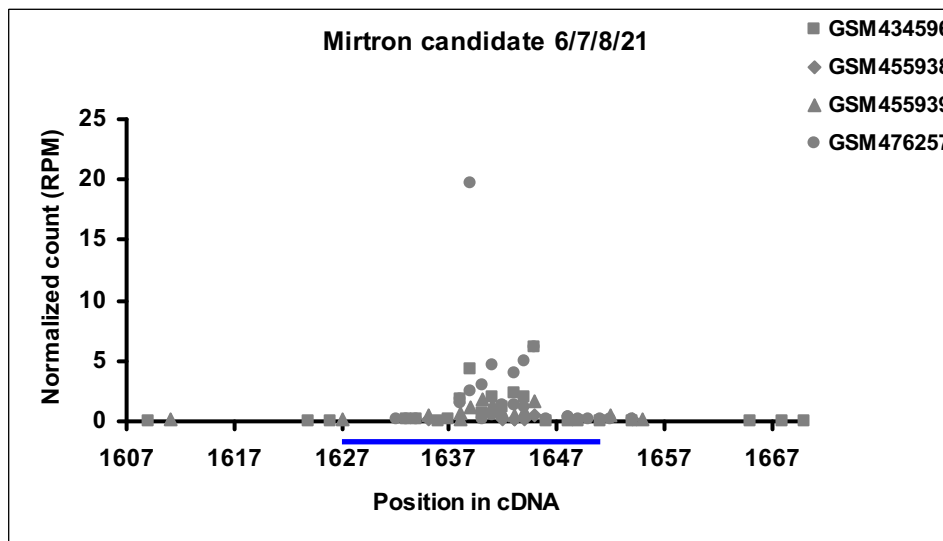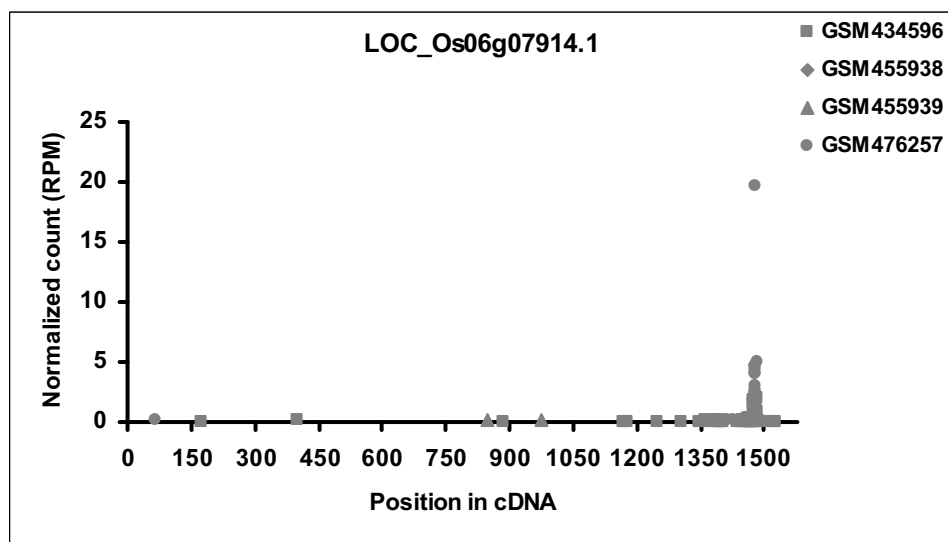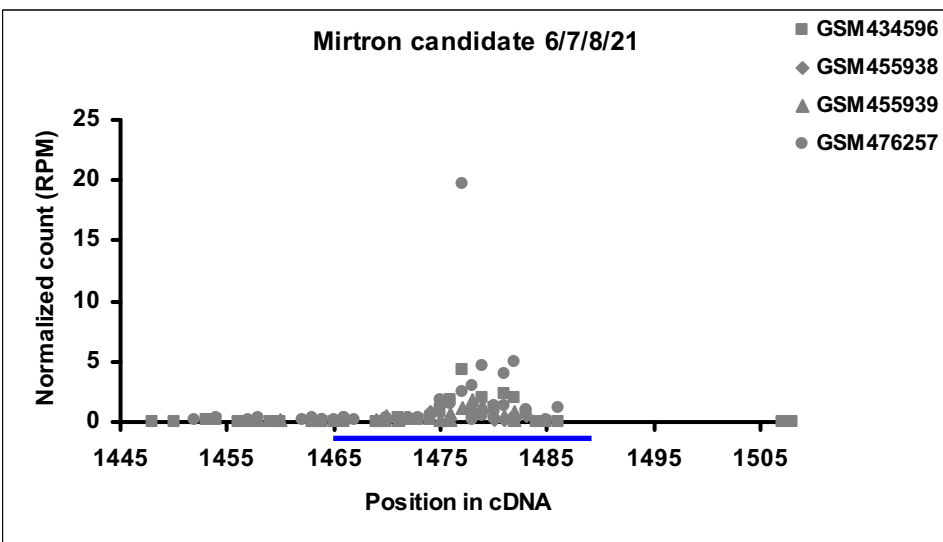

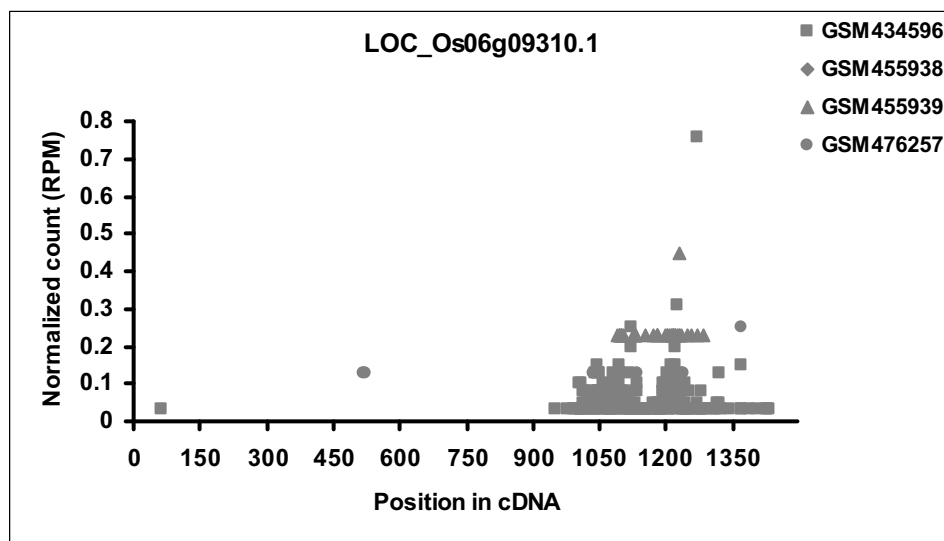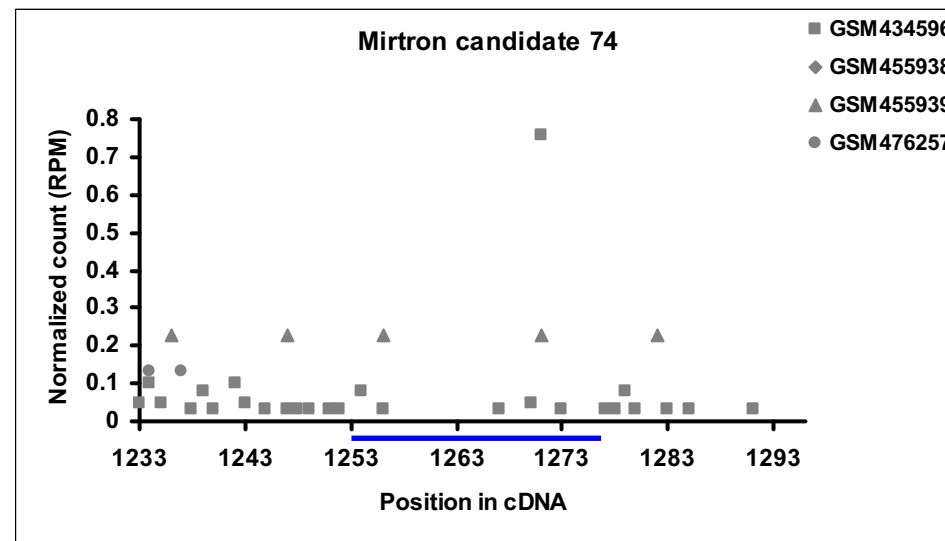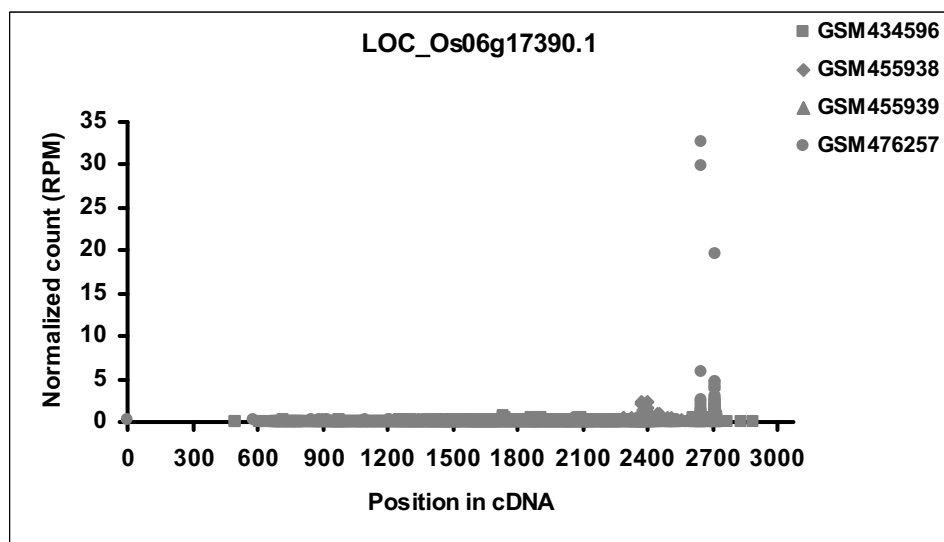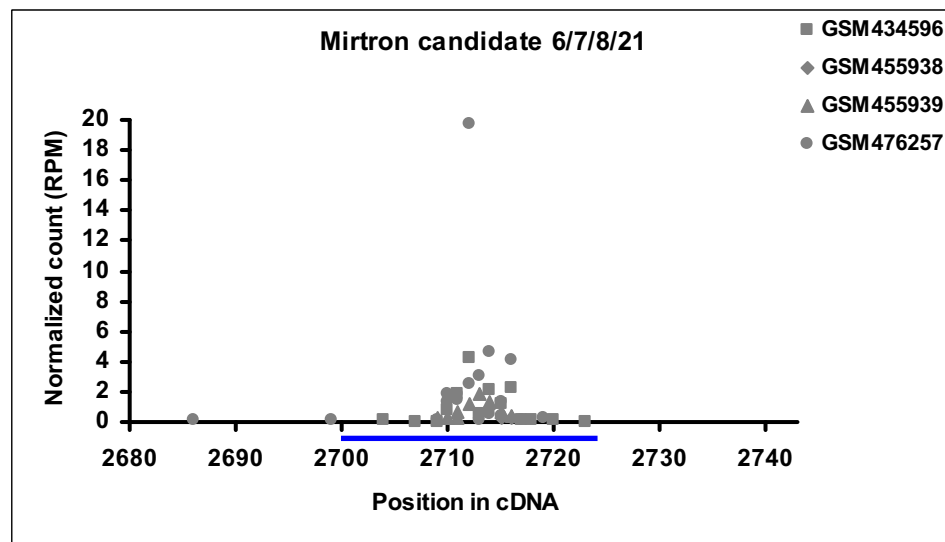

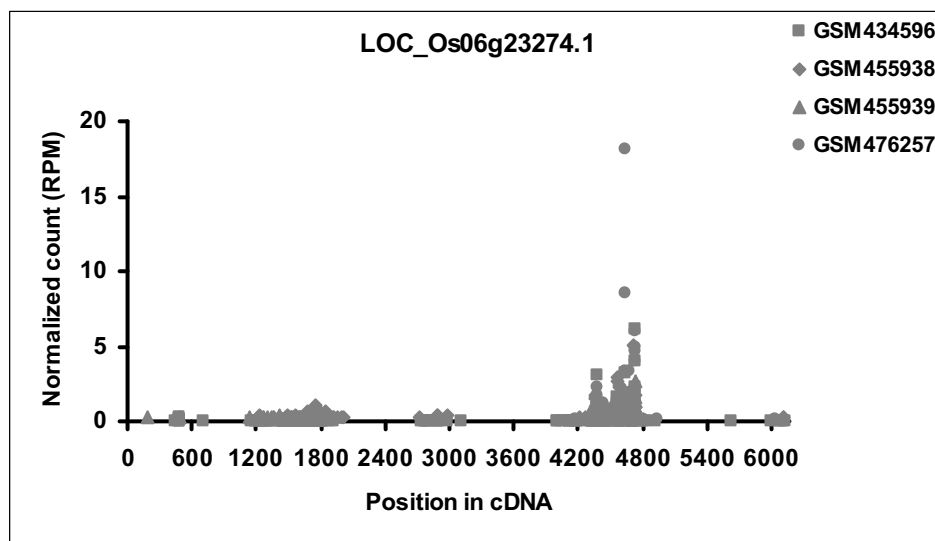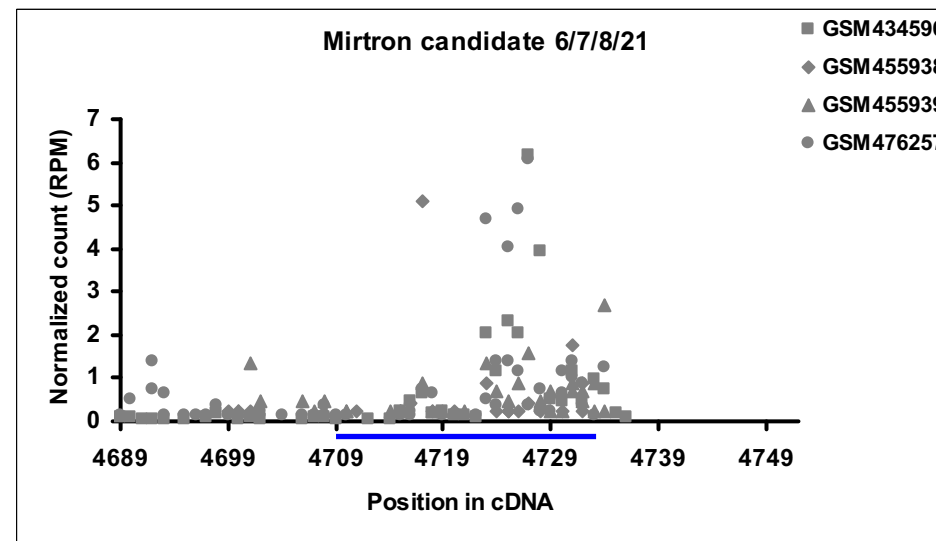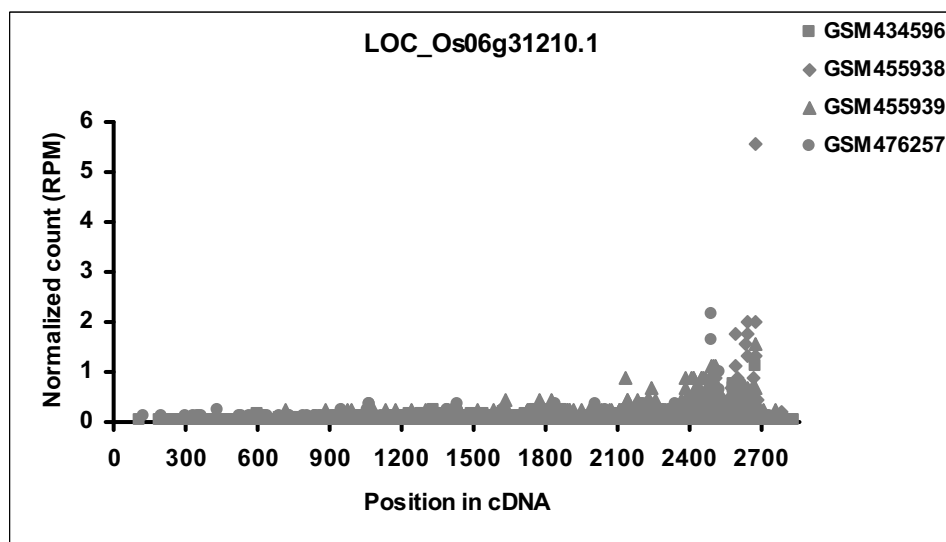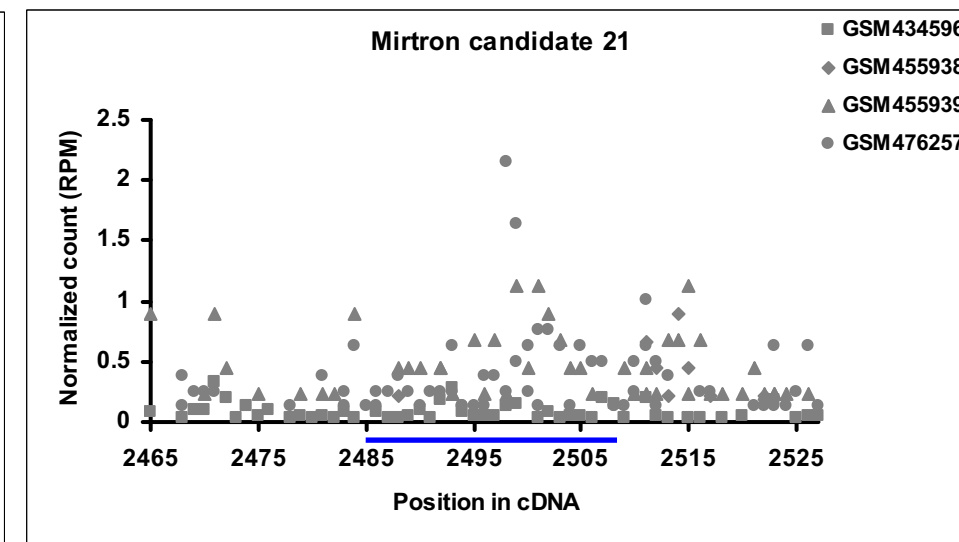

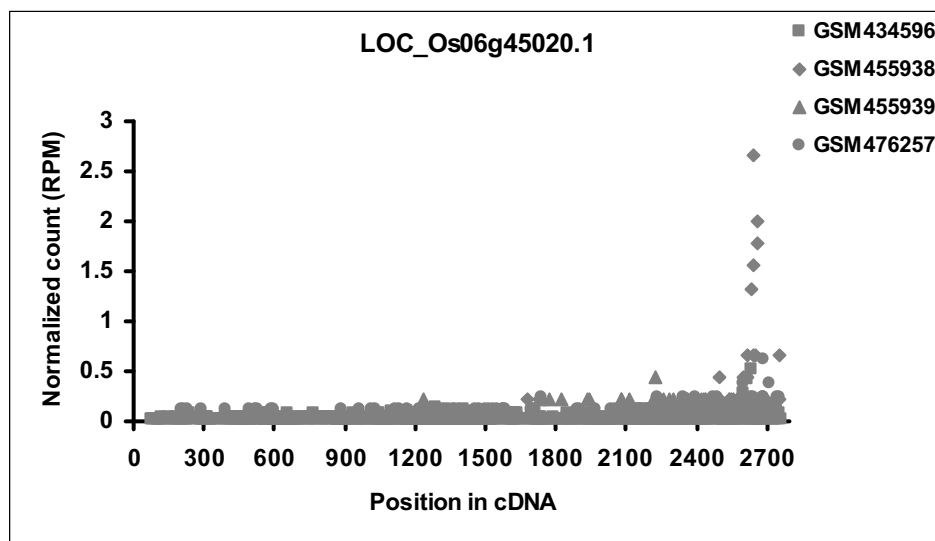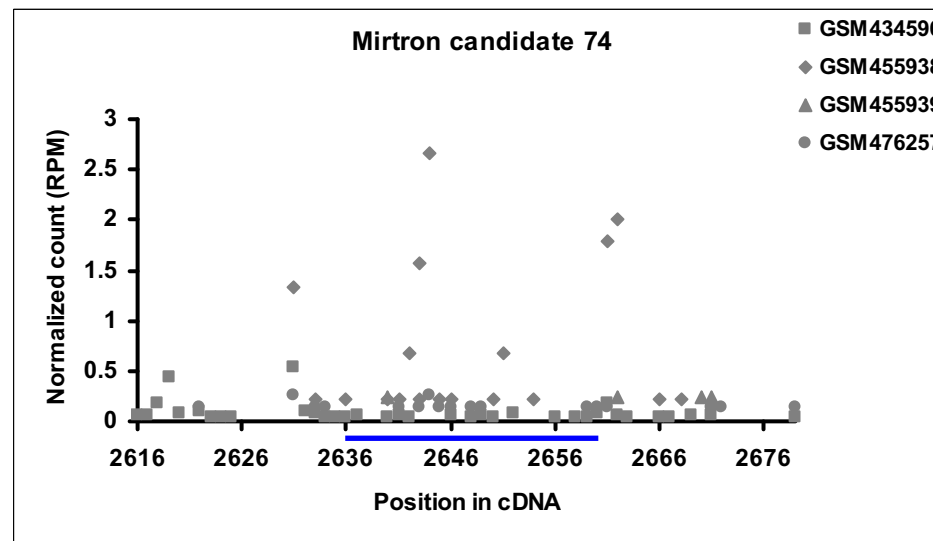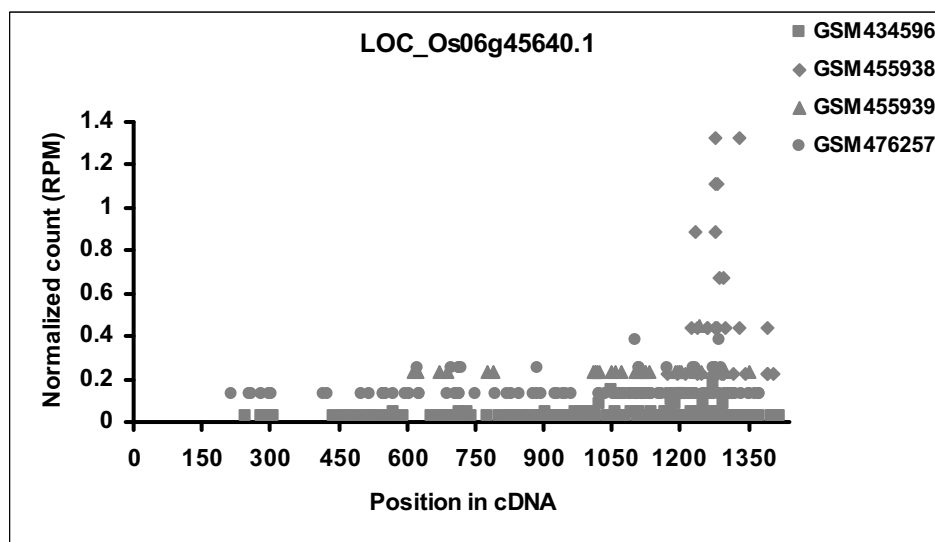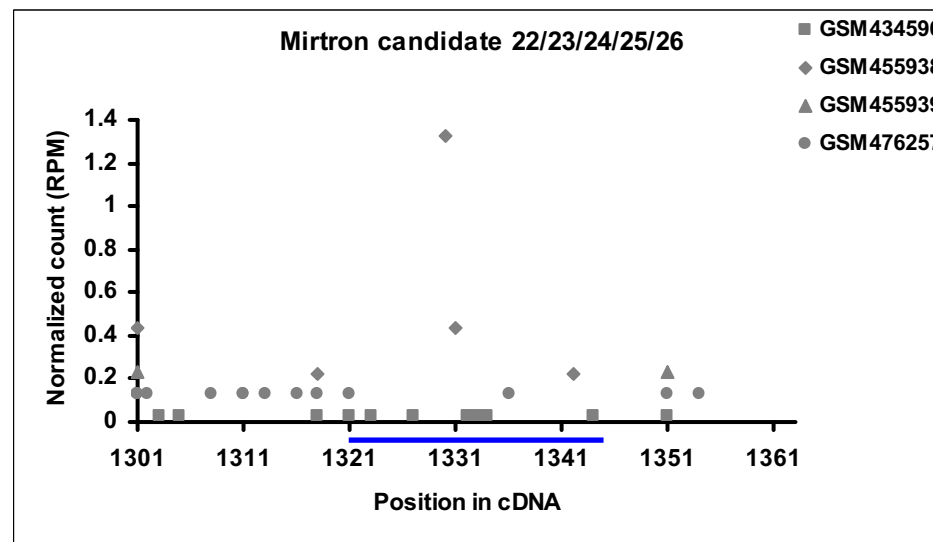

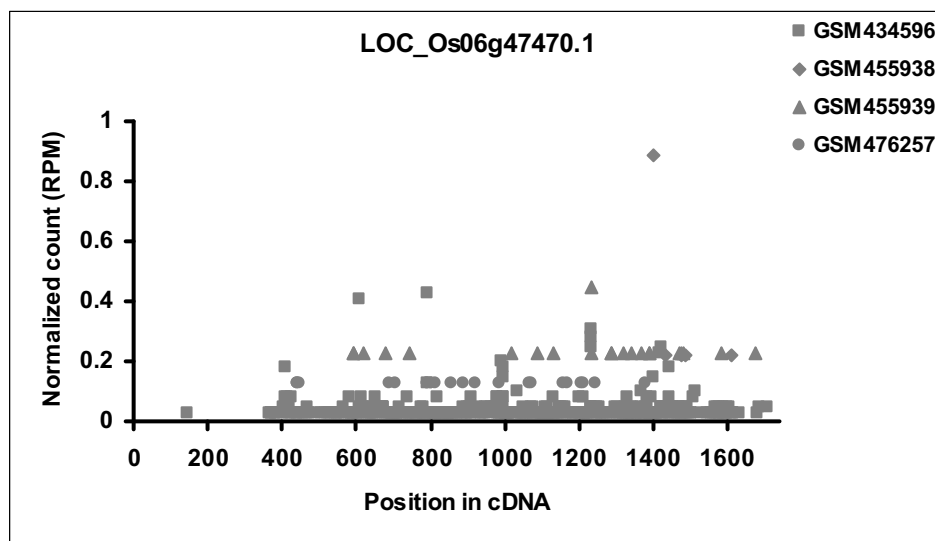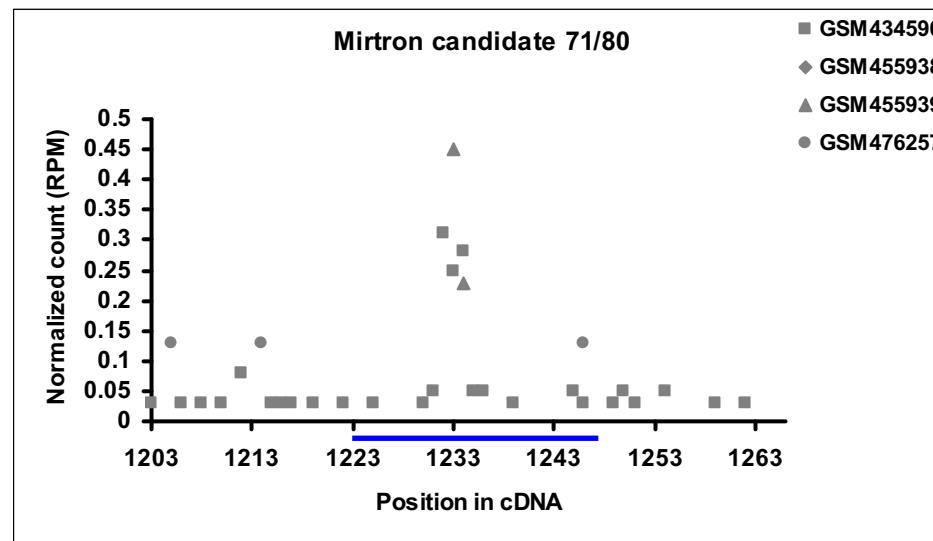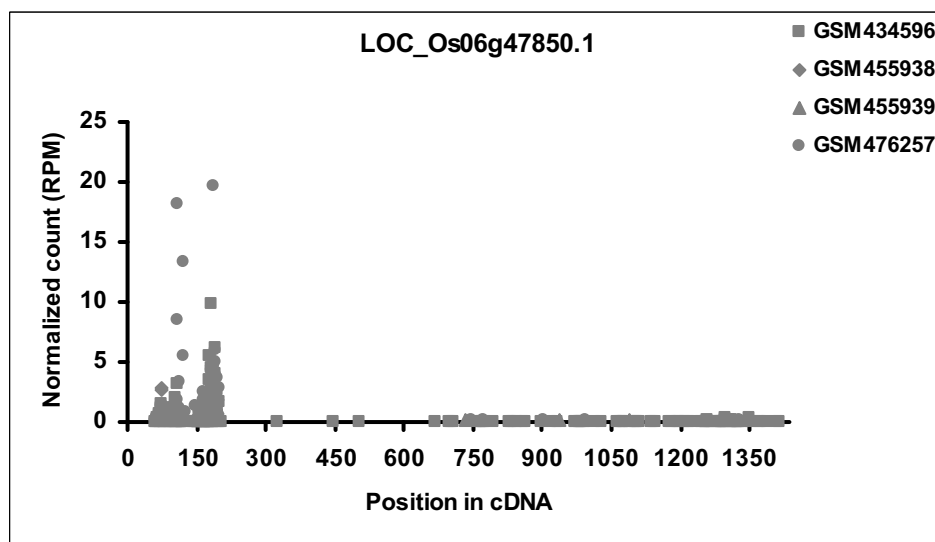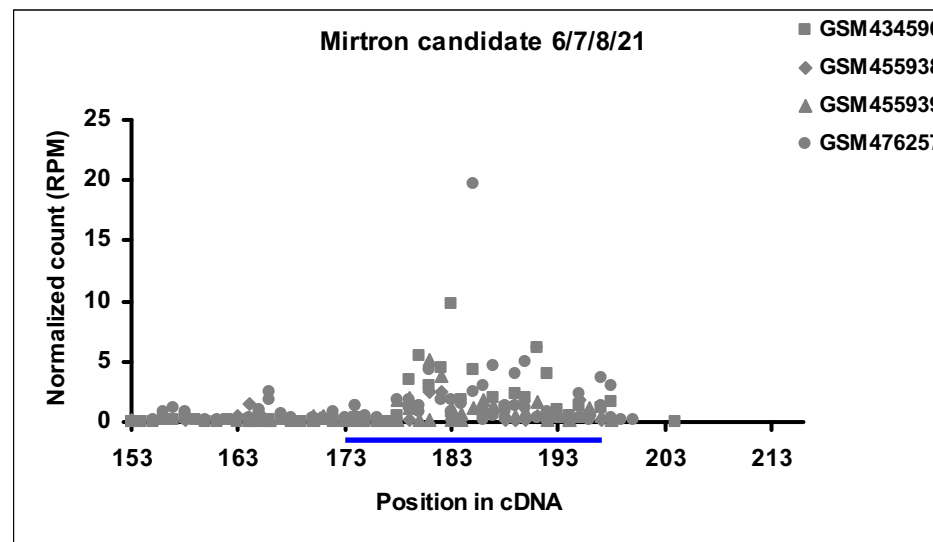

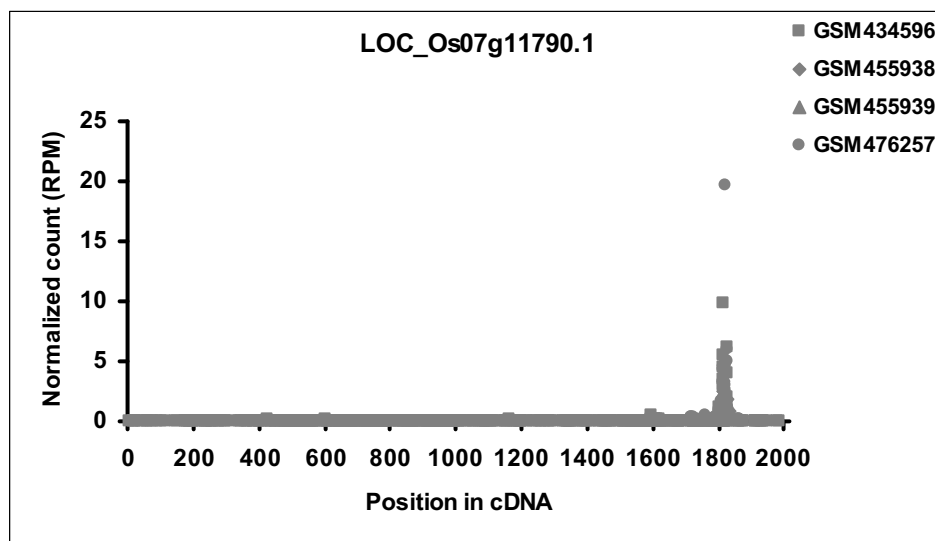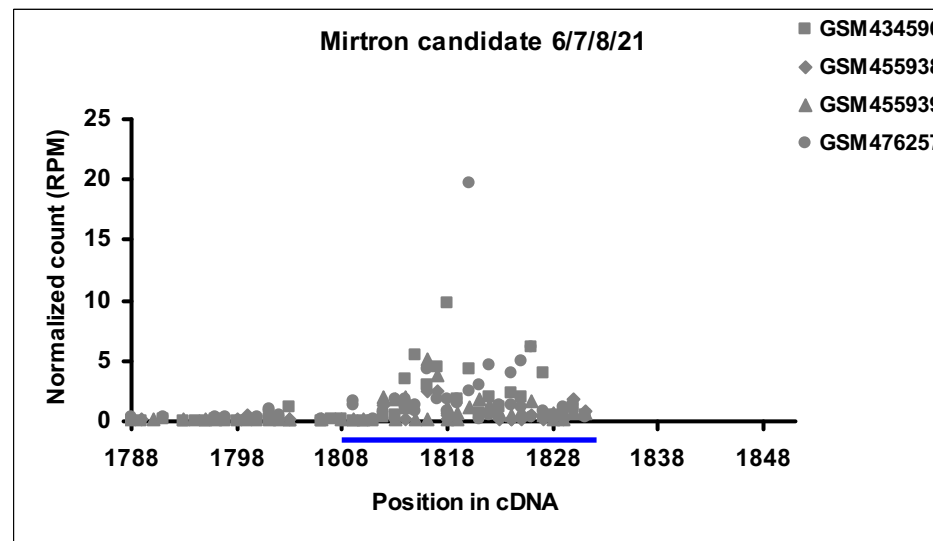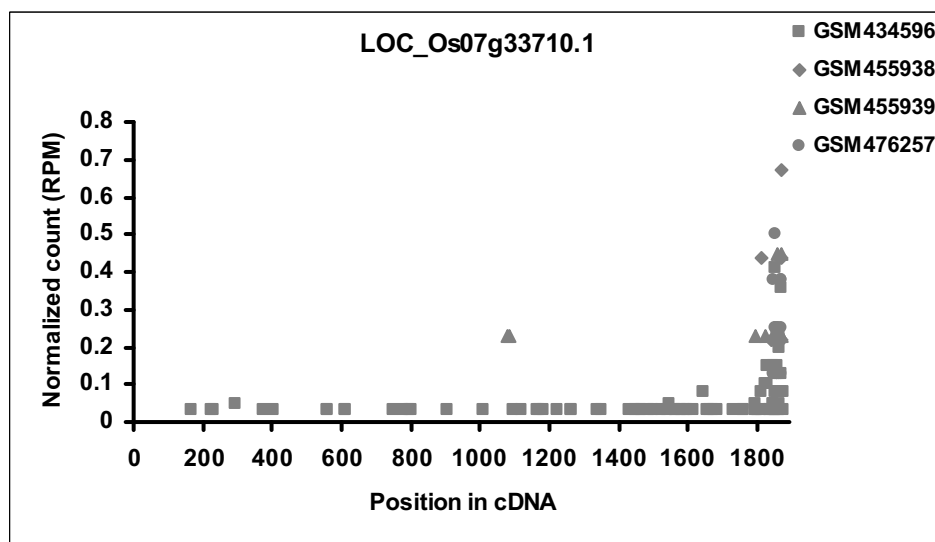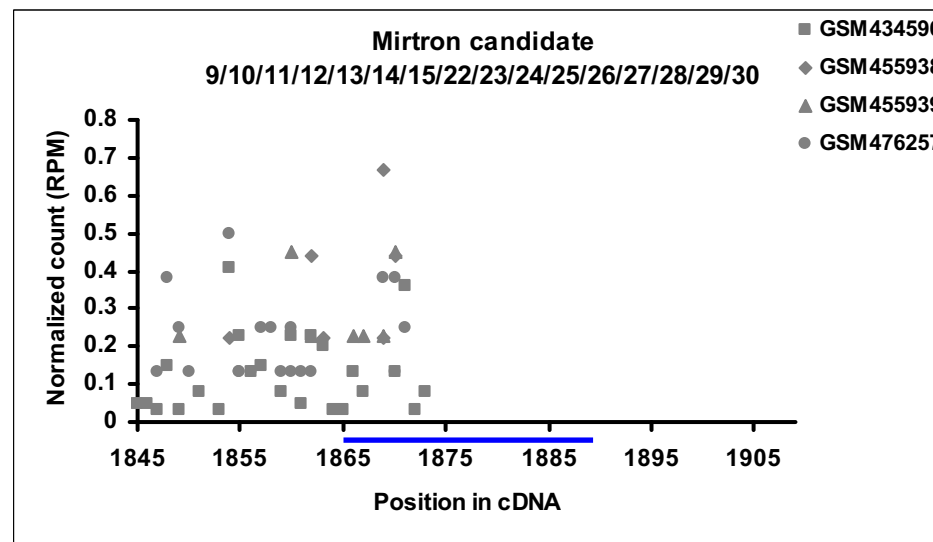

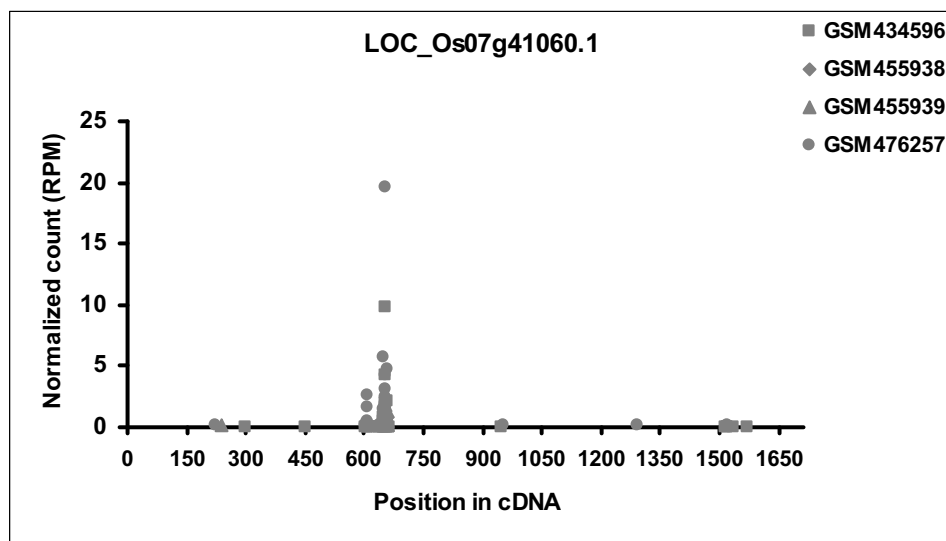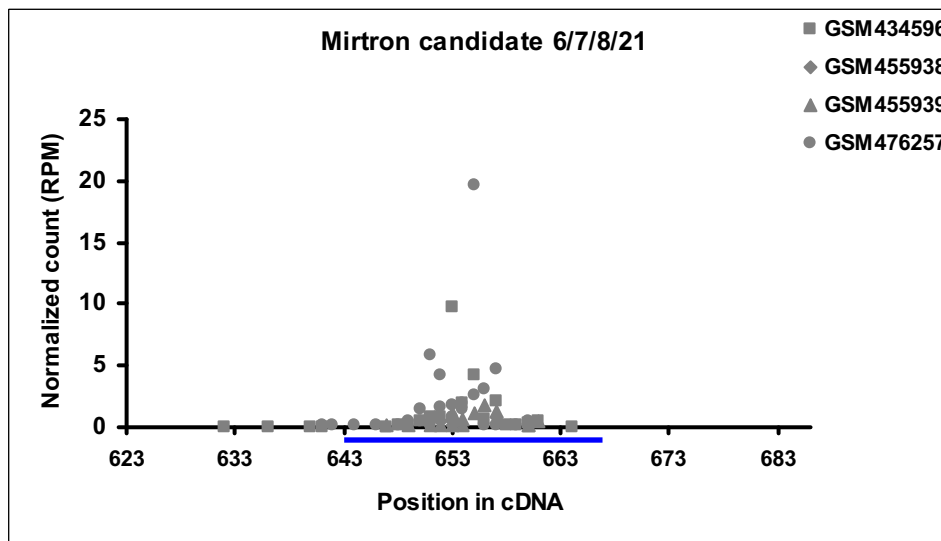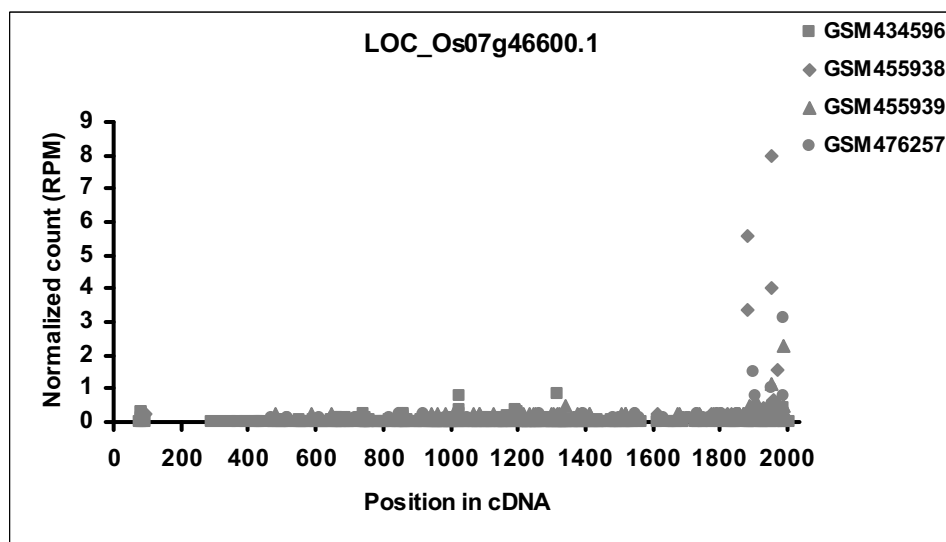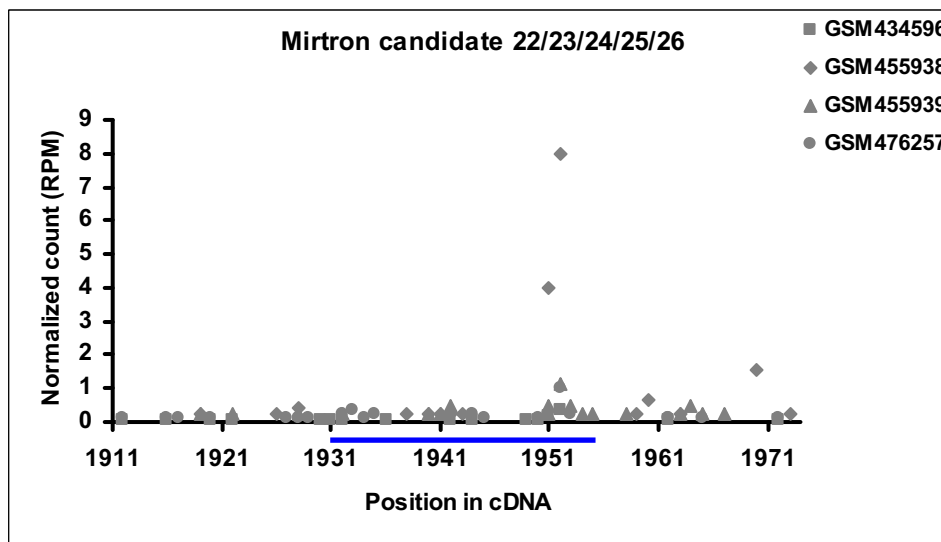

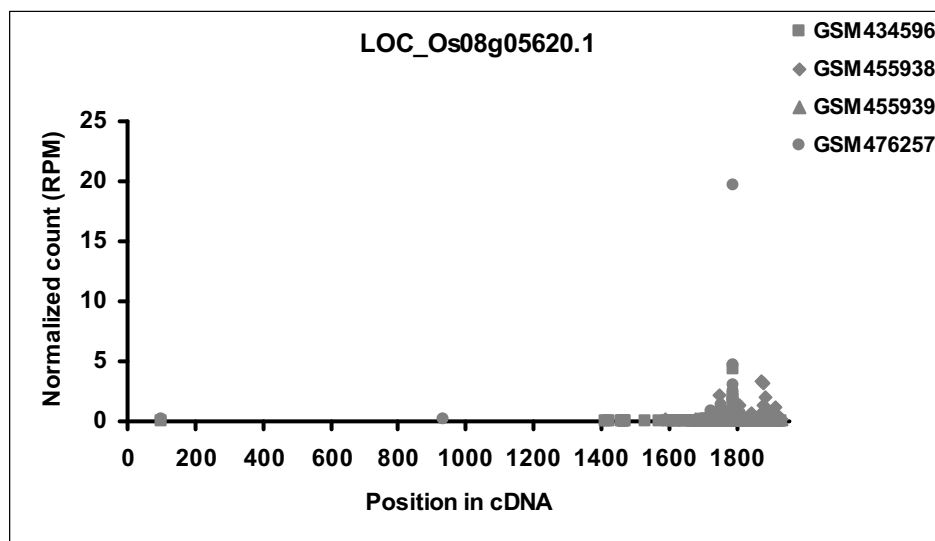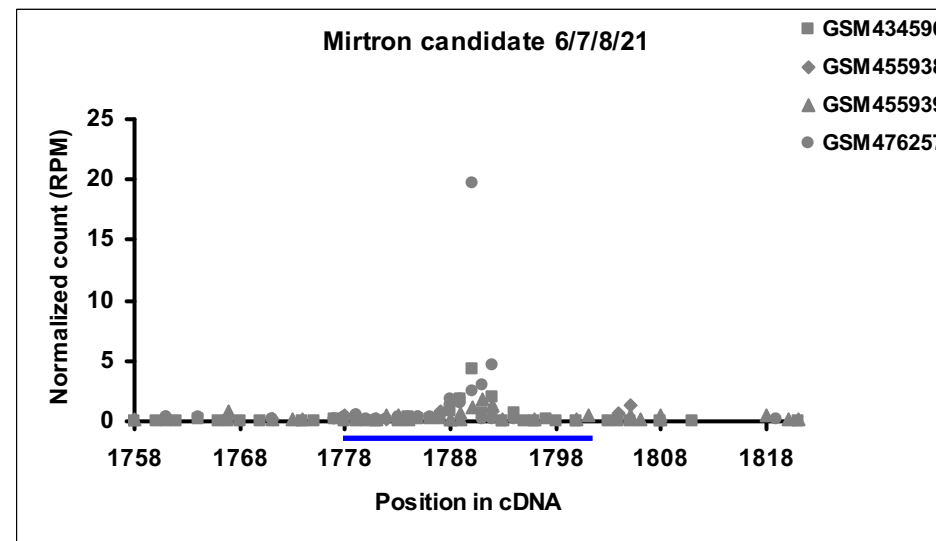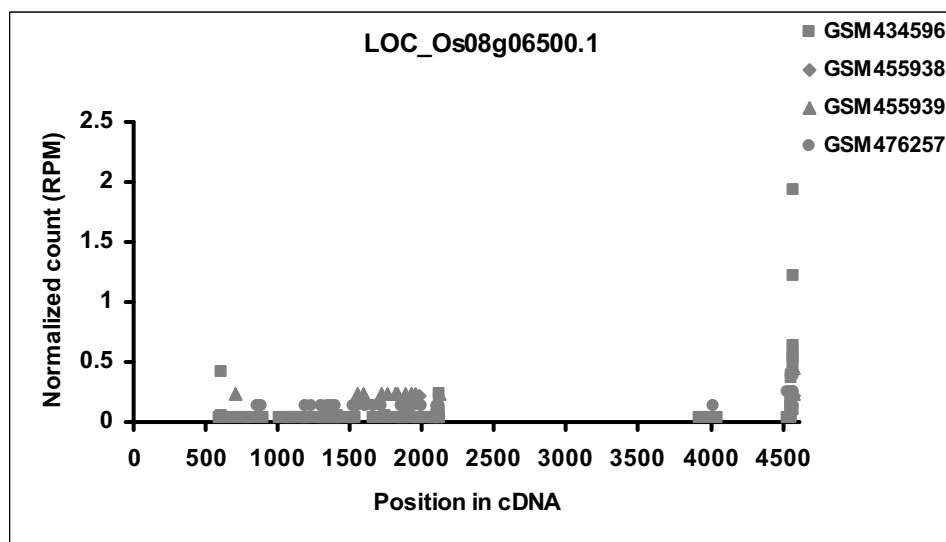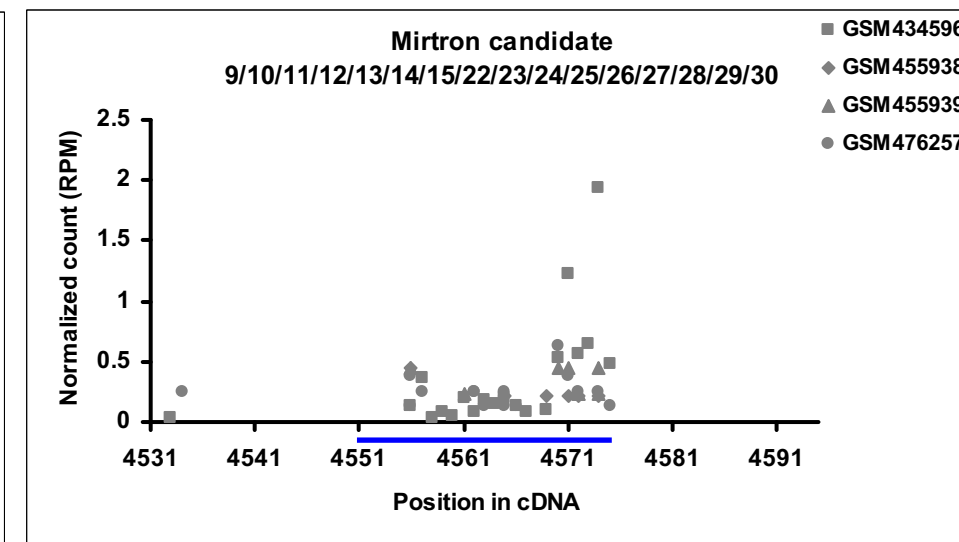

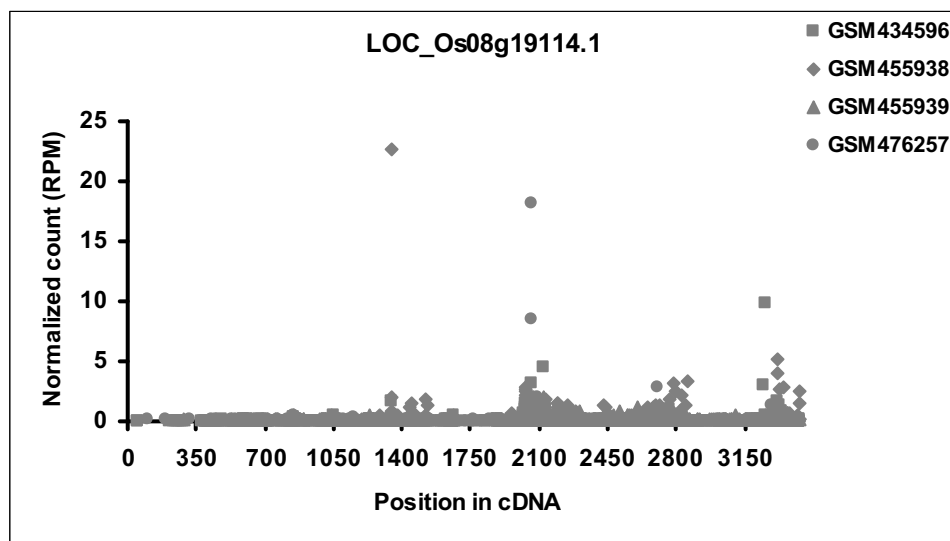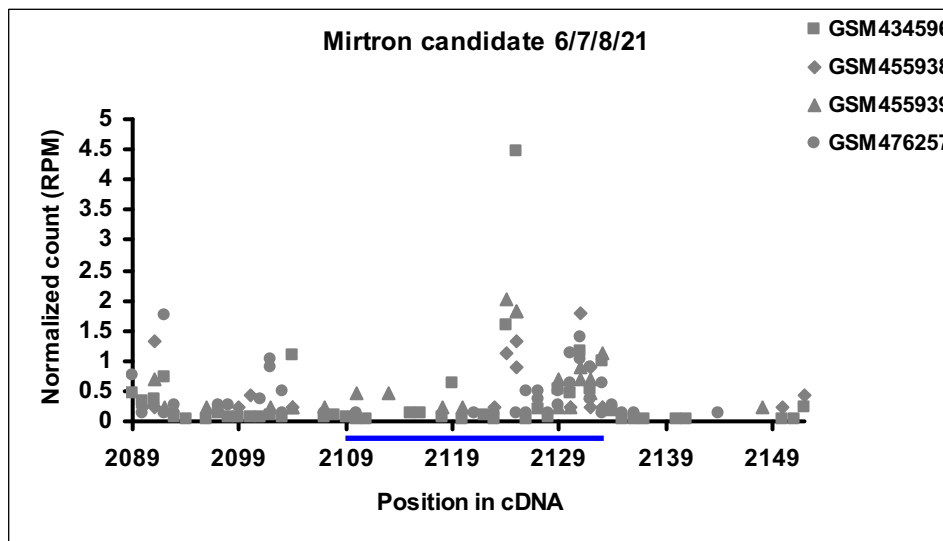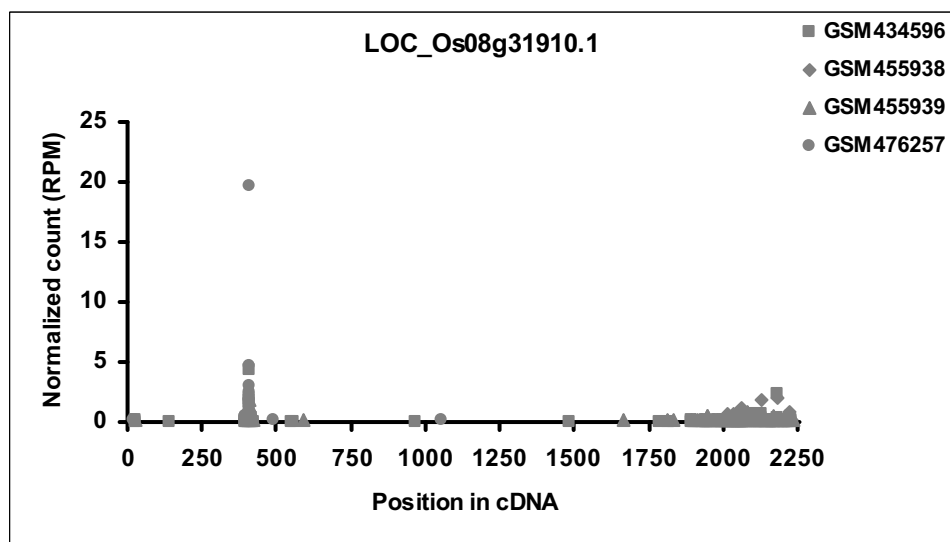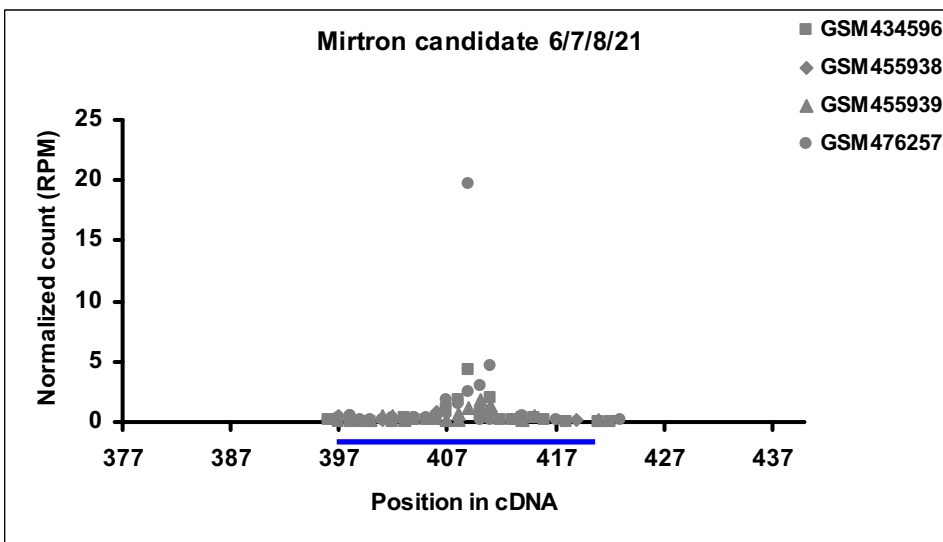

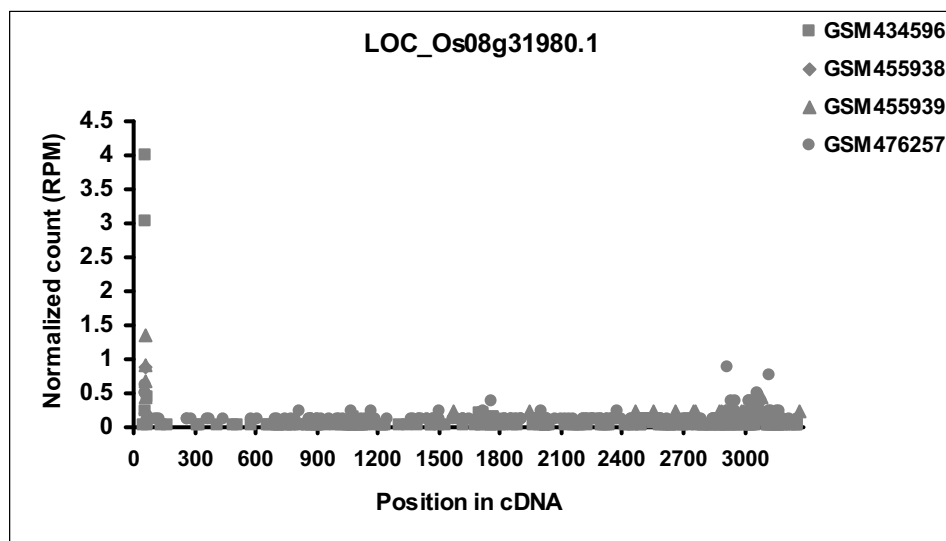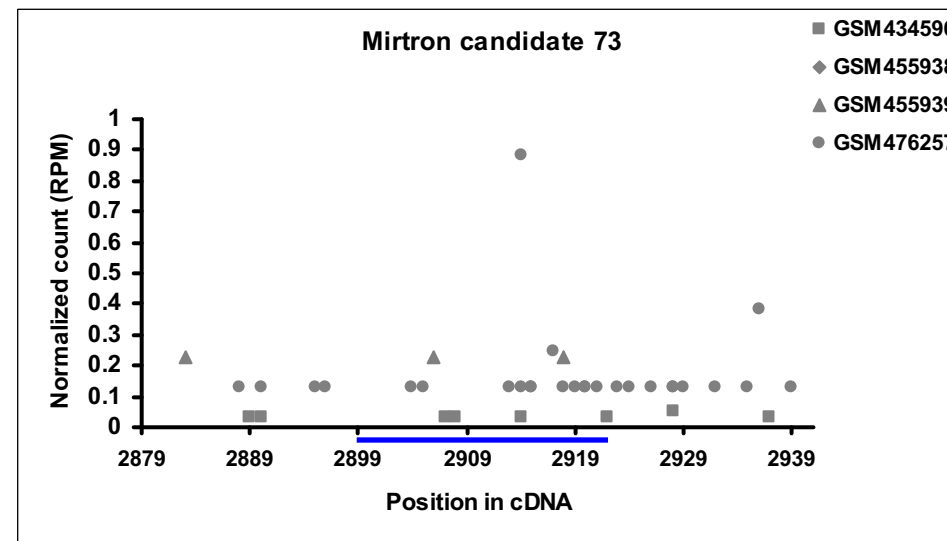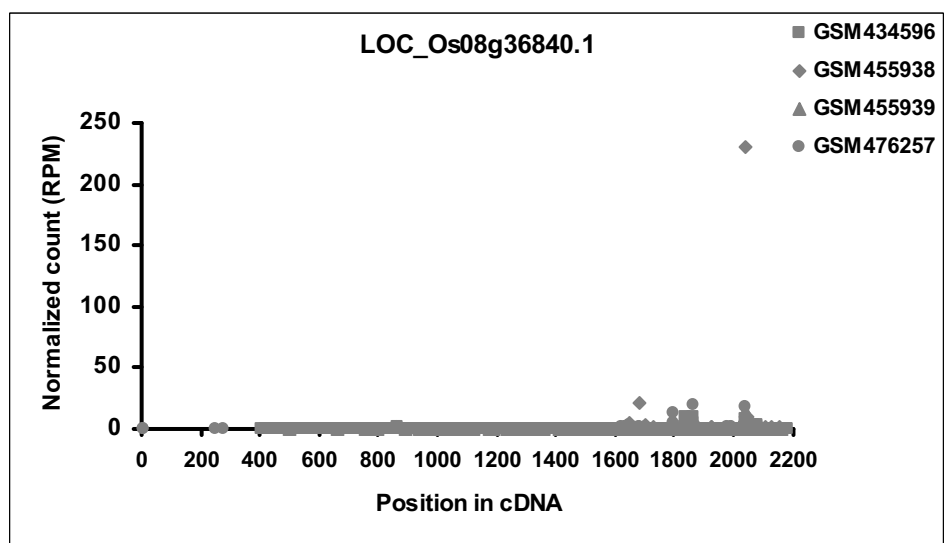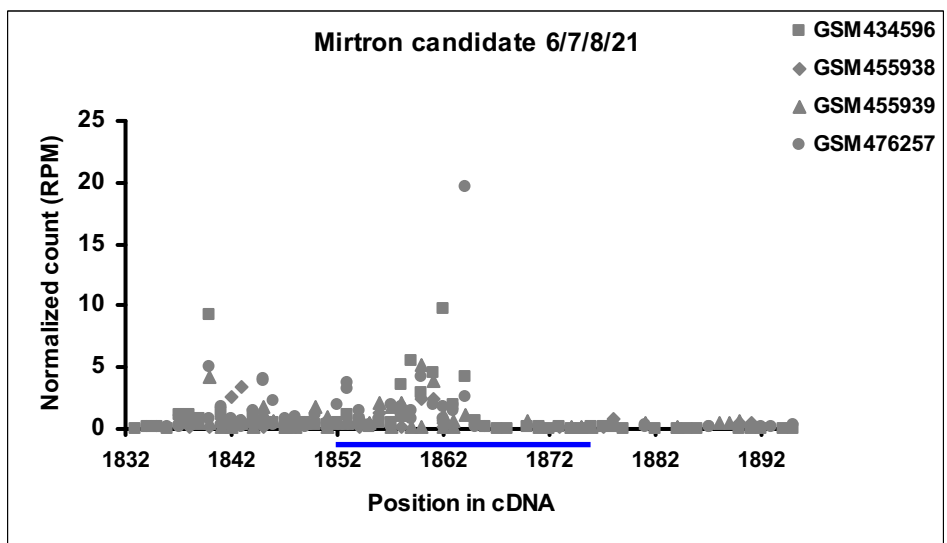

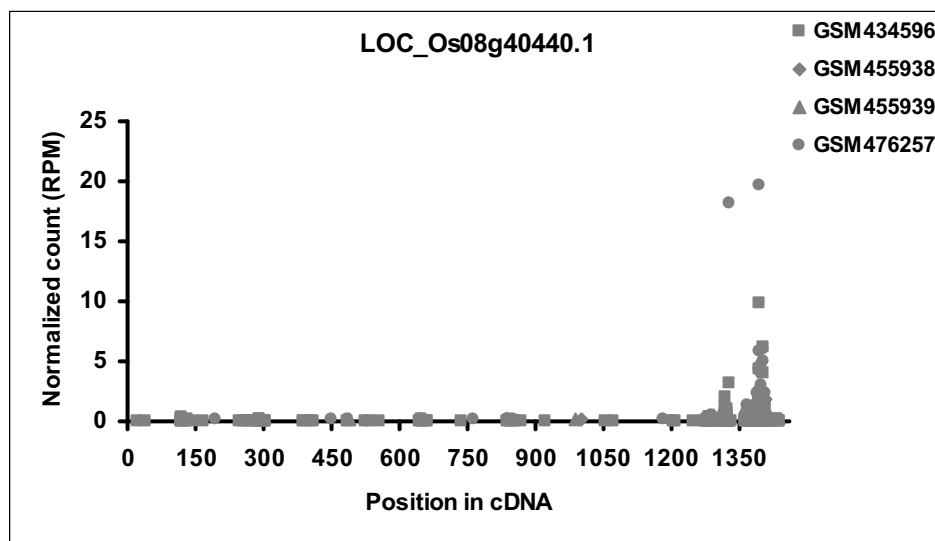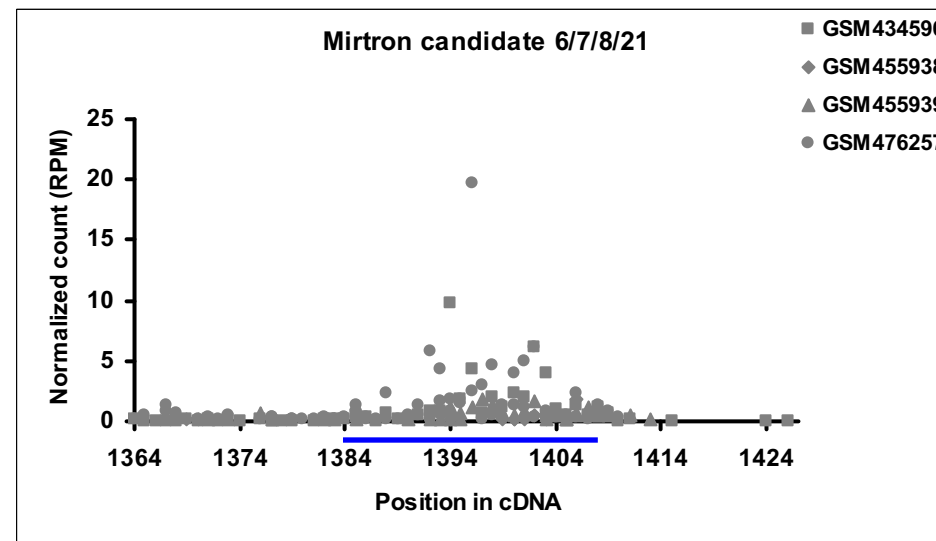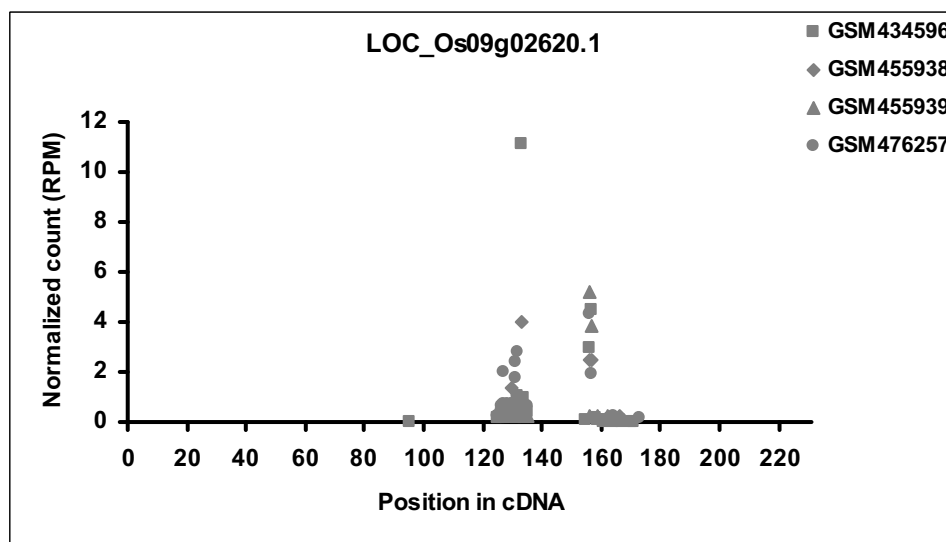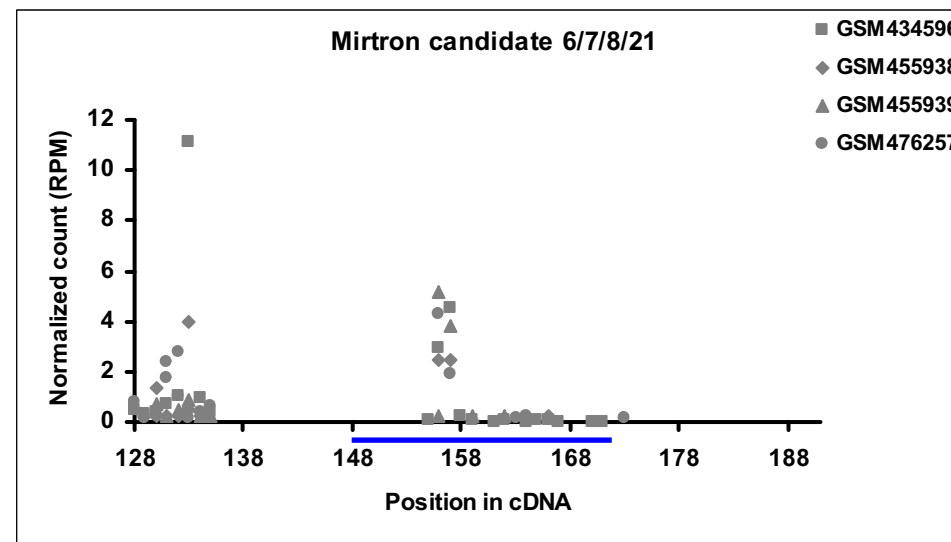

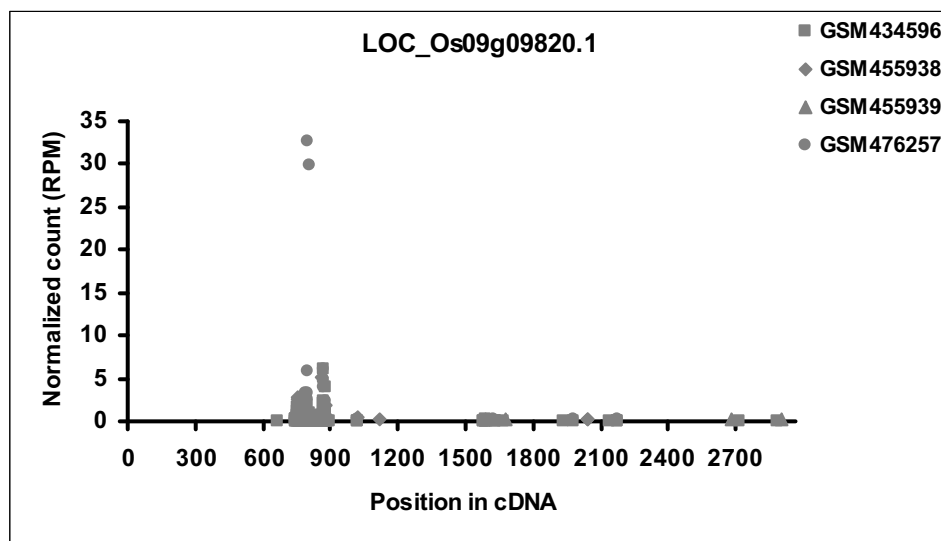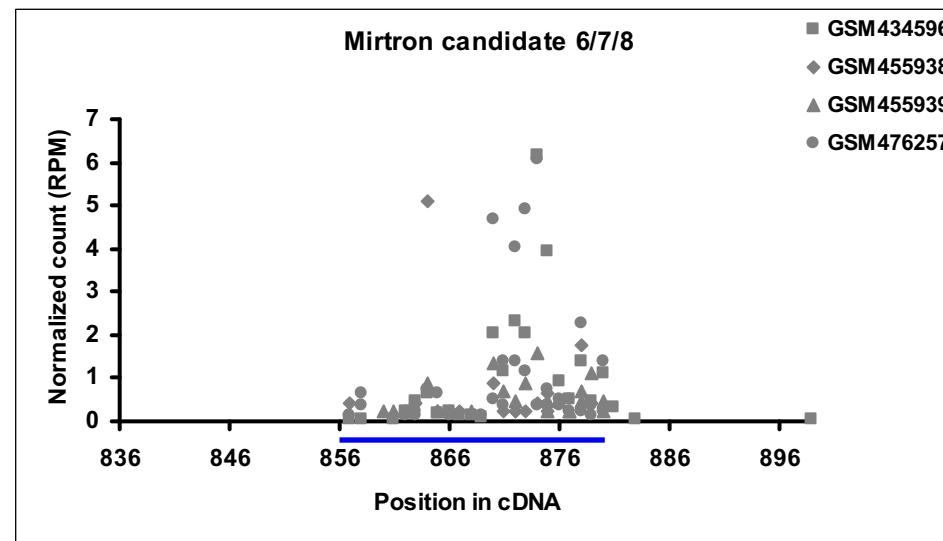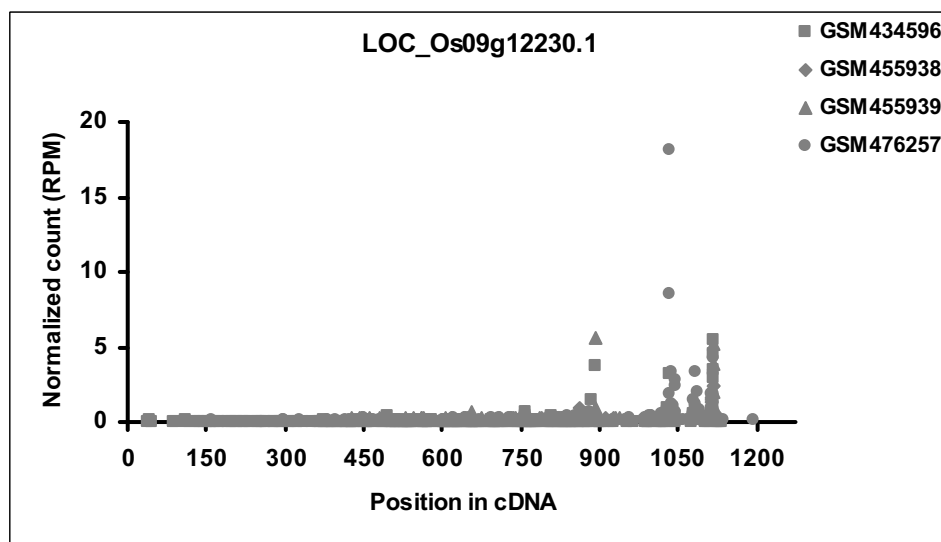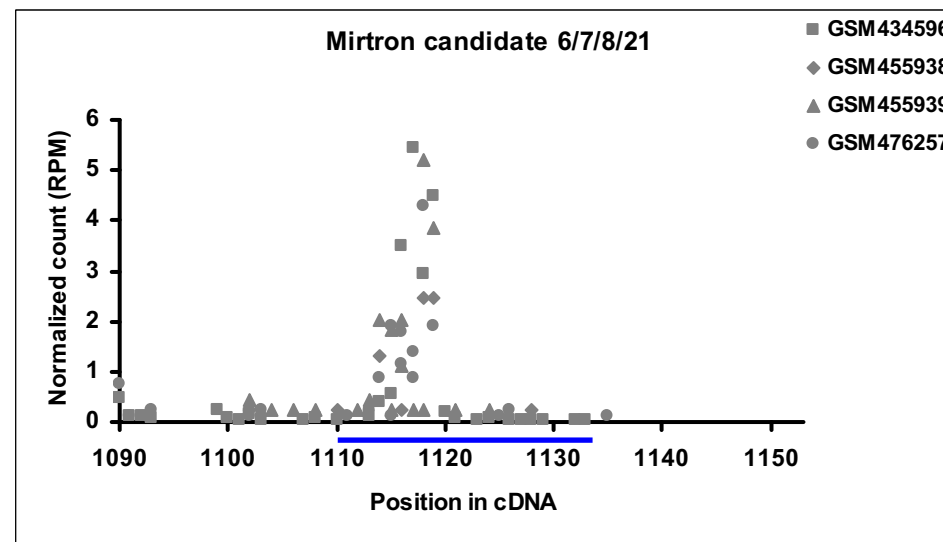

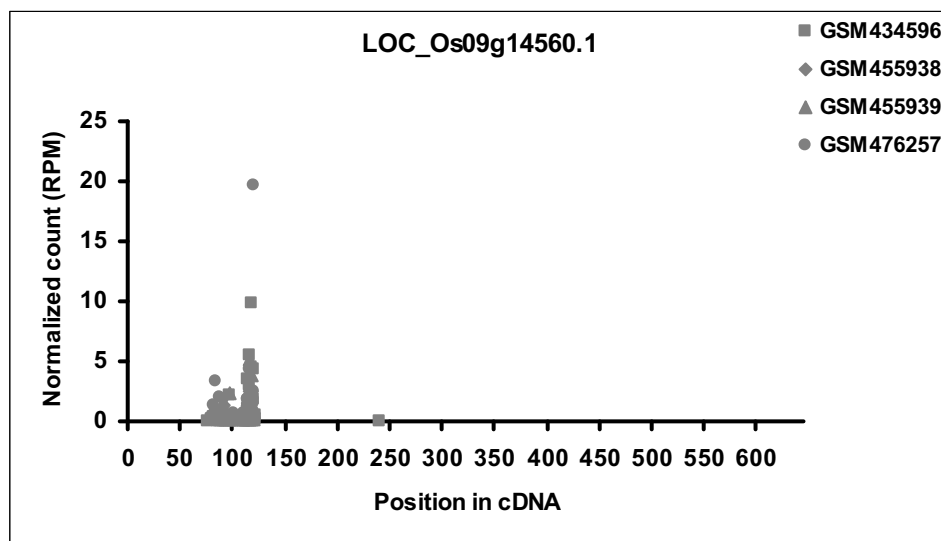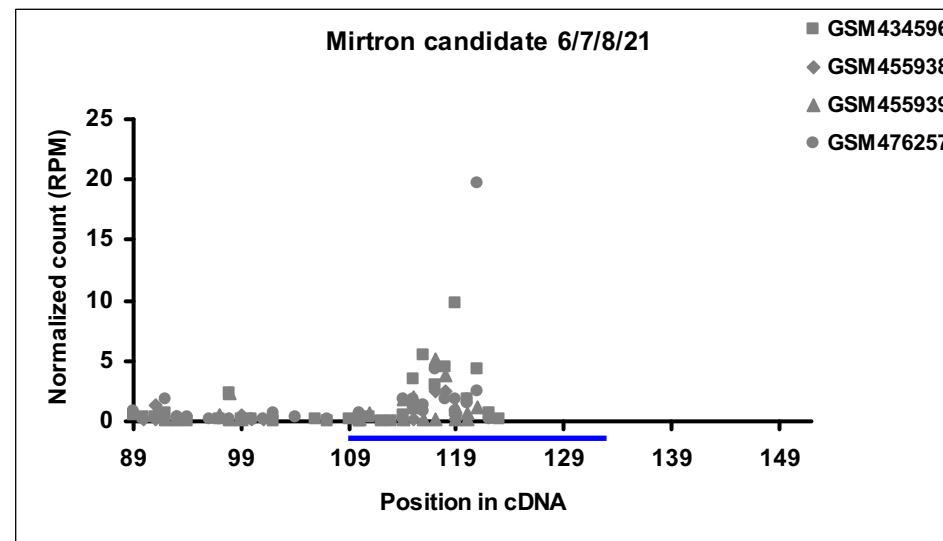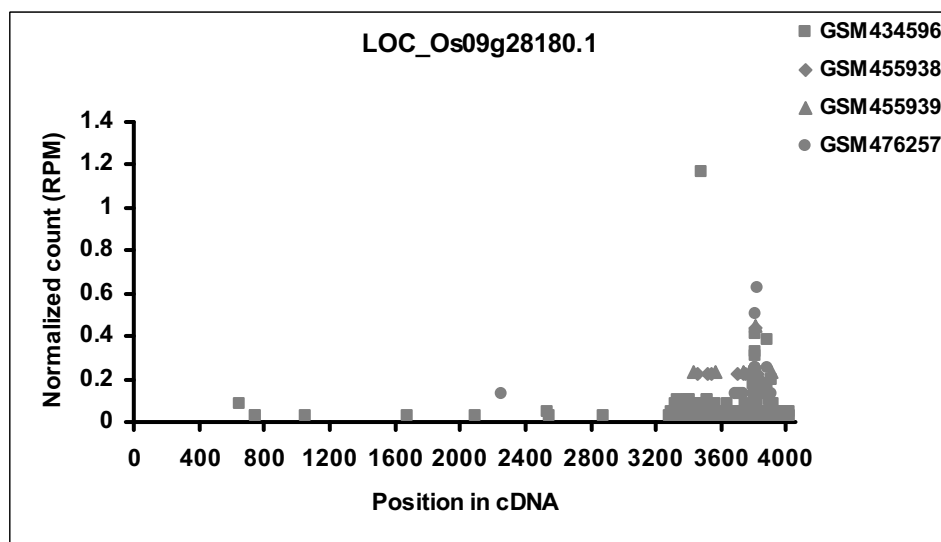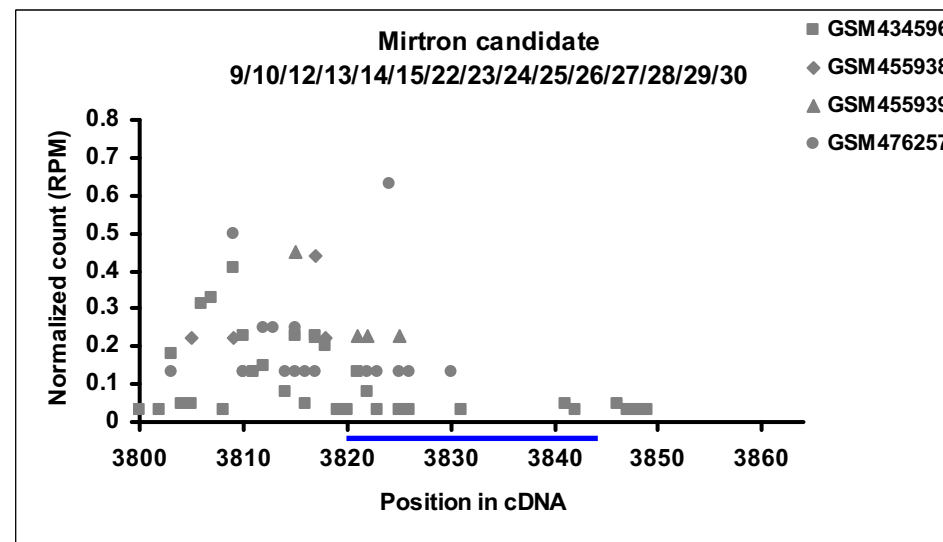

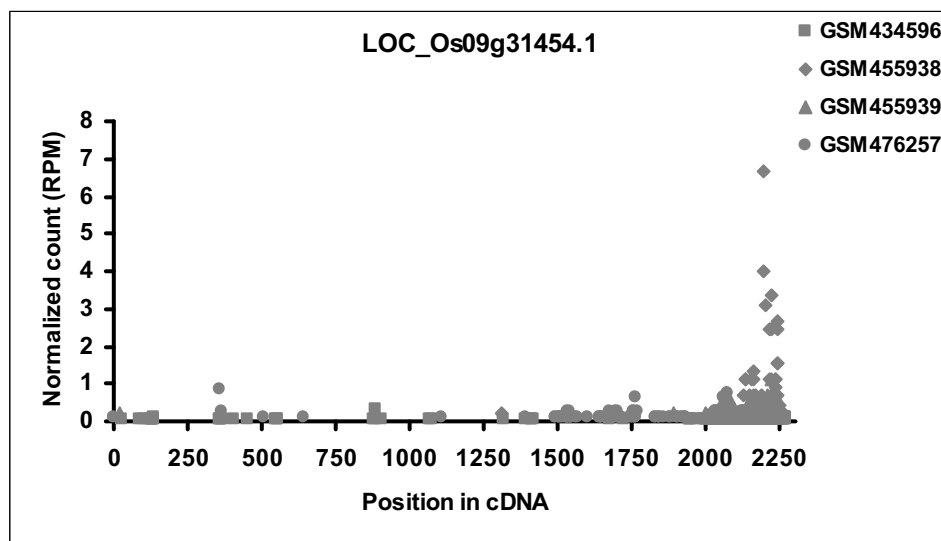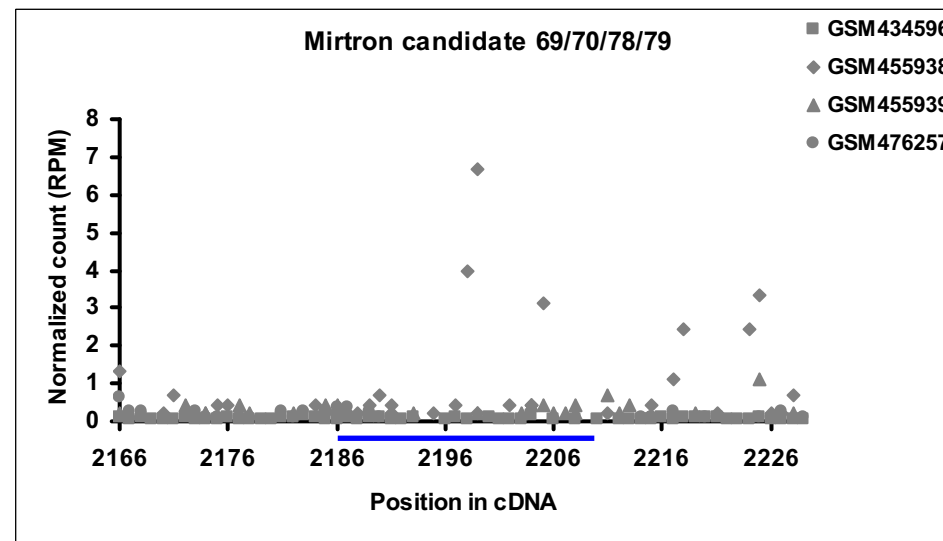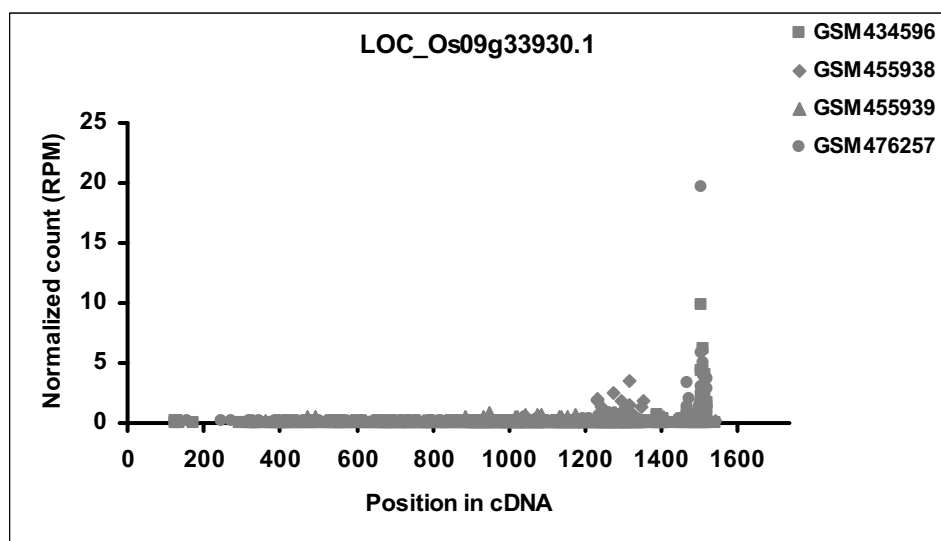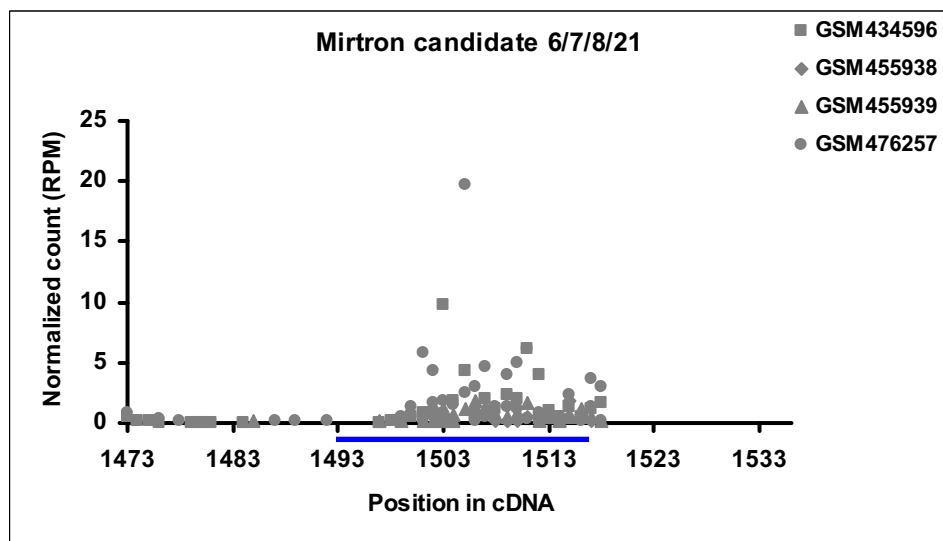

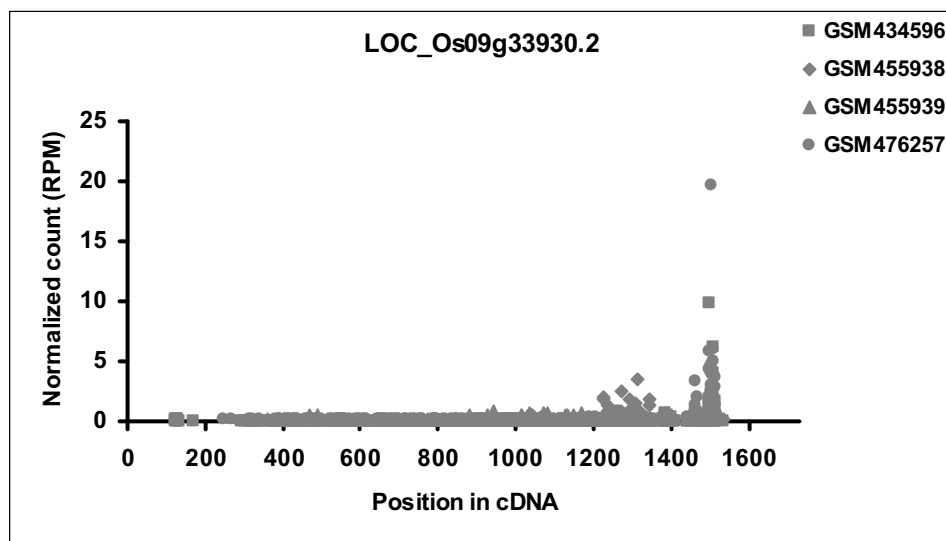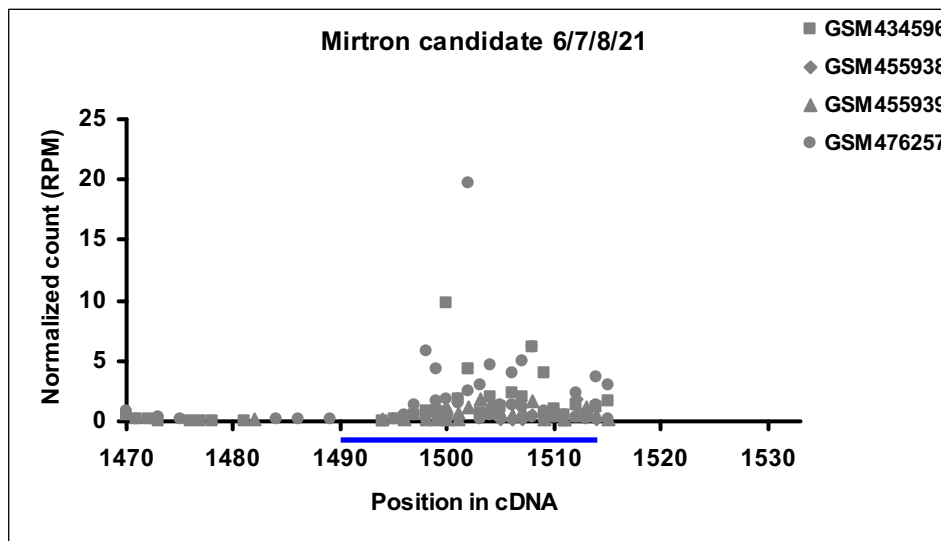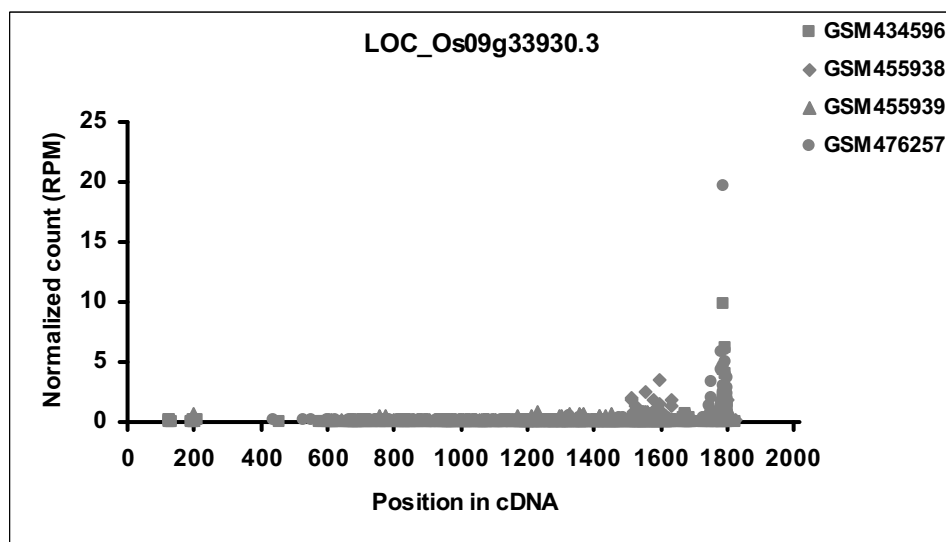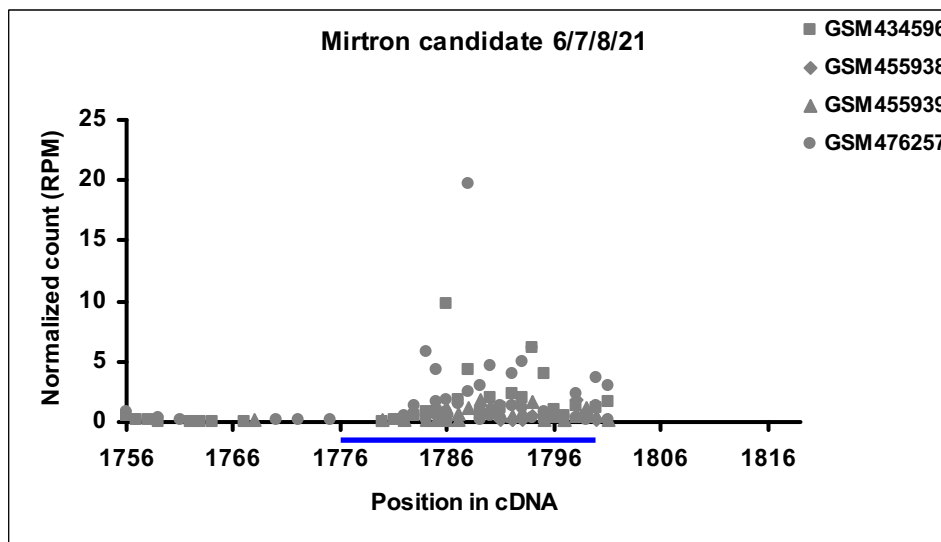

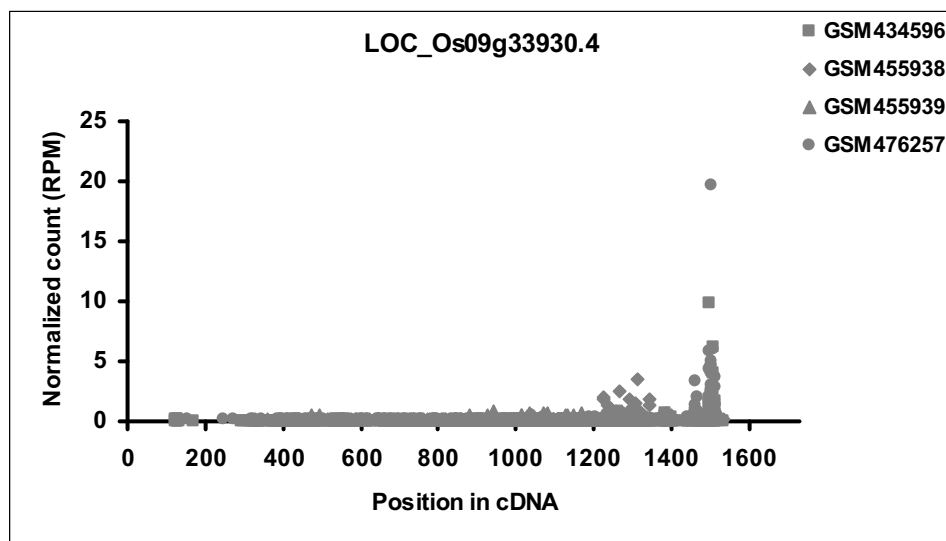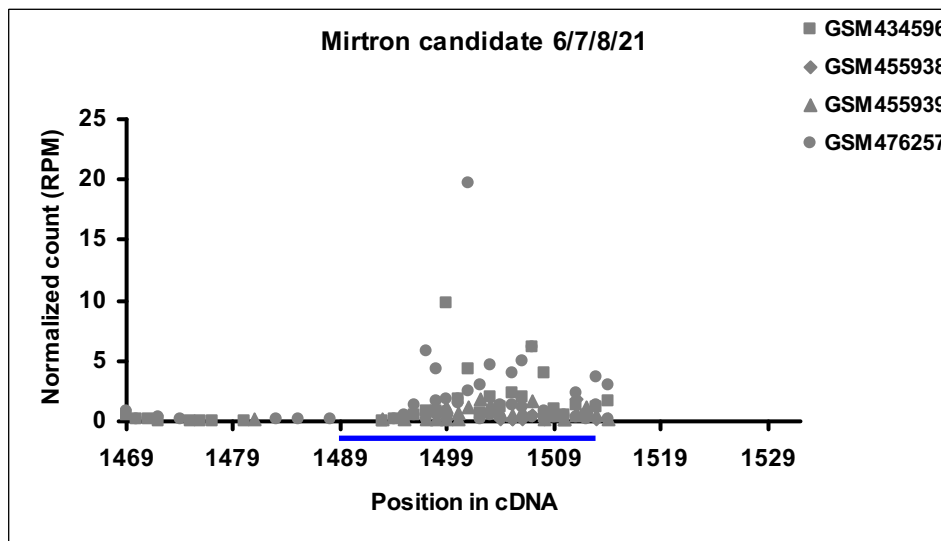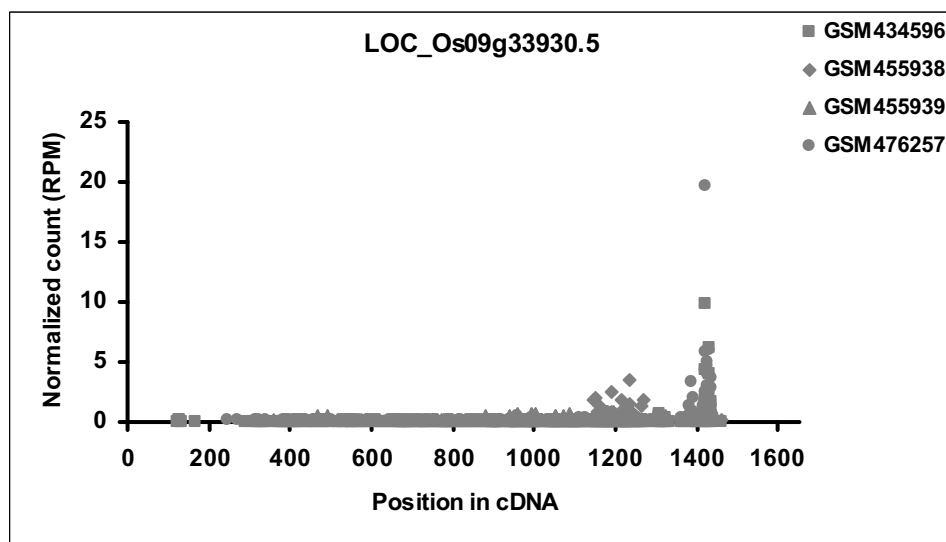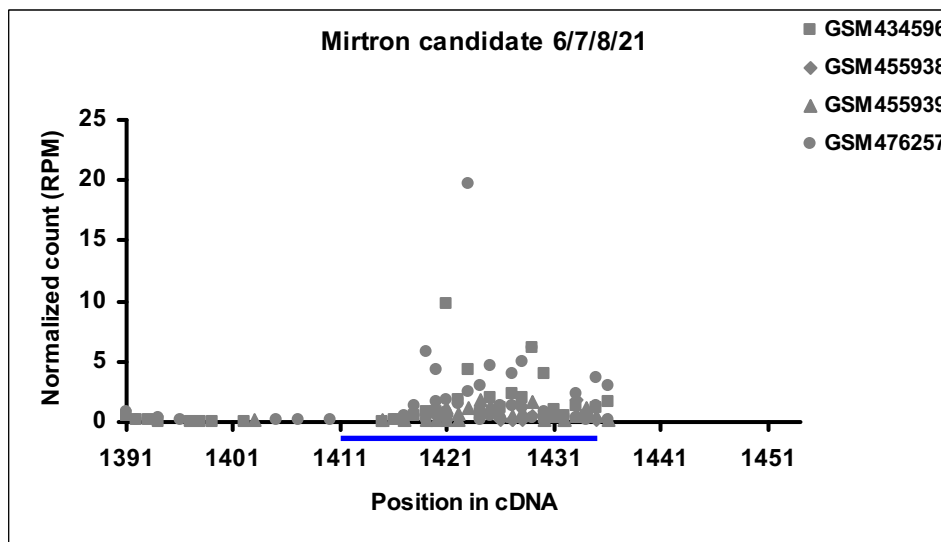

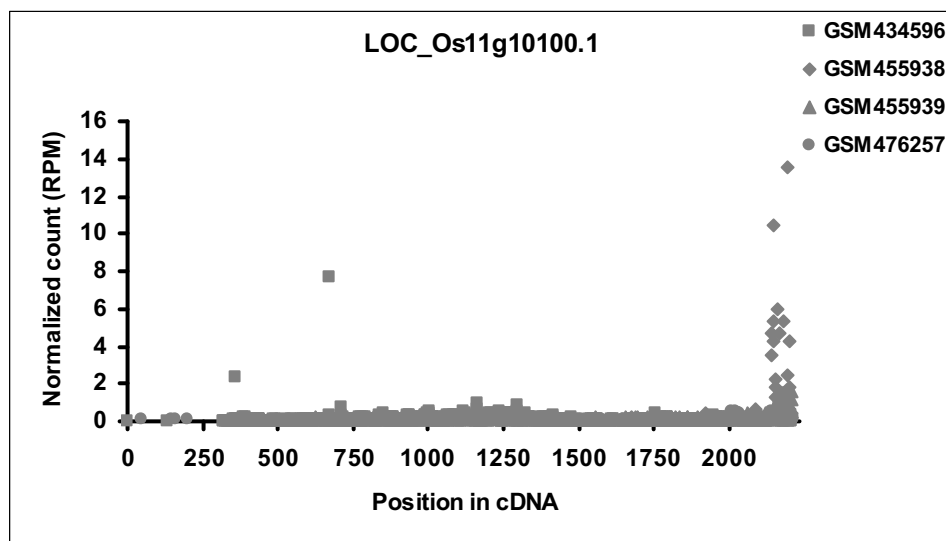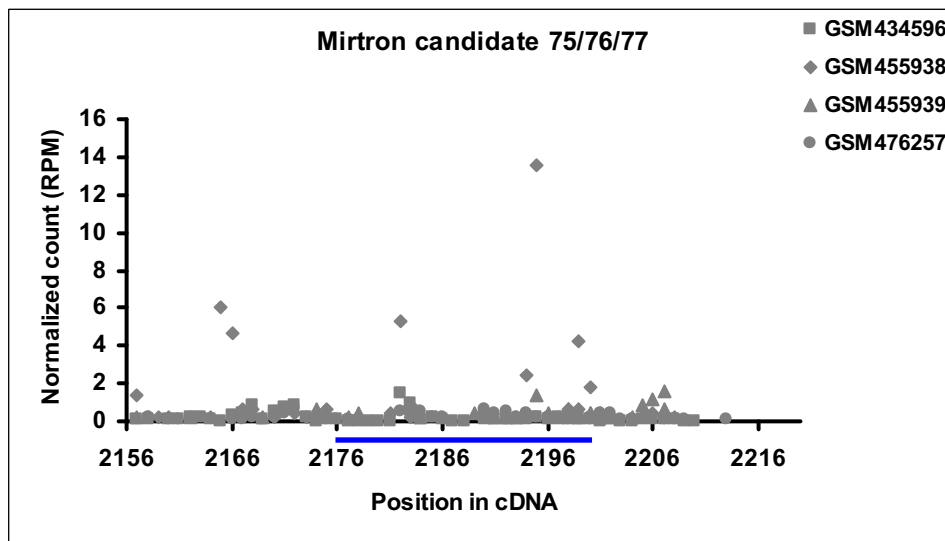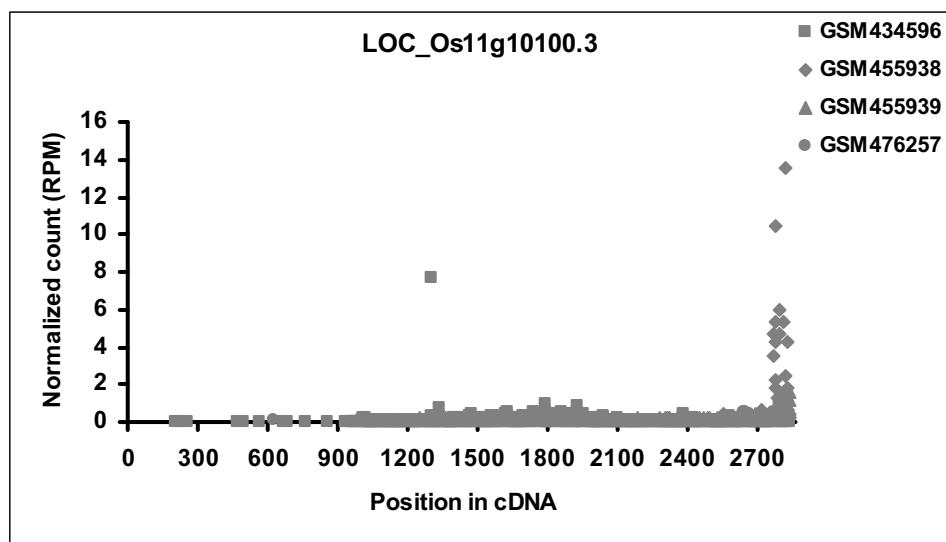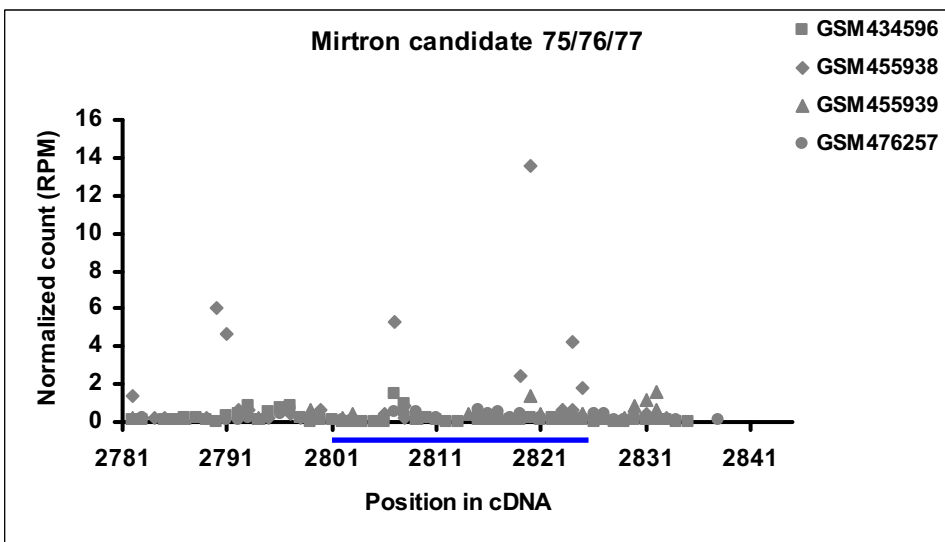

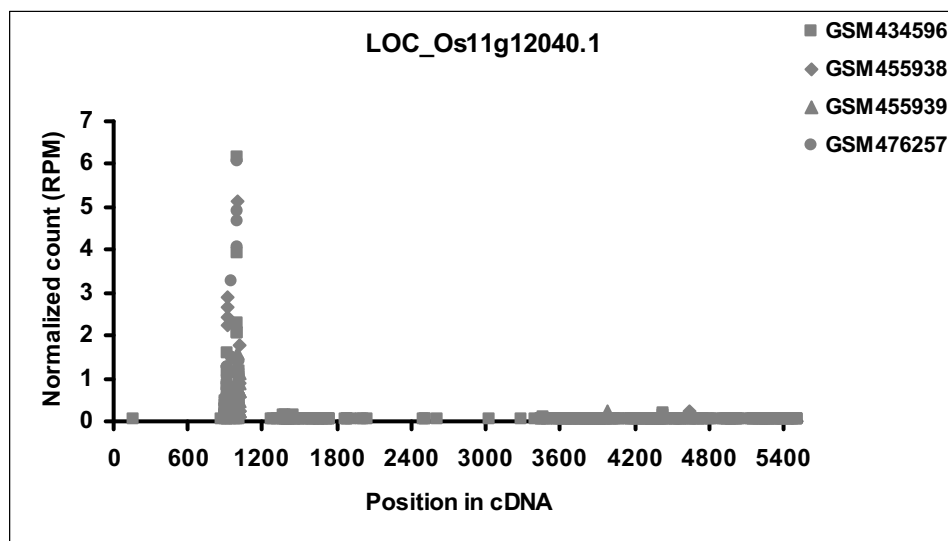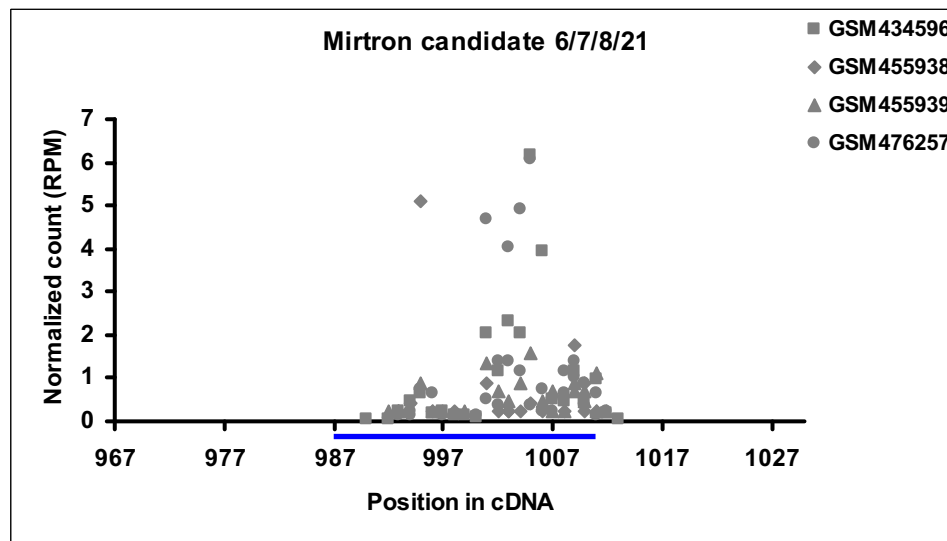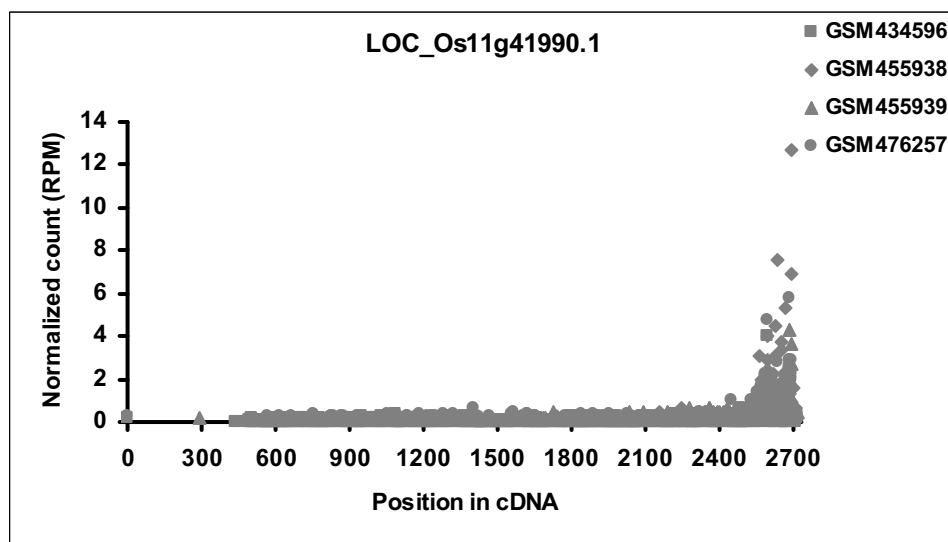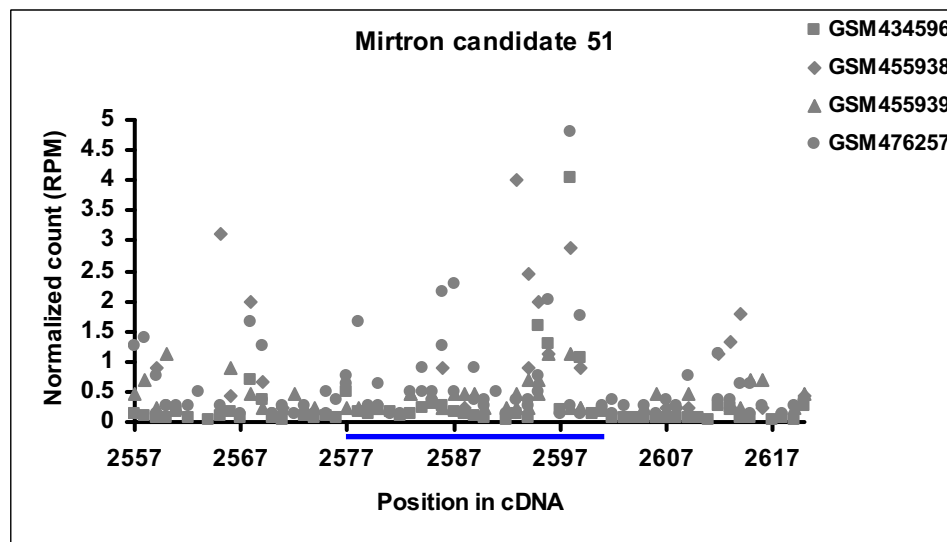

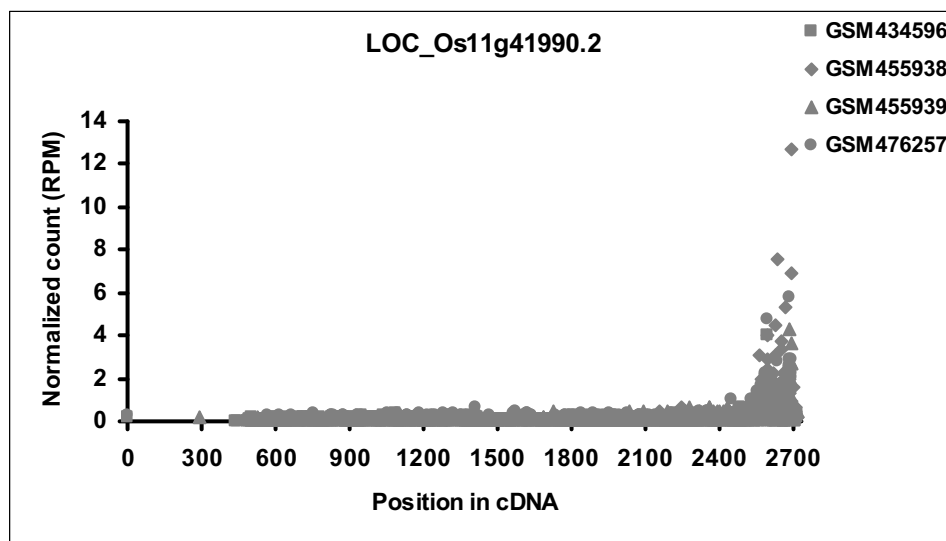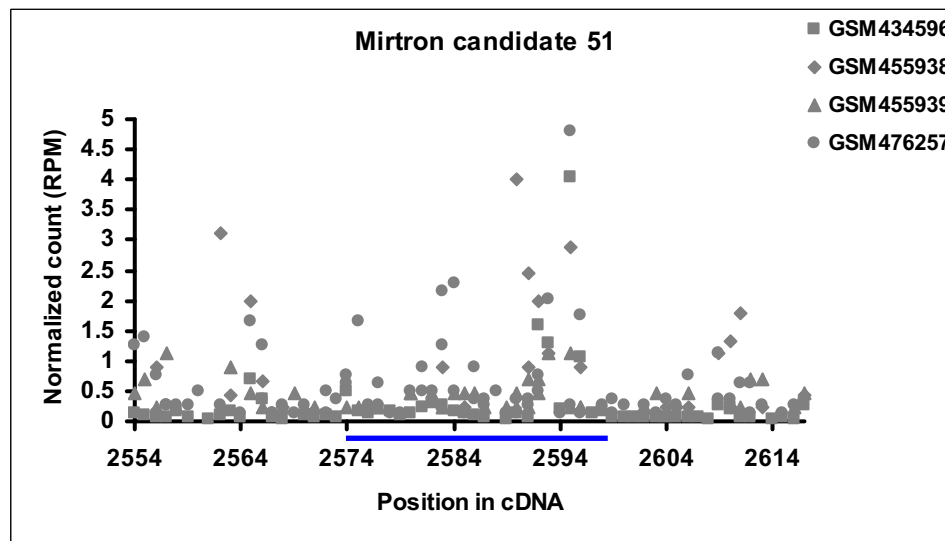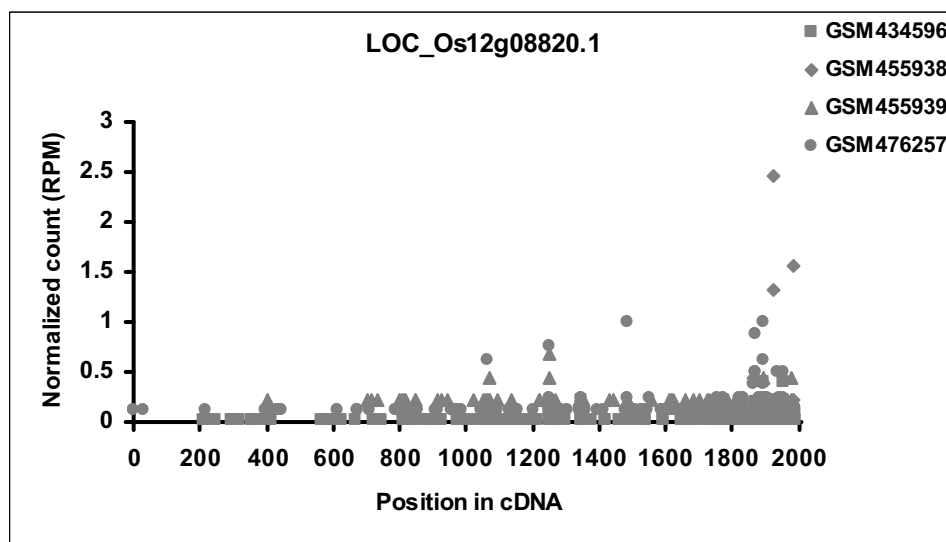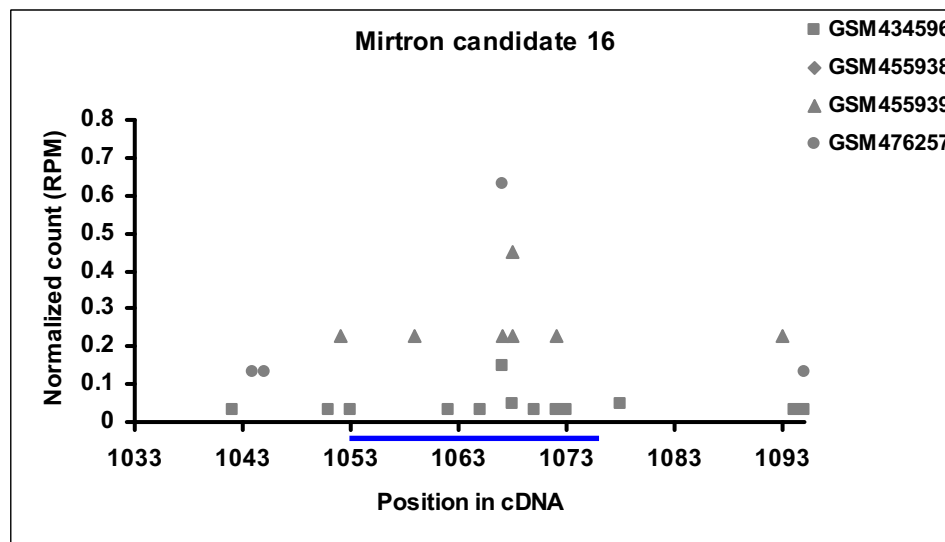

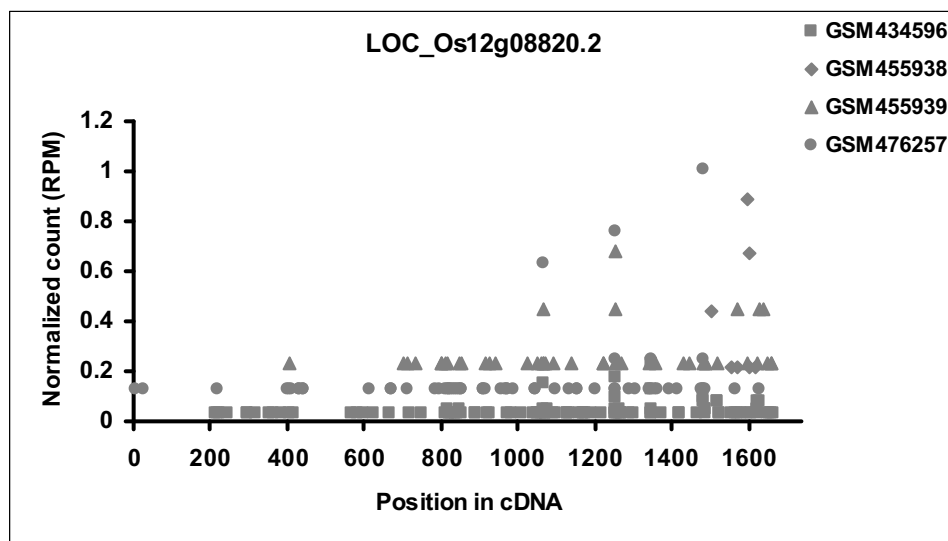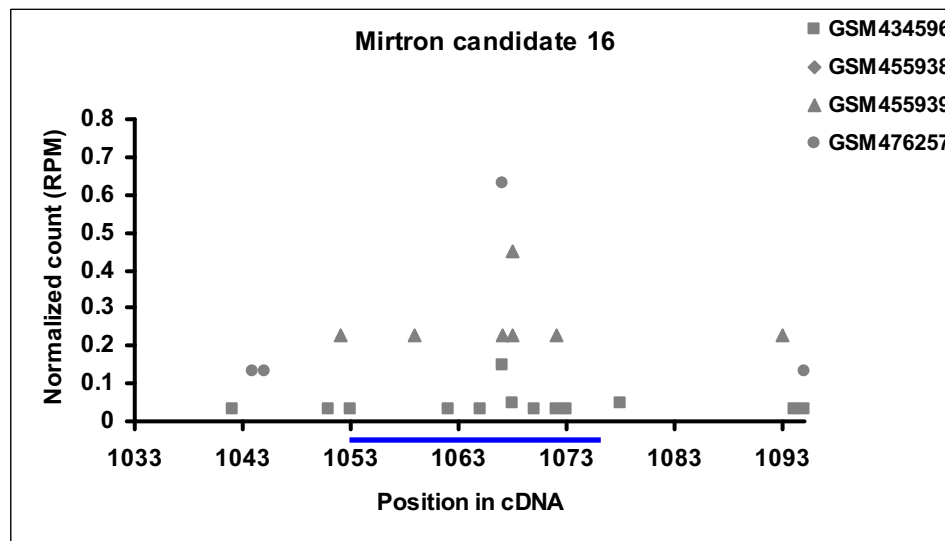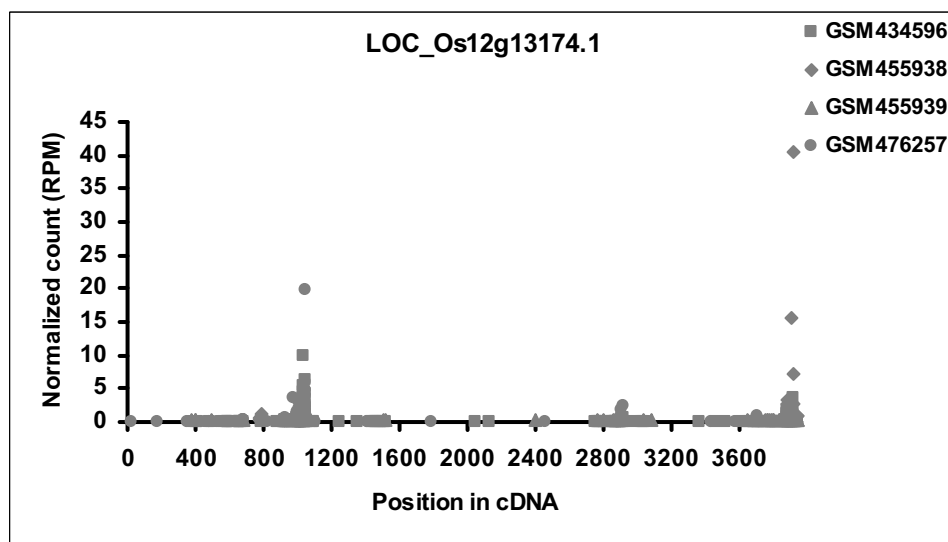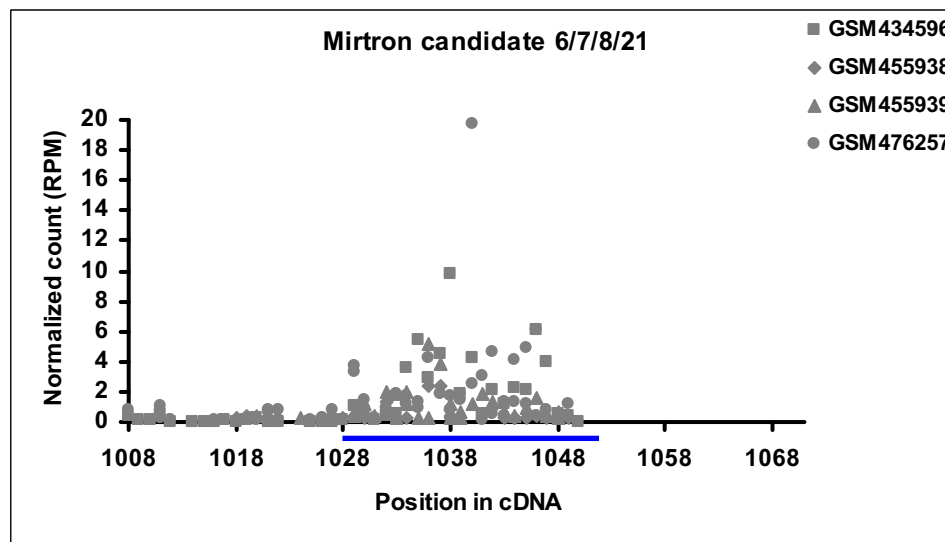

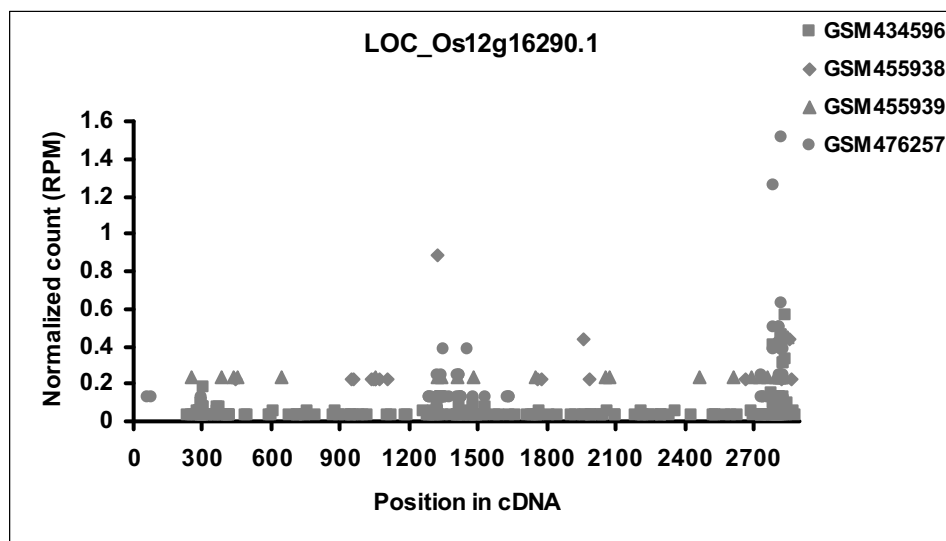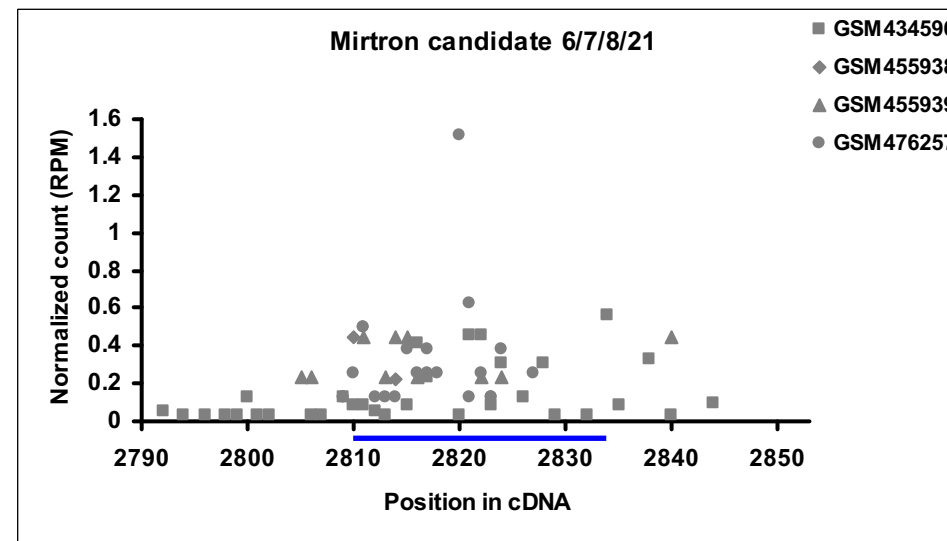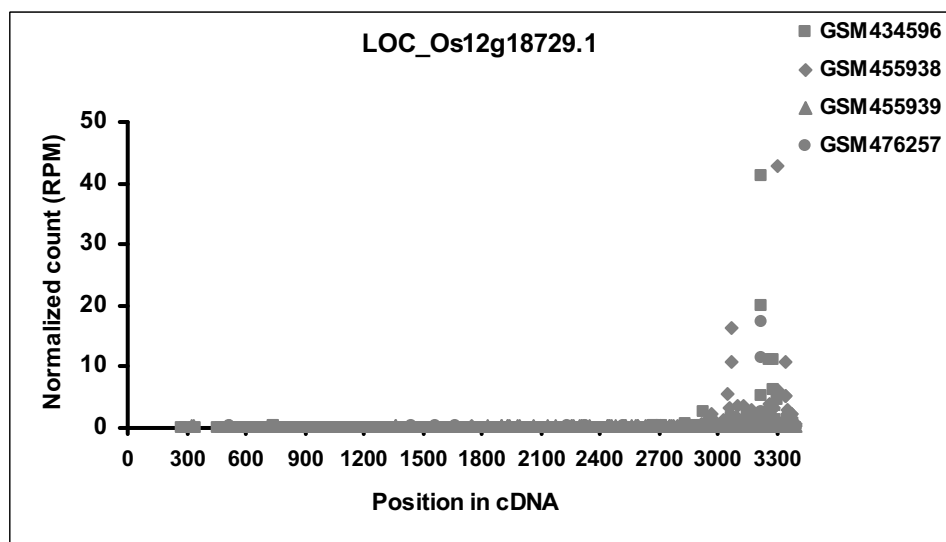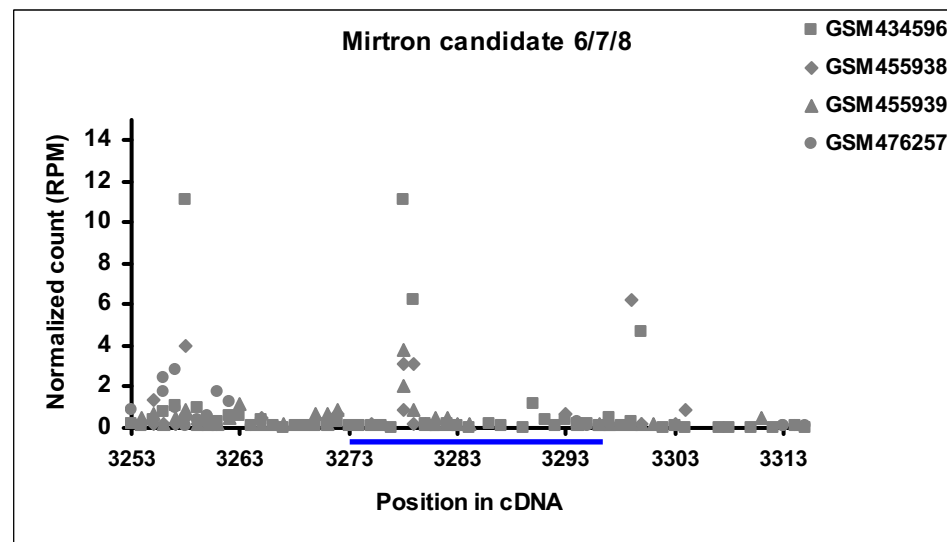

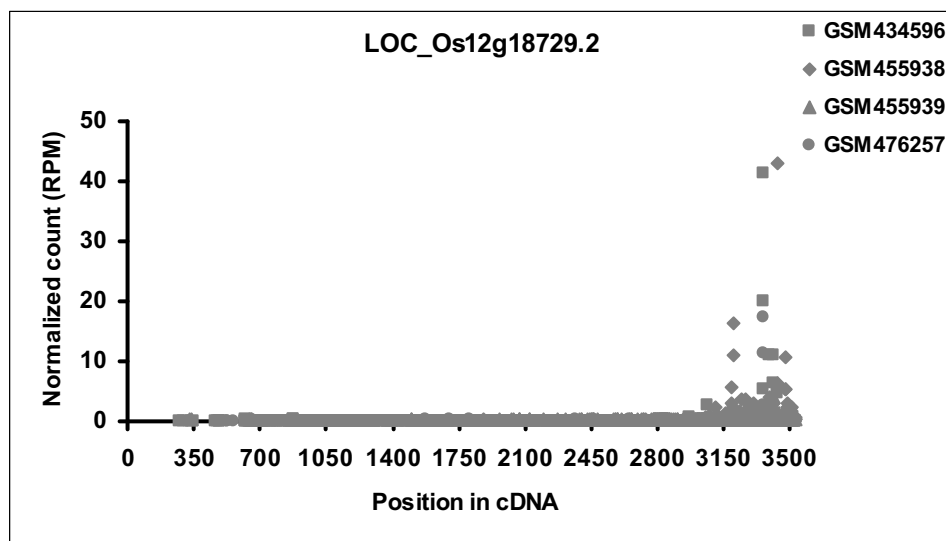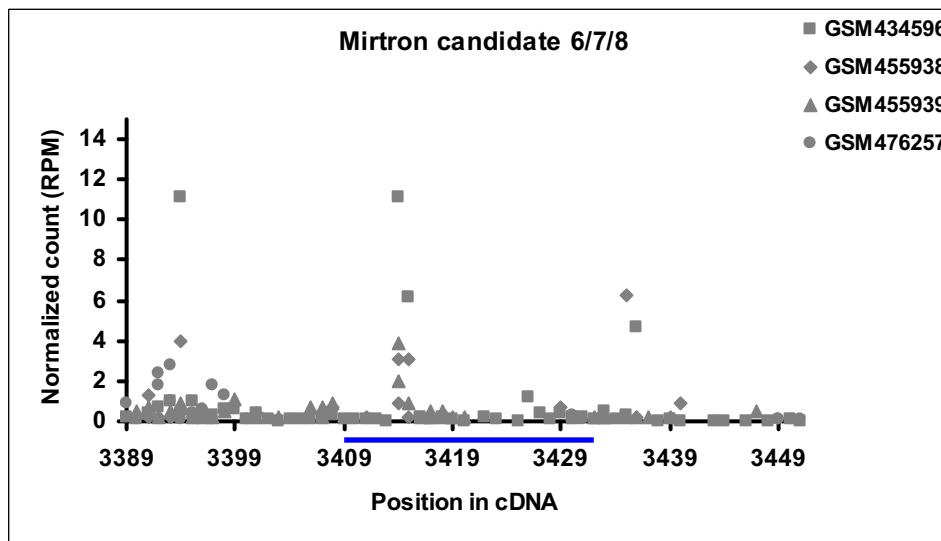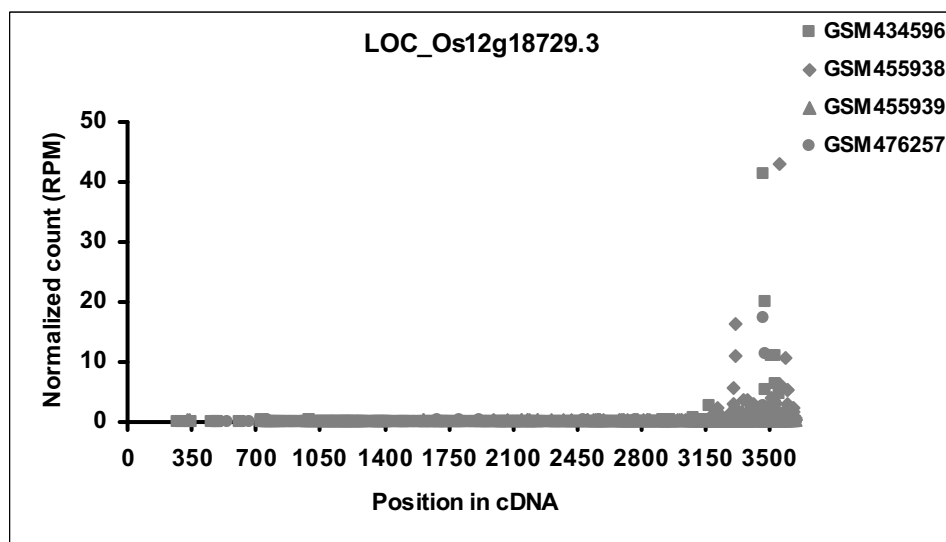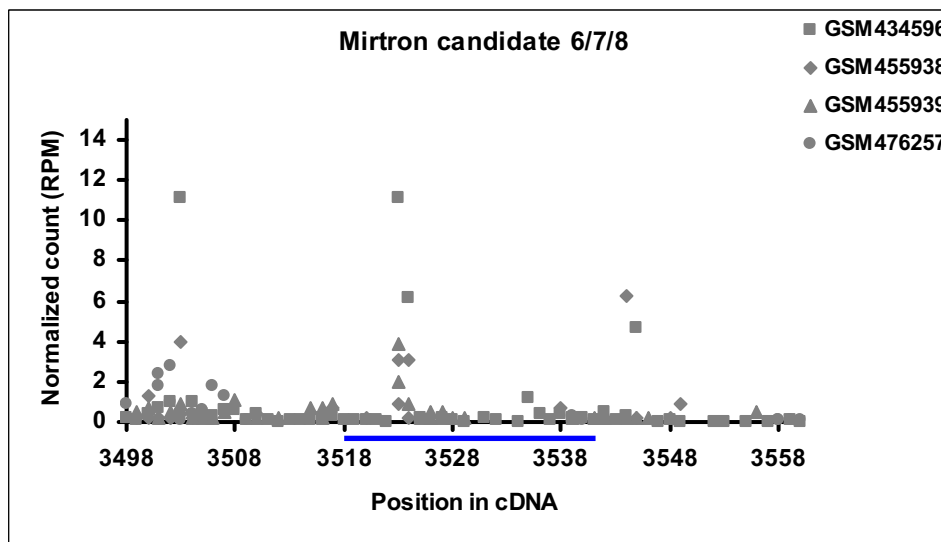

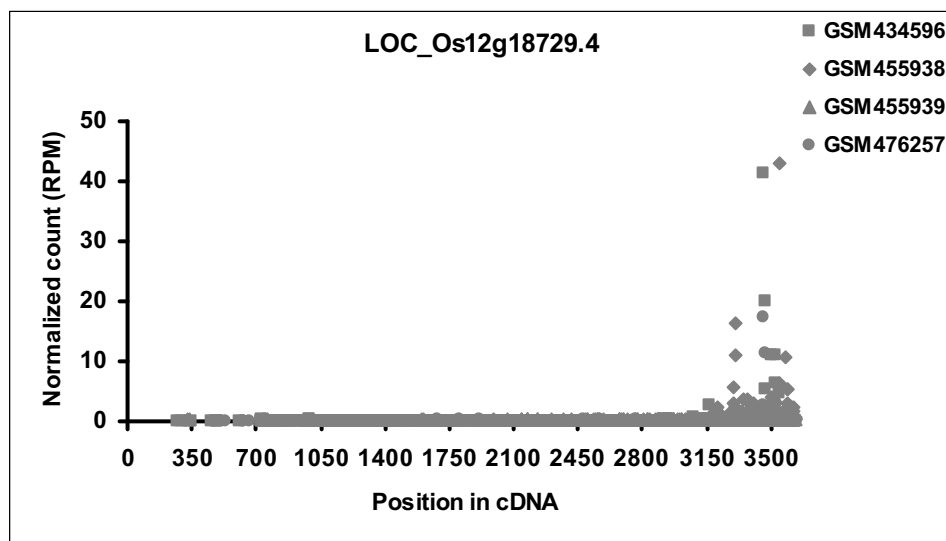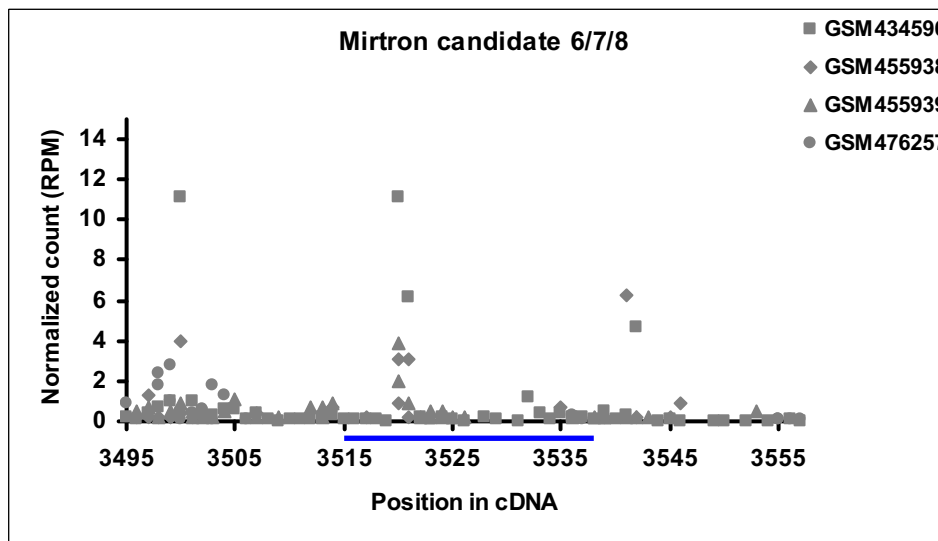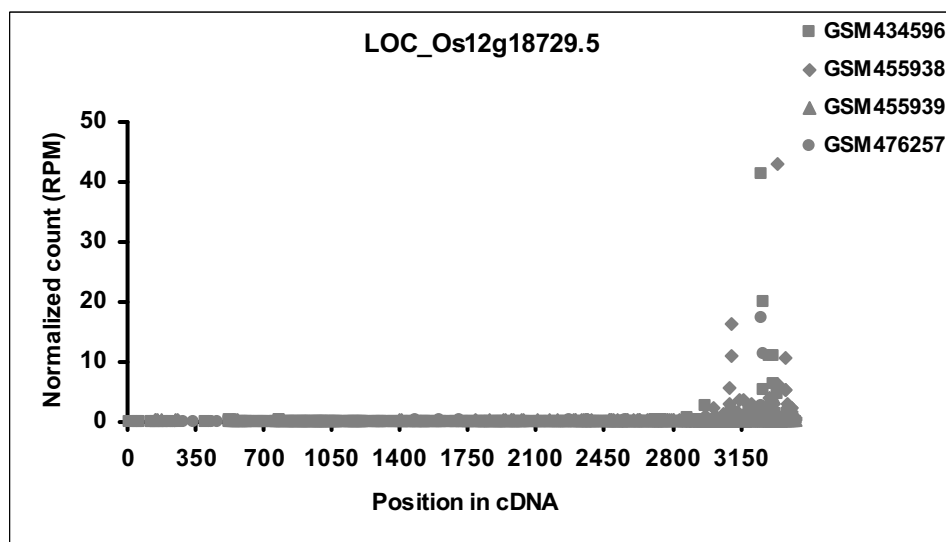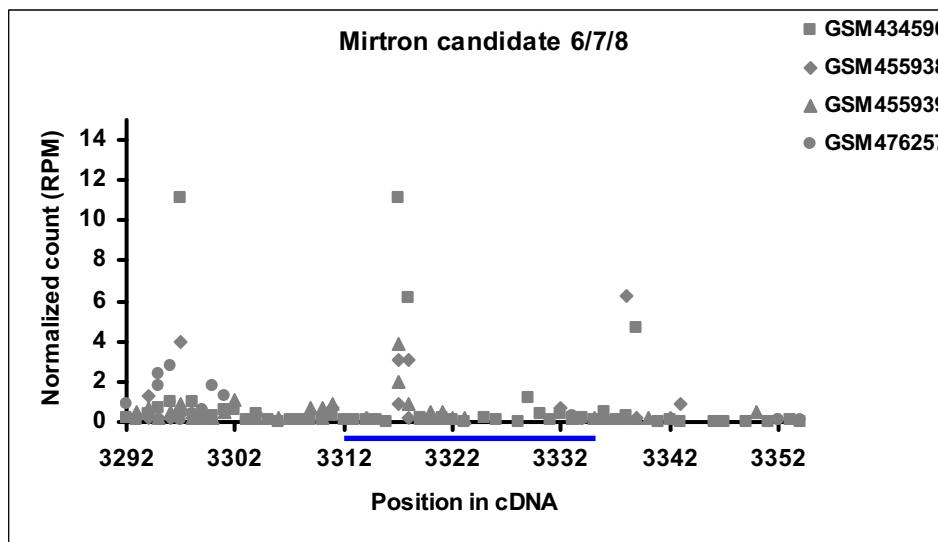

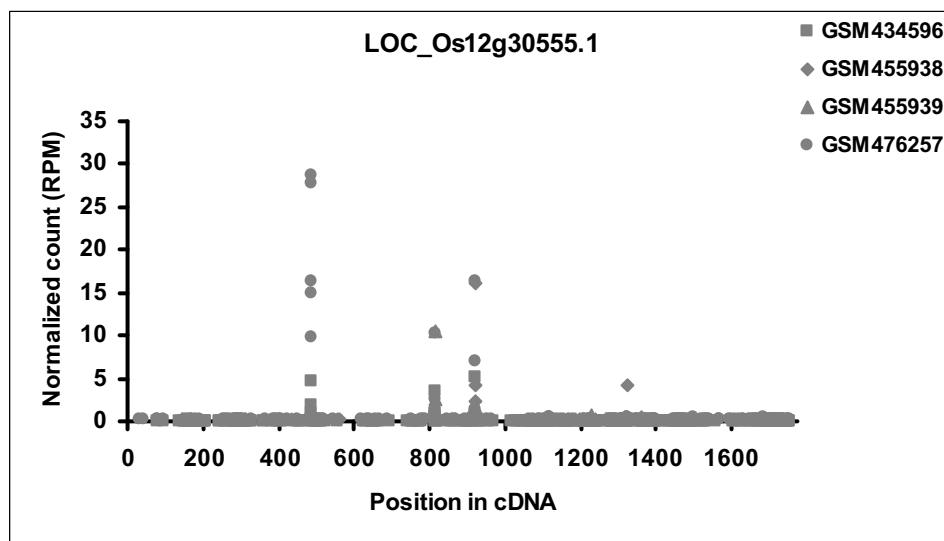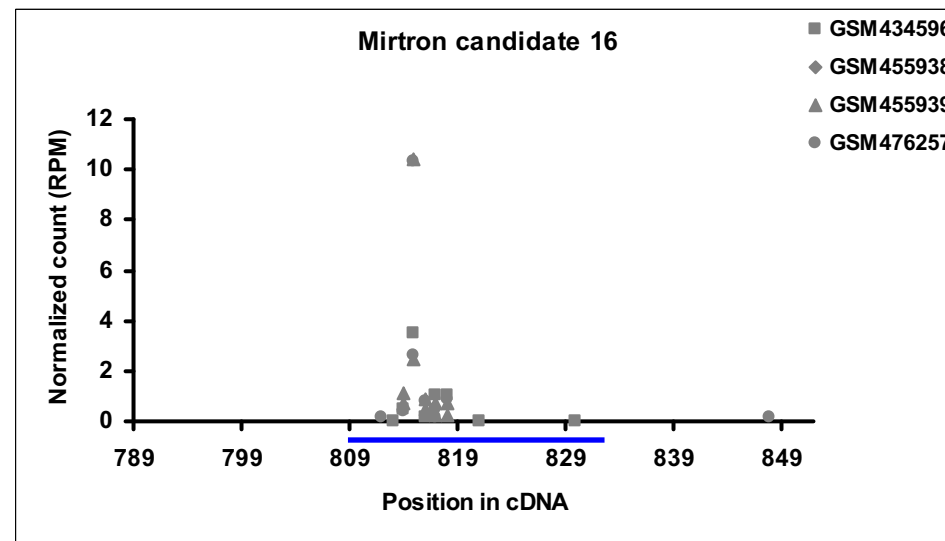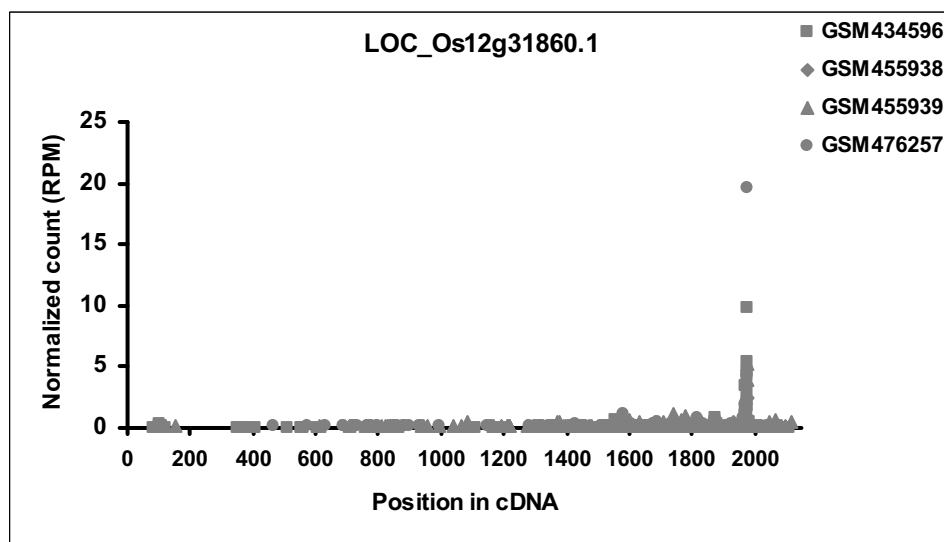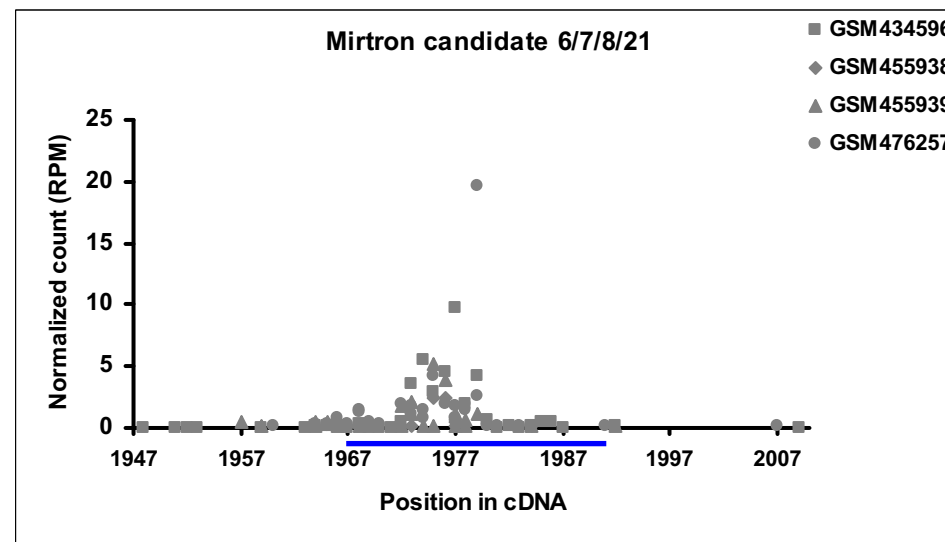

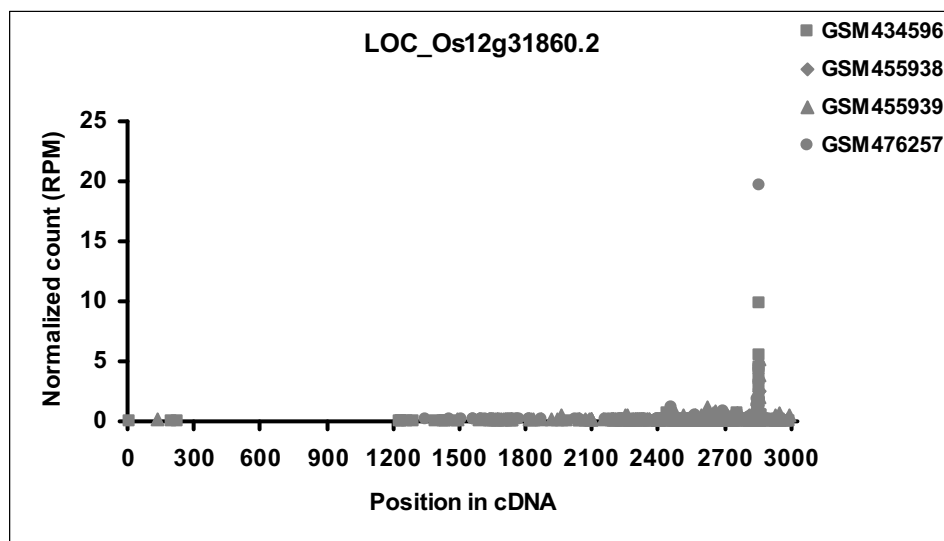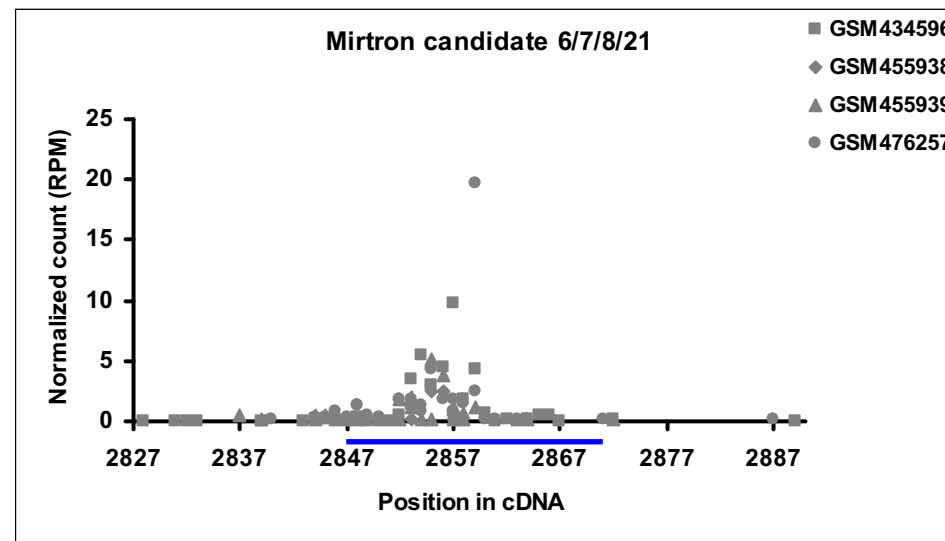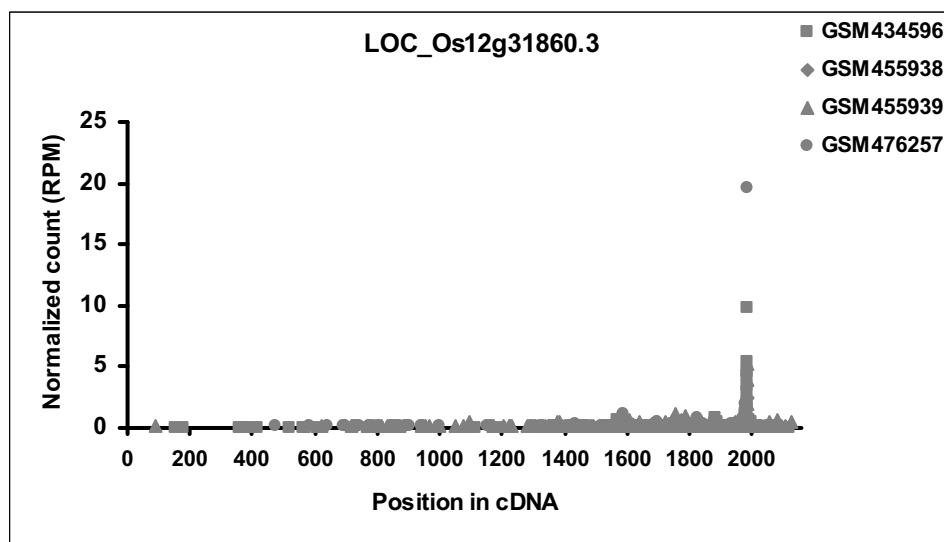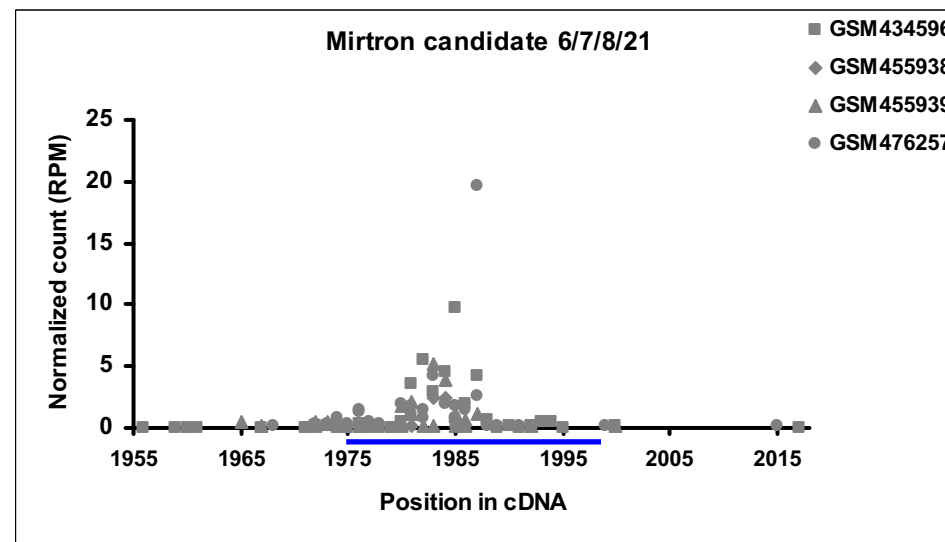

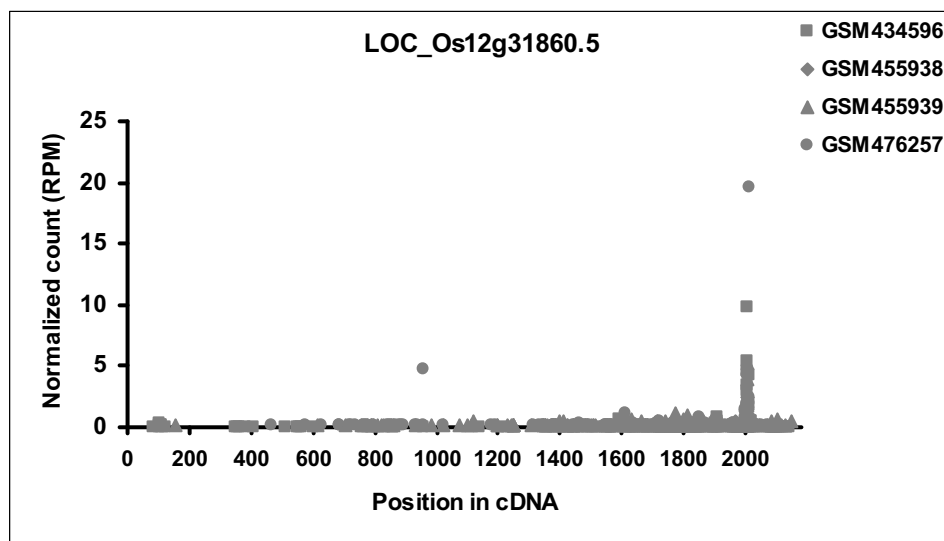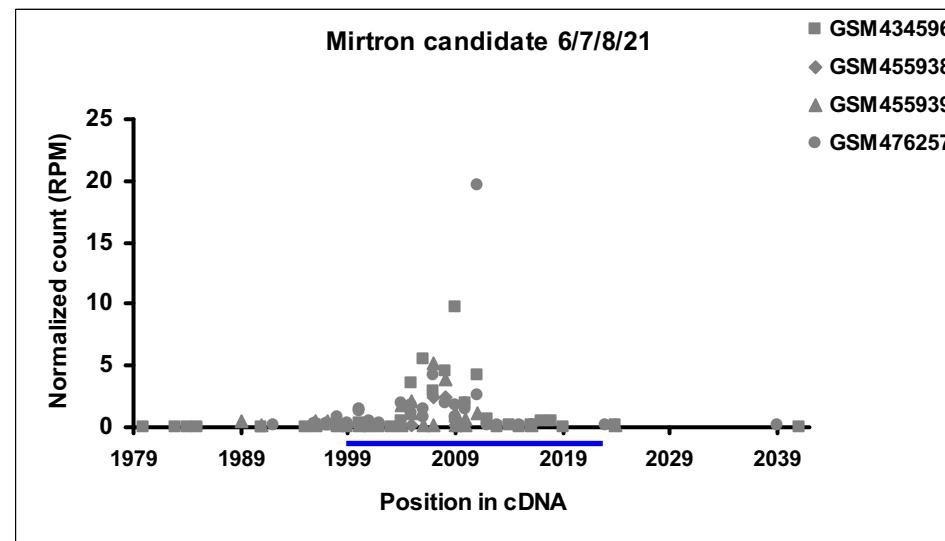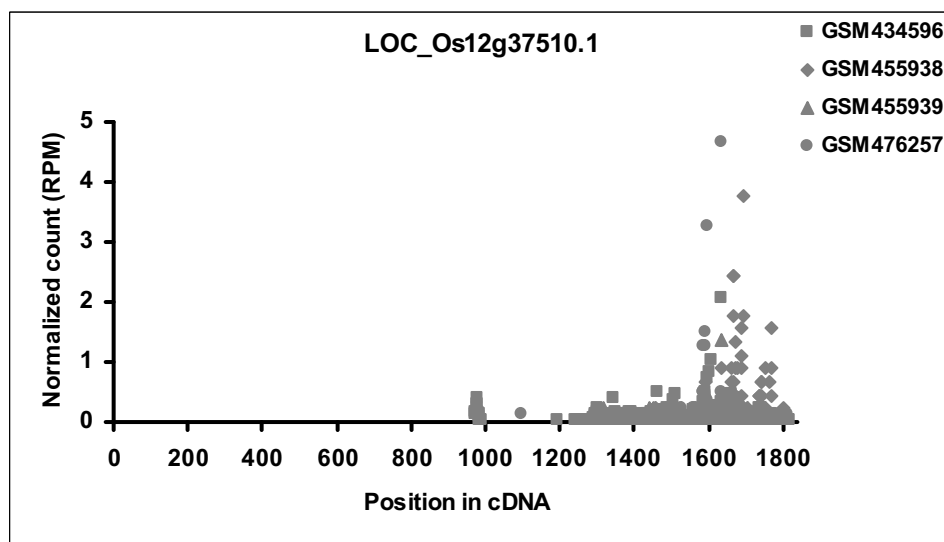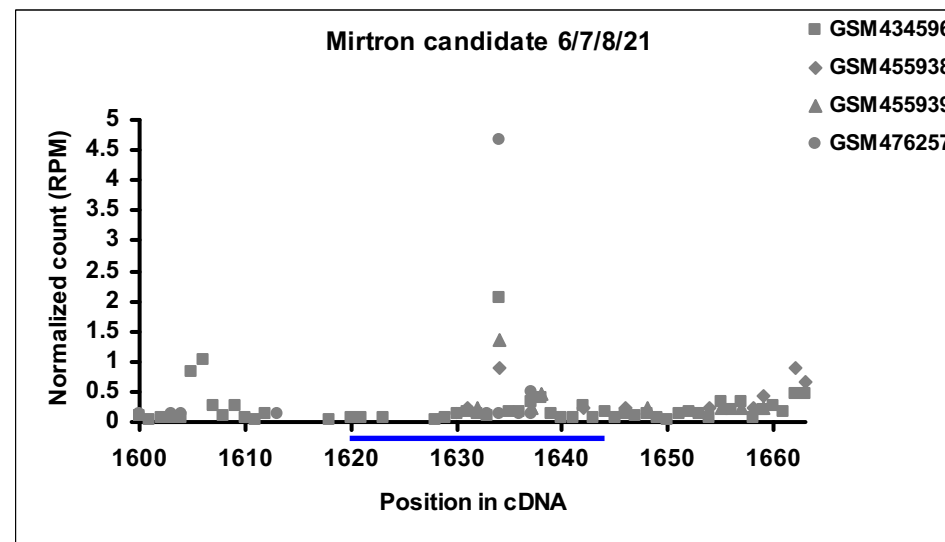

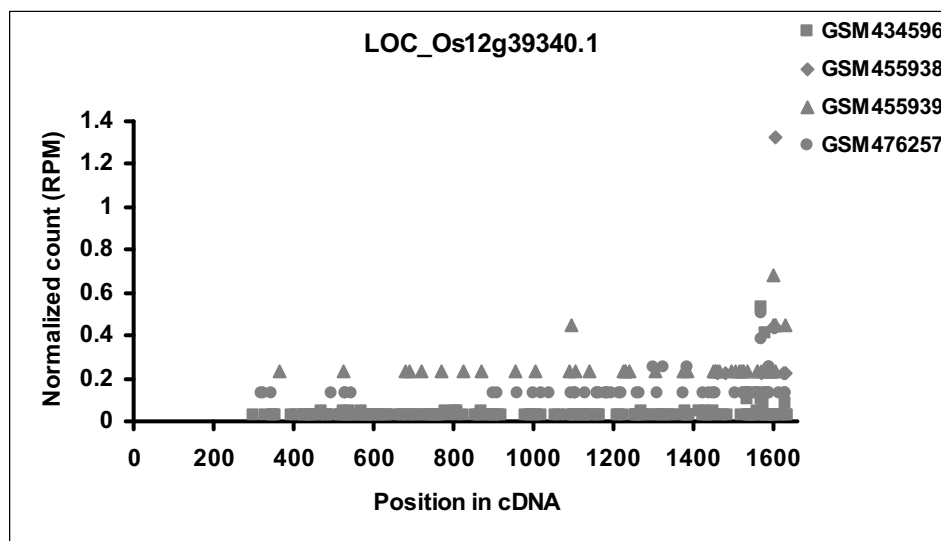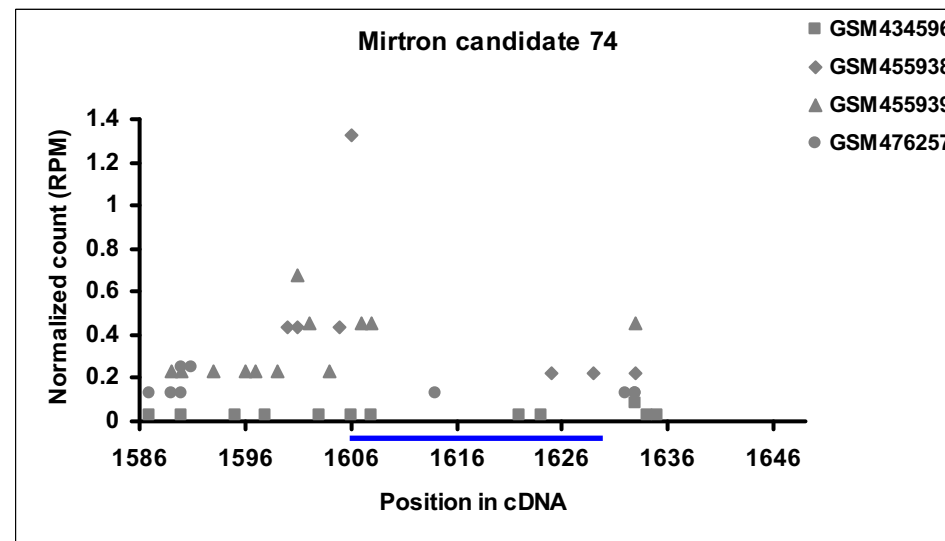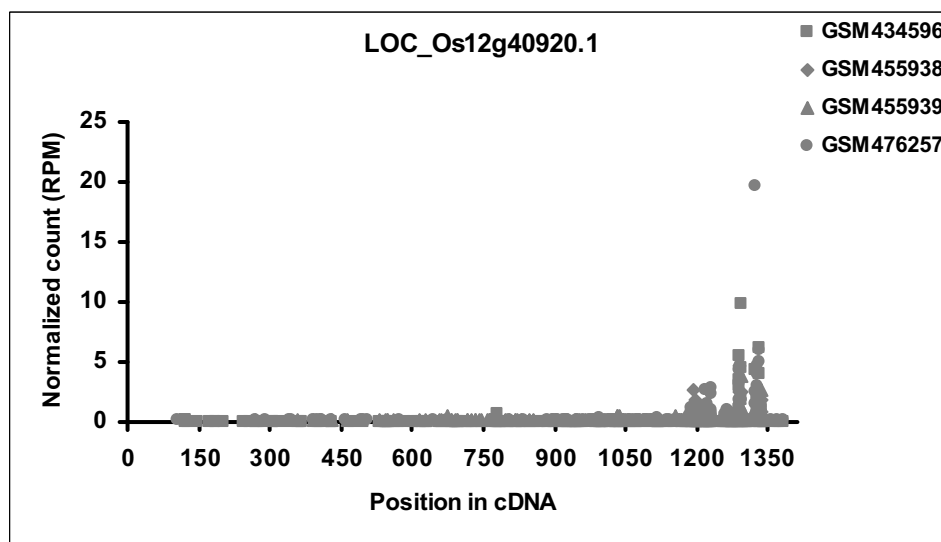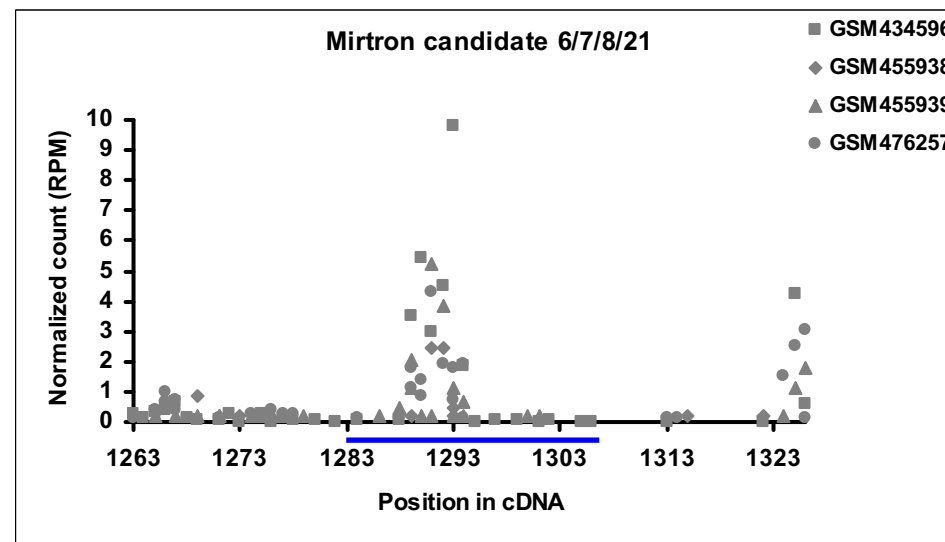

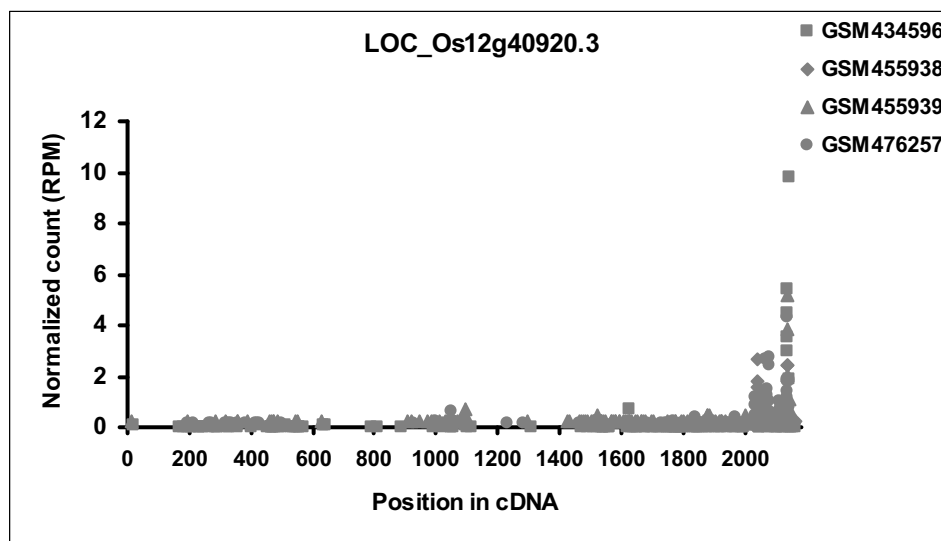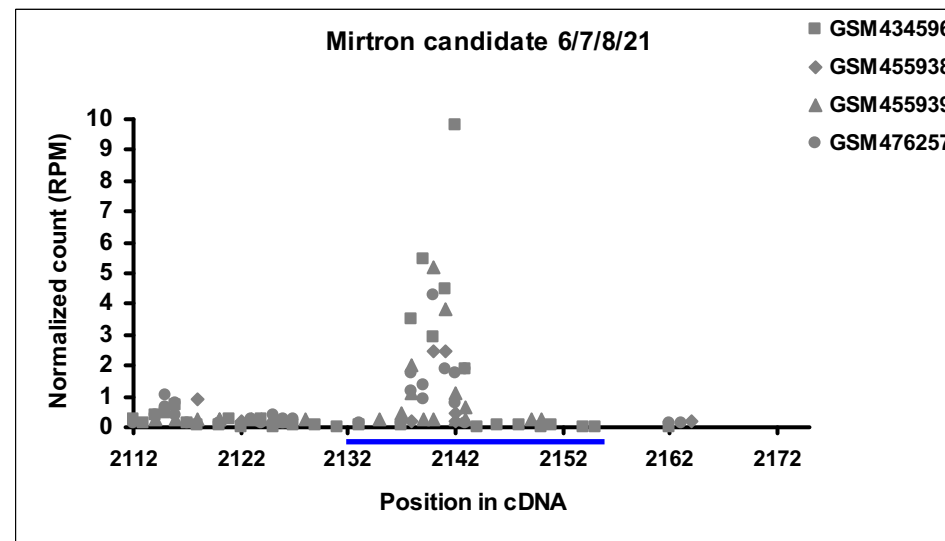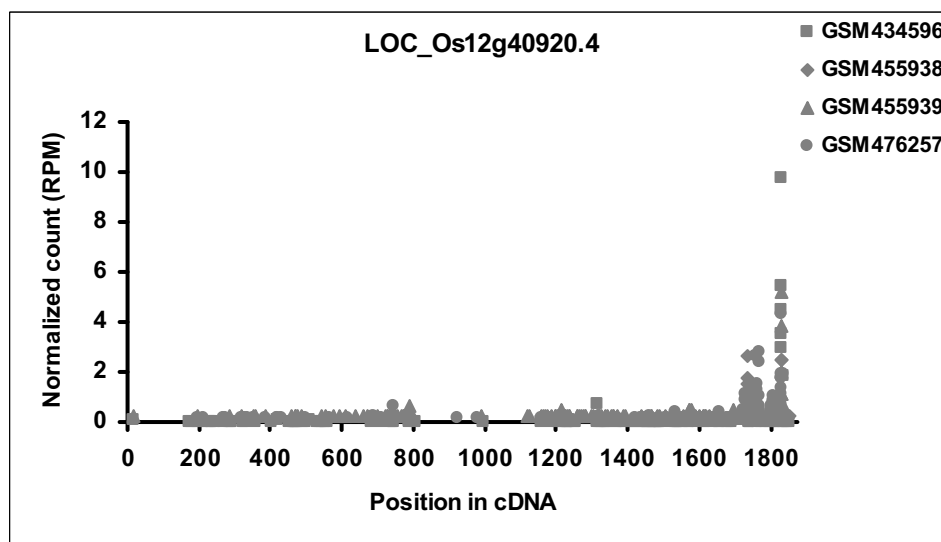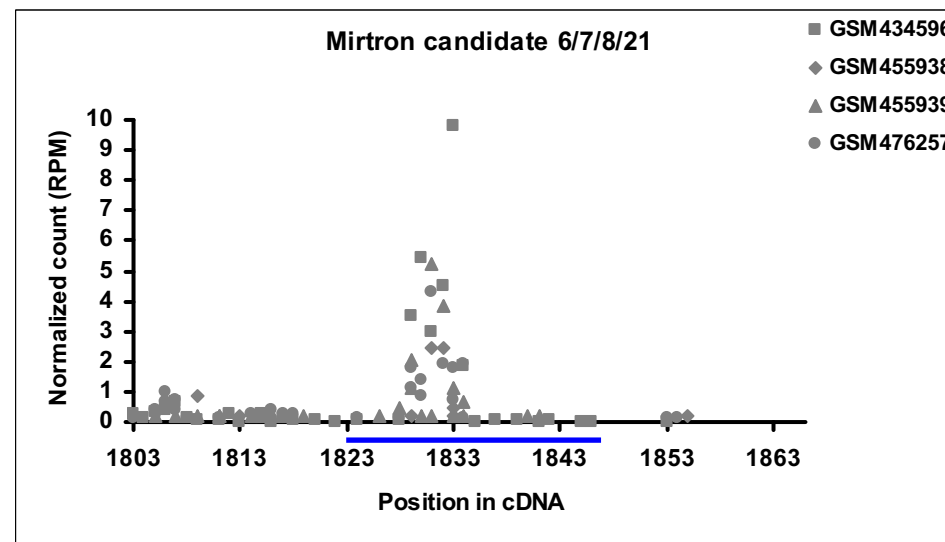

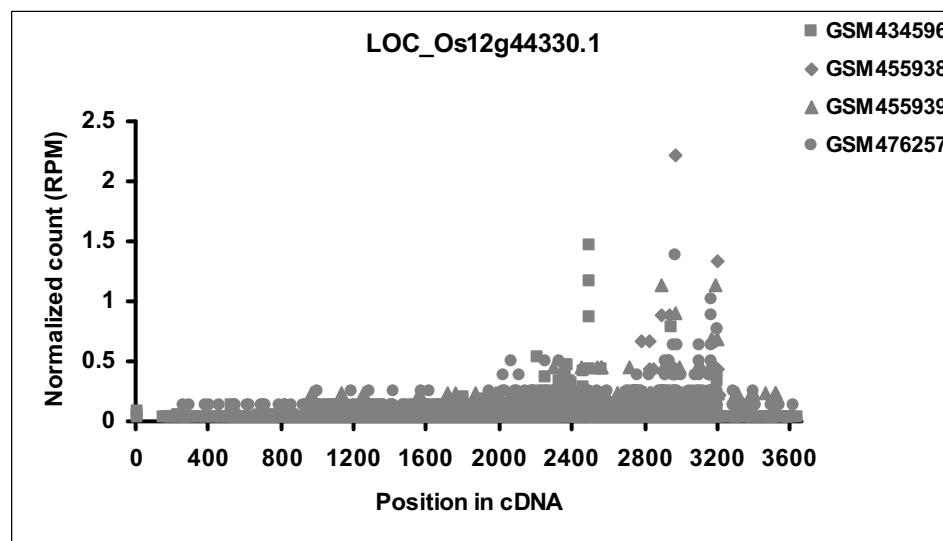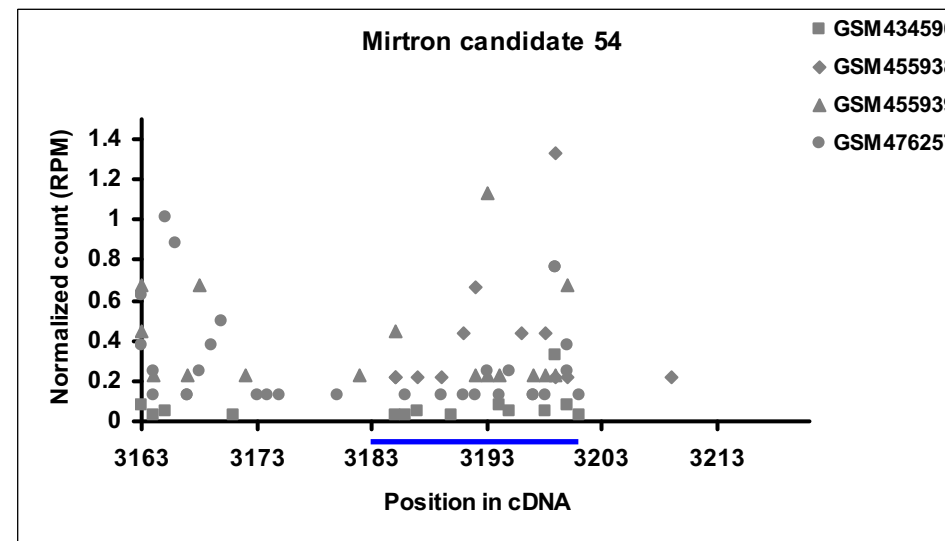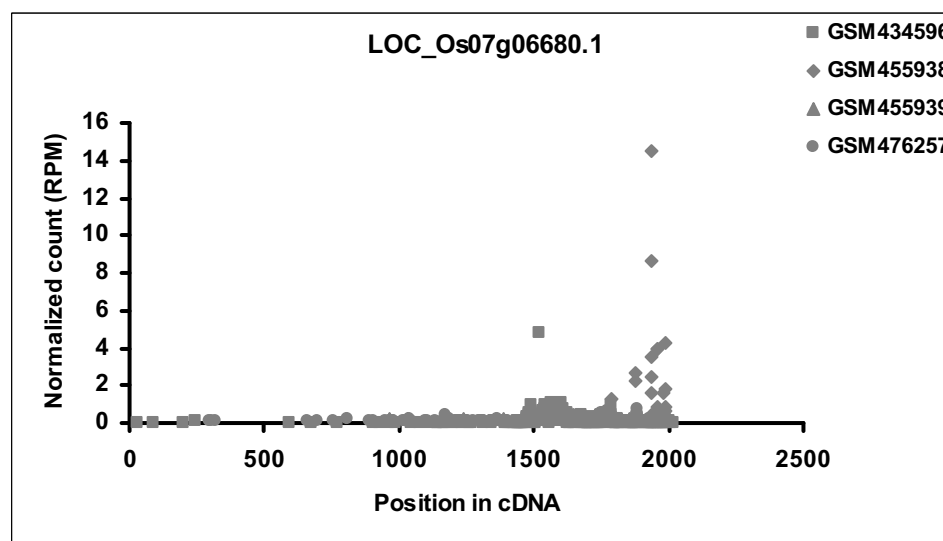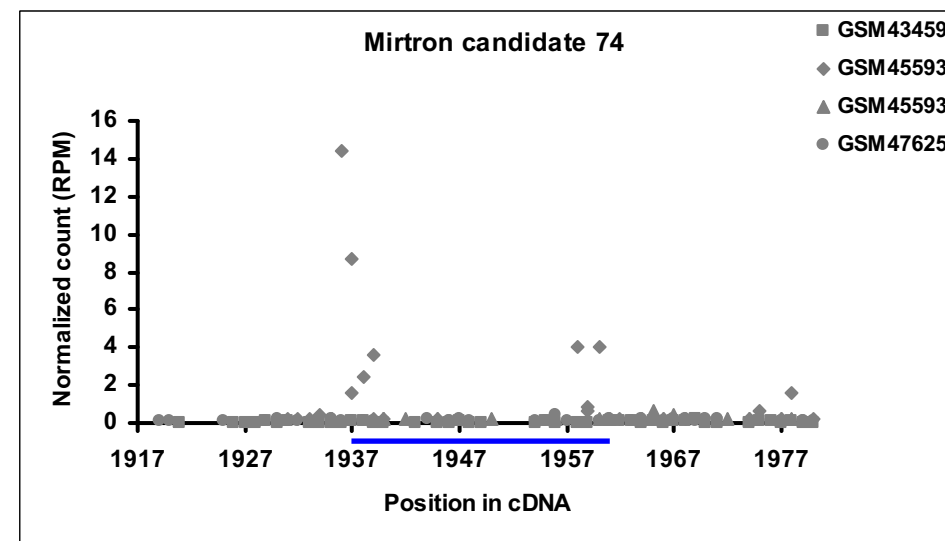

Supplement: Figure S4 — Degradome sequencing data-based identification of the targets of the mature mirtrons in rice. For all the sub-figures, the first panels depict the degradome signals all along the target transcripts, and the other panels provide detailed views of the cleavage signals within the regions surrounding the target recognition sites (denoted by blue horizontal lines). The transcript IDs are shown in the first panels, and the mirtron IDs are listed in the other panels (see Table S3 and S4 for the sequence information corresponding to the mirtron IDs). The x axes measure the positions of the signals along the transcripts, and the y axes measure the signal intensities based on normalized counts (in RPM, reads per million), allowing cross-library comparison. See Table S2 for the degradome data sets used in this analysis. (PDF) [file pone.0031163.s004.pdf]
