# Supplementary material for: Large-Scale Identification of Mirtrons in Arabidopsis and Rice
Source: PLoS One. 2012 Feb 13;7(2):e31163. doi: 10.1371/journal.pone.0031163 (PMC3278437; doi:10.1371/journal.pone.0031163)

AT1G76680.1-2

	GSM366866	GSM304283	GSM366870	GSM304284	Total
GTAAGCACATTTTGGATTTGGA	0.86	0	0	0	0.86
GCACATTTTGGATTTGGA	0	0.68	0	0	0.68
TGTGTCCTTGTTGTTGCAG	0	0	1.74	0	1.74
TGTGTCCTTGTTGTTGCA	0	0	0	3.67	3.67

```
      GTAAGCACATTTTGGATTTGGAATTCAAGAAGCTGAAACCATTCAAGATTCTTATATGTGTCCTTGTTGTTGCAG
-13.10 ....((((....((((.....((..(((((..((((....))))...)))))..))..))))....))).))..  []
```

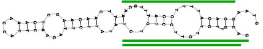

AT2G10921.1-2

Sequence	GSM343001	GSM518432	GSM442934	GSM342999	GSM456945	GSM442932	GSM257235	GSM442935	GSM253623	GSM442933	GSM343002	GSM366870	GSM343005	GSM343004	GSM642338	GSM366865	GSM366868	GSM304285	GSM304284	GSM304282	GSM154336	GSM343000	Total
ATCGAATCTCCTGTTATTATGAAC	0.81	0	0.26	0	0	0	0	0	0	0	0	0	0	0	0	0	0	0	0	0	0	0	1.07
ATCGAATCTCCTGTTATTATGA	0	0.24	0	0	0	0	0	0	0	0	0	0	0	0	0	0	0	0	0	0	0	0	0.24
CTCCTGTTATTATGAACATGGTAT	1.21	0	0	0.38	0	0	0	0	0	0	0	0	0	0	0	0	0	0	0	0	0	0	1.59
CTCCTGTTATTATGAACATGGTA	0	0	0	0	0.15	0	0	0	0	0	0	0	0	0	0	0	0	0	0	0	0	0	0.15
TCCTGTTATTATGAACATGGTATA	0	0	0	0	0	0.28	0	0	0	0	0	0	0	0	0	0	0	0	0	0	0	0	0.28
CCTGTTATTATGAACATGGTATA	0	0	0	0	0.15	0	0	0	0	0	0	0	0	0	0	0	0	0	0	0	0	0	0.15
ATTATGAACATGGTATATAGAATG	0	0	0.26	0	0	0	0	0	0	0	0	0	0	0	0	0	0	0	0	0	0	0	0.26
ATTATGAACATGGTATATAGAAT	0	0	0	0	0.15	0	0	0	0	0	0	0	0	0	0	0	0	0	0	0	0	0	0.15
ATGAACATGGTATATAGAATGGA	0	0	0	0	0	0.55	0	0	0	0	0	0	0	0	0	0	0	0	0	0	0	0	0.55
ATGAACATGGTATATAGAATGGAT	0	0	0.26	0	0	0.28	13.31	0	0	0	0	0	0	0	0	0	0	0	0	0	0	0	13.85
TGAACATGGTATATAGAATGGATA	0	0	0	0	0	0	0	0.27	0	0	0	0	0	0	0	0	0	0	0	0	0	0	0.27
TGAACATGGTATATAGAATGGA	0	0	0	0	0	0	0	0.27	0	0	0	0	0	0	0	0	0	0	0	0	0	0	0.27
GAACATGGTATATAGAATGG	0	0	0.26	0	0	0	0	0.27	0	0	0	0	0	0	0	0	0	0	0	0	0	0	0.53
GAACATGGTATATAGAATGGATA	0	0	0.26	0	0	0.55	0	0	0	0	0	0	0	0	0	0	0	0	0	0	0	0	0.81
GAACATGGTATATAGAATGGATAT	0	0	0.26	0	0	0	0	0	0	0	0	0	0	0	0	0	0	0	0	0	0	0	0.26
ACATGGTATATAGAATGGATATG	0	0	0.53	0	0	0	0	0	0	0.29	0	0	0	0	0	0	0	0	0	0	0	0	0.82
ACATGGTATATAGAATGGATA	0	0	0	0	0	0.28	0	0.54	7.32	0	0	0	0	0	0	0	0	0	0	0	0	0	8.14
ACATGGTATATAGAATGGATATGA	0	0	0.26	0	0	0	0	0	0	0	0	0	0	0	0	0	0	0	0	0	0	0	0.26
CATGGTATATAGAATGGATATGAA	0	0	0.26	0	0	0	0	0	0	0	0	0	0	0	0	0	0	0	0	0	0	0	0.26
ATGGTATATAGAATGGATATGAAT	0	0	0.26	0.38	0.15	0.28	0	0.27	0	0	0.28	0	0	0	0	0	0	0	0	0	0	0	1.62
ATGGTATATAGAATGGATA	0	0	0.26	0	0	0	0	0	0	0	0	0	0	0	0	0	0	0	0	0	0	0	0.26
ATGGTATATAGAATGGATATGAA	0	0	0	0	0	0	0	0.27	0	0	0	0	0	0	0	0	0	0	0	0	0	0	0.27
TGGTATATAGAATGGATATGAA	0	0	0.26	0	0	0	0	0	0	0	0	0	0	0	0	0	0	0	0	0	0	0	0.26
ATATAGAATGGATATGAATCA	0	0	0.26	0	0	0	26.63	0	0	0	0	0	0	0	0	0	0	0	0	0	0	0	26.89
ATATAGAATGGATATGAATCAATA	0	0	0.26	0	0	0.28	26.63	0.54	0	0	0	0	0	0	0	0	0	0	0	0	0	0	27.71
TATAGAATGGATATGAATCAATA	0	0	0	0	0	0	13.31	0	0	0	0	0	0	0	0	0	0	0	0	0	0	0	13.31
AGAATGGATATGAATCAATAA	0	0	0.26	0	0	0	0	0	0	0	0	0	0	0	0	0	0	0	0	0	0	0	0.26
AATGGATATGAATCAATAAAAGGT	0	0	0	0	0	0	13.31	0	0	0	0	0	0	0	0	0	0	0	0	0	0	0	13.31
AAAGGTTTCTGAATGGATAT	0	0	0	0	0	0	0	0	7.32	0	0	0	0	0	0	0	0	0	0	0	0	0	7.32
AAAGGTTTCTGAATGGATATA	0	0	0.26	0	0	0	0	0	0	0	0	0	0	0	0	0	0	0	0	0	0	0	0.26
AAAGGTTTCTGAATGGATATATA	0	0	0.26	0	0	0	0	0.54	0	0.29	0	0	0	0	0	0	0	0	0	0	0	0	1.09
AAGGTTTCTGAATGGATA	0	0	0	0	0	0	0	0	0	0	0	1.74	0	0	0	0	0	0	0	0	0	0	1.74
AAGGTTTCTGAATGGATATA	0	0	0	0	0	0	0	0	0	0	0	0	0.36	0	0	0	0	0	0	0	0	0	0.36
AGGTTTCTGAATGGATAT	0	0	0	0	0	0.28	0	0	0	0	0	0	0.36	0	0	0	0	0	0	0	0	0	0.64
GGTTTCTGAATGGATATATATAA	0	0	0	0	0	0	0	0	0	0.29	0	0	0	0	0	0	0	0	0	0	0	0	0.29
ATATAAATAGACTCGTTATTATGG	0	0	0.26	0	0	0	0	0	0	0	0	0	0	0	0	0	0	0	0	0	0	0	0.26
ATAAATAGACTCGTTATTATGGAC	0	0	0.26	0	0	0	0	0.27	0	0	0	0	0.36	0.3	0	0	0	0	0	0	0	0	1.19
AAATAGACTCGTTATTATGGACAT	0	0	0	0	0	0	0	0.27	0	0	0	0	0	0	0	0	0	0	0	0	0	0	0.27
AAATAGACTCGTTATTATGGA	0	0	0	0	0	0	0	0	0	0	0	0	0.36	0	0	0	0	0	0	0	0	0	0.36
AAATAGACTCGTTATTATGGACA	0	0	0.53	0	0	0.55	0	0.27	0	0.29	0	0	0	0	0	0	0	0	0	0	0	0	1.64
AATAGACTCGTTATTATGGACATC	0	0	0.26	0	0	0.28	0	0	0	0	0.28	0	0.36	0	0	0	0	0	0	0	0	0	1.18
ATAGACTCGTTATTATGGACATC	0	0	0	0	0	0	0	0	0	0	0	0	0	0	0.1	0	0	0	0	0	0	0	0.1
TAGACTCGTTATTATGGACATCA	0	0	0.53	0	0.15	0.28	0	0.27	0	0.87	0	0	0.72	0	0	0	0	0	0	0	0	0	2.82
AGACTCGTTATTATGGACATCAAA	0	0	0	0	0	0	0	0.54	0	0	0.57	0	0	0	0	0	0	0	0	0	0	0	1.11
AGACTCGTTATTATGGACATC	0	0	0	0	0.15	0	0	0	0	0	0	0	0	0	0	0	0	0	0	0	0	0	0.15
ATGGACATCAAAAAATTGTTTGTT	0	0	0	0	0	0.28	0	0	0	0	0	0	0	0	0	0	0	0	0	0	0	0	0.28
GGACATCAAAAAATTGTTTGTTGA	0	0	0	0	0	0	0	0	0	0	0	0	0	0.6	0	0	0	0	0	0	0	0	0.6
CAAAAAATTGTTTGTTGATTTTT	0	0	0	0	0	0	0	0	0	0	0	0	0	0	0	0.58	0	0	0	0	0	0	0.58
AAATTGTTTGTTGATTTTT	0	0	0	0	0	0	0	0	0	0	0	0	0	0	0	0	1.44	0	0	0	0	0	1.44
AATTGTTTGTTGATTTTTCTT	0	0	0	0	0	0	0	0	0	0	0	0	0	0	0	0	0	1.22	0	0	0	0	1.22
ATTGTTTGTTGATTTTTCTTT	0	0	0	0	0	0	0	0	0	0	0	0	0	0.3	0	0	0	0	0	0	0	0	0.3
ATTGTTTGTTGATTTTTC	0	0	0	0	0	0	0	0	0	0	0	0	0.36	0	0	0	0	0	0	0	0	0	0.36
TGTTTGTTGATTTTTCTTTCA	0	0	0	0	0	0	0	0	0	0	0	0	0	0	0	0	0	0	3.67	0	0	0	3.67
TTGTTGATTTTTCTTTCAGTTT	0	0	0	0	0	0	0	0	0	0	0	0	0	0	0	0	0	0	0	1.46	0	0	1.46
TTGTTGATTTTTCTTTCAGTTTGC	0	0	0	0.38	0	0	0	0	0	0	0.28	0	0	0	0	0.58	0	0	0	0	0	0	1.24
TGTTGATTTTTCTTTCAGTTTGCA	0	0.48	0	0.76	0	0	0	0	0	0	0	0	0.36	0	0	0	0	0	0	0	0	0	1.6
GTTGATTTTTCTTTCAGTTTGCAG	0	0	0	0	0	0	0	0	0	0	0	0	0	0	0	0	0	0	0	0	12.72	0	12.72
GTTGATTTTTCTTTCAGTTTGCA	0	0	0	0	0	0	0	0	0	0	0	0	0	0	0	0	0	0	0	0	0	0.81	0.81

GTTAATCGAATCTCCTGTTATTATGAACATGGTATATAGAATGGATATGAATCAATAAAAGGTTTCTGAATGGATATATATAAATAGACTCGTTATTATGGACATCAAAAAATTGTTTGTTGATTTTTCT

-20.40 ..............................(((.((.(((.(((...((((((((((..((((.((.(((..((((.((((.........))))))))..)))))..))))..))))))))))).)))

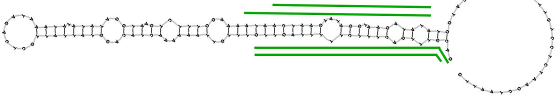

AT3G60950.1-15

Sequence	GSM343002	GSM366867	GSM518432	GSM342999	GSM343004	GSM442934	GSM343005	GSM304283	GSM442935	GSM642338	GSM642337	GSM366866	GSM442933	GSM642335	GSM366865	Total
GTTCATTGTTTAAACATGTGGGAC	0.28	1.14	0	0	0	0	0	0	0	0	0	0	0	0	0	1.42
TTCATTGTTTAAACATGTGGGAC	0	0	0.24	0.38	0	0	0	0	0	0	0	0	0	0	0	0.62
TCATTGTTTAAACATGTGGGACA	0	0	0	0	0.91	0	0	0	0	0	0	0	0	0	0	0.91
ATTGTTTAAACATGTGGGACATTT	0	0	0	0	0	0.53	0	0	0	0	0	0	0	0	0	0.53
ATTGTTTAAACATGTGGGAC	0	0	0	0.38	0	0	0.36	0	0	0	0	0	0	0	0	0.74
ATTGTTTAAACATGTGGGACA	0	0	0	0	0	0	0	0.68	0	0	0	0	0	0	0	0.68
AAACATGTGGGACATTTTTGT	0	0	0	0	0	0	0	0	0.27	0	0	0	0	0	0	0.27
ACATGTGGGACATTTTTGTATTT	0	0	0	0	0	0	0	0	0	0.15	0	0	0	0	0	0.15
ATGTGGGACATTTTTGTATTTGT	0	0	0	0	0	0	0	0	0	0	0.13	0	0	0	0	0.13
TTGTGTTACAATTGTTTTGGTTA	0.28	0	0	0	0	0	0	0	0	0	0	0	0	0	0	0.28
GTGTTACAATTGTTTTGGTTA	0	0	0	0	0	0	0	0	0	0	0	0.43	0	0	0	0.43
GTTTTGGTTATAGAAAAGTTGG	0	0	0	0	0	0	0	0	0	0	0	0	0.29	0	0	0.29
TTTGGTTATAGAAAAGTTGGGCTC	0	0	0	0	0	0	0	0	0	0	0	0	0	0.76	0	0.76
ATAGAAAAGTTGGGCTCCCTGTGA	0	0	0	0	0	0	0	0.68	0	0	0	0	0	0	0.58	1.26

GTTCATTGTTTAAACATGTGGGACATTTTTGTATTTGTGTTACAATTGTTTTGGTTATAGAAAAGTTGGGCTCCCTGTGAAAG

-11.00 ((((...((((((....(((((...(((((.......))))).)))))).))).))))).....))))).......... []

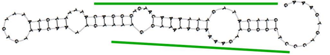

AT4G05280.1-2

Sequence	GSM442935	GSM442934	GSM304282	GSM642338	GSM343004	GSM456945	GSM253624	GSM304284	GSM257237	GSM343000	GSM343002	GSM642337	GSM442933	GSM366868	GSM518432	GSM442932	GSM342999	GSM366865	GSM304283	GSM366870	GSM253622	Total
AGGAGGAGGAAAGCATGGAGGG	0.54	0	0	0	0	0	0	0	0	0	0	0	0	0	0	0	0	0	0	0	0	0.54
AGGAGGAGGAAAGCATGGAGGGGT	0.54	0	0	0	0	0	0	0	0	0	0	0	0	0	0	0	0	0	0	0	0	0.54
GAGGAGGAAAGCATGGAGGGGTG	0	0.26	0	0	0	0	0	0	0	0	0	0	0	0	0	0	0	0	0	0	0	0.26
AGGAGGAAAGCATGGAGGGGTGGA	0	0	1.46	0	0	0	0	0	0	0	0	0	0	0	0	0	0	0	0	0	0	1.46
GAGGAAAGCATGGAGGGGTGGAAG	0	0.26	0	0	0	0	0	0	0	0	0	0	0	0	0	0	0	0	0	0	0	0.26
AGGAAAGCATGGAGGGGTGGA	0	0	0	0.1	0	0	0	0	0	0	0	0	0	0	0	0	0	0	0	0	0	0.1
AAAGCATGGAGGGGTGGAAGCA	0	0	1.46	0	0	0	0	0	0	0	0	0	0	0	0	0	0	0	0	0	0	1.46
AAAGCATGGAGGGGTGGAAGCAA	0	0.26	0	0	0	0	0	0	0	0	0	0	0	0	0	0	0	0	0	0	0	0.26
AAAGCATGGAGGGGTGGAAGCAAC	0.27	0	0	0	0	0	0	0	0	0	0	0	0	0	0	0	0	0	0	0	0	0.27
AAGCATGGAGGGGTGGAAGCAACT	0.54	0	0	0.05	0.3	0.3	2.81	0	0	0	0	0	0	0	0	0	0	0	0	0	0	4
AAGCATGGAGGGGTGGAAG	0.27	0	0	0	0	0	0	0	0	0	0	0	0	0	0	0	0	0	0	0	0	0.27
AAGCATGGAGGGGTGGAAGCAA	0.27	0	0	0	0	0	0	0	0	0	0	0	0	0	0	0	0	0	0	0	0	0.27
AAGCATGGAGGGGTGGAAGCA	0.27	0	0	0	0	0	0	0	0	0	0	0	0	0	0	0	0	0	0	0	0	0.27
AAGCATGGAGGGGTGGAAGCAAC	0	0.26	0	0	0	0	0	0	0	0	0	0	0	0	0	0	0	0	0	0	0	0.26
AGCATGGAGGGGTGGAAGCAACT	0.27	0	0	0	0	0	0	3.67	0	0	0	0	0	0	0	0	0	0	0	0	0	3.94
AGCATGGAGGGGTGGAAGCAAC	0.27	0	0	0	0	0	0	0	0	0	0	0	0	0	0	0	0	0	0	0	0	0.27
AGCATGGAGGGGTGGAAGCAA	0	0.26	0	0	0	0	0	0	0	0	0	0	0	0	0	0	0	0	0	0	0	0.26
CATGGAGGGGTGGAAGCAACTGGC	0	0	0	0	0	0.15	0	0	0	0	0	0	0	0	0	0	0	0	0	0	0	0.15
ATGGAGGGGTGGAAGCAACTGGC	0	0	0	0	0	0.15	0	0	18.72	0	0	0	0	0	0	0	0	0	0	0	0	18.87
ATGGAGGGGTGGAAGCAACTGGCA	0	0	0	0	0	0.15	0	0	0	0.81	0	0	0	0	0	0	0	0	0	0	0	0.96
ATGGAGGGGTGGAAGCAACTG	0	0	0	0.15	0	0	0	0	0	0	0	0	0	0	0	0	0	0	0	0	0	0.15
TGGAGGGGTGGAAGCAACTGGC	0	0	0	0	0	0	0	0	0	0	0.28	0	0	0	0	0	0	0	0	0	0	0.28
TGGAGGGGTGGAAGCAACTGGCA	0	0.53	0	0	0	0.15	0	0	0	0	0	0	0	0	0	0	0	0	0	0	0	0.68
TGGAGGGGTGGAAGCAACTGGCAC	0	0	0	0	0.3	0.89	0	0	0	0	0	0	0	0	0	0	0	0	0	0	0	1.19
GGAGGGGTGGAAGCAACTGGCA	0	0.26	0	0	0	0	0	0	0	0	0	0	0	0	0	0	0	0	0	0	0	0.26
GAGGGGTGGAAGCAACTGGCA	0	0	0	0	0	0.3	0	0	0	0	0	0	0	0	0	0	0	0	0	0	0	0.3
GAGGGGTGGAAGCAACTGGCAC	0	0	0	0	0	0.15	0	0	0	0	0	0	0	0	0	0	0	0	0	0	0	0.15
GAGGGGTGGAAGCAACTGGCACTA	0	0	0	0	0	0.15	0	0	0	0	0	0	0	0	0	0	0	0	0	0	0	0.15
AGGGGTGGAAGCAACTGGCAC	0	0	0	0	0	0	0	0	0	0	0	0.13	0	1.44	0	0	0	0	0	0	0	1.57
AGGGGTGGAAGCAACTGGCACTAT	0	0	0	0	0	0.45	0	0	0	0	0	0	0	0	0	0	0	0	0	0	0	0.45
AGGGGTGGAAGCAACTGGCACTA	0	0	0	0	0	0.15	0	0	0	0	0	0	0.29	0	0	0	0	0	0	0	0	0.44
GGGGTGGAAGCAACTGGCA	0	0	0	0	0	0.15	0	0	0	0	0	0	0	0	0	0	0	0	0	0	0	0.15
GGGTGGAAGCAACTGGCACTATCT	0.27	0.26	0	0	0	0.6	0	0	0	0	0	0	0	0	0	0	0	0	0	0	0	1.13
GGGTGGAAGCAACTGGCAC	0	0	0	0	0	0.15	0	0	0	0	0	0	0	0	0	0	0	0	0	0	0	0.15
GGGTGGAAGCAACTGGCACTATC	0	0	0	0	0	0.3	0	0	0	0	0	0	0	0	0	0	0	0	0	0	0	0.3
GGGTGGAAGCAACTGGCACTAT	0.27	0	0	0	0	0.15	0	0	0	0	0	0	0	0	0	0	0	0	0	0	0	0.42
GGTGGAAGCAACTGGCACTATCTC	0	0	0	0	0	0.15	0	0	0	0	0	0	0	0	0	0	0	0	0	0	0	0.15
GGTGGAAGCAACTGGCACTATC	0	0	0	0	0	0.15	0	0	0	0	0	0	0	0	0	0	0	0	0	0	0	0.15
GGTGGAAGCAACTGGCACTAT	0.27	0	0	0	0	0	0	0	0	0	0	0	0	0	0	0	0	0	0	0	0	0.27
GTGGAAGCAACTGGCACTATCTCT	0	0	0	0	0	0.15	0	0	0	0	0	0	0	0	0	0	0	0	0	0	0	0.15
TGGAAGCAACTGGCACTATCTCTT	0.27	0	0	0	0	0.3	0	0	0	0.81	0	0	0	0	0	0	0	0	0	0	0	1.38
TGGAAGCAACTGGCACTATCTC	0.27	0	0	0	0	0	0	0	0	0	0	0	0	0	0	0	0	0	0	0	0	0.27
GGAAGCAACTGGCACTATCTCTTT	0	0	0	0	0	0.15	0	0	0	0	0	0	0	0	0	0	0	0	0	0	0	0.15
GGAAGCAACTGGCACTATCTCTT	0	0	0	0	0	0.15	0	0	0	0	0	0	0	0	0	0	0	0	0	0	0	0.15
GAAGCAACTGGCACTATCTCTT	0.27	0	0	0	0	0	0	0	0	0	0	0	0	0	0	0	0	0	0	0	0	0.27
GAAGCAACTGGCACTATCTCTTT	0	0	0	0	0	0.15	0	0	0	0	0	0	0	0	0	0	0	0	0	0	0	0.15
TCTTTTTATTTTGGTTGACGGT	0	0	0	0	0	0	0	0	0	0	0	0	0	0	0.48	0	0	0	0	0	0	0.48
CTTTTTATTTTGGTTGACGGTGTG	0	0	0	0	0	0	0	0	0	0	0	0	0	0	0	0.28	0	0	0	0	0	0.28
TTTTATTTTGGTTGACGGTGTG	0	0	0	0	0	0	0	0	0	0	0	0	0	0	0	0	0.76	0	0	0	0	0.76
TTTATTTTGGTTGACGGTGTGGTA	0	0	0	0	0	0	0	0	0	0	0	0	0	1.44	0	0	0	0	0	0	0	1.44
TTATTTTGGTTGACGGTGTGGTA	0	0	0	0	0	0	0	0	0	0	0	0	0	1.44	0	0	0	0.58	0	0	0	2.02
TATTTTGGTTGACGGTGTGGTAGC	0	0	0	0	0	0	0	0	0	0	0	0	0	0	0.48	0	0	0	0	0	0	0.48
ATTTTGGTTGACGGTGTGGTAGCG	0	0	0	0	0	0	2.81	0	0	0	0	0.13	0	0	0	0	0	0	0	0	0	2.94
ATTTTGGTTGACGGTGTGGTA	0	0	0	0.2	0	0	0	0	0	0	0	0	0	1.44	0.72	0	0	0	0.68	1.74	0	4.78
ATTTTGGTTGACGGTGTGGTAGCGG	0	0	0	0.05	0	0	0	0	0	0	0	0	0	0	0	0	0	0	0	0	0	0.05
ATTTTGGTTGACGGTGTGGTAGC	0	0	0	4.45	0	0	0	0	0	0	0	5.91	0	0	0.48	0	0	0	0	0	0	10.84
ATTTTGGTTGACGGTGTGGTAG	0	0	0	1.1	0	0	0	0	0	0	0	0.53	0	0	0	0	0	0	0	0	0	1.63
ATTTTGGTTGACGGTGTGGT	0	0	0	0.35	0	0	0	0	0	0	0	0	0	0	0	0	0	0	0	0	0	0.35
TTTTGGTTGACGGTGTGGTAGC	0	0	0	0	0	0	0	0	0	0.41	0	0	0	0	0	0	0	0	0	0	0	0.41
TTTGGTTGACGGTGTGGTAGC	0	0	0	0	0	0	0	0	0	0	0	0	0	0	0	0	0	0	0	0	1.19	1.19
TTTGGTTGACGGTGTGGTAGCGGC	0	0	0	0	0.3	0	0	0	0	0	0	0	0	0	0	0	0	0	0	0	0	0.3
TTGGTTGACGGTGTGGTAGCG	0.27	0	0	0	0	0	0	0	0	0	0	0	0	0	0	0	0	0	0	0	0	0.27
TTGGTTGACGGTGTGGTAGCGG	0	0	0	0	0	0	0	0	0	0	0	0	0	0	0	0	0	0	0	0	0.59	0.59
TTGGTTGACGGTGTGGTAGCGGCC	0	0	0	0	0	0.15	0	0	0	0	0	0	0	0	0	0	0	0	0	0	0	0.15
TGGTTGACGGTGTGGTAGCGGCCC	0.27	0	0	0	0	0.3	0	0	0	0	0	0	0	0	0	0	0	0	0	0	0	0.57
GGTTGACGGTGTGGTAGCGGCC	0.27	0	0	0	0	0	0	0	0	0	0	0	0	0	0	0	0	0	0	0	0	0.27
GGTTGACGGTGTGGTAGCG	0.27	0	0	0	0	0	0	0	0	0	0	0	0	0	0	0	0	0	0	0	0	0.27
GGTTGACGGTGTGGTAGCGGCCCA	0	0.26	0	0	0	0.15	0	0	0	0	0	0	0	0	0	0	0	0	0	0	0	0.41
GGTTGACGGTGTGGTAGC	0	0	0	0	0	0.15	0	0	0	0	0	0	0	0	0	0	0	0	0	0	0	0.15
GGTTGACGGTGTGGTAGCGGCCC	0.27	0	0	0	0	0	0	0	0	0	0	0	0	0	0	0	0	0	0	0	0	0.27
GTTGACGGTGTGGTAGCGGCCCA	0.54	0.53	0	0	0	0.15	0	0	0	0	0	0	0	0	0	0	0	0	0	0	0	1.22
GTTGACGGTGTGGTAGCGGCC	0	0	0	0	0	0.3	0	0	0	0	0	0	0	0	0	0	0	0	0	0	0	0.3
GTTGACGGTGTGGTAGCGGCCC	0.27	0	0	0	0	0	0	0	0	0	0	0	0	0	0	0	0	0	0	0	0	0.27
TTGACGGTGTGGTAGCGGCCCACA	0.27	0	0	0	0	0	0	0	0	0	0	0	0	0	0	0	0	0	0	0	0	0.27
TTGACGGTGTGGTAGCGGCCC	0.27	0	0	0	0	0	0	0	0	0	0	0	0	0	0	0	0	0	0	0	0	0.27
TTGACGGTGTGGTAGCGGCCCA	0.27	0	0	0	0	0	0	0	0	0	0	0	0	0	0	0	0	0	0	0	0	0.27
TGACGGTGTGGTAGCGGC	0	0	0	0	0	0	0	0	0	0	0	0	0	0	0.24	0	0	0	0	0	0	0.24
ACGGTGTGGTAGCGGCCCACAG	0	0.26	0	0	0	0	0	0	0	0	0	0	0	0	0	0	0	0	0	0	0	0.26
CGGTGTGGTAGCGGCCCA	0	0	0	0	0	0.15	0	0	0	0	0	0	0	0	0	0	0	0	0	0	0	0.15

GTAGGAGGAGGAAAGCATGGAGGGGTGGAAGCAACTGGCACTATCTCTTTTTATTTTGGTTGACGGTGTGGTAGCGGCCCACAG

-16.70 ........................((((..((..(((.(((.(((((..((......))..)).)))))).))).))))).. []

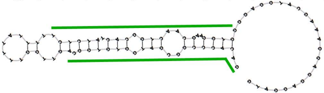

AT4G27410.1-1

Sequence	GSM642338	GSM442934	Total
GTAAATAAAGACTGAAACTTTCC	0.2	0	0.2
GTAAATAAAGACTGAAACTTTCCCAGATC	0.05	0	0.05
ATAAAGTTTGCTTCTTTGTTACAG	0	0.26	0.26
AAAGTTTGCTTCTTTGTTACAG	0.6	0	0.6

```
      GTAAATAAAGACTGAAACTTTCCCAGATCCAAATTCGTTAAAATTGAAATCAAAATCTGATAAAGTTTGCTTCTTTGTTACAG
-17.10 ((.((((((((...(((((((..(((((.....((((.......))))......)))))..)))))))...))))))))))..  []
```

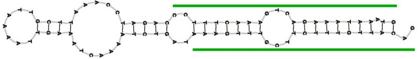

LOC_Os02g13250.1-5

Sequence	GSM278572	GSM693279	GSM693280	GSM520635	GSM520636	GSM278571	GSM520639	GSM455965	GSM686039	GSM686040	Total
GTGCAATTTGTGGCATCAGTCGGG	0.67	0	0	0	0	0	0	0	0	0	0.67
GTGCAATTTGTGGCATCAGT	0	0.1	0	0	0	0	0	0	0	0	0.1
TGCAATTTGTGGCATCAGTCGGG	0.33	0	0	0	0	0	0	0	0	0	0.33
GCAATTTGTGGCATCAGTCGGGTC	0.33	0	0	0	0	0	0	0	0	0	0.33
CAATTTGTGGCATCAGTCGGGTC	0	0	0.11	0	0	0	0	0	0	0	0.11
AATTTGTGGCATCAGTCGGGTCAG	0	0	0	0.16	0	0.31	0	0	0	0	0.47
AATTTGTGGCATCAGTCGGGTCA	0	0	0	0	0.23	0	0	0	0	0	0.23
AATTTGTGGCATCAGTCGGGT	0	0	0.11	0	0	0	0	0	0	0	0.11
TTTGTGGCATCAGTCGGGTCAGAC	0	0.1	0	0	0	0	0	0	0	0	0.1
GGCATCAGTCGGGTCAGACAAAGA	0	0	0	0.16	0	0	0	0	0	0	0.16
ATCAGTCGGGTCAGACAAAGAAGA	0	0.1	0	0.33	0.23	0	0.44	0.25	0	0	1.35
TCAGTCGGGTCAGACAAAGAAGAA	0	0	0	0	0	0.31	0	0	0	0	0.31
TCGGGTCAGACAAAGAAGAAGTAC	0	0	0.11	0	0	0	0	0	0	0	0.11
AGAAGTACTAGGATTGGAATGGGA	0.33	0	0	0.16	0	0	0	0	0	0	0.49
AGAAGTACTAGGATTGGAATGGG	0	0	0	0	0	0	0	0	0.08	0	0.08
GAAGTACTAGGATTGGAATGGGA	0	0.1	0	0	0	0	0	0	0	0	0.1
AGTACTAGGATTGGAATGGGACGG	0	0	0	0	0	0	0	0	0.08	0.08	0.16
AGGATTGGAATGGGACGGTACAG	0	0	0	0	0	0	0	0	0	0.08	0.08
GATTGGAATGGGACGGTACAG	0	0	0	0	0	0	0	0	0.08	0	0.08
ATTGGAATGGGACGGTACA	0	0	0	0	0	0	0	0	0	0.08	0.08

GTGCAATTTGTGGCATCAGTCGGGTCAGACAAAGAAGAAGTACTAGGATTGGAATGGGACGGTACAG

-8.10 ((((...((.((...((((((.(((...((.........)))))..)))))))..)).)).)..)))).. []

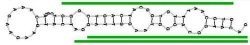

LOC_Os02g52100.1-2

Sequence	GSM520635	GSM520636	GSM278571	GSM693280	GSM693279	GSM571078	GSM520638	GSM520637	GSM686039	GSM520634	GSM278572	GSM409318	GSM686040	GSM455965	Total
GTTGCATGGCCGCCCTAAGTCGGT	0.66	0	0	0	0	0	0	0	0	0	0	0	0	0	0.66
ATGGCCGCCCTAAGTCGGTTCA	0	0.23	0	0	0	0	0	0	0	0	0	0	0	0	0.23
CGCCCTAAGTCGGTTCATATCGAG	0.33	0	0	0	0	0	0	0	0	0	0	0	0	0	0.33
CCCTAAGTCGGTTCATATCGAGGC	0	0	0.31	0.11	0	0	0	0	0	0	0	0	0	0	0.42
CCTAAGTCGGTTCATATCGA	0	0	0	0	0.1	0	0	0	0	0	0	0	0	0	0.1
CCTAAGTCGGTTCATATCGAGGCT	0	0	0	0	0.1	0.24	0	0	0	0	0	0	0	0	0.34
CTAAGTCGGTTCATATCGAGGCT	0	0	0	0.11	0.1	0	0	0	0	0	0	0	0	0	0.21
CTAAGTCGGTTCATATCGA	0	0	0	0.11	0.1	0	0	0	0	0	0	0	0	0	0.21
CTAAGTCGGTTCATATCGAGG	0	0	0	0	0	0	0.2	0	0	0	0	0	0	0	0.2
CTAAGTCGGTTCATATCG	0	0	0	0	0.1	0	0	0	0	0	0	0	0	0	0.1
CTAAGTCGGTTCATATCGAG	0	0	0	0.11	0	0	0	0	0	0	0	0	0	0	0.11
TAAGTCGGTTCATATCGAG	0	0	0	0.11	0	0	0	0	0	0	0	0	0	0	0.11
TAAGTCGGTTCATATCGAGGCT	0	0	0	0	0.1	0	0	0	0	0	0	0	0	0	0.1
TAAGTCGGTTCATATCGAGGC	0	0	0	0	0.1	0	0.2	0.44	0	0	0	0	0	0	0.74
TAAGTCGGTTCATATCGAGGCTGG	0	0	0	0.11	0.1	0	0.2	0	0	0	0	0	0	0	0.41
TAAGTCGGTTCATATCGA	0	0	0	0.11	0	0	0	0	0	0	0	0	0	0	0.11
AAGTCGGTTCATATCGAG	0	0	0	0.11	0.1	0	0	0	0	0	0	0	0	0	0.21
AAGTCGGTTCATATCGAGGCT	0	0	0	0.11	0.1	0	0	0	0	0	0	0	0	0	0.21
AAGTCGGTTCATATCGAGGC	0	0	0	0.11	0	0.24	0	0	0.08	0	0	0	0	0	0.43
AAGTCGGTTCATATCGAGGCTGGG	0	0	0	0	0	0	0	0	0	0.37	0	0	0	0	0.37
AGTCGGTTCATATCGAGGCTGG	0	0	0	0	0	0	0.2	0	0	0	0	0	0	0	0.2
AGTCGGTTCATATCGAGGCTGGGG	0.16	0	0	0	0	0	0	0	0	0	0	0	0	0	0.16
TCGGTTCATATCGAGGCTGGG	0	0	0	0	0	0	0	0	0	0	0.33	17.53	0	0	17.86
CATATCGAGGCTGGGGGAGAAGGC	0	0	0	0	0.2	0	0	0	0	0	0	0	0.08	0	0.28
AGGCGCTACCCTTATTTAAGCTCC	0	0	0	0.11	0	0	0	0	0	0	0	0	0	0	0.11
GCGCTACCCTTATTTAAGCTCCTC	0	0	0	0	0	0.24	0	0	0	0	0	0	0	0	0.24
TAAGCTCCTCAAACGCTCCGGGCC	0	0	0	0	0	0	0	0	0	0	0	0	0	0.25	0.25
TAAGCTCCTCAAACGCTCCGGG	0	0	0	0	0	0	0	0	0	0	0	0	0	0.25	0.25
AAGCTCCTCAAACGCTCCGGGCCG	0.16	0	0.31	0	0	0	0	0	0	0	0	0	0.08	0	0.55
GCTCCTCAAACGCTCCGGGCCGTT	0.16	0	0	0	0	0	0	0	0	0	0	0	0	0	0.16
GCTCCGGGCCGTTTACTTGGACGG	0	0	0	0.11	0	0	0	0	0	0	0	0	0.08	0	0.19
CGGGCCGTTTACTTGGACGGAG	0	0	0	0	0.1	0	0	0	0	0	0	0	0.08	0	0.18
CGGGCCGTTTACTTGGACGGA	0	0	0	0	0.1	0	0	0	0	0	0	0	0	0	0.1

```
       GTTGCATGGCCGCCCTAAGTCGGTTCATATCGAGGCTGGGGGAGAAGGCGCTACCCTTATTTAAGCTCCTCAAACGCTCCGGGCCGTTTACTTGGACGGAG
-28.01 .........(((..((((((((((.....(((..((..((((((((((......)))........))))))....))..)))))))...)))))).)))..  []
```

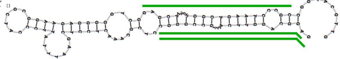

LOC_Os03g06520.1-6

Sequence	GSM520640	GSM520638	GSM455965	GSM520636	GSM520637	GSM520635	GSM520634	GSM520639	GSM686039	GSM693279	GSM409322(Leaf 1)	Total
GTATGCACATTCGTGGTAG	0.2	0.2	0.5	0	0	0	0	0	0	0	0	0.9
GTATGCACATTCGTGGTAGCCAGGAT	0.2	0	0	0	0	0	0	0	0	0	0	0.2
GTATGCACATTCGTGGTAGCCAGG	0	0.2	0	0.23	0	0.16	0	0	0	0	0	0.59
GTATGCACATTCGTGGTAGCCAGGA	0.2	0	0.5	0	0.22	0.16	0	0	0	0	0	1.08
GTATGCACATTCGTGGTAGCC	0	0.41	0	0	0	0	0	0	0	0	0	0.41
GTATGCACATTCGTGGTAGCCA	0	0	0	0.23	0	0	0	0	0	0	0	0.23
GTATGCACATTCGTGGTAGCCAG	0	0.2	0	0	0	0	0	0	0	0	0	0.2
TAGCCAGGATTCGAACTCGAGACC	0	0	0	0	0	0.16	0	0	0	0	0	0.16
AGCCAGGATTCGAACTCGAGACC	0	0	0.5	0	0	0.16	0.37	0	0	0	0	1.03
AGCCAGGATTCGAACTCGAGAC	0	0.2	0	0	0	0	0	0	0	0	0	0.2
AGCCAGGATTCGAACTCGAGACCT	0	0	0.74	0	0.22	0.99	0	0	0	0	0	1.95
AGCCAGGATTCGAACTCGAGACCTCT	0.2	0	0	0	0	0	0	0	0	0	0	0.2
GCCAGGATTCGAACTCGAGACCTC	0.2	0	0.74	0	0.22	0.33	0	0	0	0	0	1.49
GCCAGGATTCGAACTCGAGACCT	0	0	0.25	0	0	0	0	0	0	0	0	0.25
CCAGGATTCGAACTCGAGACCTC	0	0	0	0	0	0	0	0.22	0	0	0	0.22
CAGGATTCGAACTCGAGACCTC	0.2	0.2	0.25	0	0.22	0	0	0	0.08	0	0	0.95
AGGATTCGAACTCGAGACCTCTGG	0.39	0.2	0.25	0	0	0	0.73	0.66	0.08	0	11.76	14.07
AGGATTCGAACTCGAGACCTC	0	0	0	0	0	0	0	0	0	0.1	0	0.1
AGGATTCGAACTCGAGACCTCT	0	0.2	0	0	0	0	0	0	0	0	0	0.2
GATTCGAACTCGAGACCTCTGG	0	0	0	0	0	0.16	0	0	0	0	0	0.16
GATTCGAACTCGAGACCTC	0	0	0.25	0	0	0	0	0	0	0	0	0.25
GATTCGAACTCGAGACCTCTG	0	0.2	0	0	0	0	0	0	0	0	0	0.2
ATTCGAACTCGAGACCTCTGGGTT	0	0	0	0	0.22	0	0	0	0	0	0	0.22
ATTCGAACTCGAGACCTCTGGGTTTCT	0.2	0	0	0	0	0	0	0	0	0	0	0.2
TTCGAACTCGAGACCTCTGGGTT	0	0	0.25	0	0	0	0	0	0	0	0	0.25
TCGAACTCGAGACCTCTGGGTTTCT	0	0	0.25	0	0	0	0	0	0	0	0	0.25
TCGAACTCGAGACCTCTGGGTTT	0	0	0.25	0	0	0	0	0	0	0	0	0.25
TCGAGACCTCTGGGTTTCTGA	0	0.2	0	0	0	0	0	0	0	0	0	0.2
TCTGATACCATGTGTGTATGCA	0	0	0.74	0	0	0	0	0	0	0	0	0.74
CTGATACCATGTGTGTATGCA	0.2	0	0	0	0	0	0	0	0	0	0	0.2
TGATACCATGTGTGTATGCAG	0	0	0.25	0	0	0	0	0	0	0	0	0.25
GATACCATGTGTGTATGCA	0	0	0.25	0	0	0	0	0	0	0	0	0.25

GTATGCACATTCGTGGTAGCCAGGATTCGAACTCGAGACCTCTGGGTTTCTGATACCATGTGTGTATGCAG

-27.90 ((((((((..(((((((..(((((.....((((.((....))))))))))).)))))))))))))))).. []

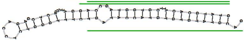

LOC_Os03g36170.1-6

Sequence	GSM520635	GSM455965	GSM520636	GSM693279	GSM693280	GSM686039	GSM455962	GSM278572	GSM686040	GSM409313	Total
GTACTATGCTTGATGAATATGGTG	0.16	0	0	0	0	0	0	0	0	0	0.16
ACTATGCTTGATGAATATGGTG	0	0.25	0	0	0	0	0	0	0	0	0.25
ACTATGCTTGATGAATATGGTGTT	0	0	0.23	0	0	0	0	0	0	0	0.23
TATGCTTGATGAATATGGTGTT	0	0	0	0.1	0	0	0	0	0	0	0.1
ATGCTTGATGAATATGGTGTT	0	0	0	0.1	0	0	0	0	0	0	0.1
CTTGATGAATATGGTGTTTCCGA	0	0	0	0	0.11	0	0	0	0	0	0.11
CTTGCCATGCAACCAATAGGCTT	0	0	0	0	0	0.08	0	0	0	0	0.08
TCTACGAGCTAATTGTTGG	0	0	0	0	0	0	3.96	0	0	0	3.96
GAGCTAATTGTTGGGAGGAAG	0	0	0	0	0	0	0	0.33	0	0	0.33
CTAATTGTTGGGAGGAAGCCAAAT	0	0	0	0	0.11	0	0	0	0	0	0.11
CTAACCAAATTTAAAAGTCGGTGT	0	0	0	0	0	0.08	0	0	0	0	0.08
AACCAAATTTAAAAGTCGGTGTGA	0	0	0	0	0	0.16	0	0	0	0	0.16
AAAGTCGGTGTGATGAAGGGTTTC	0	0	0	0	0.11	0	0	0	0	0	0.11
AAGTCGGTGTGATGAAGGGTTT	0	0	0	0	0	0	0	0	0.08	0	0.08
CGGTGTGATGAAGGGTTTCTTCTA	0	0	0	0	0	0	0	0	0	10.05	10.05

```
GTACTATGCTTGATGAATATGGTGTTTCCGATACCTTTTGGGTGGAAGCCATAAACACTGCTTGCCATGCAACCAATAGGCTTTATTTGCATCATCTCTTGATAGACTTTCTACGAGCTAATTGTTGGGAGGAAGCCAAATATTGCCTATTTTTGAGTTTTTGGTTGCAAGTGTTATATTTACCGAAAGGGAGTTAGACTAACCAAATTTAAAAGTCGGTGTGATGAAGGGTTTCTTCTAG
-64.10 .((((..((((..(((((.((((...((..((.((((((.((((((.......((((((((..((((...((((((((((..((((((.((..((((..((((.(((.....)))....))))..)))))).)).)))))..))))))...)).))).)..)))).)).)))..)))).))).)))))...)))))).)))))).))..))...)))).))))).)))).))))...................... []
```

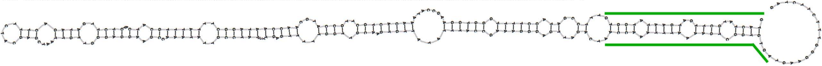

LOC_Os03g57750.2-1

		GSM520637	GSM278572	GSM409321	Total
	GTGTGAGACGAATTGTTGGT	0.22	0	0	0.22
	CGAACAGTAGGAATTCGACA	0.22	0	0	0.22
	TCCGGCAATCGTCTCATCTG	0	0.33	0	0.33
	CGGCAATCGTCTCATCTGCAG	0	0	7.33	7.33
	GTGTGAGACGAATTGTTGGTTCAGCTTCGCTGATCAAATCGAATTGACTTCCTCTTTTTTTTTCCTTTCTCTGCACATTAGCTCTTGTTAGAATCGAACAGTAGGAATTCGACAAATTCTGTTCTCGCGTTCTTCTTCATCAATCATCTTCGTGATCCGGCAATCGTCTCATCTGCAG				
-38.16	(((((((((.((((((((.(((....(((.((.((.......((((.((((((((.(((..(((........((......))........)))..))).)).)))))).)))).......)).))..)))......................))).)))))))))))))))))..))..	[]			

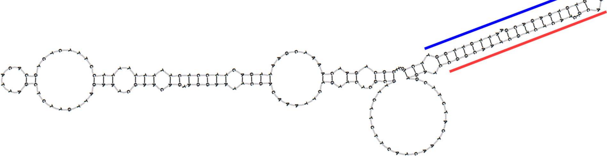

LOC_Os04g09380.1-5

	GSM693280	GSM693279	GSM571078	GSM278572	GSM278571	GSM520635	GSM520637	GSM520634	GSM520638	GSM686039	GSM520636	Total
GTGGAGCACGTGGATGATCAGGAA	0.75	1.1	0.49	0.33	0.92	0	0	0	0	0	0	3.59
GTGGAGCACGTGGATGATCAGGA	0.11	0.1	0	0	0	0	0	0	0	0	0	0.21
TGGAGCACGTGGATGATCAGGAA	0.11	0.1	0	0	0	0	0	0	0	0	0	0.21
TGGAGCACGTGGATGATCAG	0.11	0	0	0	0	0	0	0	0	0	0	0.11
TGGAGCACGTGGATGATCAGGA	0.22	0	0	0.33	0	0	0	0	0	0	0	0.55
TGGAGCACGTGGATGATCA	0.11	0	0	0	0	0	0	0	0	0	0	0.11
TGGAGCACGTGGATGATCAGG	0	0	0	0	0.31	0	0	0	0	0	0	0.31
TGGAGCACGTGGATGATCAGGAAA	0.32	0	0	0.33	0	0	0	0	0	0	0	0.65
GGAGCACGTGGATGATCAGGAAAC	0.11	0	0	0	0	0	0	0	0	0	0	0.11
GAGCACGTGGATGATCAGGAAACT	0	0	0	0	0.31	0	0	0	0	0	0	0.31
AGCACGTGGATGATCAGGAAACT	0	0	0	0	0.31	0	0	0	0	0	0	0.31
AGCACGTGGATGATCAGGAAAC	0	0.1	0	0	0	0	0	0	0	0	0	0.1
GGATGATCAGGAAACTAAAATTGT	0	0	0	0	0	0.16	0	0	0	0	0	0.16
AATAATGTTCATGATACTGTTCAG	0	0	0	0	0	0	0.22	0	0	0	0	0.22
ATGATACTGTTCAGCACTCACCTC	0	0	0	0	0	0	0	0.37	0.2	0	0	0.57
GATACTGTTCAGCACTCACCTCCT	0	0	0	0	0	0.16	0	0	0	0	0	0.16
TACTGTTCAGCACTCACCTCCTGT	0	0	0	0	0	0	0	0	0	0.08	0	0.08
ACTGTTCAGCACTCACCTCCTGTT	0	0	0	0	0	0.16	0	1.47	0	0	0.94	2.57

```
       GTGGAGCACGTGGATGATCAGGAAACTAAAATTGTTGATAATAATGTTCATGATACTGTTCAGCACTCACCTCCTGTTTTGTAG
-13.61 ..((((...(((..(((.(((..........(((((.....)))))..........))).))).)))....))))..........   []
```

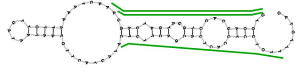

LOC_Os04g45540.1-3

	GSM520636	GSM520635	GSM693280	GSM278571	GSM693279	GSM571078	GSM520638	GSM520637	GSM686039	GSM520634	GSM520639	GSM686040	GSM409321	GSM409318	GSM278572	GSM409324	GSM455965	GSM571077	GSM520640	Total
ATGGCCGCCCTAAGTCGGTTCA	0.23	0	0	0	0	0	0	0	0	0	0	0	0	0	0	0	0	0	0	0.23
CGCCCTAAGTCGGTTCATATCGAG	0	0.33	0	0	0	0	0	0	0	0	0	0	0	0	0	0	0	0	0	0.33
CCCTAAGTCGGTTCATATCGAGGC	0	0	0.11	0.31	0	0	0	0	0	0	0	0	0	0	0	0	0	0	0	0.42
CCTAAGTCGGTTCATATCGAGGCT	0	0	0	0	0.1	0.24	0	0	0	0	0	0	0	0	0	0	0	0	0	0.34
CCTAAGTCGGTTCATATCGA	0	0	0	0	0.1	0	0	0	0	0	0	0	0	0	0	0	0	0	0	0.1
CTAAGTCGGTTCATATCGA	0	0	0.11	0	0.1	0	0	0	0	0	0	0	0	0	0	0	0	0	0	0.21
CTAAGTCGGTTCATATCGAG	0	0	0.11	0	0	0	0	0	0	0	0	0	0	0	0	0	0	0	0	0.11
CTAAGTCGGTTCATATCGAGGCT	0	0	0.11	0	0.1	0	0	0	0	0	0	0	0	0	0	0	0	0	0	0.21
CTAAGTCGGTTCATATCGAGG	0	0	0	0	0	0	0.2	0	0	0	0	0	0	0	0	0	0	0	0	0.2
CTAAGTCGGTTCATATCG	0	0	0	0	0.1	0	0	0	0	0	0	0	0	0	0	0	0	0	0	0.1
TAAGTCGGTTCATATCGAGGC	0	0	0	0	0.1	0	0.2	0.44	0	0	0	0	0	0	0	0	0	0	0	0.74
TAAGTCGGTTCATATCGAGGCTGG	0	0	0.11	0	0.1	0	0.2	0	0	0	0	0	0	0	0	0	0	0	0	0.41
TAAGTCGGTTCATATCGAGGCT	0	0	0	0	0.1	0	0	0	0	0	0	0	0	0	0	0	0	0	0	0.1
TAAGTCGGTTCATATCGA	0	0	0.11	0	0	0	0	0	0	0	0	0	0	0	0	0	0	0	0	0.11
TAAGTCGGTTCATATCGAG	0	0	0.11	0	0	0	0	0	0	0	0	0	0	0	0	0	0	0	0	0.11
AAGTCGGTTCATATCGAGGC	0	0	0.11	0	0	0.24	0	0	0.08	0	0	0	0	0	0	0	0	0	0	0.43
AAGTCGGTTCATATCGAGGCT	0	0	0.11	0	0.1	0	0	0	0	0	0	0	0	0	0	0	0	0	0	0.21
AAGTCGGTTCATATCGAG	0	0	0.11	0	0.1	0	0	0	0	0	0	0	0	0	0	0	0	0	0	0.21
AAGTCGGTTCATATCGAGGCTGGG	0	0	0	0	0	0	0	0	0	0.37	0	0	0	0	0	0	0	0	0	0.37
AGTCGGTTCATATCGAGGCTGGGA	0	0	0	0	0	0	0	0	0	0	0.66	0	0	0	0	0	0	0	0	0.66
AGTCGGTTCATATCGAGGCTGG	0	0	0	0	0	0	0.2	0	0	0	0	0	0	0	0	0	0	0	0	0.2
TCGGTTCATATCGAGGCTGGGAGA	0	0	0.11	0	0.1	0.24	0	0	0	0	0	0.08	0	0	0	0	0	0	0	0.53
TCGGTTCATATCGAGGCTGGGA	0	0	0	0	0	0	0	0	0	0	0	0	7.33	0	0	0	0	0	0	7.33
TCGGTTCATATCGAGGCTGGG	0	0	0	0	0	0	0	0	0	0	0	0	0	17.53	0.33	0	0	0	0	17.86
CGGTTCATATCGAGGCTGGGAGAG	0	0	0	0	0.1	0	0	0	0	0	0	0	0	0	0	0	0	0	0	0.1
CGGTTCATATCGAGGCTGGGAGA	0	0	0	0	0.1	0	0	0	0	0	0	0	0	0	0	0	0	0	0	0.1
GTTCATATCGAGGCTGGGAGAGAA	0	0.16	0	0	0	0	0	0	0	0	0	0	0	0	0	0	0	0	0	0.16
TTCATATCGAGGCTGGGAGAGAAG	0	0	0	0	0	0	0	0	0	0	0	0	0	0	0.33	0	0	0	0	0.33
TCATATCGAGGCTGGGAGAGAAGG	0	0	0	0	0	0	0	0	0	0	0	0	0	0	0.33	9.04	0	0	0	9.37
CATATCGAGGCTGGGAGAGAAGGC	0	0.16	1.29	2.76	1.99	0.49	0	0	2.58	0	0	6.78	0	0	1.67	0	0.25	0.79	0	18.76
CATATCGAGGCTGGGAGAGAAGG	0	0	0.11	0	0	0	0	0	0.16	0	0	0.15	0	0	0	0	0	0	0	0.42
CATATCGAGGCTGGGAGAGAAG	0	0	0	0	0	0	0	0	0.08	0	0	0.3	0	0	0	0	0	0	0	0.38
CATATCGAGGCTGGGAGAGAAGGCG	0	0	0	0	0	0	0	0	0.08	0	0	0.08	0	0	0	0	0	0	0	0.16
ATATCGAGGCTGGGAGAGAAGGC	0	0	0.11	0	0	0	0	0	0	0	0	0.15	0	0	0	0	0	0	0.2	0.46
ATATCGAGGCTGGGAGAGAAGGCG	0	0	0	0	0.5	0	0	0	0.23	0	0	0.08	0	0	0	0	0	0	0	0.81
ATATCGAGGCTGGGAGAGAAGG	0	0	0.11	0	0	0	0	0	0	0	0	0	0	0	0.33	0	0	0.26	0	0.7
TATCGAGGCTGGGAGAGAAGGCG	0	0	0.11	0	0.2	0	0	0	0	0	0	0.08	0	0	0	0	0	0	0	0.39
TATCGAGGCTGGGAGAGAAGGCGC	0	0	0.22	0.61	0.4	0.24	0	0	0	0	0	0.38	0	0	0	0	0	0	0	1.85
TATCGAGGCTGGGAGAGAAGGC	0	0	0.11	0	0	0	0	0	0.23	0	0	0.38	0	0	0.33	0	0	0	0	1.05
TATCGAGGCTGGGAGAGAAGGCGCT	0	0	0.11	0	0	0	0	0	0	0	0	0	0	0	0	0	0	0	0	0.11
ATCGAGGCTGGGAGAGAAGGCGC	0	0	0	0	0	0	0	0	0.08	0	0	0	0	0	0	0	0	0	0	0.08
ATCGAGGCTGGGAGAGAAGGC	0	0	0	0	0	0	0	0	0.16	0	0	0.15	0	0	0	0	0	0	0	0.31
ATCGAGGCTGGGAGAGAAGGCGCT	0	0	0	0	0	0	0	0	0	0	0	0.23	0	0	0	0	0	0	0	0.23
TCGAGGCTGGGAGAGAAGGCGC	0	0	0.11	0	0	0	0	0	0	0	0	0.08	0	0	0	0	0	0	0	0.19
TCGAGGCTGGGAGAGAAGGCGCTA	0	0	0.11	0.31	0.7	0.24	0	0	0	0	0	0.3	0	0	0	0	0	0	0	1.66
TCGAGGCTGGGAGAGAAGGCGCT	0	0	0	0	0.1	0	0	0	0.08	0	0	0	0	0	0	0	0	0	0	0.18
TCGAGGCTGGGAGAGAAGGCG	0	0	0	0	0	0	0	0	0.08	0	0	0	0	0	0	0	0	0	0	0.08
TCGAGGCTGGGAGAGAAGGC	0	0	0	0	0	0	0	0	0.08	0	0	0	0	0	0	0	0	0	0	0.08
CGAGGCTGGGAGAGAAGGCGCTA	0	0	0	0	0	0	0	0	0	0	0	0.15	0	0	0	0	0	0	0	0.15
CGAGGCTGGGAGAGAAGGC	0	0	0	0	0.1	0	0	0	0	0	0	0	0	0	0	0	0	0	0	0.1
CGAGGCTGGGAGAGAAGGCGCT	0	0	0	0	0	0	0	0	0.08	0	0	0	0	0	0	0	0	0	0	0.08
CGAGGCTGGGAGAGAAGGCGCTAC	0	0	0	0	0.2	0	0	0	0	0	0	0	0	0	0	0	0	0	0	0.2
GAGGCTGGGAGAGAAGGCGCT	0	0	0.11	0	0.2	0	0	0	0.16	0	0	0	0	0	0	0	0	0	0	0.47
GAGGCTGGGAGAGAAGGCGCTAC	0	0	0	0	0	0	0	0	0	0	0	0.08	0	0	0	0	0	0	0	0.08
GAGGCTGGGAGAGAAGGCGCTACC	0	0.66	0	0	0.1	0	0	0	0	0	0	0	0	0	0	0	0	0	0	0.76
GAGGCTGGGAGAGAAGGCGCTA	0	0	0	0	0.4	0	0	0	0.08	0	0	0.08	0	0	0	0	0	0	0	0.56
GAGGCTGGGAGAGAAGGC	0	0	0	0	0	0	0	0	0	0	0	0.08	0	0	0	0	0	0	0	0.08
GAGGCTGGGAGAGAAGGCG	0	0	0	0	0	0	0	0	0.08	0	0	0	0	0	0	0	0	0	0	0.08
GAGGCTGGGAGAGAAGGCGC	0	0	0	0	0	0	0	0	0.08	0	0	0	0	0	0	0	0	0	0	0.08
AGGCTGGGAGAGAAGGCGCTA	0	0	0	0	0.2	0	0	0	0	0	0	0	0	0	0	0	0	0	0	0.2
AGGCTGGGAGAGAAGGCGCTACCC	0	0.33	0	0	0	0	0	0	0	0	0	0	0	0	0	0	0	0	0	0.33
GCTCCGGGCCGTTTATCTGGACGG	0	0	0	0	0	0	0	0	0	0	0	0	0	0	0	0	0.25	0	0	0.25
CCGGGCCGTTTATCTGGACGGAG	0	0	0	0	0	0	0	0	0	0	0	0.08	0	0	0	0	0	0	0	0.08

GTATGGCCGCCCTAAGTCGGTTCATATCGAGGCTGGGAGAGAAGGCGCTACCCCTATTTAAACTCGTCAAACGCTCCGGGCCGTTTATCTGGACGGAG

-24.33 ....((.(((((.((.((((((.((((....((((..................))))....)))).)))).)).)).))))).)). []

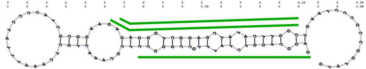

LOC_Os06g23260.1-5

Sequence	GSM693279	GSM455965	GSM693280	GSM520640	GSM520635	GSM520638	GSM686040	GSM686039	GSM520636	GSM520634	GSM520637	GSM278572	GSM278571	GSM571078	Total
GTCATCGATCCGGATAGATTAGA	0.1	0	0	0	0	0	0	0	0	0	0	0	0	0	0.1
GTCATCGATCCGGATAGATTAGAA	0.2	0.25	0	0.59	0.16	0	0	0	0	0	0	0	0	0	1.2
GTCATCGATCCGGATAGATTA	0.1	0	0.11	0	0	0	0	0	0	0	0	0	0	0	0.21
GTCATCGATCCGGATAGATTAG	0.1	0	0	0	0	0	0	0	0	0	0	0	0	0	0.1
CATCGATCCGGATAGATTAGAA	0	0	0	0	0	0.2	0	0	0	0	0	0	0	0	0.2
ATCGATCCGGATAGATTAGAA	0	0	0	0	0	0	0.08	0	0	0	0	0	0	0	0.08
ATCGATCCGGATAGATTAGAAG	0	0	0	0	0	0.2	0	0	0	0	0	0	0	0	0.2
TCGATCCGGATAGATTAGAAG	0	0	0	0	0	0.41	0	0	0	0	0	0	0	0	0.41
TCCGGATAGATTAGAAGCCCT	0	0	0.11	0	0	0	0	0	0	0	0	0	0	0	0.11
TCCGGATAGATTAGAAGCCC	0	0	0	0	0	0	0	0.08	0	0	0	0	0	0	0.08
AGAATGATGTGGTGCAAT	0.1	0	0	0	0	0	0	0	0	0	0	0	0	0	0.1
AATGATGTGGTGCAATGTCTCGTC	0	0	0	0.2	0	0	0	0	0.23	0	0	0	0	0	0.43
ATGATGTGGTGCAATGTCTCGTCA	0	0	0	0	0	0	0	0	0	0.37	0	0	0	0	0.37
GATGTGGTGCAATGTCTCGTCAGT	0	0.25	0	0	0.16	0	0	0	0	0	0	0	0	0	0.41
TGCAATGTCTCGTCAGTTTTGAGT	0	0	0	0	0	0	0	0	0	0	0.22	0	0	0	0.22
GCAATGTCTCGTCAGTTTTG	0	0	0	0	0	0	0	0	0	0	0	0.33	0	0	0.33
AATGTCTCGTCAGTTTTGAGTTGA	0	0	0	0.59	0.16	0.2	0	0	0.94	0	0.44	0	0	0	2.33
AATGTCTCGTCAGTTTTGAGT	0	0	0	0	0	0.2	0	0	0	0	0	0	0	0	0.2
ATGTCTCGTCAGTTTTGAGTTGAT	0	0	0	0.2	0	0.2	0	0	0	0	0	0	0	0	0.4
GTCTCGTCAGTTTTGAGTTGATATT	0	0	0	0	0	0.2	0	0	0	0	0	0	0	0	0.2
TCAGTTTTGAGTTGATATTTCCAC	0	0	0	0	0	0	0	0	0	0	0	0.33	0.31	0	0.64
ATTTTTCAATATAATGACGCAT	0	0.25	0	0	0	0	0	0	0	0	0	0	0	0	0.25
TTTTTCAATATAATGACGCATCT	0	0.25	0	0	0	0	0	0	0	0	0	0	0	0	0.25
TTTCAATATAATGACGCATCTGCT	0	0	0	0	0	0	0.15	0	0	0	0	0	0	0	0.15
TTCAATATAATGACGCATCTGCTT	0	0	0.11	0	0	0	0	0	0	0	0	0	0	0	0.11
TTCAATATAATGACGCATCTGC	0	0	0.11	0	0	0	0	0	0	0	0	0	0	0	0.11
TCAATATAATGACGCATCTGCTTT	0	0	0	0	0	0	0	0	0	0	0	0.33	0	0	0.33
CAATATAATGACGCATCTGCTTTG	0	0	0	0	0	0	0.08	0	0	0	0	0	0	0	0.08
AATATAATGACGCATCTGCTTTGT	0	0	0	0	0	0	0	0	0	0	0	0.33	0	0	0.33
ATATAATGACGCATCTGCTTTGT	0	0	0	0.2	0	0	0	0	0	0	0	0	0	0	0.2
ATAATGACGCATCTGCTTTGTCAC	0	0	0	0	0	0	0	0	0	0	0	0	0	0.24	0.24
ATAATGACGCATCTGCTTTGT	0	0	0	0	0	0	0.08	0.08	0	0	0	0	0	0	0.16
ATAATGACGCATCTGCTTT	0	0	0	0	0	0.2	0	0.08	0	0	0	0	0	0	0.28
GCATCTGCTTTGTCACCTTGTGA	0	0.25	0	0	0	0	0	0	0	0	0	0	0	0	0.25
CTGCTTTGTCACCTTGTGAAAGAG	0.1	0	0	0	0	0	0	0	0	0	0	0	0	0	0.1

```
       GTCATCGATCCGGATAGATTAGAAGCCCTTTAGAATGATGTGGTGCAATGTCTCGTCAGTTTTGAGTTGATATTTCCACATTCATTTTTCAATATAATGACGCATCTGCTTTGTCACCTTGTGAAAGAG
-26.77 .(((.(((....((((((.((((.((.....((((((((((((...((((((...((......))...)))))).)))))).)))))).............)).)))).))))))...)))))).....  []
```

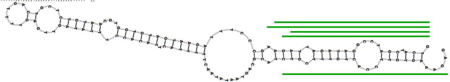

LOC_Os06g29760.1-1

Sequence	GSM455965	GSM520635	GSM606040	GSM693280	GSM693279	GSM520637	GSM520640	GSM606039	GSM278572	GSM278571	GSM571077	GSM520636	GSM520638	GSM529298	GSM520639	Total
GTTAGATTGTAAATAGGAAAGCGC	0.25	1.81	0.38	0.32	0.9	0.22	0.99	0.08	0.67	0.61	0	0	0	0	0	6.23
GTTAGATTGTAAATAGGAAAGC	0	0	0.15	0	0	0	0	0	0	0	0	0	0	0	0	0.15
GTTAGATTGTAAATAGGAAA	0	0.16	0	0	0	0	0	0.08	0	0	0	0	0	0	0	0.24
GTTAGATTGTAAATAGGAAAG	0	0	0	0	0.1	0	0	0	0	0	0	0	0	0	0	0.1
TTAGATTGTAAATAGGAAAGCGCGCT	0	0	0	0	0	0.44	0	0	0	0	0	0	0	0	0	0.44
TTAGATTGTAAATAGGAAAGC	0	0	0	0	0.2	0	0	0.08	0.33	0	0	0	0	0	0	0.61
TTAGATTGTAAATAGGAAAG	0	0	0	0	0	0	0	0.08	0	0	0	0	0	0	0	0.08
TTAGATTGTAAATAGGAAAGCG	0	0	0.08	0	0	0	0	0	0	0	0	0	0	0	0	0.08
TTAGATTGTAAATAGGAAAGCGCG	0	0	0	0.22	0	0	0	0	0.33	0	0	0	0	0	0	0.55
TTAGATTGTAAATAGGAAAGCGC	0	0	0	0	0.1	0	0	0.08	0.33	0	0	0	0	0	0	0.51
TAGATTGTAAATAGGAAAG	0	0	0.08	0	0	0	0	0	0	0	0	0	0	0	0	0.08
TAGATTGTAAATAGGAAAGCGCG	0	0	0.08	0	0.2	0	0	0	0.33	0	0.26	0	0	0	0	0.87
TAGATTGTAAATAGGAAAGCGCGC	0	0	0.45	0.32	1	0.22	0	0.08	1	0.61	0.26	0	0	0	0	3.94
TAGATTGTAAATAGGAAAGC	0	0	0.08	0	0	0	0	0	0	0	0	0	0	0	0	0.08
TAGATTGTAAATAGGAAAGCGC	0	0	0	0.32	0	0	0	0	0.33	0	0	0	0	0	0	0.65
TAGATTGTAAATAGGAAAGCG	0	0	0	0	0.1	0	0	0	0	0	0	0	0	0	0	0.1
AGATTGTAAATAGGAAAGCGCG	0	0	0	0.11	0	0	0	0	0	0	0	0	0	0	0	0.11
AGATTGTAAATAGGAAAGCGCGCT	0	0.33	0.08	0.11	0.2	0	0	0.23	0	0.31	0.26	0	0	0	0	1.52
AGATTGTAAATAGGAAAGC	0	0	0	0	0.2	0	0	0	0	0	0	0	0	0	0	0.2
GATTGTAAATAGGAAAGCGCGCTC	0	1.64	0.23	0	0.2	0	0	0	0.33	0	0	0.23	0	0	0	2.63
ATTGTAAATAGGAAAGCGCGCTC	0	0.16	0	0	0	0	0	0	0	0	0	0	0	0	0	0.16
ATTGTAAATAGGAAAGCGCGCT	0	0	0	0	0	0	0	0.08	0	0	0	0	0	0	0	0.08
TTGTAAATAGGAAAGCGCGCT	0	0	0	0	0	0	0	0	0	0	0.26	0	0	0	0	0.26
TGTAAATAGGAAAGCGCGCTCATC	0	0.16	0	0	0.2	0	0	0	0.33	0.31	0	0	0	0	0	1
GTAAATAGGAAAGCGCGCTCATCA	0	0.16	0	0	0	0	0	0	0	0	0	0	0	0	0	0.16
GTAAATAGGAAAGCGCGC	0	0	0.08	0	0	0	0	0	0	0	0	0	0	0	0	0.08
TAAATAGGAAAGCGCGCTCATCAC	0	0	0	0	0.1	0	0	0	0	0	0	0	0	0	0	0.1
AAATAGGAAAGCGCGCTCATCACC	0	0	0	0.11	0.1	0	0	0	0.33	0	0	0	0	0	0	0.54
AAATAGGAAAGCGCGCTCATC	0	0	0	0	0	0	0	0	0.33	0	0	0	0	0	0	0.33
AATAGGAAAGCGCGCTCATCACCT	0	0	0	0	0.1	0	0	0	0	0	0	0	0.2	0	0	0.3
ATAGGAAAGCGCGCTCATCACC	0	0	0.08	0	0	0	0	0	0	0	0	0	0	0	0	0.08
ATAGGAAAGCGCGCTCATCACCTT	0	0	0	0	0.1	0	0	0	0	0	0	0	0	0	0	0.1
TAGGAAAGCGCGCTCATCACCTT	0	0	0	0	0	0	0	0.08	0	0	0	0	0	0	0	0.08
TAGGAAAGCGCGCTCATCACC	0	0	0	0	0	0	0	0.23	0	0	0	0	0	0	0	0.23
AGGAAAGCGCGCTCATCACCTTTC	0	0	0	0.11	0.1	0	0	0	0	0	0	0	0	0	0	0.21
AGGAAAGCGCGCTCATCAC	0	0	0.08	0	0	0	0	0	0	0	0	0	0	3.46	0	3.54
AGGAAAGCGCGCTCATCACC	0	0	0	0	0	0	0	0	0	0	0	0	0.2	0	0	0.2
AAAGATATCTTTGCATGGAAACCA	0	0	0	0.11	0	0	0	0	0	0	0	0	0	0	0	0.11
GGAAACCATCTGATATGCCCGGTAT	0	0	0	0	0.1	0	0	0	0	0	0	0	0	0	0	0.1
GGAAACCATCTGATATGCCCGGTA	0	0.16	0.15	0	0.2	0	0.2	0	0	0	0.26	0	0	0	0	0.97
GAAACCATCTGATATGCCCGGTAT	0	0.33	0	0	0	0	0	0	0	0	0	0	0	0	0.89	1.22
GAAACCATCTGATATGCCCGGTATT	0	0	0.08	0	0	0	0	0	0	0	0	0	0	0	0	0.08
GAAACCATCTGATATGCCCGGT	0	0	0	0	0	0	0	0	0	0	0	0	0.2	0	0	0.2
CATCTGATATGCCCGGTATTCCT	0	0	0.08	0	0	0	0	0	0	0	0	0	0	0	0	0.08
ATCTGATATGCCCGGTATTCCTAG	0	0.16	0.08	0	0	0	0	0.08	0	0	0	0	0	0	0	0.32
ATCTGATATGCCCGGTATTCC	0	0	0	0	0	0	0	0.08	0	0	0	0	0	0	0	0.08
GATATGCCCGGTATTCCTAG	0	0	0	0	0.1	0	0	0	0	0	0	0	0	0	0	0.1

```
      GTTAGATTGTAAATAGGAAAGCGCGCTCATCACCTTTCTTCAAAACAATAAAGATATCTTTGCATGGAAACCATCTGATATGCCCGGTATTCCTAG
-14.20 .............((((((..((.((..((((.......(((.......(((((....)))))....)))......))))..)).))...)))))).  []
```

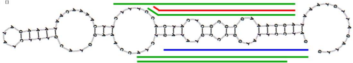

LOC_Os06g31910.1-2

Sequence	GSM686040	GSM686039	GSM278572	GSM455965	GSM520635	GSM520638	GSM693279	GSM571077	Total
GTAGACGAGCAGTTAGTAGGCTT	0.08	0.08	0.33	0	0	0	0	0	0.49
GTAGACGAGCAGTTAGTAGGCTTG	0	0	0	0.25	0	0	0	0	0.25
TAGACGAGCAGTTAGTAGGCTT	0	0	0	0	0.16	0	0	0	0.16
TAGACGAGCAGTTAGTAGGCT	0	0	0	0	0	0.2	0	0	0.2
GACGAGCAGTTAGTAGGCTT	0	0	0	0	0	0	0.1	0	0.1
TCTTCTCGTTTGTTTTTCTGCTAG	0	0	0	0	0	0	0	0.26	0.26

```
       GTAGACGAGCAGTTAGTAGGCTTGCGTGTGTGTTCTTCTCGTTTGTTTTTCTGCTAG
-11.90 (((((.((((((..((.(((..(((....)))..)))))...)))))).)))))...  []
```

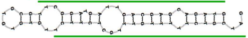

LOC_Os07g12850.1-2

	GSM520635	GSM693279	GSM520636	GSM520638	GSM686040	GSM520637	GSM455965	GSM571077	GSM329296	GSM520634	GSM693280	GSM571078	GSM686039	GSM520639	GSM520640	GSM278572	GSM278571	GSM455963	GSM329298	GSM329297	GSM309691	GSM409315	GSM278533	GSM455964	GSM409322	GSM409323	GSM309693	GSM409324	GSM409318	GSM409314	GSM409321	Total
GTAAGTCATTCTAGCATTTTCCA	0.49	0	0	0	0	0	0	0	0	0	0	0	0	0	0	0	0	0	0	0	0	0	0	0	0	0	0	0	0	0	0	0.49
GTAAGTCATTCTAGCATTTT	0.33	0	0.23	0.2	0	0	0	0	0	0	0	0	0	0	0	0	0	0	0	0	0	0	0	0	0	0	0	0	0	0	0	0.76
GTAAGTCATTCTAGCATTTTCCAT	0	0.1	0	0	0	0	0	0	0	0	0	0	0	0	0	0	0	0	0	0	0	0	0	0	0	0	0	0	0	0	0	0.1
TAAGTCATTCTAGCATTTTCCA	0	0	0	0	0.23	0.22	0.25	0	0	0	0.11	0	0	0	0	0	0	0	0	0	0	0	0	0	0	0	0	0	0	0	0	0.81
TAAGTCATTCTAGCATTTTCC	0	0.7	0	0.2	0.08	0	0.5	0	0	0	0.11	0	0	0	0	0	0	0	0	0	0	0	0	0	0	0	0	0	0	0	0	1.59
TAAGTCATTCTAGCATTTTCCATA	0.49	0.1	0	0	0	0	0	0	0	0	0	0	0	0	0	0	0	0	0	0	0	0	0	0	0	0	0	0	0	0	0	0.59
TAAGTCATTCTAGCATTTTC	0	0	0	0	0	0	0.25	0	4.07	0	0	0	0	0	0	0	0	0	0	0	0	0	0	0	0	0	0	0	0	0	0	4.32
TAAGTCATTCTAGCATTTT	0	0	0	0	0	0	0.5	0.26	0	0.37	0	0	0	0	0	0	0	0	0	0	0	0	0	0	0	0	0	0	0	0	0	1.13
AAGTCATTCTAGCATTTTCCATA	0	0	0.23	0	0	0	0	0	0	0	0	0	0	0	0	0	0	0	0	0	0	0	0	0	0	0	0	0	0	0	0	0.23
AAGTCATTCTAGCATTTTCCATAT	0.33	0.1	0	0	0	0	0	0	0	0.37	0	0	0.08	0	0	0	0	0	0	0	0	0	0	0	0	0	0	0	0	0	0	0.88
AAGTCATTCTAGCATTTTCCA	0	0	0	0.2	0	0	0	0	0	0	0	0.24	0	0	0	0	0	0	0	0	0	0	0	0	0	0	0	0	0	0	0	0.44
AGTCATTCTAGCATTTTCCAT	0	0.1	0	0	0	0	0	0	0	0	0	0	0	0	0	0	0	0	0	0	0	0	0	0	0	0	0	0	0	0	0	0.1
AGTCATTCTAGCATTTTCCATA	0	0	0	0	0.08	0	0	0	0	0	0	0	0.08	0	0	0	0	0	0	0	0	0	0	0	0	0	0	0	0	0	0	0.16
TCATTCTAGCATTTTCCAT	0	0	0	0.2	0	0	0	0	0	0	0	0	0	0	0	0	0	0	0	0	0	0	0	0	0	0	0	0	0	0	0	0.2
ATTAACATCAATATGTATGTGGGA	0	0	0.47	0.41	0	0.22	0.25	0	0	0.37	0	0	0	0.22	0.2	0	0	0	0	0	0	0	0	0	0	0	0	0	0	0	0	2.14
TTAACATCAATATGTATGTGG	0	0	0	0	0	0	0	0	0	0	0	0	0	0	0.2	0	0	0	0	0	0	0	0	0	0	0	0	0	0	0	0	0.2
TTAACATCAATATGTATGTGGGA	0	0	0	0	0	0	0	0	0	0	0	0	0	0	0.2	0	0	0	0	0	0	0	0	0	0	0	0	0	0	0	0	0.2
TTAACATCAATATGTATGTGGG	0	0	0	0.2	0	0	0	0	0	0	0	0	0	0	0	0	0	0	0	0	0	0	0	0	0	0	0	0	0	0	0	0.2
TTAACATCAATATGTATGT	0	0	0	0	0	0	0.25	0	0	0	0	0	0	0	0	0	0	0	0	0	0	0	0	0	0	0	0	0	0	0	0	0.25
TAACATCAATATGTATGTGGGAA	0	0	0	0	0	0	0	0	0	0	0	0	0	0	0.2	0	0	0	0	0	0	0	0	0	0	0	0	0	0	0	0	0.2
TAACATCAATATGTATGTGG	0	0	0	0	0	0	0	0	0	0	0	0	0	0	0.39	0	0	0	0	0	0	0	0	0	0	0	0	0	0	0	0	0.39
TAACATCAATATGTATGTGGGAAA	0	0	0	0	0	0	0	0	0	0	0	0	0	1.11	0.2	0	0	0	0	0	0	0	0	0	0	0	0	0	0	0	0	1.31
AACATCAATATGTATGTGGGAA	0	0	0	0.2	0	0	0	0	0	0	0	0	0	0	0	0	0	0	0	0	0	0	0	0	0	0	0	0	0	0	0	0.2
AACATCAATATGTATGTGGGAAAT	0	0	0	0	0	0	0	0	0	0.37	0	0	0	0	0	0	0	0	0	0	0	0	0	0	0	0	0	0	0	0	0	0.37
AACATCAATATGTATGTGGGA	0	0	0	0	0	0	0.25	0	0	0	0	0	0	0	0.2	0	0	0	0	0	0	0	0	0	0	0	0	0	0	0	0	0.45
AACATCAATATGTATGTGGG	0	0	0	0	0	0	0	0	0	0	0	0	0	0	0.2	0	0	0	0	0	0	0	0	0	0	0	0	0	0	0	0	0.2
ACATCAATATGTATGTGGGAA	0	0	0	0.41	0	0	0	0	0	0	0	0	0	0	1.18	0	0	0	0	0	0	0	0	0	0	0	0	0	0	0	0	1.59
ACATCAATATGTATGTGGGA	0	0	0	0.2	0	0	0	0	0	0	0	0	0	0.66	0	0	0	0	0	0	0	0	0	0	0	0	0	0	0	0	0	0.86
ATCAATATGTATGTGGGAAATGCT	0.16	0	0	0	0	1.09	0.25	0	0	1.84	0	0	0	0	0.2	0	0	0	0	0	0	0	0	0	0	0	0	0	0	0	0	3.54
ATCAATATGTATGTGGGAAAT	0	0	0	0.41	0	0	0	0	0	0	0	0	0	0	0	0	0	0	0	0	0	0	0	0	0	0	0	0	0	0	0	0.41
ATCAATATGTATGTGGGAAATG	0	0	0	0	0	0.22	0	0	0	0	0	0	0	0	0	0	0	0	0	0	0	0	0	0	0	0	0	0	0	0	0	0.22
TCAATATGTATGTGGGAAA	0	0	0	0	0	0.22	0	0	0	0	0	0	0	0	0	0	0	0	0	0	0	0	0	0	0	0	0	0	0	0	0	0.22
TCAATATGTATGTGGGAAATGCTA	0.16	0	0	0	0	0	0	0	0	0	0	0	0	0	0	0.33	0	0	0	0	0	0	0	0	0	0	0	0	0	0	0	0.49
CAATATGTATGTGGGAAATGCTA	0	0	0	0	0	0	0	0	0	0	0	0	0	0	0	0	0.31	0	0	0	0	0	0	0	0	0	0	0	0	0	0	0.31
CAATATGTATGTGGGAAATGCTAG	0	0.1	0	0	0	0	0	0	0	0	0	0	0	0	0	0	0	0	0	0	0	0	0	0	0	0	0	0	0	0	0	0.1
AATATGTATGTGGGAAATGCT	0	0	0	0.2	0	0	0	0	0	0	0	0	0	0.22	0.2	0	0	0	0	0	0	0	0	0	0	0	0	0	0	0	0	0.62
AATATGTATGTGGGAAATGCTA	0	0	0	0.41	0	0	0	0	0	0	0	0	0	0	0	0	0	0	0	0	0	0	0	0	0	0	0	0	0	0	0	0.41
ATATGTATGTGGGAAATGCTA	0	0	0	0	0	0	0	0	0	0	0	0	0	0	0.2	0.33	0	0	0	0	0	0	0	0	0	0	0	0	0	0	0	0.53
ATATGTATGTGGGAAATGCTAGAA	0	0	0.23	0	0	0	0	0.26	0	0	0	0	0	0	0	0	0	0	0	0	0	0	0	0	0	0	0	0	0	0	0	0.49
TATGTATGTGGGAAATGCTA	0	0.1	0	0	0	0	0	0	0	0	0	0	0	0	0	0	0	0	0	0	0	0	0	0	0	0	0	0	0	0	0	0.1
TATGTATGTGGGAAATGCTAGAATG	0.16	0	0	0	0	0	0	0	0	0	0	0	0	0	0	0	0	0	0	0	0	0	0	0	0	0	0	0	0	0	0	0.16
TATGTATGTGGGAAATGCT	0	0.2	0	0	0	0	0	0	0	0	0	0	0	0	0	0	0	0	0	0	0	0	0	0	0	0	0	0	0	0	0	0.2
ATGTATGTGGGAAATGCTAGAATG	0	0	0.23	0	0	0	0	0	0	0	0	0	0	0	0	0	0	0	0	0	0	0	0	0	0	0	0	0	0	0	0	0.23
ATGTATGTGGGAAATGCTAGA	0	0	0	0.41	0	0.22	0	0	0	0	0	0	0	0	0	0	0	0	0	0	0	0	0	0	0	0	0	0	0	0	0	0.63
ATGTATGTGGGAAATGCTAG	0	0	0	0	0	0	0	0	0	0	0	0	0	1.11	0	0	0	0	0	0	0	0	0	0	0	0	0	0	0	0	0	1.11
ATGTATGTGGGAAATGCTAGAA	0	0	0	0.2	0	0.22	0	0	0	0	0	0	0	0	0.2	0	0	0	0	0	0	0	0	0	0	0	0	0	0	0	0	0.62
TGTATGTGGGAAATGCTAGAA	0	0	0	0	0	0	0	0	0	0	0	0	0	0.22	0.2	0	0	0	0	0	0	0	0	0	0	0	0	0	0	0	0	0.42
TGTATGTGGGAAATGCTAGAATG	0	0	0	0	0	0	0	0	0	0	0	0	0	0	0.2	0	0	0	0	0	0	0	0	0	0	0	0	0	0	0	0	0.2
GTATGTGGGAAATGCTAGAATGAT	0	0	0	0	0	0	0	0	0	0	0	0	0.08	0	0	0	0	0	0	0	0	0	0	0	0	0	0	0	0	0	0	0.08
GTATGTGGGAAATGCTAGAATG	0	0	0	0	0	0	0	0	0	0	0	0	0	0	0.2	0	0	0	0	0	0	0	0	0	0	0	0	0	0	0	0	0.2
TATGTGGGAAATGCTAGAA	0	0	0	0	0	0	0	0	0	0	0	0	0	0	0	0	0	1.63	0	0	0	0	0	0	0	0	0	0	0	0	0	1.63
TATGTGGGAAATGCTAGAATG	0	0	0	0	0	0	0	0	0	0	0	0	0	0.22	0	0	0	0	0	0	0	0	0	0	0	0	0	0	0	0	0	0.22
ATGTGGGAAATGCTAGAATG	0	0	0.7	0.41	0	0.66	0	0	0	0	0	0	0	0	0.2	0	0	0	0	0	0	0	0	0	0	0	0	0	0	0	0	1.97
ATGTGGGAAATGCTAGAATGA	0.16	0	0.23	7.74	0	0.44	1.24	0	0	0	0	0	0	5.76	3.95	0	0	0	3.46	0	0	0	0	0	0	0	0	0	0	0	0	22.98
ATGTGGGAAATGCTAGAATGATTT	0.49	0	0	0.2	0	0	0.25	0	0	0.37	0	0	0	0	0	0	0	0	0	0	0	0	0	0	0	0	0	0	0	0	0	1.31
ATGTGGGAAATGCTAGAATGATT	0	0	0	0	0	0	0	0	0	0	0	0	0	0	0	0	0.31	0	0	0	0	0	0	0	0	0	0	0	0	0	0	0.31
TGTGGGAAATGCTAGAATGATT	0	0	0	0	0	0.22	0	0	0	0	0	0	0	0	0	0	0	0	0	0	0	0	0	0	0	0	0	0	0	0	0	0.22
TGTGGGAAATGCTAGAATG	0	0	0.7	0	0.08	0.22	0	0	0	0.37	0	0	0	0	0	0	0	0	0	0	0	0	0	0	0	0	0	0	0	0	0	1.37
TGTGGGAAATGCTAGAATGA	0	0	0	0	0	0.22	0	0	0	0	0	0	0.08	0.44	0	0	0	0	0	0	0	0	0	0	0	0	0	0	0	0	0	0.74
TGTGGGAAATGCTAGAATGATTT	0	0	0	0	0	0	0	0	0	0	0	0	0	0.22	0	0	0	0	0	0	0	0	0	0	0	0	0	0	0	0	0	0.22
GTGGGAAATGCTAGAATGATTTACA	0	0	0	0	0	0	0.25	0	0	0.37	0	0	0	0	0	0	0	0	0	0	0	0	0	0	0	0	0	0	0	0	0	0.62
GTGGGAAATGCTAGAATGATTTA	0	0	0	0	0.08	0	0	0	0	0	0	0	0	0	0	0	0	0	0	0	0	0	0	0	0	0	0	0	0	0	0	0.08
GTGGGAAATGCTAGAATGA	0	0	0	0	0.08	0.44	0	0	0	0	0	0	0.08	0	0.59	0	0	0	0	0	0	0	0	0	0	0	0	0	0	0	0	1.19
GTGGGAAATGCTAGAATGATTTAC	0.16	0	0	0	0	0	0.25	0	0	0	0	0	0.16	0	0	0	0	0	0	0	0	0	0	0	0	0	0	0	0	0	0	0.57
GTGGGAAATGCTAGAATGATTT	0	0	0	0	0	0	0	0	0	0	0	0	0.08	0	0	0	0	0	0	0	0	0	0	0	0	0	0	0	0	0	0	0.08
GTGGGAAATGCTAGAATGAT	0	0	0	0	0	0	0	0	0	0	0	0	0	0	0.2	0	0	0	0	0	0	0	0	0	0	0	0	0	0	0	0	0.2
GTGGGAAATGCTAGAATGATT	0	0	0	0.2	0	0	0	0	0	0	0	0	0	0	0	0	0	0	0	0	0	0	0	0	0	0	0	0	0	0	0	0.2
TGGGAAATGCTAGAATGATTT	0	0	0	1.63	0.08	0.44	0	0	0	0	0	0	0	0.44	0.39	0	0	0	0	0	0	0	0	0	0	0	0	0	0	0	0	2.98
TGGGAAATGCTAGAATGATT	0	0	0	0.81	0	0.44	0.25	0	0	0	0	0	0.08	0	0.79	0	0	0	0	0	0	0	0	0	0	0	0	0	0	0	0	2.37
TGGGAAATGCTAGAATGA	0	0.2	0	0	0	0	0	0	0	0	0	0	0.08	0	0	0	0	0	0	0	0	0	0	0	0	0	0	0	0	0	0	0.28
TGGGAAATGCTAGAATGATTTA	0	0	0	0.2	0	0.22	0	0	0	0	0	0	0	0.66	0	0	0	0	0	0	0	0	0	0	0	0	0	0	0	0	0	1.08
TGGGAAATGCTAGAATGAT	0	0	0	0.41	0	0	0	0	0	0	0	0	0	0	0	0	0	0	0	4.09	0	0	0	0	0	0	0	0	0	0	0	4.5
GGGAAATGCTAGAATGATTTACA	0	0	0	0	0	0	0.74	0	0	0	0	0	0	0	0	0	0	0	0	0	0	0	0	0	0	0	0	0	0	0	0	0.74
GGGAAATGCTAGAATGATTTACAT	4.11	0.1	0.23	0.41	0.08	0.22	3.47	0	0	0	0	0	0.23	1.33	0.79	0	0	0	0	0	0	0	0	0	0	0	0	0	0	0	0	10.97
GGGAAATGCTAGAATGATTT	0	0.1	0	0.2	0	0	0	0	0	0	0	0	0	0	0.2	0	0	0	0	0	0	0	0	0	0	0	0	0	0	0	0	0.5
GGGAAATGCTAGAATGATTTACATT	0	0	0	0	0	0	0.25	0	0	0	0	0	0	0	0	0	0	0	0	0	0	0	0	0	0	0	0	0	0	0	0	0.25
GGAAATGCTAGAATGATTTACATT	0	0	0	0	0	0.22	0.25	0	0	0	0	0	0	0	0	0	0	0	0	0	0	0	0	0	0	0	0	0	0	0	0	0.47
GAAATGCTAGAATGATTTACA	0	0	0	0.41	0	0	0.25	0	0	0	0	0	0	0.22	0	0	0	0	0	0	0	0	0	0	0	0	0	0	0	0	0	0.88
AAATGCTAGAATGATTTACA	0	0	0	0	0	0	0	0	0	0	0	0	0	0	0	0	0	0.81	0	0	0	0	0	0	0	0	0	0	0	0	0	0.81
AAATGCTAGAATGATTTAC	0	0	0	0	0	0	0	0	0	0	0	0	0	0	0	0	0	0	0	0	10.12	0	0	0	0	0	0	0	0	0	0	10.12
AAATGCTAGAATGATTTACAT	0	0	0	0.81	0.15	0	0	0.26	0	0	0	0	0.08	0	0.2	0	0	0	0	0	0	0	0	0	0	0	0	0	0	0	0	1.5
AAATGCTAGAATGATTTACATT	0	0	0	0.2	0.15	0	0	0	0	0	0	0	0.39	0	0	0	0	0	0	0	0	0	0	0	0	0	0	0	0	0	0	0.74
AAATGCTAGAATGATTTACATTGT	0	0.2	0.23	0.61	0	0.66	1.99	1.06	0	1.47	0.11	0.49	0.08	0	0.2	0	0	0	0	0	0	0	0	0	0	0	0	0	0	0	0	7.1
AAATGCTAGAATGATTTACATTG	0	0	0	0	0	0	0	0	0	0	0	0	0	0	0.2	0	0	0	0	0	0	0	0	0	0	0	0	0	0	0	0	0.2
AATGCTAGAATGATTTACATT	0	0	0	0.2	0	0	0.25	0	0	0.37	0	0	0	0	0	0	0	0	0	0	0	0	0	0	0	0	0	0	0	0	0	0.82
AATGCTAGAATGATTTACATTGTGA	0	0	0	0.2	0	0	0	0	0	0	0	0	0	0	0	0	0	0	0	0	0	0	0	0	0	0	0	0	0	0	0	0.2
AATGCTAGAATGATTTACATTGTG	0.82	0.3	2.81	0.2	0	1.75	4.72	0	0	5.14	0.43	0.98	0	0.44	0.99	0	0	0	0	0	0	0	0	0	0	0	0	0	0	0	0	18.58
AATGCTAGAATGATTTACATTG	0	0	0	0.2	0	0	0	0	0	0	0	0	0	0	0.2	0	0	0	0	0	0	0	0	0	0	0	0	0	0	0	0	0.4
AATGCTAGAATGATTTACA	0	0	0	0.2	0	0	0	0	0	0	0	0	0	0	0.2	0	0	0	0	0	0	0	0	0	0	0	0	0	0	0	0	0.4
AATGCTAGAATGATTTACAT	0	0	0	0	0	0	0	0	0	0	0	0.24	0	0	0	0	0	0	0	0	0	0	0	0	0	0	0	0	0	0	0	0.24
AATGCTAGAATGATTTACATTGT	0	0	0	0	0	0	0	0	0	0.37	0	0	0	0	0	0	0	0	0	0	0	0	0	0	0	0	0	0	0	0	0	0.37
ATGCTAGAATGATTTACATTGTG	0	0.1	0	0.2	0	0	0	0.26	0	0.37	0	0	0	0	0	0	0	0	0	0	0	0	0	0	0	0	0	0	0	0	0	0.93
ATGCTAGAATGATTTACATTGT	0	0.1	0	1.02	0	0	0.25	0	0	0	0	0	0	0	0	0	0	0	0	0	0	0	0	0	0	0	0	0	0	0	0	1.37
ATGCTAGAATGATTTACATTG	0	0.1	0	0	0	0	0	0.79	0	0	0.11	0.24	0	0	0	0	0	0	0	0	0	0	0	0	0	0	0	0	0	0	0	1.24
ATGCTAGAATGATTTACATTGTGA	0.16	0.6	0.23	0.2	0.08	0.66	0	2.38	0	2.2	0.32	0.73	0	0.22	1.38	0	0	0	0	0	0	0	0	0	0	0	0	0	0	0	0	9.16
ATGCTAGAATGATTTACATT	0	0.1	0	0	0	0	0	0.26	0	0	0	0	0	0	0	0	0	0	0	0	0	0	0	0	0	0	0	0	0	0	0	0.36
ATGCTAGAATGATTTACAT	0	0	0	0.2	0	0	0	0	0	0	0	0	0	0.22	0	0	0	0	0	0	0	0	0	0	0	0	0	0	0	0	0	0.42
TGCTAGAATGATTTACATTG	0	0	0	0	0	0	0	0	0	0	0	0	0	0	0.39	0	0	0	0	0	0	0	0	0	0	0	0	0	0	0	0	0.39
TGCTAGAATGATTTACATTGTG	0	0	0	0.2	0	0	0	0	0	0	0	0	0	0	0	0	0	0	0	0	0	0	0	0	0	0	0	0	0	0	0	0.2
TGCTAGAATGATTTACATTGT	0	0	0	1.22	0	0	0.74	0	0	0	0	0	0	0.22	0	0	0	0	0	0	0	0	0	0	0	0	0	0	0	0	0	2.18
TGCTAGAATGATTTACATTGTGAA	0	0	0	0	0	0	0.5	0	0	0	0	0	0	0	0	0	0	0	0	0	0	0	0	0	0	0	0	0	0	0	0	0.5
GCTAGAATGATTTACATTGTGAAA	1.15	0	0	0	0	0	0.74	0	0	0.37	0	0	0	0	0	0	0	0	0	0	0	8.87	0	0	0	0	0	0	0	0	0	11.13
GCTAGAATGATTTACATTGTG	0	0	0	0	0	0	0	0	0	0	0	0	0	0	0.2	0	0	0	0	0	0	0	0	0	0	0	0	0	0	0	0	0.2
GCTAGAATGATTTACATTGTGAA	0	0	0	0	0	0	0.25	0	0	0	0	0	0	0	0	0	0	0	0	0	0	0	0	0	0	0	0	0	0	0	0	0.25
CTAGAATGATTTACATTGTGA	0	0	0.23	15.06	0	0.44	3.23	0	0	0	0.11	0	0.08	6.2	6.91	0	0	0	0	0	0	0	0	0	0	0	0	0	0	0	0	32.26
CTAGAATGATTTACATTGT	0	0	0	0.2	0	0	0	0	0	0	0	0	0	0	0	0	0	0	0	0	0	0	0	0	0	0	0	0	0	0	0	0.2
CTAGAATGATTTACATTGTG	0	0	0	0.2	0	0.22	0.25	0	0	0	0	0	0	0	0.39	0	0	0	0	0	0	0	0	0	0	0	0	0	0	0	0	1.06
CTAGAATGATTTACATTGTGAAAC	0	0	0	0	0	0	0	0	0	0	0	0	0	0	0.2	0	0	0	0	0	0	0	19.37	0	0	0	0	0	0	0	0	19.57
CTAGAATGATTTACATTGTGAAA	0	0	0	0	0	0	0	0	0	0	0	0	0	0	0	0	0	0	0	0	0	0	19.37	0	0	0	0	0	0	0	0	19.37
CTAGAATGATTTACATTGTGAAACG	0	0	0	0	0	0	0.25	0	0	0	0	0	0	0	0	0	0	0	0	0	0	0	0	0	0	0	0	0	0	0	0	0.25
TAGAATGATTTACATTGTGAAA	0	0	0	0.2	0	0	0	0	0	0	0	0	0	0	0	0	0	0	0	0	0	0	0	0	0	0	0	0	0	0	0	0.2
TAGAATGATTTACATTGTG	0	0	0	0.41	0	0	0.99	0	0	0	0	0	0	0.44	0.39	0	0	0	0	0	0	0	0	0	0	0	0	0	0	0	0	2.23
TAGAATGATTTACATTGTGA	0	0	0	6.72	0	1.09	3.72	0	0	0	0	0	0	4.65	9.48	0	0	0	0	0	0	0	0	6.42	0	0	0	0	0	0	0	32.08
TAGAATGATTTACATTGTGAA	0	0	0	0.81	0.08	0	0.99	0	0	0	0	0	0	0.66	0.99	0	0	0	0	0	0	0	0	0	11.76	0	0	0	0	0	0	15.29
AGAATGATTTACATTGTGA	0	0	0.23	0	0	0	0	0	0	0.37	0	0	0	0	0	0	0	0	0	0	0	0	0	0	0	0	0	0	0	0	0	0.6
AGAATGATTTACATTGTGAAACGGA	0	0	0	0.2	0	0	0	0	0	0	0	0	0	0	0.2	0	0	0	0	0	0	0	0	0	0	0	6.05	0	0	0	0	6.45
AGAATGATTTACATTGTGAAACGG	1.64	0.9	0.94	1.43	1.28	3.5	0.74	2.38	0	1.1	0.43	0.49	1.95	0.89	2.96	0.33	0	0	0	0	0	8.87	0	0	0	15.26	0	18.07	8.76	0	0	71.92
AGAATGATTTACATTGTG	0	0	0	0	0	0	0	0	0	0	0	0	0	0	0	0	0	0	0	0	0	0	0	0	0	0	6.05	0	0	0	0	6.05
AGAATGATTTACATTGTGAAA	0	0	0	0.81	0	0	0.25	0	0	0	0	0	0	0.66	0	0	0	0	0	0	0	0	0	0	0	0	0	0	0	0	0	1.72
AGAATGATTTACATTGTGAAAC	0	0	0	0	0	0	0	0	0	0	0	0	0	0	0	0	0	0	0	0	0	0	0	0	0	0	6.05	0	0	0	0	6.05
AGAATGATTTACATTGTGAAACG	0	0	0	0	0	0	0	0	0	0	0	0	0.08	0	0	0	0	0	0	0	0	0	0	0	0	0	0	0	0	0	0	0.08
GAATGATTTACATTGTGAAAC	0	0	0	0	0	0	0	0	0	0	0	0	0	0	0	0	0	0	0	0	0	0	0	0	0	0	6.05	0	0	0	0	6.05
GAATGATTTACATTGTGAAA	0	0	0	0	0	0	0	0	0	0	0	0	0	0	0	0	0	0	0	0	0	0	0	0	0	0	6.05	0	0	0	0	6.05
GAATGATTTACATTGTGAAACGGA	1.32	1	0	0	0.75	0.87	0	3.43	0	0	0.22	1.71	1.41	0.22	0.39	0.33	0	0	0	0	0	0	0	0	0	0	0	0	0	0	0	11.65
GAATGATTTACATTGTGAAACGG	0	0	0	0	0	0	0	0	0	0	0	0.24	0	0	0	0	0	0	0	0	0	8.87	0	0	0	0	0	0	0	0	0	9.11
AATGATTTACATTGTGAAACGGAG	0.82	0.9	21.04	0.61	0.08	5.68	1.24	1.06	0	4.41	0.65	2.2	1.17	2.21	3.75	0.67	0	0	0	0	0	0	0	0	0	0	0	0	0	0	0	46.49
AATGATTTACATTGTGAAACGG	0	0	0.23	0.2	0	0	0	0	0	0	0	0	0	0	0	0	0	0	0	0	0	0	0	0	0	0	0	0	0	0	0	0.43
AATGATTTACATTGTGAAACGGA	0	0	0.23	0	0	0.22	0.74	1.32	0	0.37	0	0.24	0.16	0	0	0	0.31	0	0	0	0	0	0	0	0	0	0	0	0	0	0	3.59
ATGATTTACATTGTGAAACGGAGG	1.64	0.1	8.18	1.83	0	4.59	1.24	1.32	0	2.57	0	0.73	0	0.44	4.15	0.33	0	0	0	0	0	0	0	0	0	0	0	0	0	0	0	27.12
ATGATTTACATTGTGAAACGGAG	0	0.1	0.7	1.22	0.08	1.97	0	0	0	0.37	0	0.24	0.08	0	0.99	0	0	0	0	0	0	0	0	0	0	0	0	0	0	0	0	5.75
ATGATTTACATTGTGAAACGG	0	0.1	0	0.41	0.15	0.22	0	0	0	0	0	0	0	0	0.2	0	0	0	0	0	0	0	0	0	0	0	0	0	0	0	0	1.08
ATGATTTACATTGTGAAACGGA	0	0	0	1.22	0	0	0	0	0	0	0	0.49	0.16	0	0	0	0	0	0	0	0	0	0	0	0	0	0	0	0	0	0	1.87
TGATTTACATTGTGAAACGGAG	0	0.1	0	0.81	0	0	0	0	0	0	0	0	0.23	0	0	0	0	0	0	0	0	0	0	0	0	0	0	0	0	0	0	1.14
TGATTTACATTGTGAAACGGA	0	0.1	0	0.2	0.08	0.22	0	0	0	0	0	0	0.47	0	0.39	0	0	0	0	0	0	0	0	0	0	0	0	0	0	0	0	1.46
TGATTTACATTGTGAAACG	0	0.1	0	0	0	0	0	0	0	0	0.11	0	0	0	0	0	0	0	0	0	0	0	0	0	0	0	0	0	0	0	0	0.21
TGATTTACATTGTGAAACGGAGG	0	0	0	0	0	0.66	0	0.26	0	0	0	0	0	0	0	0	0	0	0	0	0	0	0	0	0	0	0	0	0	0	0	0.92
TGATTTACATTGTGAAACGGAGGG	0.33	0	0	0	0	0.44	0.25	0	0	0	0	0	0.08	0	0	0	0	0	0	0	0	0	0	0	0	0	0	0	0	0	0	1.1
TGATTTACATTGTGAAACGGAGGGA	0.16	0	0	0	0	0	0	0	0	0	0	0	0	0	0	0	0	0	0	0	0	0	0	0	0	0	0	0	0	0	0	0.16
GATTTACATTGTGAAACGGAGGGA	0.49	0.1	0.23	0	0.08	0.66	0.74	0.26	0	0	0	0.49	0.23	0	0.2	0	0	0	0	0	0	0	0	0	0	0	0	0	0	0	0	3.48
GATTTACATTGTGAAACGGAGGG	0.16	0	0	0	0	0.44	0.25	0	0	0	0	0.24	0	0	0.2	0	0	0	0	0	0	0	0	0	0	0	0	0	0	0	0	1.29
GATTTACATTGTGAAACGGAGG	0	0.1	0	0.2	0	0	0	0	0	0	0	0	0	0	0	0	0	0	0	0	0	0	0	0	0	0	0	0	0	0	0	0.3
GATTTACATTGTGAAACGGAG	0	0.5	0.23	0.2	0.15	0.22	0	0	0	0	0.32	0	0	0	0.2	0	0	0	0	0	0	0	0	0	0	0	0	0	0	0	0	1.82
GATTTACATTGTGAAACGGA	0	0.1	0	0	0	0	0	0	0	0	0	0	0.08	0	0	0	0	0	0	0	0	0	0	0	0	0	0	0	0	0	0	0.18
GATTTACATTGTGAAACGG	0	0.1	0	0	0	0	0	0	0	0	0	0	0	0	0	0	0	0	0	0	0	0	0	0	0	0	0	0	0	0	0	0.1
ATTTACATTGTGAAACGGAG	0	0	0	0.2	0	0.44	0	0	0	0	0	0	0	0	0	0	0	0	0	0	0	0	0	0	0	0	0	0	0	0	0	0.64
ATTTACATTGTGAAACGGAGGGAG	0	0.1	0.23	0	0	0.22	0.25	0	0	0	0.11	0	0	0	0	0	0	0	0	0	0	0	0	0	0	0	0	0	0	0	0	0.91
ATTTACATTGTGAAACGGAGG	0	0	0	0.81	0	0	0	0	0	0	0	0	0	1.33	1.38	0	0	0	0	0	0	0	0	0	0	0	0	0	0	0	0	3.52
ATTTACATTGTGAAACGGAGGGA	0.33	0	0	0	0	0	0	0	0	0	0	0	0	0	0.2	0	0	0	0	0	0	0	0	0	0	0	0	0	0	0	0	0.53
ATTTACATTGTGAAACGGA	0	0	0	0	0	0.22	0	0	0	0	0	0	0	0	0	0	0	0	0	0	0	0	0	0	0	0	0	0	0	0	0	0.22
TTTACATTGTGAAACGGAGGG	0	0	0	0.81	0	0	0	0	0	0	0.11	0.24	0	0.22	0	0	0.31	0	0	0	0	0	0	0	0	0	0	0	0	0	0	1.69
TTTACATTGTGAAACGGAGGGAGT	0	0.1	0	0	0.08	0.22	0	0.53	0	0	0	0.24	0.08	0	0	0.33	0	0	0	0	0	0	0	0	0	0	0	0	0	0	0	1.58
TTTACATTGTGAAACGGAGGGA	0	0	0	0	0	0.22	0	0	0	0	0	0	0	0	0.2	0	0	0	0	0	0	0	0	0	0	0	0	0	0	0	0	0.42
TTTACATTGTGAAACGGAGGGAG	0	0	0	0	0	0	0	0	0	0	0	0	0	0	0.2	0	0	0	0	0	0	0	0	0	0	0	0	0	0	0	0	0.2
TTTACATTGTGAAACGGAGG	0	0	0	0.2	0	0	0	0	0	0	0	0	0	0	0.2	0	0	0	0	0	0	0	0	0	0	0	0	0	0	0	0	0.4
TTTACATTGTGAAACGGAG	0	0	0	0.41	0	0	0	0	0	0	0	0	0	0	0.39	0	0	0	0	0	0	0	0	0	0	0	0	0	0	0	0	0.8
TTACATTGTGAAACGGAGG	0	0	0	1.43	0	0.66	0.25	0	0	0	0	0	0.08	0.44	0.2	0	0	0	0	0	0	0	0	0	0	0	0	0	0	0	0	3.06
TTACATTGTGAAACGGAGGGAG	0	0.1	0	1.43	0.08	0.66	0	0.79	0	0	0.11	0	0.08	0	0	0.67	0	0	0	0	0	0	0	0	0	0	0	0	0	0	0	3.92
TTACATTGTGAAACGGAGGGAGT	0	0.4	0	1.22	0	0.22	0.25	0.79	0	0	0.43	0.24	0.08	0	0.2	1	0	0	0	0	0	0	0	0	0	0	0	0	0	0	0	4.83
TTACATTGTGAAACGGAGGGAGTA	0.16	0.8	0	0	0	0.87	0	0.79	0	0	0.43	0	0	0.66	0	2	1.23	0	0	4.09	0	0	0	0	0	0	0	0	0	16.03	0	27.06
TTACATTGTGAAACGGAGGGA	0	0	0	2.44	0	0	0.74	0.26	0	0	0	0.24	0.16	0.22	0.39	0.33	0	0	0	0	0	8.87	0	0	0	0	0	0	0	0	0	13.65
TTACATTGTGAAACGGAGGG	0	0.1	0	1.83	0.15	0	0.25	0.26	0	0	0	0.24	0.23	0	0	1	0	0	0	0	0	0	0	0	0	0	0	0	0	0	0	4.06
TTACATTGTGAAACGGAG	0	0	0	0	0.15	0	0	0	0	0	0	0	0.08	0	0	0.33	0	0	0	8.19	0	0	0	0	0	0	0	0	0	0	0	8.75
TACATTGTGAAACGGAGGGAGTACT	0	0	0	0.2	0	0.22	0	0	0	0	0	0	0	0	0.39	0	0.31	0	0	0	0	0	0	0	0	0	0	0	0	0	0	1.12
TACATTGTGAAACGGAGGGAGTA	0	0.6	0	0	0	0.44	0.25	0.53	0	0	0.65	0.49	0.23	0	0.2	0.33	0	0	0	0	0	0	0	0	11.76	0	0	0	0	0	7.33	22.81
TACATTGTGAAACGGAGGGAGT	0	0.1	0	1.22	0	0.22	0	0.26	0	0	0.32	0	0.23	0.66	0.39	0.33	0	0	0	0	0	0	0	0	0	0	0	0	0	0	0	3.73
TACATTGTGAAACGGAGGGAG	0	0	0	0.61	0	0.22	0	0.26	0	0	0	0	0	0.44	0.39	0.33	0	0	0	0	0	0	0	0	0	0	0	18.07	0	0	0	20.32
TACATTGTGAAACGGAGGG	0	0.6	0	0.61	0	0.22	0	0	0	0.37	0.32	0	0	0	0.2	0	0	0	0	0	0	0	0	0	0	0	0	0	0	0	0	2.32
TACATTGTGAAACGGAGGGA	0.16	0	0	0.2	0	0.22	0	0	0	0	0	0	0.08	0	0.59	0	0	0	0	0	0	0	0	0	0	0	0	0	0	0	0	1.25
TACATTGTGAAACGGAGGGAGTAC	0	0.3	0	0	0	0.22	0	0.53	0	0	0.54	0	0	0	0.2	0.33	0.31	0	0	0	0	0	0	0	0	0	0	0	0	0	7.33	9.76
TACATTGTGAAACGGAGG	0	0.1	0	0	0.08	0	0	0	0	0	0	0	0.16	0	0	0.67	0	0	0	0	0	0	0	0	0	0	0	0	0	0	0	1.01
ACATTGTGAAACGGAGGGAGTA	0	0	0	1.43	0	0	0.25	0.26	0	0	0	0	0	0	0.2	0.33	0	0	0	0	0	0	0	0	0	0	0	0	0	0	0	2.47
ACATTGTGAAACGGAGGGAGT	0	0.1	0	2.24	0	0	0	0.26	0	0	0.22	0.24	0.08	0.89	0.99	1	0.61	0	0	0	0	0	0	0	0	0	0	0	0	0	0	6.63
ACATTGTGAAACGGAGGGAGTAC	0	0.1	0	0	0	0	0	0.53	0	0	0.22	0	0	0	0	0	0	0	0	0	0	0	0	0	0	0	0	0	0	0	0	0.85
ACATTGTGAAACGGAGGGAG	0	0.2	0	0.41	0	0.22	0	0	0	0	0.11	0	0	0	0	0	0.61	0	0	0	0	0	0	0	0	0	0	0	0	0	0	1.55
ACATTGTGAAACGGAGGGA	0	0.3	0	0	0	0	0	0	0	0	0.11	0	0	0	0	0	0	0	0	0	0	0	0	0	0	0	0	0	0	0	0	0.41
ACATTGTGAAACGGAGGGAGTACT	0.16	0	0	0	0	0	0	0	0	0.37	0.11	0	0	0	0.2	0	0	0	0	0	0	0	0	0	0	0	0	0	0	0	0	0.84
ACATTGTGAAACGGAGGG	0	0.6	0	0	0	0	0	0.26	0	0	0	0	0.08	0	0	0	0	0	0	0	0	0	0	0	0	0	0	0	0	0	0	0.94
CATTGTGAAACGGAGGGAGT	0	0.1	0	0.2	0	0	0.25	0	0	0	0.22	0.24	0	1.11	0.2	0	0	0	0	0	0	0	0	0	0	0	0	0	0	0	0	2.32
CATTGTGAAACGGAGGGAGTAC	0	0.1	0	0.41	0	0	0	0	0	0	0.11	0	0	0	0	0	0	0	0	0	0	0	0	0	0	0	0	0	0	0	0	0.62
CATTGTGAAACGGAGGGAGTA	0	0	0	0.41	0	0	0	0	0	0	0	0	0	0	0.2	0.33	0	0	0	0	0	0	0	0	0	0	0	0	0	0	0	0.94
CATTGTGAAACGGAGGGAGTACT	0	0	0	0	0	0	0	0	0	0	0	0.24	0	0	0	0	0	0	0	0	0	0	0	0	0	0	0	0	0	0	0	0.24
CATTGTGAAACGGAGGGA	0	0.3	0	0	0	0	0	0	0	0	0	0	0	0	0	0	0	0	0	0	0	0	0	0	0	0	0	0	0	0	0	0.3
CATTGTGAAACGGAGGGAG	0	0.1	0	0	0	0	0	0.26	0	0	0	0	0	0	0	0	0	0	0	0	0	0	0	0	0	0	0	0	0	0	0	0.36
ATTGTGAAACGGAGGGAGT	0	0	0	0.41	0	0	0	0	0	0	0	0.24	0.08	0	0	0	0	0	0	0	0	0	0	0	0	0	0	0	0	0	0	0.73
ATTGTGAAACGGAGGGAGTA	0	0	0	0	0	0	0	0.26	0	0	0	0	0.08	0	0.2	0	0.31	0	0	0	0	0	0	0	0	0	0	0	0	0	0	0.85
ATTGTGAAACGGAGGGAGTAC	0	0	0	0	0	0	0	0.26	0	0	0	0	0	0	0	0	0	0	0	0	0	0	0	0	0	0	0	0	0	0	0	0.26
ATTGTGAAACGGAGGGAG	0	0.1	0	0	0	0	0	0	0	0	0	0	0.16	0	0	0	0	0	0	0	0	0	0	0	0	0	0	0	0	0	0	0.26
ATTGTGAAACGGAGGGAGTACTA	0.16	0	0	0	0	0	0	0	0	0	0	0	0	0	0	0	0	0	0	0	0	0	0	0	0	0	0	0	0	0	0	0.16
ATTGTGAAACGGAGGGAGTACT	0	0	0	0.2	0	0	0	0	0	0	0.11	0	0	0	0	0	0	0	0	0	0	0	0	0	0	0	0	0	0	0	0	0.31
TTGTGAAACGGAGGGAGTACT	0	0.1	0	0.61	0	0	0.25	0	0	0	0	0	0.08	0.44	0.2	0	0	0	0	0	0	0	0	0	0	0	0	0	0	0	0	1.68
TTGTGAAACGGAGGGAGTA	0	0.4	0	0	0	0	0	0.53	0	0	0	0	0.08	0	0	0.33	0.61	0	0	0	0	0	0	0	0	0	0	0	0	0	0	1.95
TTGTGAAACGGAGGGAGTACTATT	0	0	0	0	0	0	0	0	0	0	0	0	0.08	0	0	0	0	0	0	0	0	0	0	0	0	0	0	0	0	0	0	0.08
TTGTGAAACGGAGGGAGT	0	0	0	0	0	0	0	0.26	0	0	0	0	0	0	0	0	0	0	0	0	0	0	0	0	0	0	0	0	0	0	0	0.26
TTGTGAAACGGAGGGAGTAC	0	0	0	0.2	0	0	0	0	0	0	0	0	0	0	0	0	0	0	0	0	0	0	0	0	0	0	0	0	0	0	0	0.2
TGTGAAACGGAGGGAGTA	0	0.7	0	0	0	0	0	0	0	0	0.22	0	0.08	0	0	0.67	0	0	0	0	0	0	0	0	0	0	0	0	0	0	0	1.67
TGTGAAACGGAGGGAGTACT	0	0	0	0	0	0	0.25	0	0	0	0	0	0	0	0	0	0	0	0	0	0	0	0	0	0	0	0	0	0	0	0	0.25
GTGAAACGGAGGGAGTACTA	0.16	0	0	0	0	0	0	0	0	0	0	0	0	0	0	0	0	0	0	0	0	0	0	0	0	0	0	0	0	0	0	0.16
TGAAACGGAGGGAGTACT	0	0	0	0	0	0	0	0	0	0	0	0	0	0	0	0.33	0	0	0	0	0	0	0	0	0	0	0	0	0	0	0	0.33
TGAAACGGAGGGAGTACTATTTAA	0	0	0	0	0	0	0	0	0	0	0	0	0.08	0	0	0	0	0	0	0	0	0	0	0	0	0	0	0	0	0	0	0.08
TGAAACGGAGGGAGTACTATT	0	0	0	0	0	0.22	0	0	0	0	0	0	0	0	0	0	0	0	0	0	0	0	0	0	0	0	0	0	0	0	0	0.22

GTAAGTCATTCTAGCATTTTCCATATATATATTAACATCAATATGTATGTGGGAAATGCTAGAATGATTTACATTGTGAAACGGAGGGAGTACTATTTAATAG

-45.60 (((((((((((((((((((((((((((((((((......))))))))))))))))))))))))))))))))))............................... []

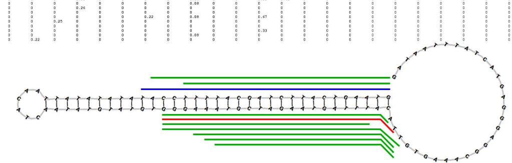

LOC_Os07g32220.1-5

	GSM520635	GSM455965	GSM693280	GSM520634	GSM520640	GSM686039	GSM520637	GSM693279	GSM278572	GSM278571	GSM520636	GSM520638	Total
GTTGGTGGTCTCAATTGTGATAGA	0.16	0	0	0	0	0	0	0	0	0	0	0	0.16
TGGTGGTCTCAATTGTGATAGACT	0	0.25	0	0	0	0	0	0	0	0	0	0	0.25
GTGGTCTCAATTGTGATAGACTAA	0	0	0.11	0	0	0	0	0	0	0	0	0	0.11
ATTTTAGTACTTATATATGATCGA	0	0	0.32	0.37	0.2	0.08	0	0	0	0	0	0	0.97
TTTTAGTACTTATATATGATCGA	0	0	0.11	0	0	0	0	0	0	0	0	0	0.11
TTTAGTACTTATATATGATCG	0	0	0	0	0	0	0.22	0	0	0	0	0	0.22
TTAGTACTTATATATGATCGAT	0	0	0	0	0	0	0.22	0	0	0	0	0	0.22
TAGTACTTATATATGATCGATCAA	0	0	0.65	0	0	0	0	0.3	0	0	0	0	0.95
AGTACTTATATATGATCGATCAAC	0	0	0.32	0	0	0	0	0	0	0	0	0	0.32
TACTTATATATGATCGATCAACT	0	0	0.11	0	0	0	0	0	0	0	0	0	0.11
ACTTATATATGATCGATCAACTAA	0	0	0.22	0	0	0	0	0.1	0.67	0	0	0	0.99
ACTTATATATGATCGATCAACTAAT	0	0	0	0	0	0	0	0	0	0.31	0	0	0.31
ACTTATATATGATCGATCAACTA	0	0	0.11	0	0	0	0	0	0	0	0	0	0.11
TTATATATGATCGATCAACTAA	0	0	0	0	0	0	0	0.1	0	0	0	0	0.1
TTATATATGATCGATCAACTAATC	0	0	0.11	0	0	0	0	0	0.67	0	0	0	0.78
ATATATGATCGATCAACTAATC	0	0	0.97	0	0	0	0	0.4	0	0	0	0	1.37
ATATATGATCGATCAACTAATCT	0	0	0.11	0	0	0	0	0	0	0	0	0	0.11
ATATATGATCGATCAACTA	0	0	0	0	0	0	0	0	0.33	0	0	0	0.33
ATATATGATCGATCAACTAAT	0	0	0.11	0	0	0	0	0	0	0	0	0	0.11
ATATATGATCGATCAACTAA	0	0	0	0	0	0	0	0	0	0.31	0	0	0.31
TATATGATCGATCAACTAATCT	0	0	0.11	0	0	0	0	0	0	0	0	0	0.11
TATATGATCGATCAACTAATC	0	0	0.75	0	0	0	0	0.2	0	0	0	0	0.95
TATATGATCGATCAACTAATCTGG	0	0	0.11	0	0	0	0	0	0	0	0	0	0.11
ATATGATCGATCAACTAATCTGGAA	0	0.25	0	0	0	0	0	0	0	0	0	0	0.25
ATATGATCGATCAACTAATCTGGA	0	0.25	0	0	0	0	0	0	0	0	0	0	0.25
ATATGATCGATCAACTAATC	0	0	1.72	0	0	0	0	0.7	0	0	0	0	2.42
ATATGATCGATCAACTAATCT	0	0	0	0	0	0.08	0	0	0	0	0	0	0.08
TATGATCGATCAACTAATC	0	0	0.22	0	0	0	0	0.5	0	0	0	0	0.72
TATGATCGATCAACTAATCTGGAA	0	0	0	0	0	0	0.66	0	0	0	0	0	0.66
ATGATCGATCAACTAATCTGGAAT	0.16	0	0	0	0	0	0	0	0	0	0.23	0	0.39
GATCAACTAATCTGGAATTATGAC	0	0	0	0.37	0	0	0	0	0	0	0	0	0.37
ATCAACTAATCTGGAATTATGACA	0	0	0.11	0	0	0	0	0	0	0	0	0	0.11
TCAACTAATCTGGAATTATGA	0	0.25	0	0	0	0	0	0	0	0	0	0	0.25
TCAACTAATCTGGAATTATGAC	0	0	0	0	0	0	0	0	0	0	0	0.41	0.41
ACTAATCTGGAATTATGACAATGT	0	0	0.11	0.73	0.2	0	0.22	0	0	0	0	0	1.26

GTTGGTGGTCTCAATTGTGATAGACTAACTATATTTTAGTACTTATATATGATCGATCAACTAATCTGGAATTATGACAATGTAG

-13.20 ((((((((((.....(((((...(((((.......)))))..)))))...)))).))))))......................... []

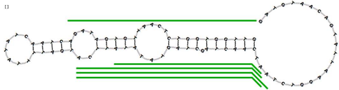

LOC_Os08g04190.1-1 (osa-MIR1429)

	GSM455965	GSM571078	GSM686039	GSM520634	GSM571077	GSM686040	GSM409323	GSM409322	GSM409320	GSM520640	GSM520637	GSM520638	GSM520639	GSM520635	GSM520636	GSM409319	GSM693279	GSM329297	GSM693280	GSM329298	GSM278571	GSM278572	GSM278532	GSM329296	GSM409316	GSM409317	Run1	GSM407071	GSM329299	GSM409318	GSM407072	Run2	GSM409324	GSM309691	GSM278533	GSM455963	GSM455962	GSM455964	Total
GTAATATACTAATCCGTGCATCCA	81.15	3.9	2.58	3.3	10.29	4.67	0	35.29	0	29.62	24.26	0.61	24.13	266.46	6.31	13.71	5.78	12.28	7.97	10.38	1.54	1.67	70.37	40.73	39.12	42.11	0	0	0	0	0	0	0	0	0	0	0	0	738.23
GTAATATACTAATCCGTGCATC	0.5	0.24	0.23	0	0.26	0.23	15.26	0	0	0	0.22	0.2	0	0.33	0	0	0.2	0	0	0	0	0	0	0	0	0	0	0	0	0	0	0	0	0	0	0	0	0	17.67
GTAATATACTAATCCGTGCA	0	0	0.16	0	0.26	0	0	0	0	0.2	0	0	0.22	0.33	0	0	0.2	0	0	0	0	0	0	0	0	0	0	0	0	0	0	0	0	0	0	0	0	0	1.37
GTAATATACTAATCCGTG	0	0	0.08	0	0	0	0	0	0	0	0	0	0	0	0	0	0	0	0	0	0	0	0	0	0	0	0	0	0	0	0	0	0	0	0	0	0	0	0.08
GTAATATACTAATCCGTGCAT	0	0	0.08	0.37	0.26	0.08	0	0	0	0	0	0	0	0.16	0	0	0	0	0.11	0	0	0	0	0	0	0	0	0	0	0	0	0	0	0	0	0	0	0	1.06
GTAATATACTAATCCGTGCATCCAT	0.25	0	0	0	0	0	0	0	0	0.2	0	0	0	0	0	0	0	0	0	0	0	0	0	0	0	0	0	0	0	0	0	0	0	0	0	0	0	0	0.45
GTAATATACTAATCCGTGCATCC	0.25	0	0	0	0.26	0.08	0	0	36.87	0	0.22	0.2	0	0.49	0	0	0	0	0.11	3.46	0	0	0	4.07	0	0	0	0	0	0	0	0	0	0	0	0	0	0	46.01
AATATACTAATCCGTGCATCCA	0	0	0	0	0	0	0	0	0	0	0.22	0	0	0.82	0	0	0	0	0	0	0	0	0	0	0	0	0	0	0	0	0	0	0	0	0	0	0	0	1.04
ATATACTAATCCGTGCATCCA	0	0	0.08	0	0	0	0	0	0	0.2	0.66	0	0.44	0	0	0	0.1	0	0	0	0	0	0	0	0	0	0	0	0	0	0	0	0	0	0	0	0	0	1.48
TATACTAATCCGTGCATCCA	0.25	0	0	0	0	0	0	0	0	0	0	0	0	0.16	0	0	0	0	0	0	0	0	0	0	0	0	0	0	0	0	0	0	0	0	0	0	0	0	0.41
ATACTAATCCGTGCATCCA	0	0	0	0	0	0	0	0	0	0	0.22	0	0.22	0.33	0	0	0	0	0	0	0	0	0	0	0	0	0	0	0	0	0	0	0	0	0	0	0	0	0.77
ACGTCATGTGAACGTGCATCGGTT	0	0	0	0	0	0	0	0	0	0	0	0	0	0.16	0	0	0	0	0	0	0.31	0	0	0	0	0	0	0	0	0	0	0	0	0	0	0	0	0	0.47
CGTCATGTGAACGTGCATCGGT	0	0	0	0	0	0	0	0	0	0	0.22	0	0	0	0	0	0	0	0	0	0	0	0	0	0	0	0	0	0	0	0	0	0	0	0	0	0	0	0.22
ACGTGCATCGGTTCATAATGTTGT	0.25	0	0	0	0	0	0	0	0	0	0	0	0	0	0	0	0	0	0	0	0	0	0	0	0	0	0	0	0	0	0	0	0	0	0	0	0	0	0.25
TGGTTGCACGGGTTTGTATGTTGCA	0	0	0	0	0	0	0	0	0	0	0	0.2	0	0	0	0	0	0	0	0	0	0	0	0	0	0	0	0	0	0	0	0	0	0	0	0	0	0	0.2
GGTTGCACGGGTTTGTAT	0	0	0	0	0.26	0	0	0	0	0	0	0	0	0	0	0	0	0	0	0	0	0	0	0	0	0	0	0	0	0	0	0	0	0	0	0	0	0	0.26
GTTGCACGGGTTTGTATGT	0	0	0.08	0	0	0	0	0	0	0.2	0.44	0	0	1.15	0	0	0.5	0	0.11	0	0	0	0	0	0	0	0	0	0	0	0	0	0	0	0	0	0	0	2.48
GTTGCACGGGTTTGTATGTTGC	0.99	0	0	0	0	0	0	0	0	1.78	0.87	0	1.77	0.99	0	0	0.1	0	0.11	0	0	0	0	0	0	0	0	0	0	0	0	0	0	0	0	0	0	0	6.61
GTTGCACGGGTTTGTATG	0	0	0.16	0	0	0.15	0	0	0	0	0	0	0	0	0	0	0.1	0	0.22	0	0	0	0	0	0	0	0	0	0	0	0	0	0	0	0	0	0	0	0.63
GTTGCACGGGTTTGTATGTTGCAG	54.85	53.68	40.53	0.37	39.59	47.25	30.52	58.81	0	39.5	15.08	1.02	29.44	80.43	2.34	20.57	154.36	118.73	84.77	65.74	26.46	28.9	234.6	44.8	13.04	14.04	28.16	28.16	32.73	8.76	90.76	90.76	45.19	10.12	174.36	0	0	0	1808.42
GTTGCACGGGTTTGTATGTTGCA	2.48	0.98	0	0	0.26	0.15	0	0	0	2.57	1.31	0.2	1.33	4.77	0	0	0.7	0	0.75	3.46	0	0.33	0	0	0	0	0	0	0	0	0	0	0	0	0	0	0	0	19.29
GTTGCACGGGTTTGTATGTTG	0	0	0.16	0	0.26	0.08	0	0	0	0.2	0.22	0	0.22	0.66	0	0	0.5	0	0.43	3.46	0	0	0	12.22	0	0	0	0	0	0	0	0	0	0	0	0	0	0	18.41
GTTGCACGGGTTTGTATGTT	0	0	0	0	0	0.15	0	0	0	0.99	0.22	0	0	0.33	0	0	2.89	0	2.48	0	0	0	0	0	0	0	0	0	0	0	0	0	0	0	0	0	0	0	7.06
TTGCACGGGTTTGTATGTTGC	1.49	0	0	0	0	0	0	11.76	0	0.2	0	2.65	0.89	0	0	0	0	0	0	0	0	0	0	0	0	0	0	0	0	0	0	0	0	0	0	84.51	11.08	0	112.58
TTGCACGGGTTTGTATGTTGCA	0.99	0	0.08	0	0.53	0	0	0	0	0.79	0.66	1.83	0.89	0.16	0	0	0.2	0	0.32	0	0	0	0	0	0	0	0	0	0	0	0	0	0	0	0	0	0	0	6.45
TTGCACGGGTTTGTATGTTGCAG	0.25	1.22	0.39	0	1.32	0.6	0	0	0	0	0	0.41	0.22	0.16	0	0	3.69	0	0.43	3.46	0.61	0.33	23.46	8.15	0	0	0	0	0	8.76	0	0	0	0	0	0	0	0	53.46
TTGCACGGGTTTGTATGTTG	0.25	0.24	0	0	0	0	0	0	0	0	0	0.81	0	0	0	0	0	0	0	0	0	0	0	0	0	0	0	0	0	0	0	0	0	0	0	89.38	0	5.14	95.82
TTGCACGGGTTTGTATGTT	0.25	0	0	0	0	0	0	0	0	0	0	0.2	0	0	0	0	0	0	0	0	0	0	0	0	0	0	0	0	0	0	0	0	0	0	0	0	0	0	0.45
TGCACGGGTTTGTATGTT	0	0	0	0	0	0	0	0	0	0	0	0	0	0	0	0	0.1	0	0	0	0	0	0	0	0	0	0	0	0	0	0	0	0	0	0	0	0	0	0.1
TGCACGGGTTTGTATGTTGCAG	0	0.24	0	0	0	0	0	0	0	0.2	0	0	0	0	0	0	0.1	0	0	0	0	0	0	4.07	0	0	0	0	0	0	0	0	0	0	0	0	0	0	4.61
TGCACGGGTTTGTATGTTGCA	0.25	0.24	0	0	0	0	0	0	0	0	0	0.2	0	0	0	0	0.1	0	0	0	0	0	0	0	0	0	0	0	0	0	0	0	0	0	0	0	0	0	0.79
GCACGGGTTTGTATGTTGCAG	0	0	0	0	0.26	0	0	0	0	0.39	0.44	0	0.66	0.16	0	0	0	4.09	0	0	0	0	0	0	0	0	0	0	0	0	0	0	0	0	0	0	0	0	6
CACGGGTTTGTATGTTGCAG	0	0	0	0	0	0.15	0	0	0	0.59	0.22	0	0.22	0	0	0	0	4.09	0	0	0	0	0	0	0	0	0	0	10.91	0	0	0	0	0	0	0	0	0	16.18
ACGGGTTTGTATGTTGCAG	0.25	0	0	0	0	0	0	0	0	5.33	1.97	0.41	1.11	0	0	0	0	4.09	0	0	0	0	0	0	0	0	0	0	0	0	0	0	0	0	0	0	0	0	13.16

```
      GTAATATACTAATCCGTGCATCCATCGTATATCTTACACGTCATGTGAACGTGCATCGGTTCATAATGTTGTGTTTATAAATGTTAATGGTATGTGTGGTTGCACGGGTTTGTATGTTGCAG
-47.39 ((((((((.(((((((((.(((.((((((((..(((((.((((((((........)))))))).)).))))).............)))))))))).)))))))))).))))))))..  []
```

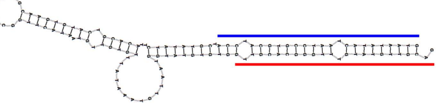

LOC_Os08g09090.1-2

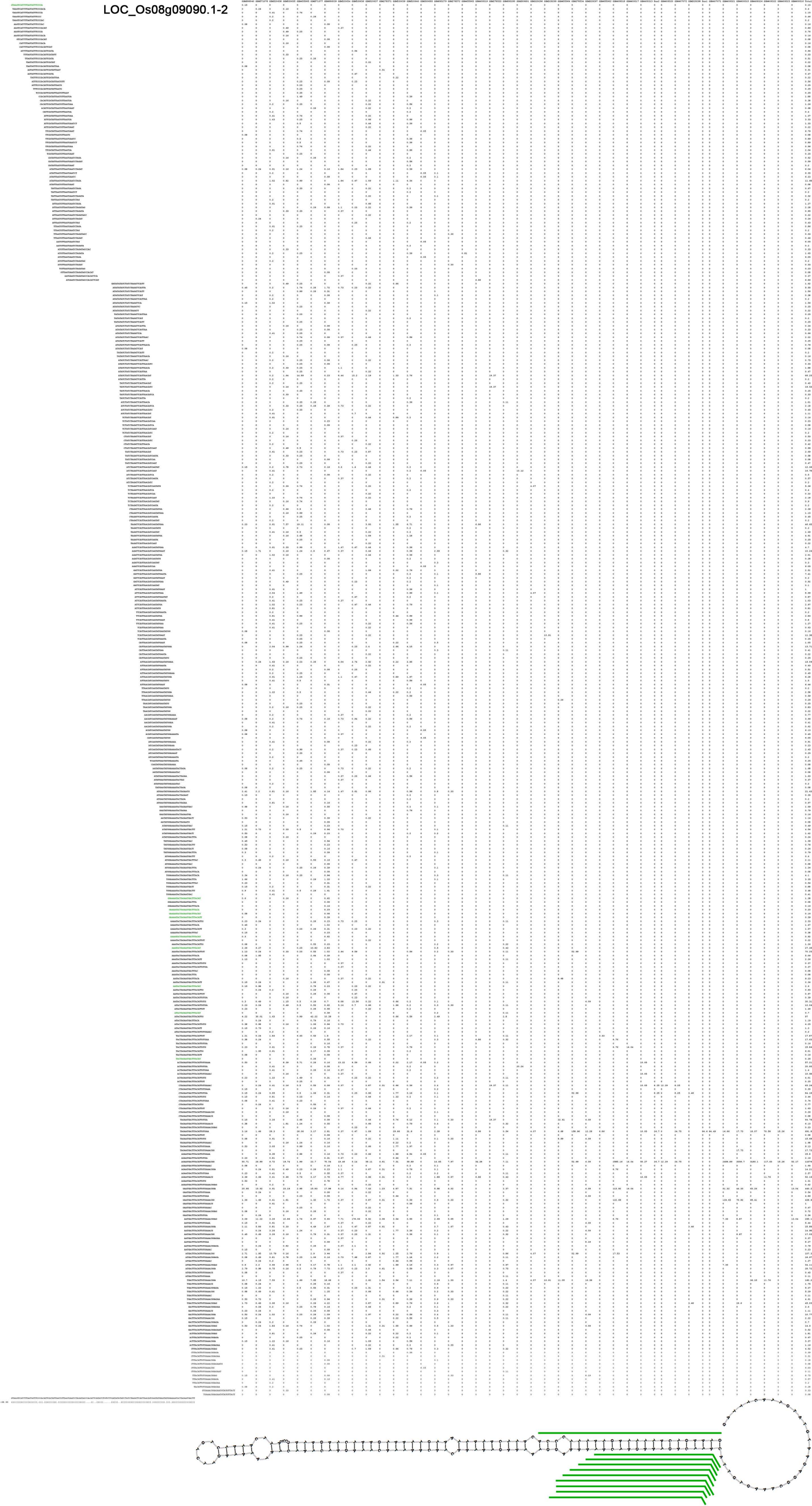

	GSM520637	GSM520638	GSM520640	GSM455965	Total
GTAAGTTGAACAAAATCATCAACA	0.66	0	0	0	0.66
TTGATGCTGATTTTGCGCATTTGTAG	0	0.2	0.59	0.25	1.04
TGATGCTGATTTTGCGCATTTGTAG	0	0.41	0.2	0	0.61
GATGCTGATTTTGCGCATTTGTAG	0.22	0	0.2	0	0.42
GCTGATTTTGCGCATTTGTA	0	0	0.2	0	0.2

LOC_Os08g42590.1-4

```
       GTAAGTTGAACAAAATCATCAACATGGTTCATGCCCCATTTCTTGCAATCTTGATGCTGATTTTGCGCATTTGTAG
-12.80 .((((.((..((((((((.((.((.((((((............)).)))).)).)).))))))))..))))))...   []
```

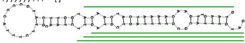

	LOC_Os09g04260.1-13	GSM686040	GSM693279	GSM329296	Total
	CAAGACCAAGTTGATTATGCCG	0.08	0	0	0.08
	ATGCCTTGATCAACCAATCT	0	0.1	0	0.1
	CCTTGATCAACCAATCTGGAG	0	0	8.15	8.15

```
       GTGCACAAATTTCCAAGACCAAGTTGATTATGCCGTACAACATGCCTTGATCAACCAATCTGGAG
-12.20 ...........(((.(((....((((((((.((..........))..))))))))...))))))).  []
```

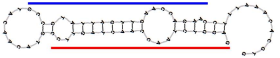

LOC_Os11g26670.1-2

Sequence	GSM686040	GSM686039	GSM278572	GSM455965	GSM520635	GSM520638	GSM693279	GSM571077	Total
GTAGACGAGCAGTTAGTAGGCTT	0.08	0.08	0.33	0	0	0	0	0	0.49
GTAGACGAGCAGTTAGTAGGCTTG	0	0	0	0.25	0	0	0	0	0.25
TAGACGAGCAGTTAGTAGGCTT	0	0	0	0	0.16	0	0	0	0.16
TAGACGAGCAGTTAGTAGGCT	0	0	0	0	0	0.2	0	0	0.2
GACGAGCAGTTAGTAGGCTT	0	0	0	0	0	0	0.1	0	0.1
TCTTCTCGTTTGTTTTTCTGCTAG	0	0	0	0	0	0	0	0.26	0.26

GTAGACGAGCAGTTAGTAGGCTTGCGTGTGTGTTCTTCTCGTTTGTTTTTCTGCTAG

-11.90 (((((.((((((..((.(((..((((....)))..)))))...)))))).))))))... []

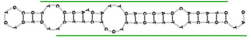

LOC_Os12g18060.1-1

	GSM571077	GSM520635	GSM693279	GSM278571	GSM278572	GSM686040	GSM693280	GSM520638	GSM686039	GSM329298	GSM455965	GSM520636	GSM520640	GSM520639	Total
TTGTAAATAGGAAAGCGCGCT	0.26	0	0	0	0	0	0	0	0	0	0	0	0	0	0.26
TGTAAATAGGAAAGCGCGCTCATC	0	0.16	0.2	0.31	0.33	0	0	0	0	0	0	0	0	0	1
GTAAATAGGAAAGCGCGC	0	0	0	0	0	0.08	0	0	0	0	0	0	0	0	0.08
GTAAATAGGAAAGCGCGCTCATCA	0	0.16	0	0	0	0	0	0	0	0	0	0	0	0	0.16
TAAATAGGAAAGCGCGCTCATCAC	0	0	0.1	0	0	0	0	0	0	0	0	0	0	0	0.1
AAATAGGAAAGCGCGCTCATC	0	0	0	0	0.33	0	0	0	0	0	0	0	0	0	0.33
AAATAGGAAAGCGCGCTCATCACC	0	0	0.1	0	0.33	0	0.11	0	0	0	0	0	0	0	0.54
AATAGGAAAGCGCGCTCATCACCT	0	0	0.1	0	0	0	0	0.2	0	0	0	0	0	0	0.3
ATAGGAAAGCGCGCTCATCACCTT	0	0	0.1	0	0	0	0	0	0	0	0	0	0	0	0.1
ATAGGAAAGCGCGCTCATCACC	0	0	0	0	0	0.08	0	0	0	0	0	0	0	0	0.08
TAGGAAAGCGCGCTCATCACC	0	0	0	0	0	0	0	0	0.23	0	0	0	0	0	0.23
TAGGAAAGCGCGCTCATCACCTT	0	0	0	0	0	0	0	0	0.08	0	0	0	0	0	0.08
AGGAAAGCGCGCTCATCAC	0	0	0	0	0	0.08	0	0	0	3.46	0	0	0	0	3.54
AGGAAAGCGCGCTCATCACCTTTC	0	0	0.1	0	0	0	0.11	0	0	0	0	0	0	0	0.21
AGGAAAGCGCGCTCATCACC	0	0	0	0	0	0	0	0.2	0	0	0	0	0	0	0.2
ACCTTTCTTCAGAACAATAAAGAT	0	0.16	0	0	0	0	0	0	0	0	0	0	0	0	0.16
CTTCAGAACAATAAAGATATCTTT	0	0	0	0	0	0	0	0	0	0	0.25	0	0	0	0.25
AAAGATATCTTTGCGTGGAAACCA	0	0	0	0	0.33	0	0.11	0	0	0	0	0	0	0	0.44
AGATATCTTTGCGTGGAAACCATC	0	0	0	0	0	0	0	0	0	0	0	0.23	0	0	0.23
GGAAACCATCTGATATGCCCGGTAT	0	0	0.1	0	0	0	0	0	0	0	0	0	0	0	0.1
GGAAACCATCTGATATGCCCGGTA	0.26	0.16	0.2	0	0	0.15	0	0	0	0	0	0	0.2	0	0.97
GAAACCATCTGATATGCCCGGTAT	0	0.33	0	0	0	0	0	0	0	0	0	0	0	0.89	1.22
GAAACCATCTGATATGCCCGGTATT	0	0	0	0	0	0.08	0	0	0	0	0	0	0	0	0.08
GAAACCATCTGATATGCCCGGT	0	0	0	0	0	0	0	0.2	0	0	0	0	0	0	0.2
CATCTGATATGCCCGGTATTCCT	0	0	0	0	0	0.08	0	0	0	0	0	0	0	0	0.08
ATCTGATATGCCCGGTATTCC	0	0	0	0	0	0	0	0	0.08	0	0	0	0	0	0.08
ATCTGATATGCCCGGTATTCCTAG	0	0.16	0	0	0	0.08	0	0	0.08	0	0	0	0	0	0.32
GATATGCCCGGTATTCCTAG	0	0	0.1	0	0	0	0	0	0	0	0	0	0	0	0.1

GTTAGGTTGTAAATAGGAAAGCGCGCTCATCACCTTTCTTCAGAACAATAAAGATATCTTTGCGTGGAAACCATCTGATATGCCCGGTATTCCTAG

-15.80 .............((((((..((.((..((((......(((...((..(((((....))))).)).))).......))))..)).))...)))))). []

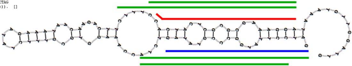

Supplement: Figure S2 — All the mirtrons in Arabidopsis and rice. The short reads perfectly mapped to the mirtron precursors along with their normalized read counts in RPM (reads per million) are shown (see Table S1 for the small RNA HTS data sources and see METHODS in the text for read count normalization). The mature mirtrons with significantly higher expression levels compared to the coordinates on the other arms were highlighted in red color, and the coordinates were in blue. For the mirtron precursors generating mirtrons with indistinguishable expression levels on both arms, their mature mirtrons were highlighted in green color. The mature mirtrons and their coordinates were also indicated in the stem-loop structures of their precursors. The parenthesis-dot formed secondary structure expression along with the free energy, and the stem-loop structures were all predicted and generated by RNAshapes (Steffen et al., 2006). (PDF) [file pone.0031163.s002.pdf]
