# Supplementary material for: Large-Scale Identification of Mirtrons in Arabidopsis and Rice
Source: PLoS One. 2012 Feb 13;7(2):e31163. doi: 10.1371/journal.pone.0031163 (PMC3278437; doi:10.1371/journal.pone.0031163)
Supplement: Table S1 — Plant small RNA high-throughput sequencing data sets used in this study. (PDF) [file pone.0031163.s005.pdf]

Table S1 Plant small RNA high-throughput sequencing data sets used in this study

Species	Data sets	Sources
Arabidopsis thaliana	GSM118372; GSM118373; GSM118374; GSM118375; GSM120717; GSM121453; GSM121454; GSM121455; GSM121456; GSM121457; GSM149079; GSM149080; GSM149081; GSM154336; GSM154361; GSM154362; GSM154363; GSM154364; GSM154365; GSM154367; GSM154368; GSM154370; GSM154375; GSM154376; GSM154377; GSM253622; GSM253623; GSM253624; GSM253625; GSM257235; GSM257236; GSM257237; GSM304282; GSM304283; GSM304284; GSM304285; GSM338557; GSM342999; GSM343000; GSM343001; GSM343002; GSM343004; GSM343005; GSM366865; GSM366866; GSM366867; GSM366868; GSM366869; GSM366870; GSM442932; GSM442933; GSM442934; GSM442935; GSM456945; GSM518432; GSM642335; GSM642336; GSM642337; GSM642338	GEO[a]
Oryza sativa	GSM278532; GSM278533; GSM278534; GSM278535; GSM278571; GSM278572; GSM309691; GSM309692; GSM309693; GSM329296; GSM329297; GSM329298; GSM329299; GSM407071; GSM407072; GSM409313; GSM409314; GSM409315; GSM409316; GSM409317; GSM409318; GSM409319; GSM409320; GSM409321; GSM409322; GSM409323; GSM409324; GSM455962; GSM455963; GSM455964; GSM455965; GSM520634; GSM520635; GSM520636; GSM520637; GSM520638; GSM520639; GSM520640; GSM571077; GSM571078; GSM686039; GSM686040; GSM693279; GSM693280	GEO[a]
	Run1(Aerial Leaf Seedling); Run2(adult stress kinase)	CSRDB[b]

[a]GEO (Gene Expression Omnibus), http://www.ncbi.nlm.nih.gov/geo/
[b]CSRDB (Cereal Small RNAs Database), http://sundarlab.ucdavis.edu/smrnas/
