# Supplementary material for: Large-Scale Identification of Mirtrons in Arabidopsis and Rice
Source: PLoS One. 2012 Feb 13;7(2):e31163. doi: 10.1371/journal.pone.0031163 (PMC3278437; doi:10.1371/journal.pone.0031163)
Supplement: Table S2 — Plant degradome sequencing data sets used in this study. (PDF) [file pone.0031163.s006.pdf]

Table S2 Plant degradome sequencing data sets used in this study

Species	Data sets	Sources
Arabidopsis thaliana	GSM278333; GSM278334; GSM278335; GSM278370	GEO[a]
	AxIDT; AxIRP; AxSRP; Col; ein5l; TWF; Tx4F	NGSDBs[b]
Oryza sativa	GSM434596; GSM455938; GSM455939; GSM476257	GEO[a]

[a]GEO (Gene Expression Omnibus), http://www.ncbi.nlm.nih.gov/geo/
[b]NGSDBs (Next-Gen Sequence Databases), http://mpss.udel.edu/
